# Global, regional, and national trends in chronic kidney disease burden (1990–2021): a systematic analysis of the global burden of disease in 2021

**DOI:** 10.1186/s41182-025-00703-x

**Published:** 2025-02-21

**Authors:** Jiaxi Chen, Miao Deng, Rubin Zheng, Yanjin Chen, Wenyi Pang, Ziyang Zhang, Zhouke Tan, Zhixun Bai

**Affiliations:** 1 Clinical College, Zunyi Medical University, Zunyi, 563000 Guizhou China; 2 Department of Nephrology, The Affiliated Hospital of Zunyi Medical University, Zunyi, 563006 Guizhou China; 3 Organ Transplant Center, The Affiliated Hospital of Zunyi Medical University, Zunyi, 563006 Guizhou China; 4 Department of Nephrology, People’s Hospital of Qianxinan Prefecture, Xingyi, 562400 Guizhou China

**Keywords:** Chronic kidney disease, Incidence, Mortality, Global Burden of Disease

## Abstract

**Background:**

Chronic kidney disease (CKD) is a global health challenge with diverse etiologies. However, research on the incidence trends of CKD attributable to specific causes remains limited, and the incidence and mortality rates vary across regions. This study aims to identify the patterns and temporal trends of CKD incidence, providing valuable information for the development of targeted prevention strategies and interventions.

**Methods:**

CKD data from the 2021 Global Burden of Disease Study (1990–2021) were analyzed by sex, region, country, cause, and Socio-demographic Index (SDI). Disease burden was assessed using age-standardized incidence (ASIR), mortality rates (ASMR), and estimated annual percentage changes (EAPC). Decomposition analysis evaluated population aging, growth, and epidemiological impacts. The autoregressive integrated moving average (ARIMA) model was used to predict the burden of CKD from 2021 to 2031, and the age-period-cohort (APC) model was employed to assess the effects of age, time, and cohort. Health inequality was analyzed using Slope Index of Inequality (SII) and Concentration Index (CI).

**Results:**

In 2021, Saudi Arabia had the highest ASIR, while Mauritius had the highest mortality. China and India contributed the most cases and deaths. ARIMA forecasts CKD cases will rise to 22.21 million and deaths to 1.81 million by 2031. Epidemiological changes drove incidence growth in medium SDI regions and mortality in high SDI regions. EAPC correlated with ASIR and ASMR. APC analysis showed incidence peaked between 70–80 years, with earlier cohorts facing higher risks. Unknown causes, type 2 diabetes, and hypertension were the leading CKD etiologies. From 1990–2021, health inequality in CKD incidence and mortality worsened, especially in high SDI regions, where the mortality CI shifted from 0.05 to − 0.09.

**Conclusions:**

This study estimated the temporal trends of CKD incidence and mortality globally, as well as at the national and regional levels, from 1990 to 2021. It was observed that countries with higher socio-demographic index (SDI) exhibited unfavorable trends, suggesting that these countries should develop more targeted and specific strategies to address the growing burden of CKD.

**Supplementary Information:**

The online version contains supplementary material available at 10.1186/s41182-025-00703-x.

## Introduction

Chronic kidney disease (CKD) is a prevalent condition globally, with an increasing incidence trend [1]. Between 1990 and 2017, the all-age mortality rate due to CKD increased by 41.5% worldwide [2]. Over the past decades, the burden of CKD has continued to grow [3], with predictions suggesting that the number of individuals requiring kidney replacement therapy (KRT) will double by 2030 compared to 2010 [4]. The incidence of CKD remains poorly defined due to reliance on cohort studies that screen heterogeneous populations, use different formulas to estimate glomerular filtration rate (GFR), and employ varied methods to measure proteinuria [5]. Despite these limitations, the prevalence of CKD in high-income countries such as the United States and Australia has consistently been reported at approximately 11%. The incidence and progression of CKD vary by race and socioeconomic status, with higher risks reported among Black and Asian individuals in the UK, Hispanic populations in the US, and Indigenous populations in Australia, New Zealand, and Canada [6].

The etiology of CKD is not fully understood. On one hand, CKD has a bidirectional relationship with aging. Despite the clear diagnostic criteria and implications of a CKD diagnosis, it remains an underrecognized condition, particularly among the elderly [7, 8]. On the other hand, diseases such as diabetes, hypertension, and glomerulonephritis, as well as nephrotoxic exposures from certain herbal medicines, drug interactions, heavy metals, and organic pollutants like pesticides, contribute to CKD development and progression [9–14]. Importantly, the Kidney Disease: Improving Global Outcomes (KDIGO) organization recently emphasized the role of genetics in CKD classification and management, recommending genetic testing to enhance diagnostic accuracy and facilitate personalized medical care for kidney disease [15]. Understanding the patterns and temporal trends of CKD incidence can aid in designing targeted preventive strategies and advancing precision prevention of CKD.

The Global Burden of Disease (GBD) study assessed the burden of 371 diseases and injuries across 204 countries and territories, including CKD and its specific etiologies, providing an opportunity to understand the current state of CKD [16]. This study retrieved detailed data on CKD incidence and mortality attributed to five specific causes from the GBD database and further evaluated the global burden of CKD by examining the temporal trends of CKD incidence and mortality due to these specific causes from 1990 to 2021, at global, national, and regional levels. The findings of this study serve as an important extension and complement to previous research on CKD burden [17], and also contribute to the design of targeted CKD prevention strategies for different countries and regions.

## Materials and methods

### Study data sources

Annual data on CKD incidence, age-standardized incidence rate (ASIR), and mortality from 1990 to 2021 were obtained from the Global Health Data Exchange (GHDx) query tool (http://ghdx.healthdata.org/gbd-results-tool). The data were categorized by sex, region, country, and etiology, including type 1 diabetes, type 2 diabetes, hypertension, glomerulonephritis, and other causes. Data from 204 countries and regions were available, further classified into five SDI-based groups (low, low-middle, middle, high-middle, and high) and 21 geographical regions. The GBD CKD Collaborators provided standardized methods for processing and modeling CKD prevalence and mortality data, using eGFR and ACR as indicators to define CKD [2]. Despite some limitations (e.g., the “Both” category in the GBD dataset is not simply the sum of data from the “Male” and “Female” categories, but rather a weighted average based on global gender ratios and population distribution, which may lead to discrepancies in reflecting gender differences in certain analyses), the comprehensiveness and broad coverage of the GBD database make it one of the most important resources for global health research. Detailed descriptions of the GBD data have been provided in previous studies [16, 18], and thus will not be further elaborated here. The summary data used in this study are publicly available and can be freely downloaded.

### Statistical analysis

#### Quantification of CKD burden

This study quantified CKD burden and its etiology using ASIR, age-standardized mortality rate (ASMR), and estimated annual percentage change (EAPC). Standardization is necessary when comparing populations with different age structures or the same population over time.

The ASR (per 100,000 population) in accordance with the direct method is calculated by summing up the products of the age-specific rates (a_i_, where I denotes the ith age class) and the number of persons (or weight) (w_i_) in the same age subgroup I of the chosen reference standard population, then dividing the sum of standard population weights, i.e.,

$$ASR = \frac{{\mathop \sum \nolimits_{{\left\{ {i = 1} \right\}}}^{A} a_{i} w_{i} }}{{\mathop \sum \nolimits_{{\left\{ {i = 1} \right\}}}^{A} w_{i} }} \times 100{,}000$$

EAPC represents the average annual change in ASR over a specified period. It is derived from a regression model fitting the natural logarithm of ASR (y = ln(ASR),) against calendar year (χ): EAPC = 100 × (exp(β) − 1). The 95% confidence interval (CI) for EAPC is obtained from the linear regression model [19]. An EAPC estimate and its 95% CI lower bound > 0 indicate an increasing ASR trend; if the upper bound is < 0, ASR is considered to be decreasing; otherwise, ASR is stable over time. The association between EAPC and ASR was evaluated at the national level.

### Decomposition analysis

Decomposition analysis considers the contributions of population size, age structure, and epidemiologic changes—specifically referring to changes in age- and population-standardized incidence and deaths. These epidemiologic changes reflect the evolving patterns of CKD incidence and outcomes over time, independent of demographic shifts [20].

### Autoregressive integrated moving average model

The autoregressive integrated moving average (ARIMA) model combines autoregressive (AR) and moving average (MA) components. It assumes time-series data as stochastic variables correlated with time, which the ARIMA model represents. The ARIMA model predicts future values based on past observations (autoregressive component) and error terms (moving average component). The ARIMA equation is expressed as: Y_t_ = φ_1_Y_t−1_ + φ_2_Y_t−2_ + ⋯+ φ_p_Y_t-p_ + e_t_ − θ_1_e_t−1_−⋯−θ_q_e_t−q_, where (φ_1_Y_t−1_ + φ_2_Y_t−2_ + ⋯+ φ_p_Y_t−p_ + e_t_) represents the AR component, and (e_t_ − θ_1_e_t−1_−⋯−θ_q_e_t-q_) represents the MA component. In the ARIMA model, Y_t−1_ represents the observation at time t–p, where φ_1_, φ_2_, …, φ_p_ are the parameters of the autoregressive (AR) model, indicating the linear relationship between the current value and the past p data points. θ_1_, θ_2_, …, θ_q_ are the parameters of the moving average (MA) model, representing the influence of error terms on the current prediction. p and q denote the order of the AR and MA models, respectively, and e_t_ is the random error term at time t [21]. In the ARIMA model, the time series should be a stationary random sequence with zero mean. The orders of the AR and MA models are determined through the autocorrelation function (ACF) and partial autocorrelation function (PACF). The model is evaluated using residual analysis and the Akaike Information Criterion (AIC), with the model having the smallest AIC value selected to ensure good fit and high predictive accuracy.

### Age-period-cohort model

The age-period-cohort (APC) model examines the effects of age, period, and birth cohort on health outcomes. Age effect refers to the risk of outcomes at different age groups; period effect captures the influence of temporal changes on outcomes across all age groups; cohort effect represents variations in outcomes among participants from the same birth cohort. It uses a log-linear regression model: log(Y_i_) = μ + α*age_i_ + β*period_i_ + γ*cohort_i_ + ε, where Y_i_ is the CKD incidence or mortality rate, α, β and γ are coefficients for age, period, and cohort, respectively, μ is the intercept, ε is the residual. Intrinsic estimations (IE) combined with APC models yielded net effects for all three dimensions [22].

### Cross-country inequalities analysis

Inequalities were measured using the Slope Index of Inequality (SII) and the Concentration Index (CI), which assess absolute and relative inequality gradients, respectively. SII regresses ASIR or ASMR against a country’s relative SDI position, defined as the midpoint of its population in a cumulative distribution sorted by SDI [23]. Weighted regression models accounted for heteroscedasticity. CI quantified the area under the Lorenz curve, aligning cumulative proportions of ASIR or ASMR with the population sorted by SDI [24].

The above statistical analyses were performed using R software (version 4.3.0), and results were considered statistically significant when P value < 0.05.

## Result

### Global CKD burden

**Fig. 1:**
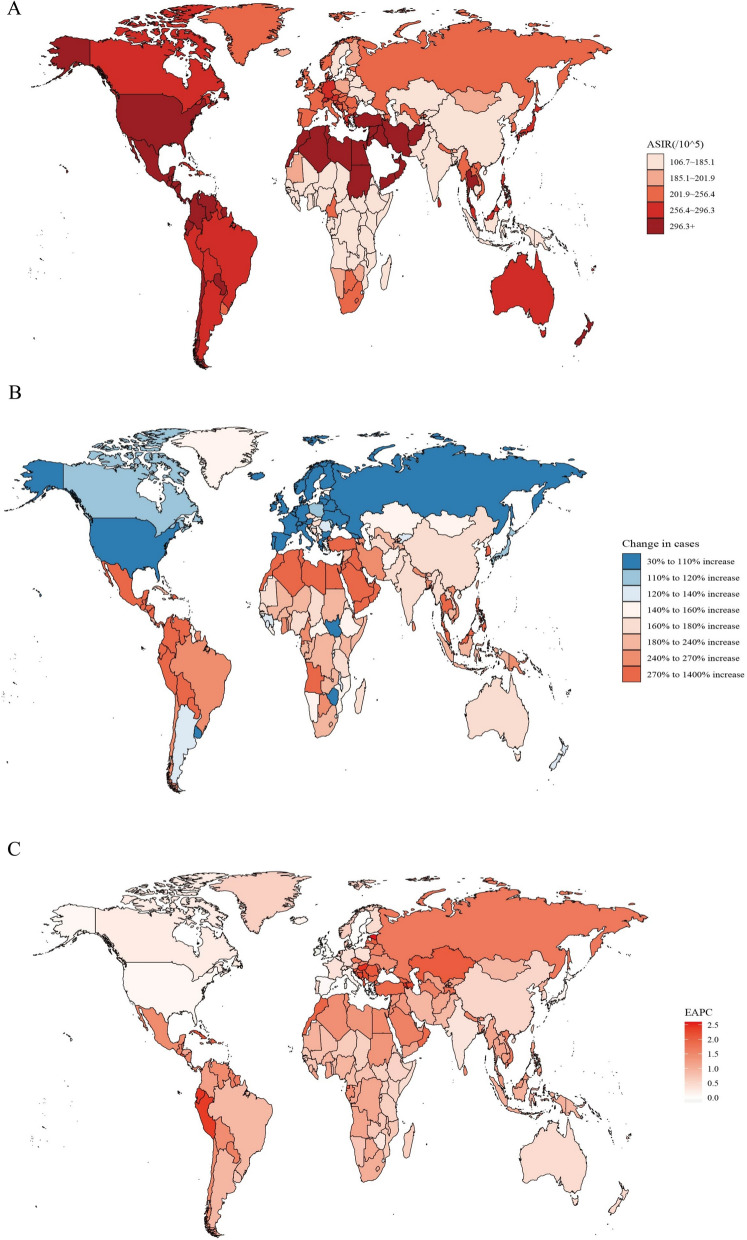
The global disease burden of CKD for both sexes in 204 countries and territories. **A** The ASIR of CKD in 2021; **B** the relative change in incident cases of CKD between 1990 and 2021; **C** the EAPC of CKD ASIR from 1990 to 2021. *CKD* chronic kidney disease, *ASIR* age standardized incidence rate, *EAPC* estimated annual percentage change

There was substantial variation in the age-standardized incidence rate (ASIR) and age-standardized mortality rate (ASMR) of CKD across the world in 2021 (Fig. 1; Figure S1). The highest ASIR was observed in Saudi Arabia (495.8 per 100,000 population), followed by Qatar (467.4 per 100,000) and the United Arab Emirates (466.4 per 100,000). In absolute numbers, China had the highest number of CKD incident cases in 2021 (3,323,175 cases), followed by India (2,235,892 cases) and the United States (1,842,212 cases) (Table S1). The highest ASMR in 2021 was observed in Mauritius (80.1 per 100,000), followed by Saudi Arabia (79.3 per 100,000) and American Samoa (73.8 per 100,000). Similarly, in absolute mortality numbers, China had the highest CKD deaths in 2021 (204,230.2 deaths), followed by India (175,637.1 deaths) and the United States (135,879.9 deaths) (Table S2).

**Fig. 2:**
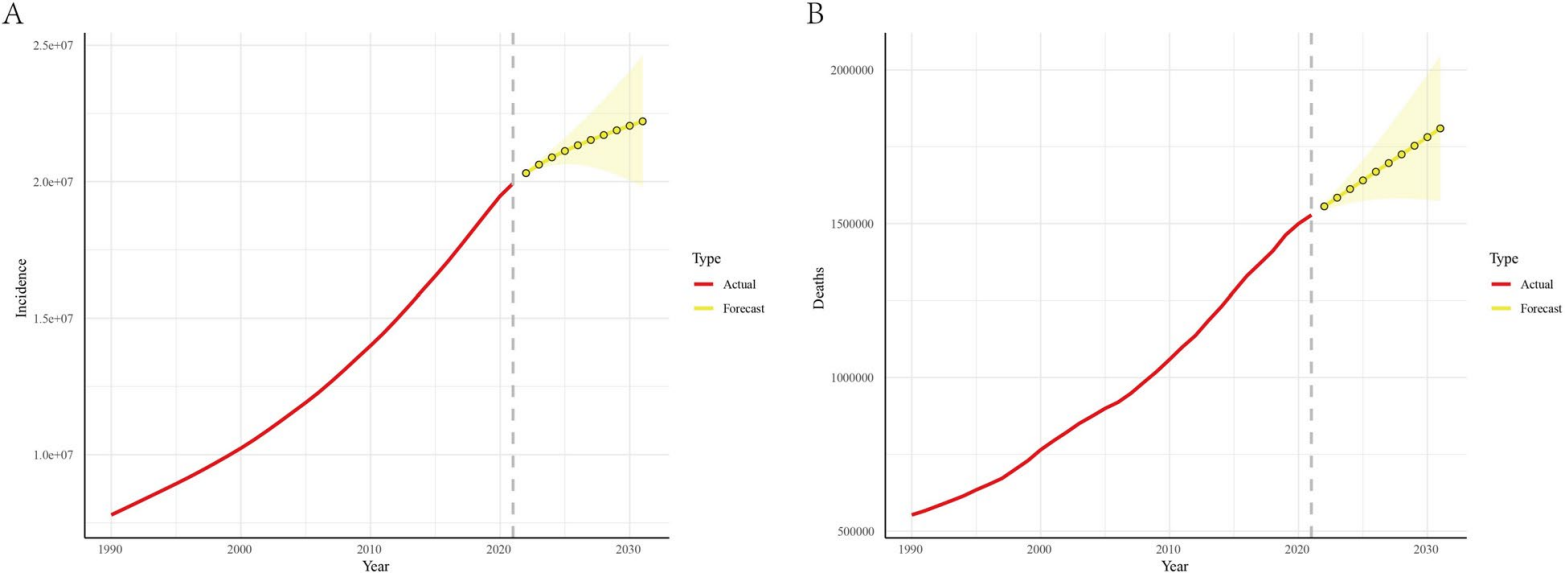
Predicted trends of CKD incidence (**A**) and deaths (**B**) in the next decade (2021–2031). Red lines represent the true trend of CKD incidence and deaths during 1990–2021; yellow dot lines and shaded regions represent the predicted trend and its 95% CI

Projections from the ARIMA model indicated that CKD incidence would increase from 19,935,037.8 cases in 2021 to 22,210,613.5 cases in 2031, with CKD-related deaths also predicted to rise from 1,527,638.7 in 2021 to 1,809,532.6 in 2031 (Fig. 2; Table S3).

### Decomposition analysis of age-standardized incidence and death numbers

**Fig. 3:**
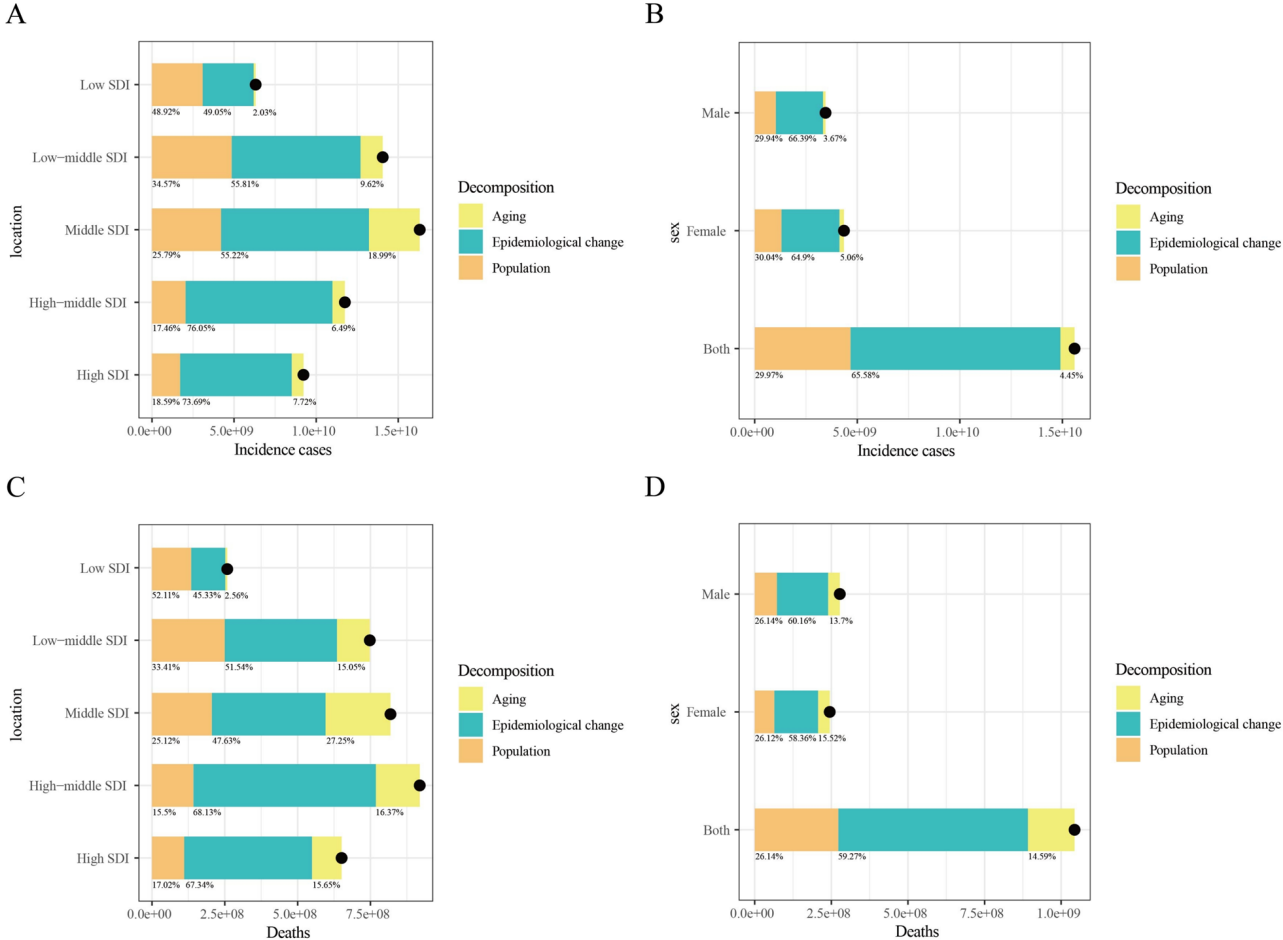
Changes in CKD incidence and CKD-related mortality from 1990 to 2021 according to population-level determinants of population growth, aging, and epidemiological change across different socio-demographic index quintiles and by sex. **A** Decomposition of CKD incidence by SDI quintile; **B** decomposition of CKD incidence by sex; **C** decomposition of CKD-related mortality by SDI quintile; **D** decomposition of CKD-related mortality by sex. The black dot represents the overall value of change contributed by all three components. Epidemiologic changes refer to the evolving patterns of CKD incidence and outcomes, independent of demographic shifts, as reflected in age- and population-standardized incidence and mortality. SDI, socio-demographic index

Between 1990 and 2021, the greatest increase in CKD incidence was observed in middle-SDI quintile regions (Fig. 3A). Epidemiological changes contributed the most to this increase (55.22%), followed by population growth (25.79%). Similar trends were observed in sex-stratified analyses (Fig. 3B), with epidemiological changes and population growth accounting for 64.90% and 30.04% of the increase in CKD incidence, respectively. For CKD-related deaths, the largest increase occurred in high-SDI quintile regions (Fig. 3C), where epidemiological changes accounted for 68.13% of the increase. In sex-stratified analyses (Fig. 3D), epidemiological changes contributed 59.27% of CKD-related deaths (Table S4).

### The influential factor for EAPC

**Fig. 4:**
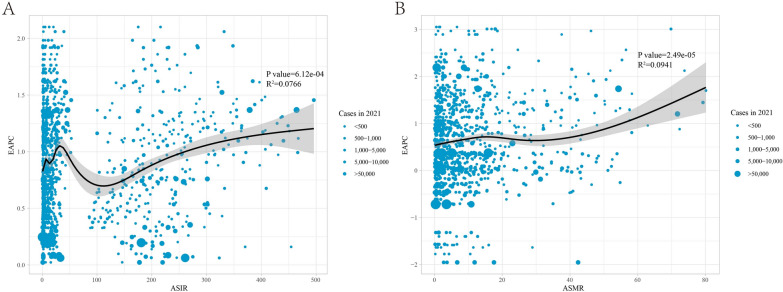
The correlation between estimated annual percentage change and **A** age-standardized incidence rate and **B** age-standardized mortality rate. Circles represent CKD cases in 2021, with larger circles indicating higher cases. The R^2^ and P values were derived from Pearson correlation analysis. *EAPC* estimated annual percentage change, *ASIR* age-standardized incidence rate, *ASMR* age-standardized mortality rate, *CKD* chronic kidney disease

A significant association was observed between EAPC and both ASIR and ASMR in 2021 (P < 0.05). EEAPC showed a slight upward trend with increasing ASIR, exhibiting some non-linear characteristics (P = 6.12E−04, R^2^ = 0.0766). Similarly, EAPC increased with higher ASMR (P = 2.49E−05, R^2^ = 0.0941) (Fig. 4).

### Age, period and cohort effects on CKD incidence and CKD-related mortality

**Fig. 5:**
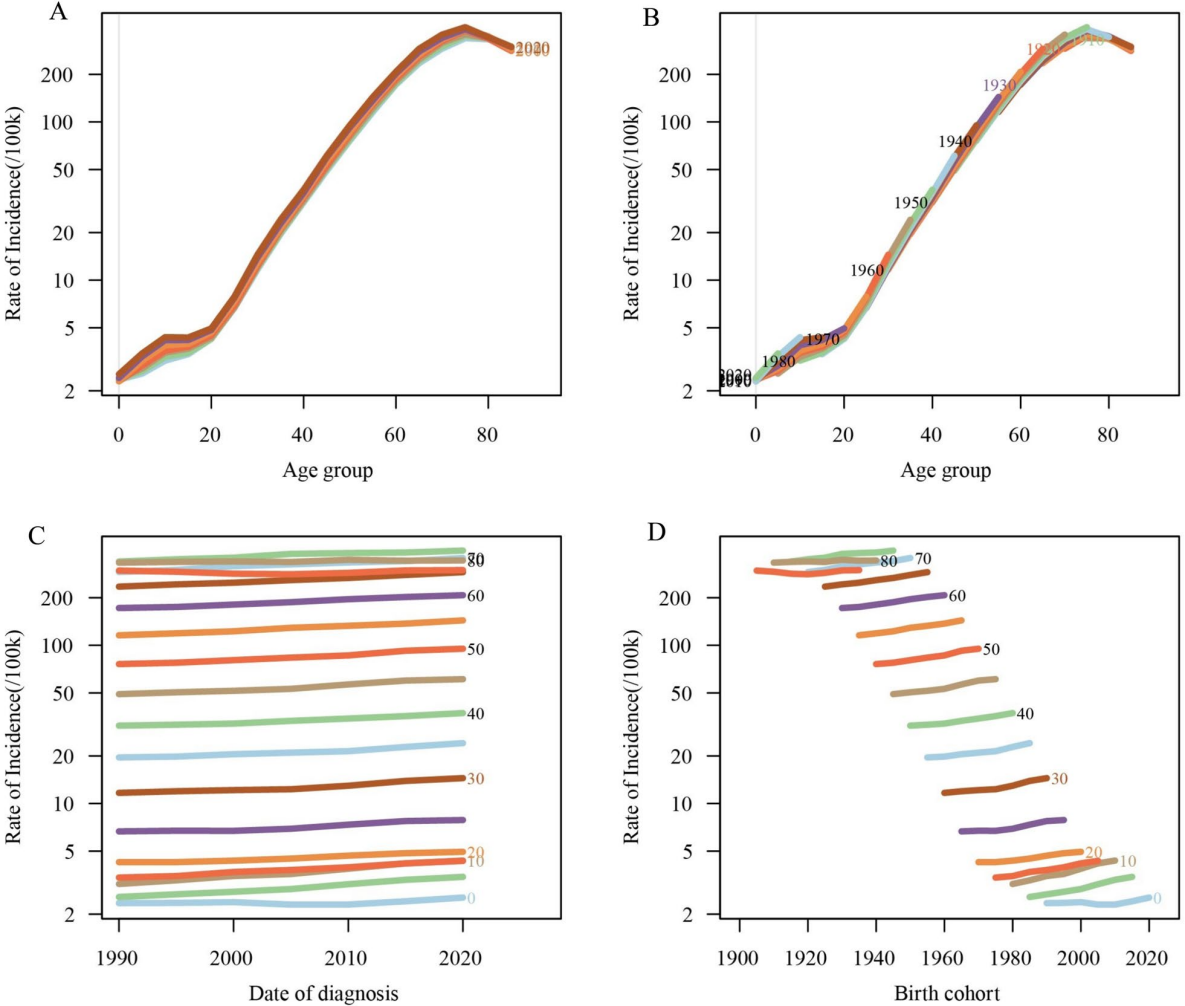
Trends in CKD incidence globally by age, period, and cohort. **A** Age-specific CKD incidence rates by year; **B** age-specific CKD incidence rates by birth cohort; **C** period-specific CKD incidence rates across age groups; **D** cohort-specific CKD incidence rates by birth cohort

**Fig. 6:**
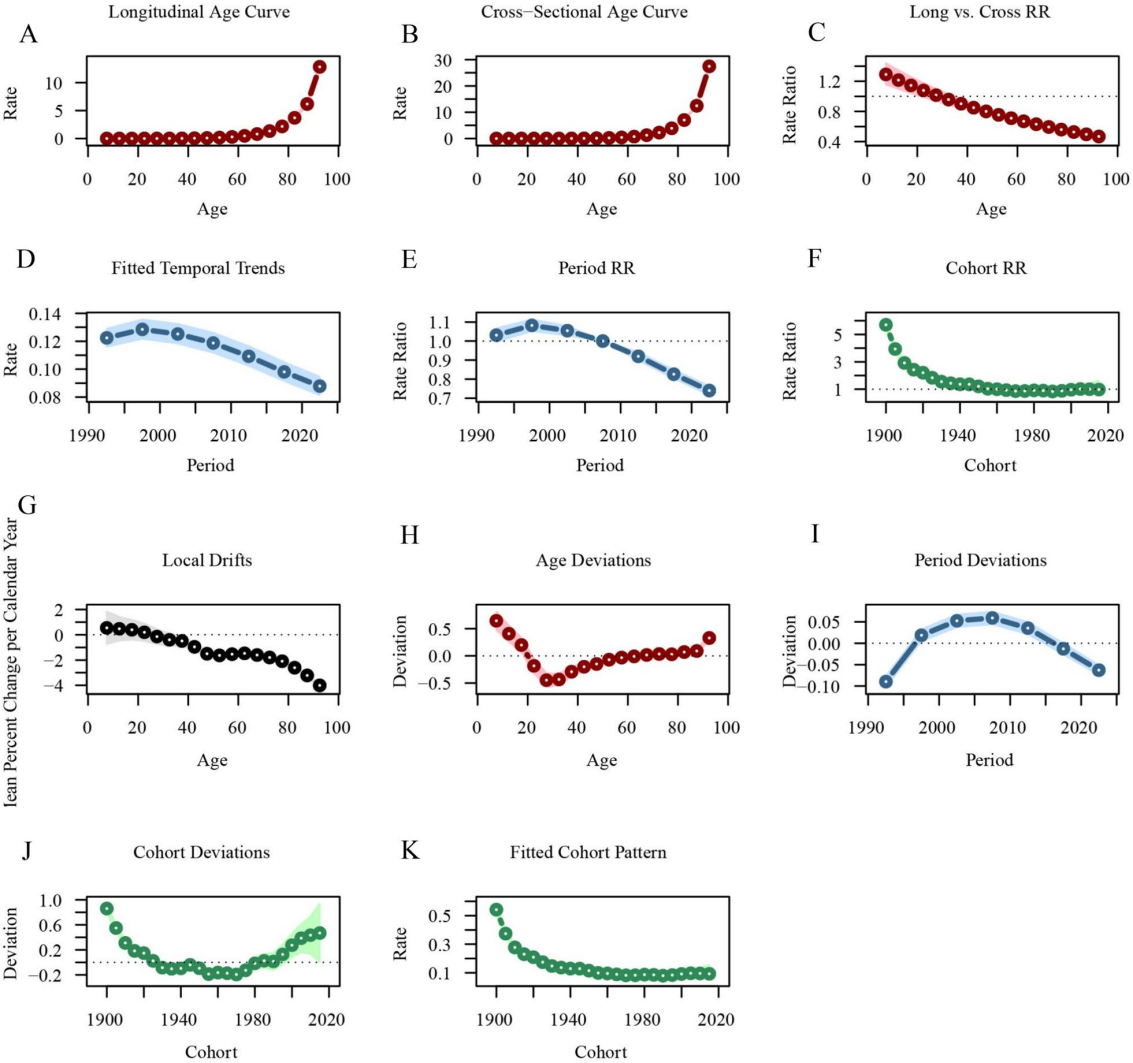
Age-period-cohort analysis of global CKD incidence from 1990 to 2021. **A** Longitudinal age curve of CKD incidence; **B** cross-sectional age curve of CKD incidence; **C** comparison of longitudinal vs. cross-sectional rate ratios; **D** fitted temporal trends in CKD incidence; **E** period rate ratios over time; **F** cohort rate ratios by birth cohort; **G** local drifts in CKD incidence by age; **H** age deviations from the fitted model; **I** period deviations from the fitted model; **J** cohort deviations from the fitted model; **K** fitted cohort pattern of CKD incidence

Figures 5 and 6 illustrate the age-period-cohort effects on CKD incidence. CKD incidence increases with age, peaking between 70–80 years. CKD incidence showed a slight upward trend over time. Earlier birth cohorts exhibited relatively higher CKD incidence across all age groups, while more recent birth cohorts had lower CKD incidence. The relative risk (RR) decreased from 1.03 (95%CI 1.00–1.07) in 1992.5 to 0.74 (95%CI 0.72–0.76) in 2022.5. The RR for CKD incidence was significantly higher for early cohorts (RR_cohort(1900)_ = 5.70, 95%CI 5.35–6.09) and lower for recent cohorts (RR_cohort(2015)_ = 0.98, 95%CI 0.59–1.62) (Table S5).

**Fig. 7:**
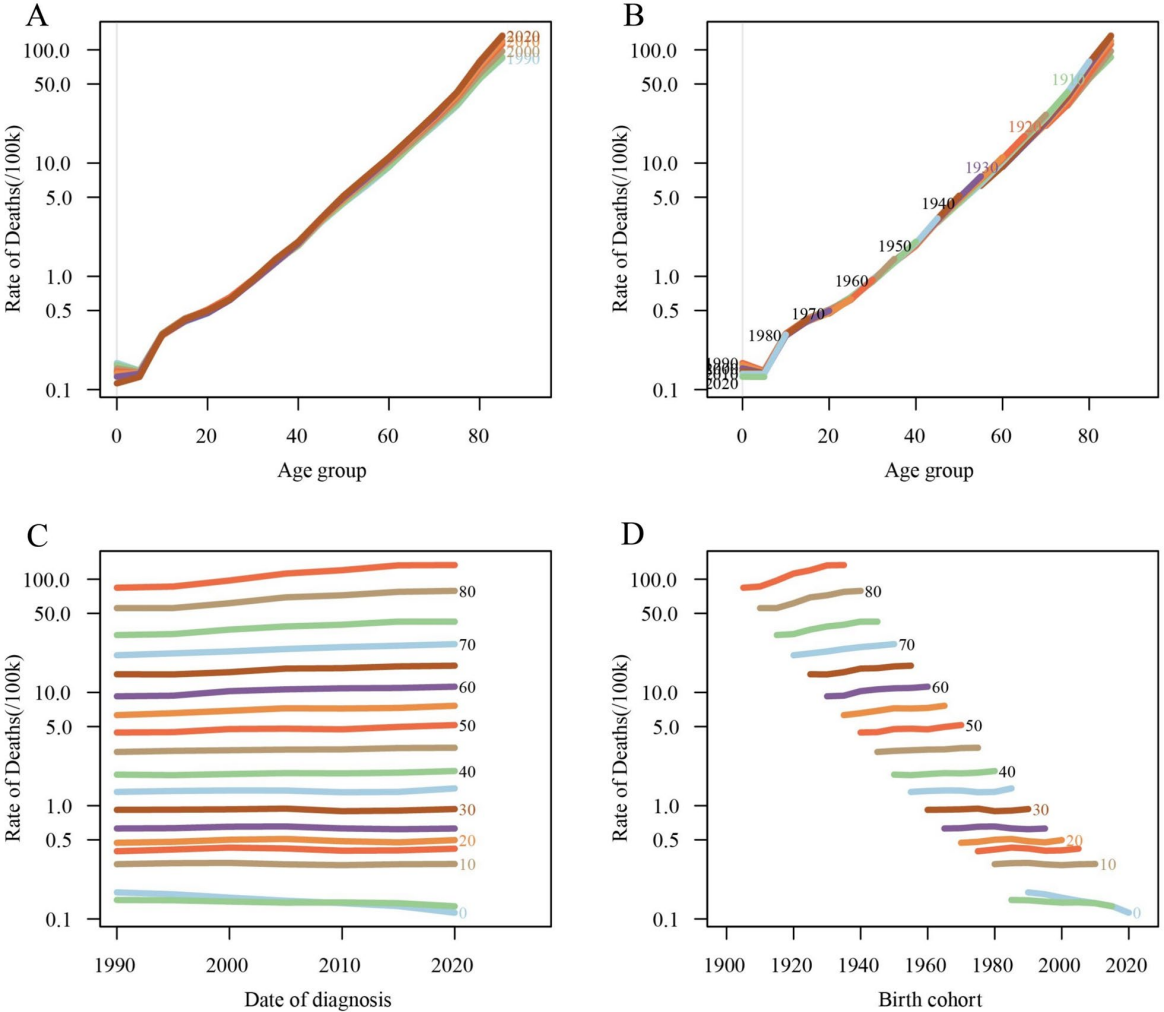
Trends in CKD-related mortality globally by age, period, and cohort. **A** Age-specific CKD-related mortality by year; **B** age-specific CKD-related mortality by birth cohort; **C** period-specific CKD-related mortality across age groups; **D** cohort-specific CKD-related mortality by birth cohort

**Fig. 8:**
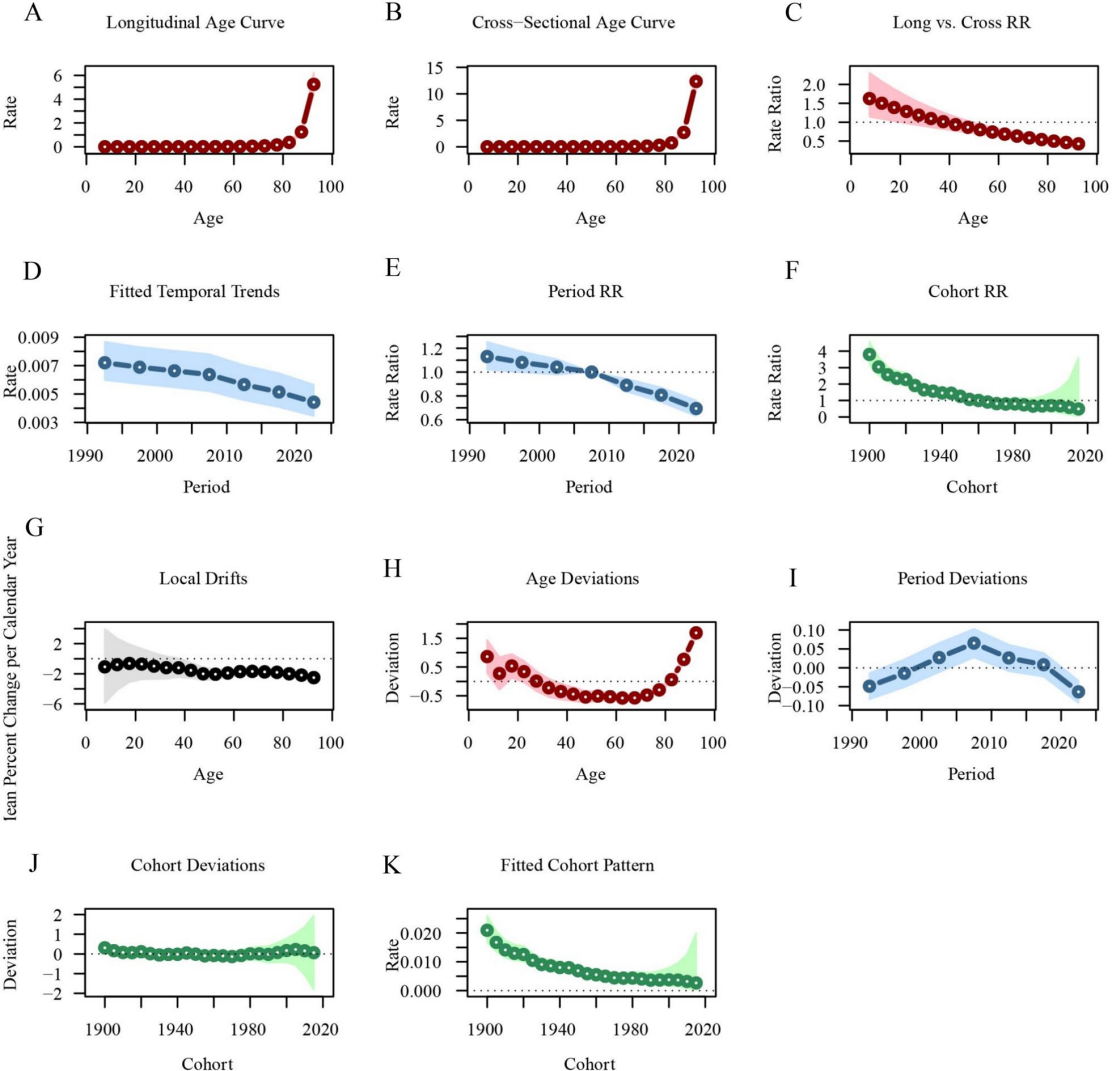
Age-period-cohort analysis of global CKD-related mortality from 1990 to 2021. **A** Longitudinal age curve of CKD-related mortality; **B** cross-sectional age curve of CKD-related mortality; **C** comparison of longitudinal vs. cross-sectional rate ratios; **D** fitted temporal trends in CKD-related mortality; **E** period rate ratios over time; **F** cohort rate ratios by birth cohort; **G** local drifts in CKD-related mortality by age; **H** age deviations from the fitted model; **I** period deviations from the fitted model; **J** cohort deviations from the fitted model; **K** fitted cohort pattern of CKD-related mortality

Similarly, CKD mortality increased with age, while remaining relatively stable across all age groups. However, CKD mortality rates for more recent birth cohorts showed a noticeable decline (Fig. 7). Longitudinal age curves indicate that CKD mortality rises slowly among individuals below 70 years of age (RR_age(72.5)_ = 0.08, 95%CI 0.06–0.09) but increases significantly for those over 70 years (RR_age(92.5)_ = 5.24, 95%CI 4.43–6.20). CKD mortality rates have shown a steady decline over time (RR_period(2022.5)_ = 0.69, 95%CI 0.63–0.76). Earlier birth cohorts experienced relatively higher CKD mortality rates (RR’_cohort(1900)_ = 3.80, 95%CI 3.17–4.56), whereas more recent cohorts exhibited significantly lower mortality rates (RR’_cohort(2015)_ = 0.48, 95%CI 0.07–3.60) (Fig. 8; Table S5).

### The distribution changes of CKD caused by different causes in different regions and periods

**Fig. 9:**
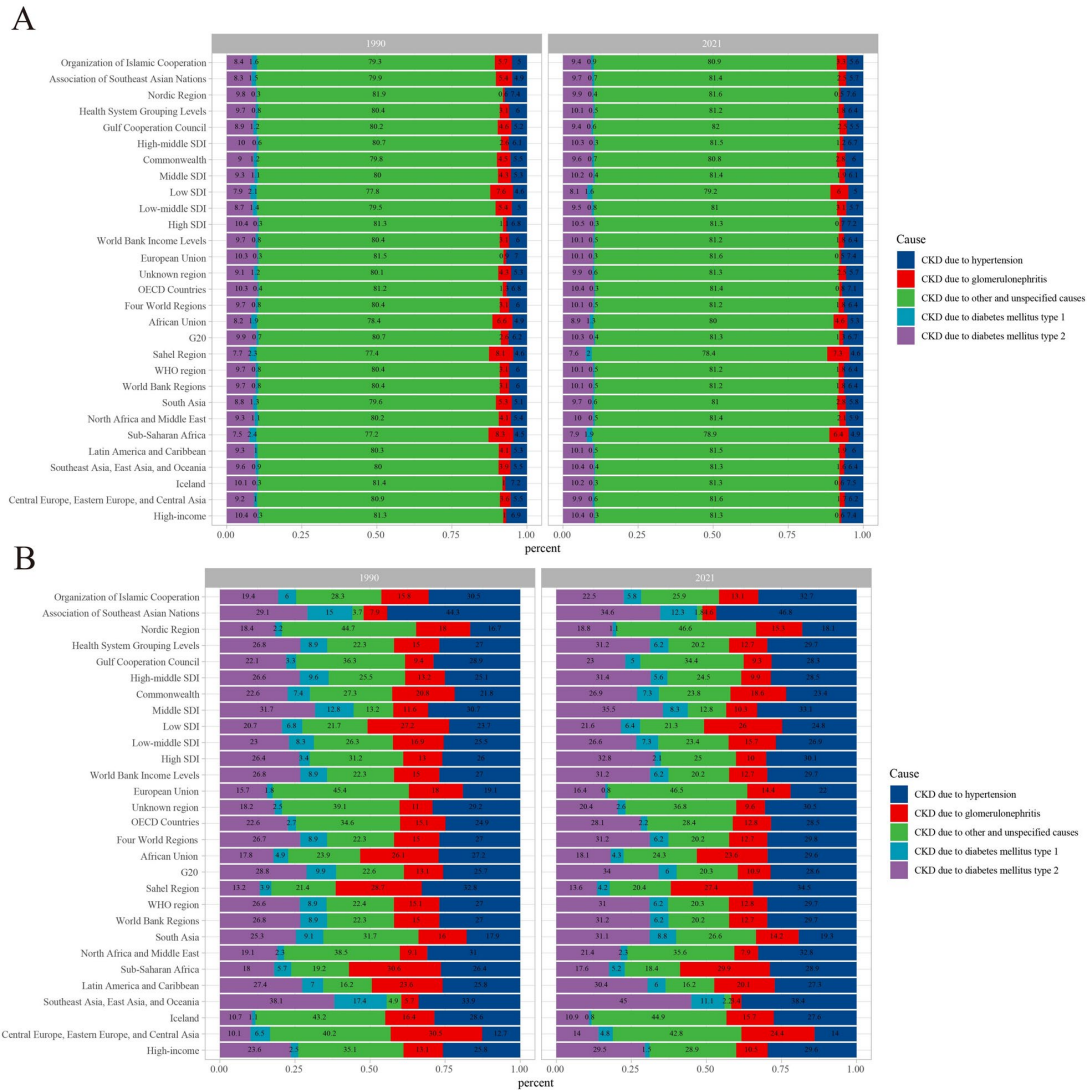
Contribution of different causes to CKD incident cases (**A**) and deaths (**B**), both sexes and by region, in 1990 and 2021. CKD, chronic kidney disease

The global proportions of CKD attributable to specific causes in 1990 and 2021 are as follows: nearly 80% of CKD cases were due to unknown causes, and notably, this distribution remained almost unchanged between 1990 and 2021. CKD caused by type 2 diabetes and hypertension ranked second, while type 1 diabetes accounted for the lowest proportion. Additionally, CKD attributable to glomerulonephritis showed a decline compared to 1990 (Fig. 9A). In contrast, there were notable regional differences in the proportions of CKD-related deaths caused by specific etiologies between 1990 and 2021. In both years, over 40% of CKD-related deaths in the Nordic Region, European Union, Central Europe, Eastern Europe, Central Asia, and Iceland were due to unknown causes. Meanwhile, in the Association of Southeast Asian Nations and Southeast Asia, East Asia, and Oceania, the proportion of CKD-related deaths due to unknown causes was below 5% (Fig. 9B).

**Fig. 10:**
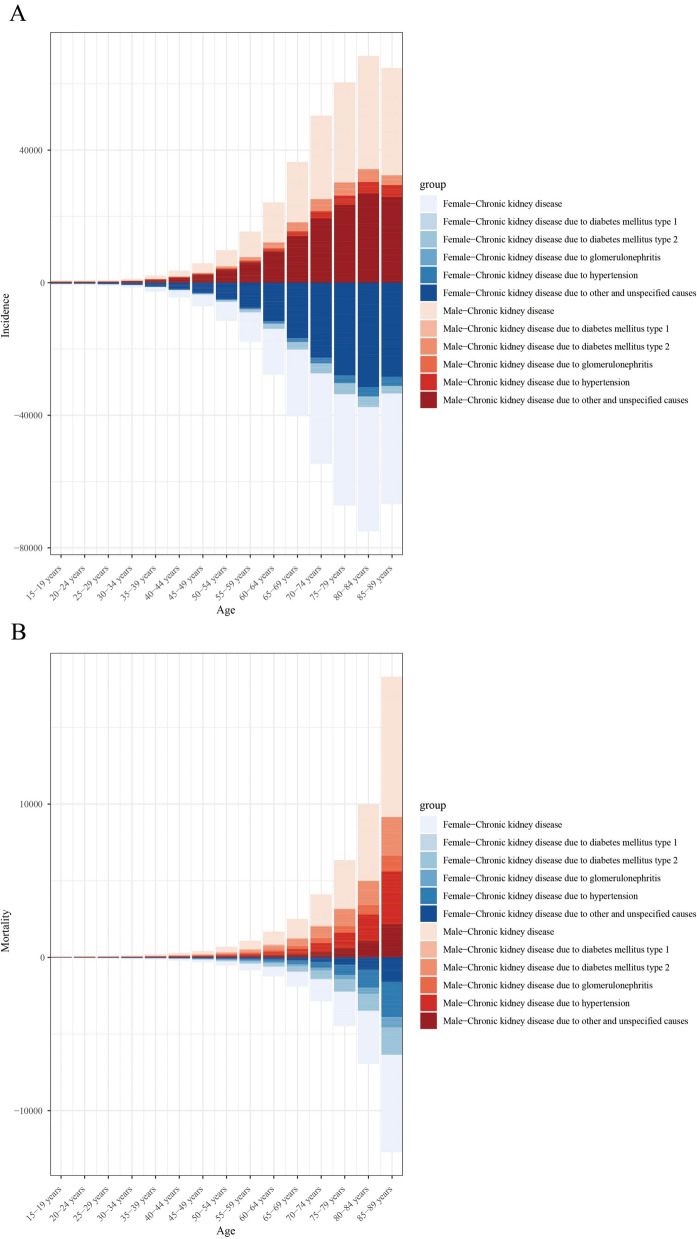
Age-specific CKD incidence rates (**A**) and CKD-related mortality (**B**) by cause and sex. CKD, chronic kidney disease

Similarly, stratified analysis by age group and gender revealed that CKD attributable to unknown causes accounted for the largest proportion across all age groups and genders, with CKD incidence increasing significantly with age (Fig. 10A). Likewise, CKD mortality rates rose substantially with age, particularly in individuals aged 70 years and older. Overall, males exhibited higher CKD mortality rates than females. The distribution of CKD mortality by etiology also showed gender and age-specific differences: in males, CKD mortality due to hypertension and unknown causes was higher, whereas in females, diabetes (particularly type 2 diabetes) and glomerulonephritis-related CKD mortality accounted for a greater proportion (Fig. 10B).

### Global health inequality analysis of incidence and mortality in CKD from 1990 to 2021

**Fig. 11:**
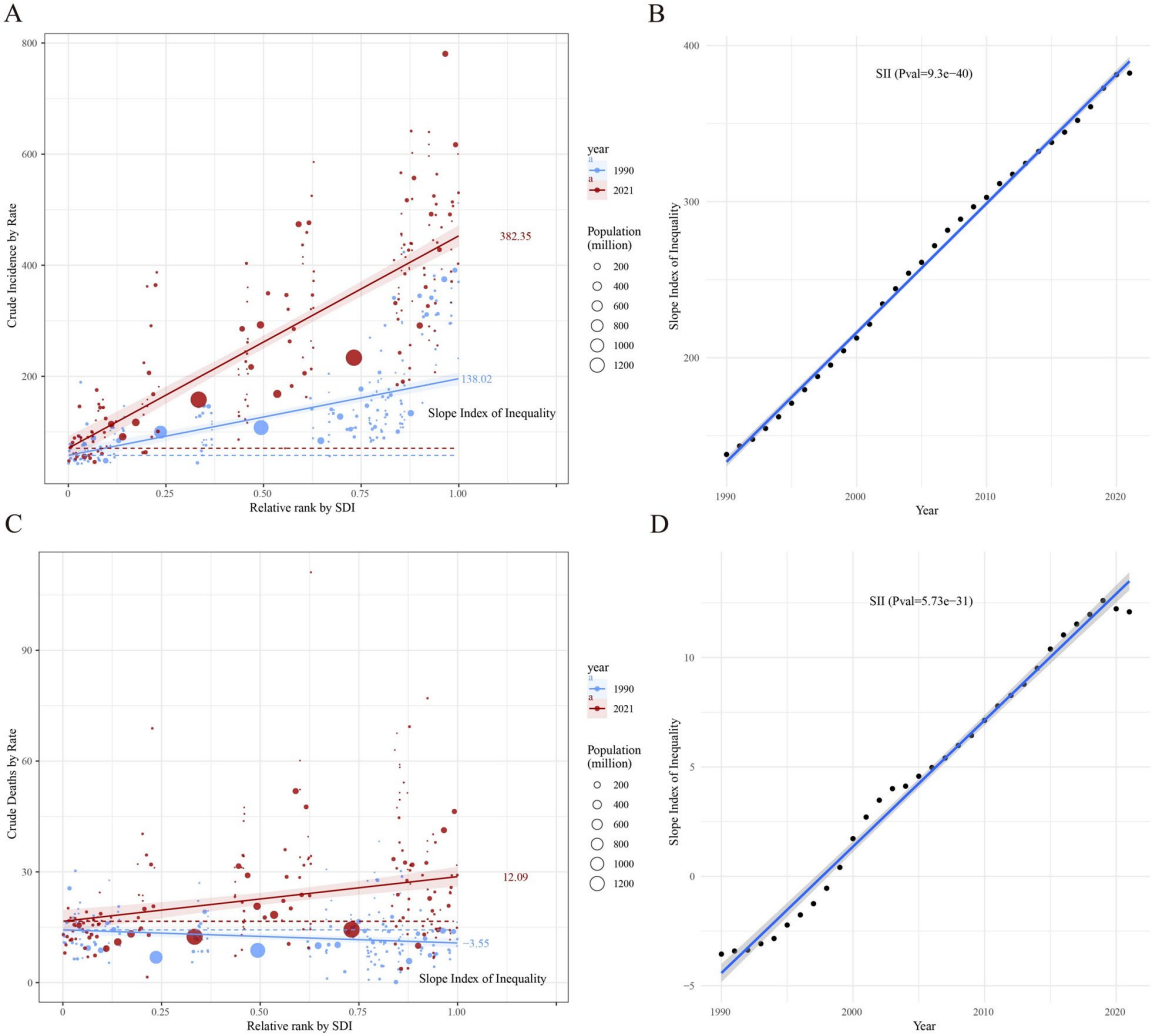
Trends in crude CKD incidence and CKD-related mortality by SDI and SII from 1990 to 2021. **A** Crude CKD incidence rate by relative SDI rank, with the slope index of inequality (SII) for 1990 and 2021; **B** change in SII over time for crude CKD incidence; **C** crude CKD-related mortality rate by relative SDI rank, with the SII for 1990 and 2021; **D** change in SII over time for crude CKD-related mortality. Circle sizes represent population size for different countries and territories

Compared with 1990, the inequality in CKD incidence and mortality among countries and regions with different SDI levels increased in 2021. CKD incidence generally showed an upward trend with increasing SDI levels, and the Slope Index of Inequality (SII) in 2021 (382.35) was significantly higher than that in 1990 (138.02) (Fig. 11A; Table S6). Moreover, the SII demonstrated a consistent annual increase, with regression results indicating extremely significant statistical relevance (P = 9.3E−40). This suggests that inequality between countries and regions with varying social development levels has steadily intensified over the past few decades, with a highly stable trend (Fig. 11B).

Similarly, the SII for CKD mortality showed an upward trend in 2021, with an SII of 12.09, in contrast to a downward trend in 1990, where the SII was − 3.55 (Fig. 11C; Table S6). The upward trend in SII regression analysis also exhibited extremely significant statistical relevance (P = 5.73E−31), indicating that inequality in CKD mortality among countries and regions with different SDI levels has steadily increased over the past few decades (Fig. 11D).

**Fig. 12:**
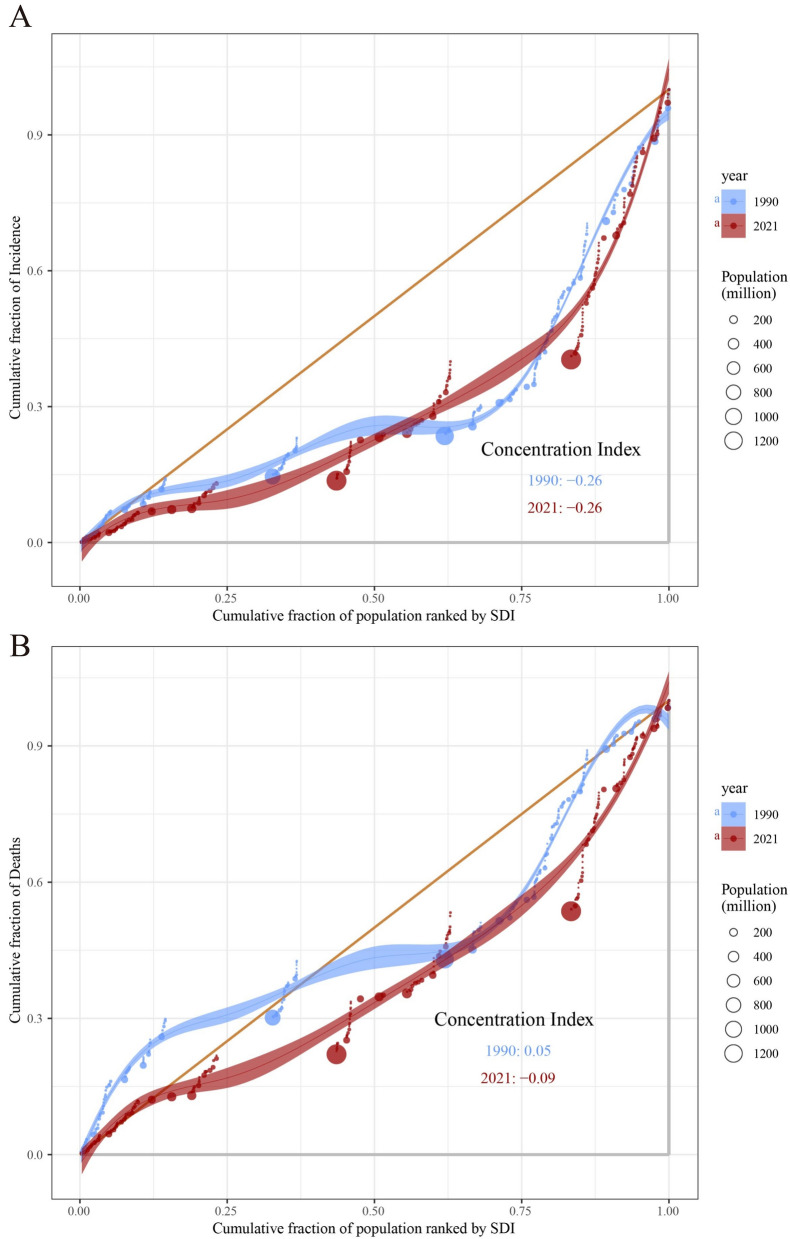
Concentration curves of CKD incidence and CKD-related mortality by SDI in 1990 and 2021. **A** Concentration curve for CKD incidence, with the concentration index for 1990 and 2021; **B** concentration curve for CKD-related mortality, with the concentration index for 1990 and 2021. The orange diagonal line represents perfect equality, where CKD incidence or mortality would be equally distributed across all SDI levels. Shaded areas represent the 95% confidence intervals of the concentration index (CI)

As shown in Fig. 12A, the cumulative curves for both 1990 and 2021 deviate from the equality line (orange diagonal), indicating that CKD incidence was more concentrated among populations with higher SDI. The concentration index (CI) for both 1990 and 2021 was − 0.26, suggesting that the degree of inequality in CKD incidence did not change significantly over time.

In contrast, the CI for CKD mortality in 1990 was 0.05, indicating a relatively equal distribution of mortality across populations with different SDI levels. However, the CI in 2021 was − 0.09, reflecting higher CKD mortality among high-SDI populations. This indicates that socioeconomic inequality in CKD mortality has been worsening over time (Fig. 12B; Table S6).

## Discussion

This study comprehensively analyzed the temporal trends in the incidence and mortality of CKD caused by five etiologies at global, regional, and national levels. Overall, from 1990 to 2021, both the incidence and mortality rates of CKD showed an upward trend. Nearly 80% of CKD cases were attributed to unknown causes, which exhibited minimal regional variation compared to specific etiologies such as diabetes and hypertension. In contrast, the distribution of CKD mortality causes was more diverse, with type 1 diabetes contributing the least, while hypertension, type 2 diabetes, and unknown causes had significant contributions, showing notable regional and temporal differences. Both CKD incidence and mortality rates increased rapidly with age. Additionally, CKD incidence was higher in females, while CKD mortality was higher in males, consistent with previous findings. In 1990 and 2021, CKD incidence was primarily concentrated in regions with medium and high SDI levels (CI − 0.26). Compared to 1990, CKD mortality in 2021 became increasingly concentrated in high-SDI regions (CI − 0.09), highlighting a growing and stable trend of health inequality among countries and regions with varying levels of social development. The results of this study expand and complement the understanding of the global burden of CKD.

Consistent with previous studies, diabetes (primarily type 2 diabetes) and hypertension were the major contributors to CKD with known etiologies [17, 25, 26], with their proportions increasing in 2021. Diabetes-related CKD (CKD-DM) was more prevalent in medium- and high-SDI regions, such as East Asia (primarily China), OECD countries, and the European Union. The rising CKD-DM burden can largely be attributed to the increasing prevalence of diabetes, such as the growing incidence of diabetes in North America and the Caribbean [27]. The proportion of hypertension-related CKD incidence was similar to that of CKD-DM. Reports indicate that the highest age-standardized incidence rate (ASIR) of hypertension-related CKD was observed in high-SDI regions, while the highest age-standardized mortality rate (ASMR) occurred in low-SDI regions, with both global ASIR and ASMR showing upward trends [26]. Glomerulonephritis-related CKD was more common in Asian and sub-Saharan African countries [17], which was also observed in this study. Notably, the global distribution of CKD with unknown causes remained relatively stable between 1990 and 2021, with approximately 80% of CKD cases lacking a clear etiology. According to the ARIMA model, the number of CKD cases is projected to rise from 19,935,037.8 in 2021 to 22,210,613.5 in 2031, with CKD-related deaths also expected to increase in the coming years. Meanwhile, the inequality in CKD burden among countries and regions with varying levels of social development continues to intensify. These findings underscore the urgent need for further CKD research to address the growing burden of this disease.

Age-period-cohort analysis revealed that CKD incidence and mortality rates increased with age, peaking between 70 and 80 years. Previous epidemiological evidence suggests that age is an independent and critical risk factor for CKD, with age-related variations in mortality due to impaired kidney function [28, 29]. Additionally, older populations are more prone to cardiovascular diseases, diabetes, and hypertension, which significantly elevate the risk of CKD [30, 31]. The period effect reflects the influence of healthcare conditions, diagnostic technologies, and economic and cultural changes during specific timeframes on the CKD burden. Cohort effects highlight the impact of socioeconomic, behavioral, and environmental exposures during early life on the risk among different birth cohorts. Our findings indicate a decline in the period effect for both CKD incidence and mortality. Moreover, earlier birth cohorts exhibited higher risks of CKD incidence and mortality compared to recent birth cohorts. This may be attributed to advancements in medical technology, improvements in treatment approaches, the implementation of public health policies, and heightened health awareness among populations. Notably, from 1990 to 2021, changes in CKD incidence and mortality were primarily driven by epidemiological shifts, particularly in regions other than low-SDI areas, with minimal influence from age and population demographics. These findings were consistent across genders. We speculate that the growing prevalence of unhealthy lifestyles (e.g., high-salt diets, sleep deprivation, and sedentary behaviors) [32–34], and rising obesity rates [35] have contributed to the increased burden of CKD by promoting hypertension, diabetes, and related conditions. Additionally, we observed that the estimated annual percentage change (EAPC) stabilized when the baseline ASIR exceeded approximately 100. A possible explanation is that higher baseline ASIRs make CKD control increasingly challenging. In summary, we recommend strengthening early CKD screening and intervention among high-risk populations (e.g., older adults and patients with hypertension or diabetes) and promoting healthy lifestyles to minimize the growing CKD burden effectively.

The health inequality analysis reveals an increasing disparity in the burden of CKD between countries and regions with different levels of socio-economic development. Notably, the CKD burden in high SDI regions is becoming progressively heavier. Currently, global aging is accelerating, with varying rates of aging across different regions [36], which may lead to an imbalanced CKD burden. For example, populations in high SDI regions typically have longer life expectancies and experience faster aging processes, resulting in a heavier CKD burden [37]. In contrast, low SDI regions have a slower aging rate, and due to inadequate healthcare resources and limited healthcare services, the diagnosis and management of CKD are suboptimal, leading to a lower CKD burden. However, inconsistencies in data collection and diagnostic biases between countries and regions may affect our analysis results. For instance, in low-income countries, CKD etiology confirmation and reporting may be uncertain due to scarce medical resources, incomplete health information, or inadequate diagnostic technologies, potentially leading to underreporting of CKD incidence and mortality. These potential reporting quality differences could influence the findings of this study, especially in the health inequality analysis. Future research should focus more on standardizing diagnostic criteria globally and improving health data collection methods to reduce reporting discrepancies of latent diseases, thereby enhancing the accuracy of health inequality analyses. At the same time, low SDI regions should strengthen primary healthcare infrastructure, improve health education, and implement preventive measures to increase awareness of early CKD symptoms and risk factors (such as diabetes and hypertension), reducing CKD risks and progression. High SDI regions should develop targeted policies for elderly health, optimize health insurance, and promote precision medicine and early CKD detection to address the growing CKD burden.

This study has several limitations. First, the accuracy and robustness of GBD estimates heavily rely on the quality and quantity of the data used in the modeling. For instance, underreporting of CKD or the lack of healthcare information in certain impoverished areas can lead to underestimation of CKD due to specific causes, incomplete health records, or poor management, resulting in CKD not being accurately documented or classified. The lack of clear classification of causes contributes to a higher proportion of unidentified CKD cases. Additionally, age-period-cohort analysis reveals population-level outcomes, which may be influenced by ecological fallacy. Lastly, differences in data collection methods, technologies, and tools across countries and regions may result in mixed evidence that cannot easily distinguish between quality and flaws. For instance, health inequality analyses are affected by variations in diagnostic standards, reporting quality, and data collection methods across different countries and regions.

## Conclusion

This study estimated the temporal trends of CKD incidence and mortality globally, as well as at the national and regional levels, from 1990 to 2021. It was observed that countries with higher socio-demographic index (SDI) exhibited unfavorable trends, suggesting that these countries should develop more targeted and specific strategies to address the growing burden of CKD.

## Supplementary Information

Supplementary Material 1.

## Data Availability

No datasets were generated or analysed during the current study.
