# Supplementary material for: Global, regional, and national trends in chronic kidney disease burden (1990–2021): a systematic analysis of the global burden of disease in 2021
Source: Trop Med Health. 2025 Feb 21;53:26. doi: 10.1186/s41182-025-00703-x (PMC11843816; doi:10.1186/s41182-025-00703-x)
Supplement: Supplementary file 1 — Supplementary Material 1. [file 41182_2025_703_MOESM1_ESM.doc]

Table S1. The CKD cases and age standardized incidence of CKD caused by different reasons from 1990 to 2021.

| Cause | Location | 1990 | | 2021 | | 1990-2021 |
| --- | --- | --- | --- | --- | --- | --- |
|  |  | Incident cases  No. ×10^3^ (95% UI) | ASIR per 100,000  No. (95% UI) | Incident cases  No. ×10^3^ (95% UI) | ASIR per 100,000  No. (95% UI) | EAPC  No. (95% CI) |
| CKD | Afghanistan | 18832.6 (16974.6-21041.3) | 255.1 (231-282.2) | 45513.8 (42293.6-49787.4) | 367.2 (338.5-402.2) | 0.91 (-1.84-3.73) |
| CKD | Albania | 2815.1 (2542.1-3125.6) | 120.7 (108.1-134.5) | 8621.4 (7733.5-9517.9) | 206.7 (188.5-225) | 1.71 (-0.68-4.16) |
| CKD | Algeria | 35770.5 (32577.6-39278.7) | 256.6 (233.4-281.9) | 153074.6 (140677.1-165614.4) | 407.5 (375.5-438.8) | 1.19 (-1.58-4.04) |
| CKD | American Samoa | 61.2 (56.6-66.4) | 212.8 (195.8-231) | 156.2 (143.3-171) | 312.6 (289.1-337.2) | 1.01 (-1.62-3.71) |
| CKD | Andorra | 126.3 (113.1-141.9) | 224.5 (203.4-250.8) | 336.4 (307-369.6) | 222.6 (202.7-245.4) | 0.1 (-2.63-2.9) |
| CKD | Angola | 4838.6 (4507.1-5162.2) | 89.5 (82.6-97.1) | 19883.4 (18458-21539.9) | 129.1 (118.2-140.5) | 0.82 (-1.46-3.14) |
| CKD | Antigua and Barbuda | 114.1 (105.1-124.2) | 219.1 (201-238.1) | 375.3 (343.2-408.5) | 344.7 (318.7-371.8) | 1.35 (-1.26-4.03) |
| CKD | Argentina | 68542.6 (62475.6-75476.1) | 213.6 (195.3-234.4) | 150963 (137930.3-163854.2) | 269.6 (246.9-290.7) | 0.76 (-1.89-3.48) |
| CKD | Armenia | 2818.9 (2505.7-3157) | 94.3 (84.4-105.1) | 7532.5 (6762.1-8397.2) | 184.3 (168.3-201.7) | 1.98 (-0.36-4.38) |
| CKD | Australia | 51807.4 (49083.9-54961.6) | 258.1 (245-272.1) | 135305.5 (123723.1-145659) | 296.3 (272-317.2) | 0.53 (-2.2-3.35) |
| CKD | Austria | 27499 (24723.9-30726.3) | 224.6 (204-247.3) | 50646.8 (46366.5-55367.9) | 275.9 (249.4-303) | 0.75 (-1.99-3.58) |
| CKD | Azerbaijan | 6575.5 (5912.6-7257.1) | 110.2 (99.7-121) | 22386.6 (19939.2-24790.5) | 203.8 (187.1-221.1) | 1.98 (-0.29-4.31) |
| CKD | Bahamas | 345.6 (320.6-373.7) | 197.3 (182.3-216.2) | 1251.5 (1140.6-1352.8) | 295.9 (274-317.2) | 1.27 (-1.29-3.9) |
| CKD | Bahrain | 611.8 (557.4-676.4) | 292.8 (268-320.8) | 4873.2 (4360.8-5457.3) | 451.2 (413.4-487.2) | 1.18 (-1.68-4.13) |
| CKD | Bangladesh | 52608.1 (48338.5-57513) | 90 (81.9-99.4) | 187707.2 (170316.3-207797.5) | 131.8 (120-144.8) | 0.85 (-1.45-3.2) |
| CKD | Barbados | 522.9 (480.2-575.9) | 194.4 (178.6-213.8) | 1473.4 (1347.8-1605.3) | 308 (286.4-332.9) | 1.31 (-1.25-3.95) |
| CKD | Belarus | 10524 (9524.7-11766.8) | 87.2 (79.8-96.2) | 20010 (18172.5-22130.6) | 141.3 (128.9-154.2) | 1.3 (-0.93-3.59) |
| CKD | Belgium | 38016.3 (34545.2-42363.3) | 240.5 (220.3-264.1) | 58480.4 (53424.5-63159.2) | 247.4 (226.5-265.9) | 0.2 (-2.55-3.04) |
| CKD | Belize | 213.2 (196.6-231.2) | 193.5 (176.1-212.4) | 1051.8 (971.6-1129.4) | 312 (288.7-335.7) | 1.35 (-1.18-3.94) |
| CKD | Benin | 3700.9 (3453.9-3983.8) | 135.8 (124.8-147.4) | 12189.5 (11329.2-13015.7) | 177.8 (162.4-192.6) | 0.61 (-1.76-3.04) |
| CKD | Bermuda | 121.2 (109.9-133.4) | 192.7 (175.3-211.4) | 392.3 (356.2-427.9) | 321.1 (295.6-348.3) | 1.57 (-1.07-4.29) |
| CKD | Bhutan | 425 (391.3-459.2) | 125.4 (115.5-137.7) | 1197.1 (1094.4-1304.9) | 185.8 (169.7-202.7) | 1.12 (-1.29-3.58) |
| CKD | Bolivia (Plurinational State of) | 6471.2 (5993.8-6956.3) | 176 (162-190.1) | 24667.1 (22843.4-26922.2) | 262.4 (242.5-285.9) | 1.13 (-1.44-3.77) |
| CKD | Bosnia and Herzegovina | 5153 (4622.9-5700.5) | 121 (109.7-132.5) | 13515 (12362.7-14758.9) | 227.9 (209.4-246.4) | 1.82 (-0.72-4.42) |
| CKD | Botswana | 1010.1 (928.3-1097.1) | 151.6 (138.7-165.3) | 3507.4 (3221.9-3805.1) | 217.2 (199.9-237) | 0.84 (-1.65-3.4) |
| CKD | Brazil | 189482.9 (174872.9-205093.5) | 192.9 (176.9-209.6) | 644744.3 (602332.7-688116.8) | 258.1 (241.7-274.5) | 0.73 (-1.81-3.35) |
| CKD | Brunei Darussalam | 322.3 (296-352) | 295 (269.3-322.7) | 1125.5 (1021.5-1250.4) | 328.3 (302.5-357.6) | 0.43 (-2.3-3.24) |
| CKD | Bulgaria | 15333.9 (13680.7-17203.9) | 129 (117.3-142.5) | 29662.5 (26863.5-32623.3) | 232.2 (215-251) | 1.73 (-0.7-4.22) |
| CKD | Burkina Faso | 7167.5 (6643.4-7770.7) | 126.5 (116.6-138.3) | 20260.8 (19125.7-21649.5) | 165.8 (153.5-179.6) | 0.78 (-1.56-3.17) |
| CKD | Burundi | 2855.9 (2657.6-3083) | 93.9 (86.4-102.2) | 6739.6 (6243.8-7274.5) | 109.7 (101-119.6) | 0.33 (-1.91-2.61) |
| CKD | Cabo Verde | 303.9 (280.4-331.1) | 114.8 (105.2-125.2) | 816.2 (742.3-885.2) | 179.5 (162.8-195.6) | 1.1 (-1.26-3.52) |
| CKD | Cambodia | 7526.5 (6954.4-8199.6) | 126.4 (115.8-139.6) | 24541.6 (22491-27010.7) | 183.3 (168.5-200.9) | 0.89 (-1.49-3.33) |
| CKD | Cameroon | 10675.6 (9918.8-11508.8) | 178.4 (164-194.7) | 39453.3 (36598.9-41900.6) | 236.2 (219.2-253.8) | 0.66 (-1.77-3.16) |
| CKD | Canada | 85191.5 (77747.6-90551.3) | 260.7 (239-276.3) | 184153 (167874.7-204154.4) | 256.4 (234.4-282.3) | 0.34 (-2.23-2.97) |
| CKD | Central African Republic | 1379 (1268-1499.4) | 91.2 (83.6-99.2) | 3386.8 (3140.1-3657.4) | 118.2 (109.2-128.5) | 0.58 (-1.68-2.9) |
| CKD | Chad | 4704.5 (4390.9-5083.2) | 127.6 (117-139.5) | 13104.5 (12245.4-14002.8) | 154.9 (142.4-168.8) | 0.48 (-1.86-2.87) |
| CKD | Chile | 21715.5 (19659.9-24017.2) | 216.6 (196.6-239.2) | 80300.2 (73876.9-86347.4) | 316.6 (292.4-340) | 1.19 (-1.54-4.01) |
| CKD | China | 1267533 (1145753.1-1385804.6) | 147.3 (133.9-161.5) | 3323175.2 (3068978.4-3559323.9) | 163.7 (153-174.1) | 0.25 (-2.21-2.76) |
| CKD | Colombia | 45649.4 (41527.7-50029.3) | 227.2 (206.6-251) | 171449.5 (156961.1-186838.9) | 314.6 (289.4-341.1) | 0.97 (-1.67-3.69) |
| CKD | Comoros | 268.4 (248.8-289.6) | 103.4 (95.2-112.6) | 672 (626.8-725.4) | 127.5 (118.5-138.8) | 0.38 (-1.89-2.7) |
| CKD | Congo | 1393.5 (1291.4-1511.9) | 102.2 (94.4-111.2) | 4644.9 (4325.3-5062.4) | 147.1 (136.7-160) | 0.82 (-1.52-3.21) |
| CKD | Cook Islands | 23.9 (21.7-26.1) | 174.2 (158.5-190.1) | 69.7 (63-76.1) | 284.5 (260.5-308.2) | 1.27 (-1.36-3.97) |
| CKD | Costa Rica | 8200.4 (7966.3-8432.3) | 436.3 (425.1-446.4) | 24923.4 (24421.5-25363.4) | 454.5 (445.2-462.6) | 0.16 (-2.67-3.07) |
| CKD | C么te d'Ivoire | 9012.2 (8351.8-9722) | 150.9 (139-164.3) | 28313.8 (26274.9-30295.9) | 193.2 (178-209.9) | 0.54 (-1.85-3) |
| CKD | Croatia | 9145.4 (8175-10238.4) | 150.6 (136.3-166.1) | 21855.6 (19854.9-23659.4) | 259.7 (238.7-279.3) | 1.64 (-0.99-4.34) |
| CKD | Cuba | 15078.4 (13825.3-16515) | 146.8 (133.9-160.7) | 49192.7 (44768.6-53426.9) | 271.1 (248.7-295.4) | 1.7 (-0.86-4.31) |
| CKD | Cyprus | 2321 (2070-2607.2) | 275.5 (254.5-300.6) | 6413 (5860.3-7052.5) | 296 (272.1-324) | 0.33 (-2.54-3.29) |
| CKD | Czechia | 17191.4 (15473.8-19227.2) | 130 (118.1-143.6) | 44761.9 (40513.3-49823.8) | 218 (200.6-238.5) | 1.35 (-1.2-3.96) |
| CKD | Democratic People's Republic of Korea | 23089.8 (20851.1-25212.4) | 138.4 (125.1-151) | 58635.6 (52974.6-64305.8) | 185.3 (168.5-201.7) | 0.71 (-1.75-3.23) |
| CKD | Democratic Republic of the Congo | 19561.9 (18061.8-21127.3) | 94.6 (86.8-103.2) | 57306.2 (53005.1-62100.7) | 128.3 (118-140.8) | 0.64 (-1.66-3) |
| CKD | Denmark | 17126.2 (15297.6-19106.9) | 207.1 (187.3-228.3) | 29642.5 (27033.8-32472.4) | 243.4 (224-263.1) | 0.64 (-2.02-3.38) |
| CKD | Djibouti | 185.2 (171.4-199.7) | 98.7 (90.6-107.5) | 935.7 (861.6-1010.7) | 128.6 (118-139) | 0.59 (-1.66-2.9) |
| CKD | Dominica | 139.3 (129.1-150.8) | 229.6 (212.4-247.7) | 261.9 (238.5-285.3) | 320.8 (295.7-345.2) | 1.08 (-1.46-3.68) |
| CKD | Dominican Republic | 6201.9 (5697-6820.6) | 139.2 (126.4-153.7) | 24963.3 (23057.3-26894.8) | 243 (224-261.7) | 1.55 (-0.94-4.1) |
| CKD | Ecuador | 10748 (9822-11798.9) | 180.7 (163.6-199.8) | 57933.7 (53462.4-63236) | 348.2 (321.3-380) | 1.93 (-0.77-4.71) |
| CKD | Egypt | 80782.5 (73484.1-89103.4) | 263.8 (240.7-289.5) | 301549.5 (272714.7-333683.6) | 428.8 (397.3-465.6) | 1.25 (-1.52-4.09) |
| CKD | El Salvador | 7712.2 (7062.8-8456.7) | 229.5 (209.1-253.9) | 24978.4 (23301.5-26600) | 408.4 (380-435.7) | 2.06 (-0.6-4.79) |
| CKD | Equatorial Guinea | 226.7 (210.2-246) | 92.5 (85.3-101.5) | 1065.4 (986.8-1146.4) | 165.2 (151.3-180.5) | 1.63 (-0.74-4.07) |
| CKD | Eritrea | 1469.5 (1358.1-1586.4) | 91.2 (84-99.7) | 3984.3 (3698.1-4299.4) | 116.9 (106.8-127.4) | 0.48 (-1.76-2.78) |
| CKD | Estonia | 1993.1 (1779.4-2237.6) | 104.5 (94.3-116.3) | 4909.1 (4492.8-5418.3) | 215.9 (198-235.7) | 2.1 (-0.27-4.53) |
| CKD | Eswatini | 688.8 (635.2-749.7) | 184.5 (169.2-202.9) | 1567.6 (1441.3-1700.1) | 232.9 (212.3-252.2) | 0.54 (-1.92-3.07) |
| CKD | Ethiopia | 25075.2 (23288-27039.9) | 92.9 (86-100.7) | 60891.8 (56784.5-64771.4) | 114.6 (105-124.2) | 0.38 (-1.86-2.66) |
| CKD | Fiji | 881.1 (810.9-958.9) | 195.3 (179.7-213) | 2163.3 (1978.3-2345.4) | 261.9 (243.7-283.2) | 0.71 (-1.79-3.28) |
| CKD | Finland | 12464.4 (11114.2-14014.6) | 171.1 (154-189.6) | 25918 (23653.7-28780.8) | 195.4 (180.1-214.1) | 0.47 (-2.17-3.18) |
| CKD | France | 179844.6 (162635.2-201948.9) | 207.5 (188.8-229.6) | 326610.7 (300075.4-352984.5) | 229.8 (212.4-249.8) | 0.33 (-2.4-3.14) |
| CKD | Gabon | 755.4 (697.7-817.9) | 115.8 (106.8-125.6) | 2115.4 (1943.9-2292.6) | 182.3 (168.3-196.9) | 1.05 (-1.35-3.51) |
| CKD | Gambia | 662.1 (623-706) | 133.9 (123.6-144.7) | 2198.3 (2064.3-2346.3) | 176.6 (162.4-191.8) | 0.63 (-1.76-3.08) |
| CKD | Georgia | 6362.2 (5719.7-7116.1) | 105 (95.2-116.6) | 9241.3 (8319.7-10162.7) | 177.7 (164.1-193) | 1.56 (-0.76-3.94) |
| CKD | Germany | 312632.9 (283719.1-347849.4) | 242.8 (222.3-269.7) | 526669.6 (491104-565042) | 269.8 (254.5-287.4) | 0.35 (-2.39-3.18) |
| CKD | Ghana | 9860.2 (9229.9-10595.4) | 120.1 (111.5-130.6) | 34499 (31871.9-37393.6) | 171.1 (157.3-186.3) | 0.92 (-1.48-3.37) |
| CKD | Greece | 44005.5 (40000.5-48285.2) | 283.9 (258.7-310.5) | 65277.6 (60702-70955.4) | 274.9 (254.2-297.8) | 0.07 (-2.76-2.99) |
| CKD | Greenland | 60.1 (54-65.4) | 201 (181.8-220.5) | 148.7 (136.4-164.6) | 231 (213.6-252.8) | 0.43 (-2.15-3.06) |
| CKD | Grenada | 149 (138.5-160.4) | 210.5 (194.1-227.4) | 413.3 (380.1-447.2) | 355 (328.5-380) | 1.46 (-1.13-4.12) |
| CKD | Guam | 166.4 (151.5-182.9) | 180.8 (165.5-199.1) | 533.7 (487.6-587) | 265.3 (244.9-289.5) | 1.15 (-1.39-3.75) |
| CKD | Guatemala | 10598.8 (9673.6-11591.4) | 244 (221.5-267) | 45885.4 (42467.4-49772.1) | 387.9 (358-420.4) | 1.42 (-1.25-4.15) |
| CKD | Guinea | 5458.9 (5059.8-5900.9) | 134.1 (123.1-146.2) | 12458.2 (11578.8-13321.8) | 171.1 (157.5-184.4) | 0.57 (-1.78-2.99) |
| CKD | Guinea-Bissau | 795.5 (740.6-850.7) | 142.9 (133.4-154.4) | 1762 (1638-1884.3) | 170.4 (157.2-184.1) | 0.37 (-1.99-2.77) |
| CKD | Guyana | 835.5 (775.8-898.6) | 177.1 (163.4-191.6) | 2045.9 (1880.1-2218.6) | 294.6 (273.7-317.6) | 1.35 (-1.21-3.97) |
| CKD | Haiti | 6772 (6073.4-7507.5) | 170.4 (152.6-191.5) | 19289.6 (17777.4-20946.2) | 226 (207.1-245.9) | 0.75 (-1.76-3.32) |
| CKD | Honduras | 5947.9 (5451.7-6582.3) | 236.2 (214.1-264.7) | 22686.3 (20944.5-24584.8) | 320.5 (295.8-348.5) | 0.82 (-1.85-3.57) |
| CKD | Hungary | 15676.4 (14056.3-17503.4) | 111.7 (101.5-123.8) | 42184.5 (38411.7-46628.4) | 227.6 (209.3-248.8) | 1.92 (-0.63-4.53) |
| CKD | Iceland | 560 (508.7-615.8) | 192.2 (175.1-210.8) | 1166.1 (1065.7-1264.3) | 199.8 (182.8-216.9) | 0.14 (-2.54-2.89) |
| CKD | India | 849302.1 (780461.6-920171.4) | 152.8 (140.4-165.7) | 2235891.8 (2056365.8-2414555) | 180.7 (166.5-195.4) | 0.2 (-2.29-2.75) |
| CKD | Indonesia | 155643.9 (144274.9-167962.8) | 130.3 (120.1-141.7) | 469569.5 (432377.1-511150.1) | 185.1 (171.2-200.1) | 0.85 (-1.44-3.19) |
| CKD | Iran (Islamic Republic of) | 93053.3 (83778.5-103124.9) | 304.2 (277.3-334.7) | 330886.9 (308639-354798.2) | 409 (382.6-436) | 0.68 (-2.02-3.44) |
| CKD | Iraq | 27066.4 (24992.9-29348.1) | 291.5 (267.3-319.7) | 117614.5 (108689.2-127824.8) | 440.3 (407.2-473.9) | 1.1 (-1.69-3.96) |
| CKD | Ireland | 11405.1 (10693.3-11948.5) | 274 (257.2-285.8) | 19200.7 (18875.6-19506.1) | 245.5 (242.3-248.8) | 0.06 (-2.55-2.74) |
| CKD | Israel | 13679.9 (12392.7-15201.6) | 273.6 (249.3-301.5) | 37867.4 (34115.5-41514.7) | 305.8 (275.3-333.6) | 0.45 (-2.39-3.37) |
| CKD | Italy | 195977.3 (175595.2-217213.3) | 217.4 (196.2-239.3) | 333141.6 (306700.2-361005) | 218.5 (202.7-235.1) | 0.19 (-2.44-2.9) |
| CKD | Jamaica | 3710.1 (3408.1-4045.5) | 203.9 (186.2-222.8) | 8988.7 (8236.1-9704.3) | 295.4 (271.5-318.4) | 1.11 (-1.47-3.77) |
| CKD | Japan | 471524.7 (434119.8-511166.9) | 280 (259.2-302.1) | 996707.6 (911293-1081250) | 285.2 (263.7-305.7) | 0.09 (-2.7-2.96) |
| CKD | Jordan | 4664.3 (4322-5035.3) | 289.6 (266.7-312.6) | 37316.1 (34745.5-40072.6) | 448.8 (420.9-478.2) | 1.23 (-1.59-4.13) |
| CKD | Kazakhstan | 14602.2 (13439-15890.5) | 101.8 (93.5-111.2) | 35051.2 (31601-38748.8) | 183.9 (168.9-201.6) | 1.58 (-0.73-3.95) |
| CKD | Kenya | 10255.3 (9567.2-10957.3) | 92.9 (85.5-101.1) | 31651 (29147.6-34091.7) | 118.9 (109.2-129.5) | 0.41 (-1.83-2.7) |
| CKD | Kiribati | 77.1 (70.7-83.7) | 155 (142.8-169) | 191.6 (177.3-207.4) | 215.9 (199.8-232.1) | 0.83 (-1.61-3.34) |
| CKD | Kuwait | 2251.8 (2070.3-2441) | 293 (271.2-317) | 13108.6 (11785.1-14632.1) | 397 (363.7-431.8) | 0.88 (-1.9-3.73) |
| CKD | Kyrgyzstan | 3990.8 (3710.1-4283.1) | 105.5 (97.4-114) | 8822.5 (8020.4-9621.9) | 151 (138-164.5) | 0.92 (-1.3-3.19) |
| CKD | Lao People's Democratic Republic | 4381.1 (4021.9-4784.7) | 169.7 (155.2-185.9) | 12020.5 (11060-12971.3) | 227.7 (209.2-246.2) | 0.77 (-1.67-3.28) |
| CKD | Latvia | 2877 (2583-3214) | 87.6 (79.5-96.7) | 5423 (4898.1-6021.6) | 165.9 (152.5-181.7) | 1.61 (-0.69-3.96) |
| CKD | Lebanon | 6124 (5556.4-6781) | 267.4 (245.4-292.7) | 25284.8 (23327-27213.3) | 435.3 (398-470.8) | 1.3 (-1.5-4.19) |
| CKD | Lesotho | 1418.7 (1304.6-1543.4) | 149.8 (137.2-163.7) | 2454.7 (2265.3-2645.9) | 201.8 (186.1-216.7) | 0.68 (-1.75-3.18) |
| CKD | Liberia | 1958.8 (1816.3-2115.1) | 130.3 (119.7-142.2) | 4918.6 (4557.8-5316.6) | 177.9 (161.7-194.5) | 0.88 (-1.51-3.34) |
| CKD | Libya | 5633.1 (5188.5-6163.4) | 262.4 (239.5-288.3) | 23278 (21518.8-25396.4) | 417.7 (384.9-453.6) | 1.06 (-1.74-3.94) |
| CKD | Lithuania | 4204.9 (3814.4-4675.7) | 99 (90.4-109.6) | 6974.5 (6366-7567.8) | 147.6 (137-159.2) | 1.08 (-1.19-3.41) |
| CKD | Luxembourg | 1367.1 (1246.1-1511) | 243.8 (223.3-267.3) | 2759.4 (2529.4-2960.8) | 263.1 (240.9-283) | 0.38 (-2.41-3.26) |
| CKD | Madagascar | 5781.5 (5385-6207.7) | 86.1 (79.3-93.7) | 15047.4 (13993.2-16360.9) | 107 (97.7-116.4) | 0.41 (-1.79-2.66) |
| CKD | Malawi | 4890.2 (4550-5238.2) | 96.8 (89.1-105.9) | 11238.9 (10342.6-11976.9) | 124 (112.8-133.9) | 0.64 (-1.61-2.94) |
| CKD | Malaysia | 18546.1 (17026-20013.1) | 175.5 (159.3-190.3) | 77138.4 (70879.4-83439) | 260 (240.1-281.2) | 1.04 (-1.57-3.71) |
| CKD | Maldives | 214.2 (193.4-236.8) | 186.9 (169.2-206.1) | 1058.7 (951.8-1154.6) | 288.3 (259.9-317.9) | 1.18 (-1.41-3.83) |
| CKD | Mali | 6631 (6181.2-7133.8) | 125.1 (115.6-135.8) | 19506.3 (18188.6-20763.9) | 159.7 (147.2-172.1) | 0.6 (-1.76-3) |
| CKD | Malta | 1094.6 (1000.3-1201.7) | 256.2 (235.6-279.7) | 2662.9 (2436-2925.8) | 266.7 (246.7-289.8) | 0.26 (-2.47-3.06) |
| CKD | Marshall Islands | 38.9 (36.1-41.7) | 167.2 (154.2-182.6) | 102 (93-110.1) | 245.2 (225.9-264) | 0.9 (-1.63-3.49) |
| CKD | Mauritania | 1887.9 (1754.4-2045.9) | 148.6 (137-161.3) | 5232.2 (4832.9-5666.8) | 200.9 (183.2-219.1) | 0.79 (-1.62-3.26) |
| CKD | Mauritius | 2071.4 (1884-2281) | 254.9 (231.4-279.3) | 7450.9 (6924.5-8054.8) | 406.8 (383.3-433.7) | 1.48 (-1.15-4.19) |
| CKD | Mexico | 151265.3 (138995.6-165600.1) | 304.8 (278-335.4) | 612576.6 (580429.3-645104.5) | 464.3 (440.4-488.1) | 1.37 (-1.37-4.18) |
| CKD | Micronesia (Federated States of) | 115.1 (105.9-125.9) | 186.5 (171.9-204.5) | 246.6 (223.1-269.1) | 290.8 (268.2-313.6) | 1.15 (-1.4-3.77) |
| CKD | Monaco | 155.8 (140-174.8) | 211.4 (191.5-233.3) | 227.2 (205.6-250.2) | 228.4 (209.3-249.9) | 0.32 (-2.39-3.1) |
| CKD | Mongolia | 2040.1 (1864.1-2228) | 139.6 (126-153.2) | 5269.2 (4829.8-5728.2) | 188.3 (171.5-203.8) | 0.7 (-1.62-3.08) |
| CKD | Montenegro | 1134.2 (1028.6-1250.5) | 181.2 (164.7-199.2) | 2638.5 (2368.2-2915.5) | 274.2 (249.2-301.4) | 1.24 (-1.28-3.83) |
| CKD | Morocco | 32616.9 (29937.9-35664.4) | 210.1 (191.8-230.8) | 135407.2 (123555.2-147156.6) | 384.3 (352.3-415.8) | 1.62 (-1.1-4.43) |
| CKD | Mozambique | 6980 (6451.2-7572.6) | 93.1 (85.2-102) | 17355.1 (16180-18601.5) | 118.7 (109.3-129.1) | 0.55 (-1.67-2.82) |
| CKD | Myanmar | 43844.9 (39884.5-48151.2) | 159.9 (145.8-176.2) | 116367.6 (106211.9-125983.1) | 227.6 (209.3-245) | 0.97 (-1.45-3.45) |
| CKD | Namibia | 1155.8 (1061.4-1260) | 153.6 (140.7-167.2) | 2984.6 (2740.1-3260.2) | 198 (181.9-217.1) | 0.59 (-1.89-3.12) |
| CKD | Nauru | 10.9 (10-11.9) | 179.9 (165.3-196) | 18.5 (16.9-20.1) | 256.1 (234.8-279.1) | 0.85 (-1.66-3.42) |
| CKD | Nepal | 14841.6 (13643.3-16160.2) | 124.3 (114-136.8) | 54612.5 (49880-59454.3) | 213.7 (195.5-232.4) | 1.2 (-1.28-3.74) |
| CKD | Netherlands | 44063.9 (40880.9-47584) | 213.5 (198.9-229.7) | 88460 (81319.9-95823.7) | 242.8 (224.7-261.3) | 0.62 (-2.11-3.43) |
| CKD | New Zealand | 10613.2 (9606.1-11746.7) | 264.7 (242.8-290.5) | 25355.9 (23124.3-27531.3) | 301.7 (277.3-327.3) | 0.55 (-2.16-3.34) |
| CKD | Nicaragua | 5617.5 (5152.8-6112) | 277.7 (252.1-306.7) | 24135.5 (22515.6-25614) | 438.2 (408.7-465.3) | 1.31 (-1.31-4) |
| CKD | Niger | 5267.4 (4895.6-5655.2) | 125 (115.7-136.1) | 17881.3 (16629.4-19192.5) | 152.1 (138-165.2) | 0.42 (-1.91-2.81) |
| CKD | Nigeria | 76055 (70880.2-81863.1) | 136 (125.2-148.4) | 210760 (196709.1-225476.3) | 181.3 (165.2-196.4) | 0.68 (-1.68-3.1) |
| CKD | Niue | 4 (3.6-4.3) | 182.6 (166.8-200.2) | 5.8 (5.2-6.5) | 277.9 (252.9-305.8) | 1.11 (-1.46-3.75) |
| CKD | North Macedonia | 2592.5 (2352.9-2866.8) | 135.2 (123.5-147.4) | 8708.4 (7721.2-9571.5) | 260.5 (236.7-282.5) | 1.86 (-0.69-4.48) |
| CKD | Northern Mariana Islands | 61.6 (54.6-67.7) | 239.9 (216.4-262.4) | 191.7 (170.2-217.4) | 340.3 (313-378.1) | 0.8 (-1.88-3.56) |
| CKD | Norway | 13125 (11762.9-14780.7) | 182.6 (166.4-202.6) | 21817.3 (19978.9-23671.4) | 212.3 (195.3-229.1) | 0.73 (-1.93-3.46) |
| CKD | Oman | 1753.6 (1613.6-1895.7) | 218.4 (197.2-237.9) | 9118.5 (8290.7-9915.3) | 396.6 (366.1-431.7) | 1.61 (-1.13-4.43) |
| CKD | Pakistan | 98897.9 (91760.5-107003.2) | 142.5 (131.4-155.3) | 275774 (256521-294772.5) | 181.9 (167.8-195.9) | 0.74 (-1.67-3.21) |
| CKD | Palau | 23.9 (21.5-26.8) | 213.2 (190.9-238.5) | 82 (74.2-92.8) | 348.8 (322.3-385.3) | 1.42 (-1.16-4.07) |
| CKD | Palestine | 2996.9 (2749.1-3267.8) | 290.3 (264.9-317.4) | 12369.2 (11404.5-13454.8) | 430.7 (398.7-464.2) | 0.97 (-1.77-3.79) |
| CKD | Panama | 3972.3 (3641.1-4354.2) | 243.7 (221.5-269.1) | 15955.6 (15016-17068.7) | 363.8 (341.5-389.3) | 1.21 (-1.46-3.95) |
| CKD | Papua New Guinea | 2465.7 (2242.7-2730.9) | 97.3 (87.8-108.6) | 8613.7 (7915.6-9315.3) | 128.7 (116.8-139.8) | 0.67 (-1.57-2.96) |
| CKD | Paraguay | 4761.1 (4372.7-5210.1) | 191.9 (174.6-210.7) | 18723.8 (17246.6-20324.5) | 307.8 (282.8-334.3) | 1.34 (-1.32-4.07) |
| CKD | Peru | 19680.4 (18106.3-21486.1) | 148.8 (136.1-163.5) | 95333.1 (88067.6-103496.1) | 284.1 (261.4-308.4) | 1.92 (-0.69-4.6) |
| CKD | Philippines | 66080.5 (61639.7-70710.3) | 184.5 (170.4-199.3) | 245592 (230482.9-262725.1) | 271.1 (254.4-289.2) | 1.09 (-1.39-3.64) |
| CKD | Poland | 63497 (56152.8-71143.4) | 149 (133.5-165.7) | 137937.1 (123814.9-154597.4) | 201.7 (183.5-223.1) | 0.17 (-2.3-2.7) |
| CKD | Portugal | 29571.5 (26316.1-33103.2) | 211.3 (190.2-233.8) | 60078.7 (54790.6-66349.8) | 236.4 (215.3-260.2) | 0.41 (-2.37-3.26) |
| CKD | Puerto Rico | 8257.3 (7527.1-9060.4) | 228.9 (208.6-251.4) | 21077.1 (19420.1-22999.4) | 354.7 (332.4-384.6) | 1.37 (-1.28-4.09) |
| CKD | Qatar | 462.9 (413-512.9) | 294.6 (267-326.9) | 6175 (5514.6-6879) | 467.4 (432.9-503.9) | 1.12 (-1.72-4.03) |
| CKD | Republic of Korea | 61022.7 (54616.5-68362.2) | 215 (193.8-239.3) | 226255.8 (210509.1-241377) | 242.8 (225.8-258.5) | 0.3 (-2.46-3.14) |
| CKD | Republic of Moldova | 2996 (2719.8-3308.8) | 69.4 (63.5-76.2) | 6550.7 (5869.7-7305.2) | 123.6 (112.7-136.2) | 1.37 (-0.87-3.65) |
| CKD | Romania | 30710.2 (27578.2-34445.4) | 113.1 (103.3-125.2) | 72834.4 (67186.2-78514.2) | 210.9 (196-225.8) | 1.62 (-0.88-4.19) |
| CKD | Russian Federation | 201891.9 (185153-222440.2) | 121.2 (112.8-131.7) | 422141.2 (385788-459284.6) | 201.9 (187.1-217.1) | 1.32 (-0.93-3.61) |
| CKD | Rwanda | 3596.3 (3325.6-3888.9) | 95.8 (88.1-104.8) | 8666.2 (7877.1-9399.3) | 120.8 (110-132.2) | 0.56 (-1.7-2.86) |
| CKD | Saint Kitts and Nevis | 81.1 (73.3-89.2) | 223.1 (203.6-245.7) | 252.7 (228.3-279.8) | 343.8 (319.5-374.2) | 1.35 (-1.27-4.04) |
| CKD | Saint Lucia | 190.3 (175.7-205.5) | 200.5 (185.5-216.2) | 736.5 (681.6-807) | 314.4 (293.1-343.5) | 1.26 (-1.35-3.94) |
| CKD | Saint Vincent and the Grenadines | 144.8 (133.8-157.4) | 189.1 (175.5-206.4) | 410.7 (373.1-444.6) | 294.1 (270.4-314.6) | 1.29 (-1.26-3.9) |
| CKD | Samoa | 196.3 (182.3-212.2) | 190.4 (176.4-206.4) | 433 (402.5-470.7) | 269.7 (250.3-292.6) | 0.84 (-1.73-3.48) |
| CKD | San Marino | 70.3 (62.7-78.6) | 195 (175.2-216.4) | 156.9 (140.7-174.3) | 207.3 (186.8-229.4) | 0.25 (-2.43-3.01) |
| CKD | Sao Tome and Principe | 130.3 (118.9-141.8) | 163.2 (148-178.8) | 325.1 (301.6-350) | 240.5 (222.6-260.8) | 1.07 (-1.34-3.55) |
| CKD | Saudi Arabia | 21157.4 (19638.5-22838.8) | 295.8 (272.1-320.4) | 123153.9 (113310.5-133631.7) | 495.8 (465.1-529.6) | 1.46 (-1.34-4.33) |
| CKD | Senegal | 5896.2 (5478.8-6340.6) | 136.1 (125.9-146.9) | 15909 (14807.5-17097.1) | 171.2 (158.9-185.5) | 0.48 (-1.9-2.92) |
| CKD | Serbia | 15572.4 (13985.4-17191.9) | 134.8 (123.2-146.9) | 39136.8 (35808.5-43024.5) | 246.2 (227-268.2) | 1.76 (-0.86-4.46) |
| CKD | Seychelles | 126.5 (116.7-137.7) | 217.2 (200.1-236.5) | 379.1 (345-413.9) | 315.6 (289.3-341.2) | 0.98 (-1.71-3.74) |
| CKD | Sierra Leone | 3353.4 (3107.8-3634) | 126.6 (115.6-139) | 7938.8 (7427.2-8505.2) | 163.2 (149.6-176.3) | 0.62 (-1.71-3.01) |
| CKD | Singapore | 5595.5 (5322-5869.4) | 254.1 (241.4-266.8) | 25251 (23412.7-27410.9) | 299 (279.2-325.6) | 0.5 (-2.28-3.37) |
| CKD | Slovakia | 8255.2 (7426.9-9097.6) | 141 (128.1-154.9) | 20773 (18944.8-22792.1) | 226.6 (207.1-244.6) | 1.24 (-1.26-3.8) |
| CKD | Slovenia | 3002.6 (2709.9-3342.5) | 126 (114-139.4) | 9212.8 (8321.9-10191.2) | 220.9 (200.9-243.5) | 1.53 (-1.04-4.17) |
| CKD | Solomon Islands | 347.3 (324.5-371.8) | 162.8 (150.8-175.9) | 825.5 (765.5-887.3) | 179 (165.2-193.5) | 0.02 (-2.34-2.45) |
| CKD | Somalia | 3438 (3199.7-3700.3) | 96.2 (88.6-104.4) | 10304.9 (9580.4-10986.7) | 113 (103-122.3) | 0.33 (-1.9-2.61) |
| CKD | South Africa | 40375.1 (37410-43474.6) | 172.9 (160.1-187.5) | 116840.4 (108462.8-124634.2) | 242.6 (225.1-257.9) | 0.77 (-1.75-3.35) |
| CKD | South Sudan | 3133.3 (2915-3386.3) | 97.8 (90.1-106.9) | 5862.5 (5451-6350.7) | 120.2 (110.5-131.2) | 0.46 (-1.78-2.76) |
| CKD | Spain | 132317 (121228.8-145627) | 237.8 (219.7-258.3) | 235483.7 (215973.6-254667.4) | 232.2 (211.9-250) | 0.07 (-2.74-2.97) |
| CKD | Sri Lanka | 21971.6 (20286.9-23967.3) | 186.6 (171.8-203.1) | 77126.6 (70183.9-84713.6) | 280.7 (257.9-305.2) | 1.05 (-1.57-3.74) |
| CKD | Sudan | 22886.2 (20725.1-25488.1) | 216.8 (195.3-241.9) | 72866.1 (66722.9-78875.5) | 334.1 (303.8-366.3) | 1.12 (-1.59-3.91) |
| CKD | Suriname | 539.8 (492.7-591.3) | 188.4 (171.8-206.6) | 1937.3 (1753.3-2093.3) | 303 (278.6-326.7) | 1.43 (-1.12-4.05) |
| CKD | Sweden | 28865.1 (26001.4-32781.7) | 180.3 (164.1-201.3) | 38458.2 (34199.8-43016.3) | 171.3 (155.2-188.6) | 0.1 (-2.55-2.82) |
| CKD | Switzerland | 25404.4 (23196.9-28054.3) | 237.3 (218.2-259.5) | 47327.8 (42803.5-51365.8) | 256.7 (233-278.1) | 0.3 (-2.51-3.19) |
| CKD | Syrian Arab Republic | 16986.7 (15685.7-18588.1) | 269.1 (247.1-295.8) | 56594 (52293.7-61422.5) | 407.4 (380.3-438.9) | 1.02 (-1.68-3.8) |
| CKD | Taiwan (Province of China) | 41232.5 (38269.3-44341.9) | 253.7 (236.8-271.7) | 123945.2 (116111.8-131502.4) | 302 (284.6-319) | 0.53 (-2.18-3.32) |
| CKD | Tajikistan | 3031.9 (2708-3348.2) | 79.4 (70.8-88.4) | 9225.7 (8357.3-10093.4) | 127.7 (116.1-140) | 1.43 (-0.77-3.67) |
| CKD | Thailand | 83500.2 (76620.3-91124.8) | 210 (193.6-228.5) | 317728.3 (294103.3-344410.7) | 301.4 (281.4-325.4) | 0.76 (-1.82-3.41) |
| CKD | Timor-Leste | 589.2 (544.6-639.2) | 148.9 (136.7-164.2) | 1926.9 (1773.1-2103.2) | 202.4 (186.1-220.2) | 0.82 (-1.64-3.33) |
| CKD | Togo | 2470.3 (2302.3-2654.4) | 137.2 (126.6-148.7) | 8279.4 (7640.9-8953.7) | 176.5 (160.1-192.2) | 0.52 (-1.85-2.94) |
| CKD | Tokelau | 2.1 (1.9-2.3) | 156.5 (144.1-170.1) | 3.6 (3.3-3.9) | 248.6 (228.6-269.1) | 1.15 (-1.4-3.77) |
| CKD | Tonga | 119.5 (109.9-131) | 184.6 (169.9-203.6) | 226.4 (209.3-243.3) | 264.4 (244.8-284.9) | 0.93 (-1.64-3.56) |
| CKD | Trinidad and Tobago | 1663.3 (1539.6-1810.5) | 183.3 (168.9-201) | 5777.7 (5276.7-6285.9) | 308.5 (285.2-332.5) | 1.54 (-1.04-4.18) |
| CKD | Tunisia | 13410.7 (12219.6-14805.2) | 249.5 (228.1-273.3) | 54366.1 (49513.8-59189.2) | 402.8 (370.6-435.3) | 1.17 (-1.65-4.08) |
| CKD | Turkey | 80830.6 (75539.3-86875.7) | 223.5 (208.2-239.6) | 384231 (352192.1-414423.5) | 404.1 (371.7-432.1) | 1.52 (-1.24-4.37) |
| CKD | Turkmenistan | 3148.7 (2914.4-3383.6) | 115.6 (107.1-125.7) | 8400.7 (7732.2-9037.5) | 179 (165.8-191.1) | 1.39 (-0.84-3.67) |
| CKD | Tuvalu | 11.3 (10.3-12.5) | 149.4 (137.4-164.8) | 26.3 (24.1-29) | 239.7 (221.7-260.7) | 1.19 (-1.3-3.75) |
| CKD | Uganda | 7431.5 (6917.6-7993.9) | 86.9 (79.9-94.5) | 19848.5 (18513.2-21371.4) | 106.7 (97.8-116.5) | 0.42 (-1.82-2.72) |
| CKD | Ukraine | 52974.2 (47074.9-59414.5) | 81.5 (73.6-89.9) | 82042.1 (75037.7-90446.9) | 123.5 (113.9-134.5) | 1.16 (-1.05-3.43) |
| CKD | United Arab Emirates | 2135.3 (1941.5-2336) | 327.3 (300.5-358.3) | 31937.9 (28287.9-36331.5) | 466.4 (433-505.6) | 0.99 (-1.74-3.8) |
| CKD | United Kingdom | 195682.3 (178100.4-218323.3) | 209 (191.3-229.7) | 290434 (265594.9-316835.7) | 219.7 (201.6-237.2) | 0.02 (-2.63-2.75) |
| CKD | United Republic of Tanzania | 14481.2 (13439.4-15616) | 104.1 (95.2-113.2) | 38552.8 (35789.5-41463) | 128.1 (117.7-139.1) | 0.44 (-1.82-2.76) |
| CKD | United States of America | 965461.6 (882901.2-1056988.9) | 303.4 (278-331) | 1842212.2 (1695726.4-1979975.5) | 323.2 (300.1-346.3) | 0.06 (-2.57-2.77) |
| CKD | United States Virgin Islands | 186.1 (171.4-201.9) | 201.4 (186-217.7) | 512.5 (466.8-564.6) | 315.1 (291.6-344) | 1.25 (-1.37-3.94) |
| CKD | Uruguay | 7727 (7006.5-8507.2) | 197.1 (179.8-215.5) | 13240.1 (12079.6-14386.1) | 238.3 (219.3-257.3) | 0.59 (-2.01-3.25) |
| CKD | Uzbekistan | 21869.3 (20280.2-23618.2) | 143.9 (133-157.3) | 62512.7 (56558.5-68430) | 205.7 (188.1-223.6) | 1.1 (-1.2-3.45) |
| CKD | Vanuatu | 123.5 (114.4-132.4) | 144.7 (133.5-157.4) | 447.4 (413.3-485.3) | 215.2 (198.3-231.4) | 0.94 (-1.54-3.48) |
| CKD | Venezuela (Bolivarian Republic of) | 27615.1 (25358.3-30283.8) | 246.2 (225.1-272.6) | 113852.1 (105688.7-122186.7) | 371.2 (346.1-396.5) | 1.21 (-1.52-4.02) |
| CKD | Viet Nam | 61002.9 (55831-66358.1) | 136.9 (125.1-149.2) | 215387.4 (195155.3-237672.2) | 214.7 (196.6-234.1) | 1.36 (-1.13-3.91) |
| CKD | Yemen | 12658.4 (11431.9-13948.4) | 213 (191.6-236.6) | 48348.7 (44355.6-52263.9) | 303.7 (277.2-331) | 0.98 (-1.7-3.73) |
| CKD | Zambia | 4312.5 (4015.1-4641.5) | 108.5 (100.2-118.2) | 12225.5 (11417.4-13119.8) | 137.1 (126.4-148.9) | 0.5 (-1.8-2.86) |
| CKD | Zimbabwe | 7853.7 (7224.3-8574.8) | 158.8 (145.4-175.6) | 15456 (14249.4-16766) | 189.5 (174-205.7) | 0.19 (-2.27-2.72) |
| CKD due to diabetes mellitus type 1 | Afghanistan | 156.1 (15.1-734.8) | 1 (0.2-4.4) | 614.9 (46.9-3017.8) | 1.3 (0.2-5.7) | 0.91 (-1.84-3.73) |
| CKD due to diabetes mellitus type 1 | Albania | 40 (4.8-186.2) | 1.1 (0.2-4.8) | 46.6 (15.9-129.1) | 2 (0.5-6.9) | 1.71 (-0.68-4.16) |
| CKD due to diabetes mellitus type 1 | Algeria | 365.1 (36.7-1700.4) | 1.1 (0.2-4.8) | 700.2 (123.2-2923.3) | 1.6 (0.3-6.5) | 1.19 (-1.58-4.04) |
| CKD due to diabetes mellitus type 1 | American Samoa | 0.8 (0-3.7) | 1.1 (0.1-5) | 0.6 (0.1-2.4) | 1.3 (0.1-6) | 1.01 (-1.62-3.71) |
| CKD due to diabetes mellitus type 1 | Andorra | 0.4 (0.2-1.1) | 0.9 (0.3-3) | 1.2 (0.6-2.3) | 1.2 (0.5-3.2) | 0.1 (-2.63-2.9) |
| CKD due to diabetes mellitus type 1 | Angola | 141.7 (8.3-719.2) | 0.8 (0.1-3.7) | 444.5 (31.5-2197.7) | 0.9 (0.1-4.1) | 0.82 (-1.46-3.14) |
| CKD due to diabetes mellitus type 1 | Antigua and Barbuda | 0.8 (0.2-3) | 1.3 (0.3-5) | 1.4 (0.5-4.1) | 1.9 (0.5-6.7) | 1.35 (-1.26-4.03) |
| CKD due to diabetes mellitus type 1 | Argentina | 368.8 (65.6-1502.9) | 1.1 (0.2-4.5) | 486.7 (137.3-1608.1) | 1.4 (0.3-5.2) | 0.76 (-1.89-3.48) |
| CKD due to diabetes mellitus type 1 | Armenia | 37.2 (5.2-158.1) | 1 (0.2-4.3) | 43.8 (13.9-132.9) | 1.7 (0.4-5.9) | 1.98 (-0.36-4.38) |
| CKD due to diabetes mellitus type 1 | Australia | 180.5 (67.3-493.2) | 1.1 (0.4-3.2) | 557.7 (240-1127.6) | 1.9 (0.7-4.7) | 0.53 (-2.2-3.35) |
| CKD due to diabetes mellitus type 1 | Austria | 81.5 (30.7-175) | 1.1 (0.3-2.8) | 202.5 (86.4-392.7) | 1.7 (0.6-3.6) | 0.75 (-1.99-3.58) |
| CKD due to diabetes mellitus type 1 | Azerbaijan | 123.4 (17-500.8) | 1.5 (0.2-5.9) | 239.3 (66.9-739.5) | 2.6 (0.6-9) | 1.98 (-0.29-4.31) |
| CKD due to diabetes mellitus type 1 | Bahamas | 3.3 (0.5-13.1) | 1.3 (0.3-5) | 5.4 (1.4-17.5) | 1.8 (0.3-6.6) | 1.27 (-1.29-3.9) |
| CKD due to diabetes mellitus type 1 | Bahrain | 6 (0.8-31.9) | 1.1 (0.2-5.3) | 17.6 (4.9-69.7) | 1.5 (0.3-6.9) | 1.18 (-1.68-4.13) |
| CKD due to diabetes mellitus type 1 | Bangladesh | 1094.9 (91.1-4917.1) | 0.7 (0.1-2.7) | 1309.6 (233.2-4953.1) | 0.9 (0.2-3.4) | 0.85 (-1.45-3.2) |
| CKD due to diabetes mellitus type 1 | Barbados | 3 (0.7-11.4) | 1.4 (0.3-5.5) | 4.4 (1.5-12.6) | 1.9 (0.4-7) | 1.31 (-1.25-3.95) |
| CKD due to diabetes mellitus type 1 | Belarus | 88 (16.4-330.5) | 1 (0.2-4.1) | 128.6 (45.9-349.2) | 1.7 (0.4-5.6) | 1.3 (-0.93-3.59) |
| CKD due to diabetes mellitus type 1 | Belgium | 81.6 (28.7-225.3) | 0.9 (0.2-3.2) | 142.9 (61.4-291.8) | 1.1 (0.4-3.1) | 0.2 (-2.55-3.04) |
| CKD due to diabetes mellitus type 1 | Belize | 3.5 (0.4-14.7) | 1.4 (0.3-5.3) | 8.6 (1.8-30.9) | 2.1 (0.4-7.6) | 1.35 (-1.18-3.94) |
| CKD due to diabetes mellitus type 1 | Benin | 98.8 (5.8-491.8) | 1.1 (0.1-5.3) | 247.5 (17.4-1225.9) | 1.2 (0.1-5.5) | 0.61 (-1.76-3.04) |
| CKD due to diabetes mellitus type 1 | Bermuda | 0.6 (0.2-2.1) | 1.2 (0.3-4.6) | 0.9 (0.3-2.1) | 1.7 (0.4-6.3) | 1.57 (-1.07-4.29) |
| CKD due to diabetes mellitus type 1 | Bhutan | 8.2 (0.7-40.4) | 0.9 (0.1-4.3) | 8.3 (1.7-31.8) | 1.2 (0.2-4.9) | 1.12 (-1.29-3.58) |
| CKD due to diabetes mellitus type 1 | Bolivia (Plurinational State of) | 94.9 (7.9-402.8) | 1 (0.2-4.2) | 152.4 (23.3-590.8) | 1.3 (0.2-5) | 1.13 (-1.44-3.77) |
| CKD due to diabetes mellitus type 1 | Bosnia and Herzegovina | 34.8 (8.3-116.2) | 0.9 (0.2-3.1) | 41.6 (16.1-99.4) | 1.5 (0.4-4.2) | 1.82 (-0.72-4.42) |
| CKD due to diabetes mellitus type 1 | Botswana | 15.9 (2.8-64.8) | 1 (0.3-3.4) | 30.7 (8.8-98.7) | 1.3 (0.4-4.1) | 0.84 (-1.65-3.4) |
| CKD due to diabetes mellitus type 1 | Brazil | 1861 (1011.6-3297.1) | 1.2 (0.7-2.1) | 3599.4 (2453-5115.2) | 1.7 (1.1-2.6) | 0.73 (-1.81-3.35) |
| CKD due to diabetes mellitus type 1 | Brunei Darussalam | 3.4 (0.6-12.5) | 1.2 (0.3-4) | 4.9 (1.2-16) | 1.3 (0.3-4.7) | 0.43 (-2.3-3.24) |
| CKD due to diabetes mellitus type 1 | Bulgaria | 77.3 (20.6-259.6) | 1.1 (0.2-4.3) | 132.5 (50-330.6) | 2.5 (0.7-7.9) | 1.73 (-0.7-4.22) |
| CKD due to diabetes mellitus type 1 | Burkina Faso | 183.5 (13.1-883.2) | 1.1 (0.2-5.1) | 474.4 (35.6-2412.9) | 1.3 (0.2-6.1) | 0.78 (-1.56-3.17) |
| CKD due to diabetes mellitus type 1 | Burundi | 79 (7.9-372.4) | 0.9 (0.2-3.9) | 169.8 (23.4-756.1) | 1 (0.2-4) | 0.33 (-1.91-2.61) |
| CKD due to diabetes mellitus type 1 | Cabo Verde | 5.8 (0.3-29.4) | 1.1 (0.1-5.1) | 5.7 (0.7-24.6) | 1.2 (0.1-5.4) | 1.1 (-1.26-3.52) |
| CKD due to diabetes mellitus type 1 | Cambodia | 177 (16.5-867.2) | 1.2 (0.2-5) | 224.6 (40.7-926.2) | 1.3 (0.2-5.3) | 0.89 (-1.49-3.33) |
| CKD due to diabetes mellitus type 1 | Cameroon | 248.8 (16.7-1202.6) | 1.5 (0.2-6.4) | 703 (65.9-3243.1) | 1.7 (0.3-7.1) | 0.66 (-1.77-3.16) |
| CKD due to diabetes mellitus type 1 | Canada | 554.7 (216.8-1216.1) | 2 (0.7-5.4) | 936.1 (410.5-1903.9) | 2.2 (0.8-6.1) | 0.34 (-2.23-2.97) |
| CKD due to diabetes mellitus type 1 | Central African Republic | 33.9 (2.3-159.3) | 0.7 (0.1-3.1) | 65.5 (5.5-301.4) | 0.8 (0.1-3.6) | 0.58 (-1.68-2.9) |
| CKD due to diabetes mellitus type 1 | Chad | 109.7 (7.1-562.3) | 1 (0.1-4.6) | 342.7 (23.8-1757.7) | 1.1 (0.2-5.1) | 0.48 (-1.86-2.87) |
| CKD due to diabetes mellitus type 1 | Chile | 138.7 (13.8-624.4) | 1 (0.1-4.3) | 187.1 (49.2-657.7) | 1.3 (0.2-5.3) | 1.19 (-1.54-4.01) |
| CKD due to diabetes mellitus type 1 | China | 9475.4 (4976.8-15577.5) | 0.9 (0.5-1.4) | 6321.3 (3948.9-9463.8) | 0.7 (0.4-1.1) | 0.25 (-2.21-2.76) |
| CKD due to diabetes mellitus type 1 | Colombia | 434.2 (29.4-2047.6) | 1.1 (0.1-4.9) | 501.7 (94.1-1997.8) | 1.3 (0.2-5.6) | 0.97 (-1.67-3.69) |
| CKD due to diabetes mellitus type 1 | Comoros | 7.1 (0.6-33.3) | 1 (0.2-4.1) | 8.6 (1.4-35.1) | 1.1 (0.2-4.3) | 0.38 (-1.89-2.7) |
| CKD due to diabetes mellitus type 1 | Congo | 31.1 (2.5-147.2) | 0.9 (0.1-3.8) | 59.2 (7.3-254.1) | 1 (0.2-4) | 0.82 (-1.52-3.21) |
| CKD due to diabetes mellitus type 1 | Cook Islands | 0.2 (0-1) | 0.9 (0.1-4.5) | 0.1 (0-0.6) | 1.1 (0.1-5.3) | 1.27 (-1.36-3.97) |
| CKD due to diabetes mellitus type 1 | Costa Rica | 48.1 (4.5-230.1) | 1.3 (0.2-5.9) | 52.9 (10.3-214.1) | 1.5 (0.2-6.6) | 0.16 (-2.67-3.07) |
| CKD due to diabetes mellitus type 1 | C么te d'Ivoire | 231.9 (16.4-1107.3) | 1.2 (0.2-5) | 499.7 (49.2-2266.1) | 1.3 (0.2-5.5) | 0.54 (-1.85-3) |
| CKD due to diabetes mellitus type 1 | Croatia | 32.9 (8.9-105) | 0.8 (0.2-3.1) | 68 (29.8-141.3) | 1.6 (0.6-4.6) | 1.64 (-0.99-4.34) |
| CKD due to diabetes mellitus type 1 | Cuba | 95.4 (12.5-412.2) | 1 (0.1-4.5) | 102.1 (27-365.9) | 1.3 (0.2-6) | 1.7 (-0.86-4.31) |
| CKD due to diabetes mellitus type 1 | Cyprus | 5.4 (1.3-20.1) | 0.8 (0.2-3) | 20.3 (8.7-42.4) | 1.4 (0.5-3.4) | 0.33 (-2.54-3.29) |
| CKD due to diabetes mellitus type 1 | Czechia | 71.9 (19.1-265.1) | 0.9 (0.2-3.6) | 139 (56.9-346.1) | 1.4 (0.4-4.7) | 1.35 (-1.2-3.96) |
| CKD due to diabetes mellitus type 1 | Democratic People's Republic of Korea | 209.2 (15.8-1065.2) | 0.9 (0.1-4.5) | 178.7 (30.6-762.6) | 1 (0.1-4.7) | 0.71 (-1.75-3.23) |
| CKD due to diabetes mellitus type 1 | Democratic Republic of the Congo | 514.6 (33-2459.4) | 0.8 (0.1-3.4) | 1031.8 (99.8-4682.3) | 0.9 (0.1-3.6) | 0.64 (-1.66-3) |
| CKD due to diabetes mellitus type 1 | Denmark | 59.8 (24.1-127.9) | 1.1 (0.4-3.2) | 115.4 (51.5-219.6) | 1.6 (0.6-3.8) | 0.64 (-2.02-3.38) |
| CKD due to diabetes mellitus type 1 | Djibouti | 5.1 (0.7-22.6) | 1 (0.2-3.5) | 15.3 (3.2-56.4) | 1.2 (0.3-4) | 0.59 (-1.66-2.9) |
| CKD due to diabetes mellitus type 1 | Dominica | 1.3 (0.2-5.6) | 1.5 (0.3-6.5) | 1.1 (0.3-4.3) | 2.3 (0.4-10.1) | 1.08 (-1.46-3.68) |
| CKD due to diabetes mellitus type 1 | Dominican Republic | 95.9 (9.9-449.5) | 1.1 (0.2-4.5) | 155.3 (28.8-611.7) | 1.5 (0.3-5.9) | 1.55 (-0.94-4.1) |
| CKD due to diabetes mellitus type 1 | Ecuador | 116.5 (11-529.6) | 0.9 (0.1-3.9) | 221.8 (36.8-909.5) | 1.3 (0.2-5.4) | 1.93 (-0.77-4.71) |
| CKD due to diabetes mellitus type 1 | Egypt | 915.2 (141.7-3755.7) | 1.4 (0.3-4.8) | 1994.5 (328.9-7874) | 1.7 (0.3-6.4) | 1.25 (-1.52-4.09) |
| CKD due to diabetes mellitus type 1 | El Salvador | 79.7 (8.2-363.1) | 1.1 (0.2-4.8) | 131.5 (23.6-537.9) | 2.1 (0.4-8.8) | 2.06 (-0.6-4.79) |
| CKD due to diabetes mellitus type 1 | Equatorial Guinea | 5.4 (0.3-26.1) | 0.7 (0.1-3.2) | 17.1 (1.7-71) | 1 (0.1-3.8) | 1.63 (-0.74-4.07) |
| CKD due to diabetes mellitus type 1 | Eritrea | 46.7 (5.7-217.9) | 0.9 (0.2-3.7) | 80.9 (13.1-345.3) | 1 (0.2-3.9) | 0.48 (-1.76-2.78) |
| CKD due to diabetes mellitus type 1 | Estonia | 17.2 (4.1-57.3) | 1.3 (0.2-4.7) | 26.4 (10.3-59.9) | 2.3 (0.7-6.7) | 2.1 (-0.27-4.53) |
| CKD due to diabetes mellitus type 1 | Eswatini | 14.3 (1.8-64.3) | 1.3 (0.4-4.9) | 21.8 (4.5-83.7) | 1.8 (0.5-6.2) | 0.54 (-1.92-3.07) |
| CKD due to diabetes mellitus type 1 | Ethiopia | 734.8 (205.3-2009.2) | 0.9 (0.3-2.2) | 1328.6 (444-3705.2) | 1 (0.4-2.5) | 0.38 (-1.86-2.66) |
| CKD due to diabetes mellitus type 1 | Fiji | 12.1 (1.2-53.5) | 1.4 (0.2-5.8) | 14.9 (2-63.2) | 1.6 (0.2-7) | 0.71 (-1.79-3.28) |
| CKD due to diabetes mellitus type 1 | Finland | 47.5 (19.1-116.2) | 0.9 (0.3-2.8) | 123.9 (59.2-231.7) | 1.6 (0.7-3.7) | 0.47 (-2.17-3.18) |
| CKD due to diabetes mellitus type 1 | France | 424.7 (141.3-1259.8) | 0.8 (0.2-2.9) | 978.4 (435.6-1901.7) | 1.2 (0.5-3.1) | 0.33 (-2.4-3.14) |
| CKD due to diabetes mellitus type 1 | Gabon | 13.2 (1.1-66.8) | 0.9 (0.1-4.4) | 23.3 (2.9-109.5) | 1.1 (0.2-5.2) | 1.05 (-1.35-3.51) |
| CKD due to diabetes mellitus type 1 | Gambia | 16.8 (1.2-84.5) | 1.1 (0.2-4.8) | 36.2 (3.8-173.5) | 1.2 (0.2-5.1) | 0.63 (-1.76-3.08) |
| CKD due to diabetes mellitus type 1 | Georgia | 55.1 (12.7-178) | 1.1 (0.2-3.7) | 51.9 (17.9-137.5) | 1.7 (0.5-5.1) | 1.56 (-0.76-3.94) |
| CKD due to diabetes mellitus type 1 | Germany | 772.5 (311.4-1917) | 1 (0.3-3.1) | 1403 (641-2763.4) | 1.3 (0.5-3.3) | 0.35 (-2.39-3.18) |
| CKD due to diabetes mellitus type 1 | Ghana | 207.6 (15.1-1010.1) | 0.9 (0.1-4) | 477.3 (51.3-2204.3) | 1.1 (0.2-4.9) | 0.92 (-1.48-3.37) |
| CKD due to diabetes mellitus type 1 | Greece | 67.8 (22-194.6) | 0.8 (0.2-2.9) | 128.6 (55.2-267.4) | 1.1 (0.3-3.1) | 0.07 (-2.76-2.99) |
| CKD due to diabetes mellitus type 1 | Greenland | 0.8 (0.3-2.7) | 1.7 (0.6-5) | 0.8 (0.3-2.2) | 1.4 (0.4-4.4) | 0.43 (-2.15-3.06) |
| CKD due to diabetes mellitus type 1 | Grenada | 1.5 (0.2-6.8) | 1.4 (0.3-6) | 1.8 (0.5-6.8) | 2.1 (0.4-8.9) | 1.46 (-1.13-4.12) |
| CKD due to diabetes mellitus type 1 | Guam | 1.6 (0.1-7.7) | 1 (0.1-4.7) | 2.1 (0.3-9.7) | 1.5 (0.2-7.4) | 1.15 (-1.39-3.75) |
| CKD due to diabetes mellitus type 1 | Guatemala | 168.9 (13.3-875.7) | 1.3 (0.2-5.9) | 312.8 (47.2-1340.2) | 2 (0.3-8.4) | 1.42 (-1.25-4.15) |
| CKD due to diabetes mellitus type 1 | Guinea | 114 (7.9-533.5) | 1.1 (0.2-4.9) | 240.6 (21-1140.6) | 1.3 (0.2-5.4) | 0.57 (-1.78-2.99) |
| CKD due to diabetes mellitus type 1 | Guinea-Bissau | 19.8 (1.5-101) | 1.2 (0.2-5.6) | 35.6 (3.5-170.9) | 1.3 (0.2-5.5) | 0.37 (-1.99-2.77) |
| CKD due to diabetes mellitus type 1 | Guyana | 11.7 (1.1-54.6) | 1.1 (0.2-4.9) | 11.9 (1.9-47.7) | 1.6 (0.2-6.4) | 1.35 (-1.21-3.97) |
| CKD due to diabetes mellitus type 1 | Haiti | 98.1 (11.5-463.2) | 1.1 (0.2-4.6) | 183.3 (31.5-738.7) | 1.3 (0.3-4.8) | 0.75 (-1.76-3.32) |
| CKD due to diabetes mellitus type 1 | Honduras | 79.5 (4.6-381.6) | 1.1 (0.1-4.8) | 127.1 (14.5-555.9) | 1.2 (0.2-5.1) | 0.82 (-1.85-3.57) |
| CKD due to diabetes mellitus type 1 | Hungary | 60.1 (15-235.3) | 0.8 (0.1-3.5) | 118.6 (48.2-302.3) | 1.4 (0.4-4.8) | 1.92 (-0.63-4.53) |
| CKD due to diabetes mellitus type 1 | Iceland | 1.9 (0.6-6) | 0.8 (0.2-2.6) | 3.3 (1.4-8.1) | 0.9 (0.3-2.6) | 0.14 (-2.54-2.89) |
| CKD due to diabetes mellitus type 1 | India | 10061.4 (5992.2-15823.7) | 1 (0.6-1.5) | 12161.7 (8065.1-18319) | 1 (0.6-1.5) | 0.2 (-2.29-2.75) |
| CKD due to diabetes mellitus type 1 | Indonesia | 2902.6 (1500.6-5453) | 1.4 (0.7-2.5) | 4601.4 (2847.2-7482.1) | 1.8 (1.1-3) | 0.85 (-1.44-3.19) |
| CKD due to diabetes mellitus type 1 | Iran (Islamic Republic of) | 1164 (682.6-1897.6) | 1.7 (1.1-2.6) | 1447.9 (1001.7-2101.3) | 2 (1.3-3) | 0.68 (-2.02-3.44) |
| CKD due to diabetes mellitus type 1 | Iraq | 335.5 (19.5-1708) | 1.2 (0.1-5.7) | 654.2 (74-3020.9) | 1.6 (0.2-7.1) | 1.1 (-1.69-3.96) |
| CKD due to diabetes mellitus type 1 | Ireland | 44.9 (14.3-137.8) | 1.3 (0.4-4.4) | 130.4 (60.3-254.9) | 2.3 (0.9-5.3) | 0.06 (-2.55-2.74) |
| CKD due to diabetes mellitus type 1 | Israel | 41.7 (9.9-150.9) | 0.8 (0.2-3) | 122.9 (42.7-334.3) | 1.2 (0.4-3.5) | 0.45 (-2.39-3.37) |
| CKD due to diabetes mellitus type 1 | Italy | 546.2 (397.5-738.1) | 1.1 (0.7-1.6) | 1348.4 (1030.6-1787.8) | 1.7 (1.2-2.3) | 0.19 (-2.44-2.9) |
| CKD due to diabetes mellitus type 1 | Jamaica | 31.8 (6-112.9) | 1.3 (0.3-4.2) | 37.2 (10.9-106) | 1.6 (0.4-5) | 1.11 (-1.47-3.77) |
| CKD due to diabetes mellitus type 1 | Japan | 777.2 (548.5-1103.2) | 0.8 (0.5-1.3) | 987.4 (757.8-1279.7) | 0.9 (0.6-1.4) | 0.09 (-2.7-2.96) |
| CKD due to diabetes mellitus type 1 | Jordan | 65.7 (5.1-332.1) | 1.2 (0.2-5.8) | 180 (26.8-846.6) | 1.6 (0.2-7.4) | 1.23 (-1.59-4.13) |
| CKD due to diabetes mellitus type 1 | Kazakhstan | 208.6 (24.6-996.8) | 1.2 (0.2-5.5) | 281.1 (55.8-1137.2) | 1.5 (0.3-6) | 1.58 (-0.73-3.95) |
| CKD due to diabetes mellitus type 1 | Kenya | 305.2 (192.7-459.4) | 1 (0.7-1.3) | 638.6 (464.8-890.8) | 1.2 (0.9-1.6) | 0.41 (-1.83-2.7) |
| CKD due to diabetes mellitus type 1 | Kiribati | 1.2 (0.1-5.4) | 1.1 (0.1-4.8) | 1.8 (0.2-7.9) | 1.3 (0.1-5.6) | 0.83 (-1.61-3.34) |
| CKD due to diabetes mellitus type 1 | Kuwait | 19.7 (3-86.7) | 1.2 (0.2-4.7) | 52 (14.9-172.6) | 1.5 (0.3-5.7) | 0.88 (-1.9-3.73) |
| CKD due to diabetes mellitus type 1 | Kyrgyzstan | 76.7 (8.1-322.3) | 1.4 (0.2-5.5) | 125.1 (21.6-436.4) | 1.7 (0.3-5.8) | 0.92 (-1.3-3.19) |
| CKD due to diabetes mellitus type 1 | Lao People's Democratic Republic | 80.4 (8.3-369.9) | 1.3 (0.2-5.4) | 122.4 (19-521.8) | 1.5 (0.3-6.4) | 0.77 (-1.67-3.28) |
| CKD due to diabetes mellitus type 1 | Latvia | 22 (4.1-87.4) | 1 (0.1-4.3) | 23.7 (8.4-63.3) | 1.7 (0.4-5.6) | 1.61 (-0.69-3.96) |
| CKD due to diabetes mellitus type 1 | Lebanon | 41.1 (4.6-190.6) | 1.1 (0.2-4.9) | 69.6 (14.1-272.2) | 1.5 (0.3-6.5) | 1.3 (-1.5-4.19) |
| CKD due to diabetes mellitus type 1 | Lesotho | 21 (4-85.3) | 1.2 (0.3-3.9) | 28.5 (7.1-99.7) | 1.5 (0.4-4.9) | 0.68 (-1.75-3.18) |
| CKD due to diabetes mellitus type 1 | Liberia | 42.3 (2.9-192.4) | 1 (0.1-4.3) | 79.4 (7.6-331.4) | 1.1 (0.2-4.5) | 0.88 (-1.51-3.34) |
| CKD due to diabetes mellitus type 1 | Libya | 62.2 (6.8-288.2) | 1.2 (0.2-4.7) | 83.8 (21.1-287.5) | 1.5 (0.3-5.9) | 1.06 (-1.74-3.94) |
| CKD due to diabetes mellitus type 1 | Lithuania | 34.9 (7-131.9) | 1.1 (0.2-4.4) | 35.4 (12.4-91.2) | 1.6 (0.4-5.5) | 1.08 (-1.19-3.41) |
| CKD due to diabetes mellitus type 1 | Luxembourg | 3.1 (1.2-8) | 0.9 (0.2-2.9) | 8.8 (4.3-17.7) | 1.2 (0.5-3.1) | 0.38 (-2.41-3.26) |
| CKD due to diabetes mellitus type 1 | Madagascar | 162.2 (18.8-737.7) | 0.9 (0.2-3.6) | 323.9 (49.3-1289.7) | 1 (0.2-3.4) | 0.41 (-1.79-2.66) |
| CKD due to diabetes mellitus type 1 | Malawi | 142.4 (18.8-650.6) | 1 (0.3-3.5) | 284.8 (55.9-1069) | 1.3 (0.4-4.1) | 0.64 (-1.61-2.94) |
| CKD due to diabetes mellitus type 1 | Malaysia | 210.5 (26-908.7) | 1 (0.2-4.1) | 364.7 (82-1323.2) | 1.3 (0.3-5) | 1.04 (-1.57-3.71) |
| CKD due to diabetes mellitus type 1 | Maldives | 4 (0.4-18.8) | 1.2 (0.2-4.9) | 6.4 (1.9-18.8) | 1.6 (0.4-5.5) | 1.18 (-1.41-3.83) |
| CKD due to diabetes mellitus type 1 | Mali | 162.6 (7.4-807.5) | 1 (0.1-4.8) | 449.4 (20.8-2249.2) | 1.1 (0.1-4.9) | 0.6 (-1.76-3) |
| CKD due to diabetes mellitus type 1 | Malta | 4.1 (1.4-10.4) | 1.1 (0.4-3.3) | 7.9 (3.3-16) | 1.5 (0.5-4.1) | 0.26 (-2.47-3.06) |
| CKD due to diabetes mellitus type 1 | Marshall Islands | 0.7 (0-3.5) | 1 (0.1-4.8) | 0.6 (0.1-3.1) | 1.1 (0.1-5.4) | 0.9 (-1.63-3.49) |
| CKD due to diabetes mellitus type 1 | Mauritania | 36.9 (3.1-175.6) | 1.2 (0.2-5) | 81.6 (10.1-366.3) | 1.5 (0.3-6) | 0.79 (-1.62-3.26) |
| CKD due to diabetes mellitus type 1 | Mauritius | 16.9 (3.2-62.3) | 1.5 (0.3-5.6) | 27.2 (8.6-73.9) | 2.6 (0.6-8.6) | 1.48 (-1.15-4.19) |
| CKD due to diabetes mellitus type 1 | Mexico | 1469.3 (848.9-2511.8) | 1.3 (0.8-2.2) | 2443.3 (1595.7-3784.9) | 2.1 (1.4-3.4) | 1.37 (-1.37-4.18) |
| CKD due to diabetes mellitus type 1 | Micronesia (Federated States of) | 1.7 (0.1-8.6) | 1.2 (0.1-5.7) | 1.5 (0.2-6.9) | 1.5 (0.2-6.9) | 1.15 (-1.4-3.77) |
| CKD due to diabetes mellitus type 1 | Monaco | 0.3 (0.1-0.7) | 0.9 (0.3-2.8) | 0.6 (0.3-1.2) | 1.2 (0.4-3) | 0.32 (-2.39-3.1) |
| CKD due to diabetes mellitus type 1 | Mongolia | 41.1 (4.3-203.3) | 1.4 (0.2-6.5) | 60.4 (11.6-250.6) | 1.7 (0.4-7.1) | 0.7 (-1.62-3.08) |
| CKD due to diabetes mellitus type 1 | Montenegro | 7.9 (1.9-28.2) | 1.4 (0.3-5.2) | 13.2 (5-32.2) | 2.3 (0.7-6.7) | 1.24 (-1.28-3.83) |
| CKD due to diabetes mellitus type 1 | Morocco | 319.4 (30.5-1481.4) | 1 (0.1-4.2) | 509.4 (85.4-2100.1) | 1.5 (0.2-6.4) | 1.62 (-1.1-4.43) |
| CKD due to diabetes mellitus type 1 | Mozambique | 199.3 (23.9-925.1) | 1 (0.2-3.9) | 495.2 (61-2149.9) | 1.2 (0.3-4.5) | 0.55 (-1.67-2.82) |
| CKD due to diabetes mellitus type 1 | Myanmar | 646.9 (83.9-2917.2) | 1.4 (0.2-5.8) | 966.6 (168.8-4015.6) | 1.8 (0.3-7.5) | 0.97 (-1.45-3.45) |
| CKD due to diabetes mellitus type 1 | Namibia | 17.5 (3.1-72.5) | 1 (0.3-3.6) | 31 (7.9-109) | 1.3 (0.4-4.1) | 0.59 (-1.89-3.12) |
| CKD due to diabetes mellitus type 1 | Nauru | 0.2 (0-0.9) | 1.1 (0.1-5.6) | 0.2 (0-0.9) | 1.4 (0.1-6.8) | 0.85 (-1.66-3.42) |
| CKD due to diabetes mellitus type 1 | Nepal | 251.7 (16.8-1330.9) | 0.8 (0.1-4.1) | 351.5 (49.9-1689.5) | 1.1 (0.2-5.4) | 1.2 (-1.28-3.74) |
| CKD due to diabetes mellitus type 1 | Netherlands | 110.6 (38.8-291) | 0.8 (0.2-2.7) | 290.7 (130.8-562.4) | 1.3 (0.5-3.3) | 0.62 (-2.11-3.43) |
| CKD due to diabetes mellitus type 1 | New Zealand | 35.2 (12.8-86.4) | 1.1 (0.4-2.9) | 79.1 (36-159.3) | 1.5 (0.6-3.6) | 0.55 (-2.16-3.34) |
| CKD due to diabetes mellitus type 1 | Nicaragua | 98.5 (6-454.9) | 1.6 (0.2-7.1) | 152.5 (21.6-629.8) | 2.3 (0.3-9.5) | 1.31 (-1.31-4) |
| CKD due to diabetes mellitus type 1 | Niger | 172.5 (8.5-804.2) | 1.1 (0.1-4.8) | 454 (28.9-2042.2) | 1.1 (0.1-4.3) | 0.42 (-1.91-2.81) |
| CKD due to diabetes mellitus type 1 | Nigeria | 1752.6 (1097.9-2750.6) | 1.2 (0.8-1.8) | 4275.5 (2674.1-6576.4) | 1.4 (0.9-2) | 0.68 (-1.68-3.1) |
| CKD due to diabetes mellitus type 1 | Niue | 0 (0-0.1) | 1 (0.1-4.7) | 0 (0-0.1) | 1.3 (0.1-6) | 1.11 (-1.46-3.75) |
| CKD due to diabetes mellitus type 1 | North Macedonia | 18.3 (4.4-60.3) | 1 (0.2-3.4) | 36.2 (14-79.7) | 1.8 (0.5-5) | 1.86 (-0.69-4.48) |
| CKD due to diabetes mellitus type 1 | Northern Mariana Islands | 0.5 (0.1-2.3) | 1.1 (0.2-4.8) | 0.6 (0.1-2.2) | 1.4 (0.2-6.1) | 0.8 (-1.88-3.56) |
| CKD due to diabetes mellitus type 1 | Norway | 33.7 (23.5-49) | 0.8 (0.5-1.2) | 94.8 (69.5-128.8) | 1.5 (1-2.2) | 0.73 (-1.93-3.46) |
| CKD due to diabetes mellitus type 1 | Oman | 28 (4.7-112.7) | 1.2 (0.4-3.8) | 81.1 (25.8-241.6) | 1.9 (0.7-5.6) | 1.61 (-1.13-4.43) |
| CKD due to diabetes mellitus type 1 | Pakistan | 1665.4 (411.6-4780.6) | 1.1 (0.3-2.8) | 3734 (1154.9-9816.2) | 1.4 (0.5-3.4) | 0.74 (-1.67-3.21) |
| CKD due to diabetes mellitus type 1 | Palau | 0.2 (0-0.9) | 1.3 (0.1-6.2) | 0.2 (0-0.9) | 1.9 (0.2-8.9) | 1.42 (-1.16-4.07) |
| CKD due to diabetes mellitus type 1 | Palestine | 45.4 (4.2-228.6) | 1.5 (0.3-6.3) | 105 (17.6-457) | 1.9 (0.4-7.7) | 0.97 (-1.77-3.79) |
| CKD due to diabetes mellitus type 1 | Panama | 30.4 (3.5-138.7) | 1.1 (0.2-4.9) | 64.3 (12.7-250.1) | 1.7 (0.3-6.7) | 1.21 (-1.46-3.95) |
| CKD due to diabetes mellitus type 1 | Papua New Guinea | 55.6 (2.7-266.2) | 0.9 (0.1-4.2) | 146.7 (9.3-707.1) | 1 (0.1-4.7) | 0.67 (-1.57-2.96) |
| CKD due to diabetes mellitus type 1 | Paraguay | 49.1 (4.8-225.4) | 0.9 (0.1-3.8) | 96.2 (17.8-359.7) | 1.4 (0.3-5.4) | 1.34 (-1.32-4.07) |
| CKD due to diabetes mellitus type 1 | Peru | 230 (20.1-1145.8) | 0.8 (0.1-3.9) | 452.8 (92-1913.6) | 1.3 (0.3-5.7) | 1.92 (-0.69-4.6) |
| CKD due to diabetes mellitus type 1 | Philippines | 1033.8 (716.1-1479.9) | 1.3 (1-1.8) | 2400.2 (1812.7-3172.9) | 2.1 (1.6-2.8) | 1.09 (-1.39-3.64) |
| CKD due to diabetes mellitus type 1 | Poland | 418 (232.1-696.7) | 1.3 (0.7-2.3) | 405.5 (287.1-575.1) | 1.4 (0.8-2.2) | 0.17 (-2.3-2.7) |
| CKD due to diabetes mellitus type 1 | Portugal | 56.4 (18.3-163.6) | 0.7 (0.2-2.5) | 97.3 (42.1-191.9) | 0.9 (0.3-2.6) | 0.41 (-2.37-3.26) |
| CKD due to diabetes mellitus type 1 | Puerto Rico | 45.4 (9.9-176.2) | 1.3 (0.3-5.4) | 45.8 (18.4-111.8) | 2 (0.5-7.9) | 1.37 (-1.28-4.09) |
| CKD due to diabetes mellitus type 1 | Qatar | 5.4 (0.8-23.2) | 1.1 (0.2-4.6) | 36.5 (10.6-123.7) | 1.6 (0.4-6) | 1.12 (-1.72-4.03) |
| CKD due to diabetes mellitus type 1 | Republic of Korea | 286.4 (52.7-1186) | 0.8 (0.1-3.3) | 243 (101.3-589.4) | 0.7 (0.1-2.7) | 0.3 (-2.46-3.14) |
| CKD due to diabetes mellitus type 1 | Republic of Moldova | 29.8 (4.4-114.4) | 0.7 (0.1-2.7) | 31 (10.2-85.6) | 1.2 (0.3-4) | 1.37 (-0.87-3.65) |
| CKD due to diabetes mellitus type 1 | Romania | 162.9 (32.6-621.9) | 0.8 (0.1-3.4) | 230.1 (85-570.5) | 1.4 (0.4-4.6) | 1.62 (-0.88-4.19) |
| CKD due to diabetes mellitus type 1 | Russian Federation | 2394.4 (1760.5-3193) | 2 (1.4-2.7) | 3230.9 (2583.1-4028.8) | 2.7 (2.1-3.6) | 1.32 (-0.93-3.61) |
| CKD due to diabetes mellitus type 1 | Rwanda | 100.5 (11.2-438.8) | 1 (0.2-3.6) | 167.3 (31-642.6) | 1.2 (0.3-3.9) | 0.56 (-1.7-2.86) |
| CKD due to diabetes mellitus type 1 | Saint Kitts and Nevis | 0.6 (0.1-2.2) | 1.3 (0.3-4.9) | 0.9 (0.3-2.8) | 1.9 (0.4-7.3) | 1.35 (-1.27-4.04) |
| CKD due to diabetes mellitus type 1 | Saint Lucia | 1.8 (0.2-8.4) | 1.1 (0.2-5) | 2.1 (0.6-7.2) | 1.6 (0.3-6.8) | 1.26 (-1.35-3.94) |
| CKD due to diabetes mellitus type 1 | Saint Vincent and the Grenadines | 1.6 (0.2-7) | 1.3 (0.2-5.5) | 1.7 (0.4-6.2) | 1.9 (0.3-7.8) | 1.29 (-1.26-3.9) |
| CKD due to diabetes mellitus type 1 | Samoa | 2.6 (0.2-12.1) | 1.1 (0.1-4.8) | 3.5 (0.3-15.5) | 1.3 (0.1-5.4) | 0.84 (-1.73-3.48) |
| CKD due to diabetes mellitus type 1 | San Marino | 0.2 (0.1-0.4) | 0.8 (0.2-2.7) | 0.4 (0.2-0.9) | 1.1 (0.3-2.9) | 0.25 (-2.43-3.01) |
| CKD due to diabetes mellitus type 1 | Sao Tome and Principe | 2.6 (0.2-14.2) | 1.4 (0.2-7) | 4.3 (0.4-20.9) | 1.8 (0.2-8.2) | 1.07 (-1.34-3.55) |
| CKD due to diabetes mellitus type 1 | Saudi Arabia | 242.1 (31.9-998.4) | 1.3 (0.3-4.6) | 749.3 (220.6-2125.4) | 2.3 (0.6-7.3) | 1.46 (-1.34-4.33) |
| CKD due to diabetes mellitus type 1 | Senegal | 136.1 (8.9-639) | 1 (0.1-4.6) | 229.4 (23.3-1020) | 1.1 (0.2-4.6) | 0.48 (-1.9-2.92) |
| CKD due to diabetes mellitus type 1 | Serbia | 68.7 (16.4-232.9) | 0.8 (0.2-3.1) | 120.8 (46.6-249) | 1.4 (0.4-3.9) | 1.76 (-0.86-4.46) |
| CKD due to diabetes mellitus type 1 | Seychelles | 0.8 (0.2-3.4) | 1.1 (0.3-4.3) | 1.2 (0.3-4.5) | 1.3 (0.3-5.4) | 0.98 (-1.71-3.74) |
| CKD due to diabetes mellitus type 1 | Sierra Leone | 75.9 (4.7-361) | 1.1 (0.1-4.6) | 145.6 (11.7-659.9) | 1.2 (0.2-5) | 0.62 (-1.71-3.01) |
| CKD due to diabetes mellitus type 1 | Singapore | 20 (2.7-89.5) | 0.8 (0.1-3.9) | 41.1 (12.7-129.9) | 1 (0.2-4.3) | 0.5 (-2.28-3.37) |
| CKD due to diabetes mellitus type 1 | Slovakia | 46.7 (9.3-182.2) | 1 (0.2-4.3) | 81.3 (29.3-200.5) | 1.7 (0.5-5.6) | 1.24 (-1.26-3.8) |
| CKD due to diabetes mellitus type 1 | Slovenia | 12 (2.8-41.6) | 0.8 (0.1-3.1) | 24.9 (10.1-60.2) | 1.3 (0.4-4.2) | 1.53 (-1.04-4.17) |
| CKD due to diabetes mellitus type 1 | Solomon Islands | 6.4 (0.6-27) | 1.3 (0.2-4.8) | 10.9 (1.3-44.5) | 1.3 (0.2-4.9) | 0.02 (-2.34-2.45) |
| CKD due to diabetes mellitus type 1 | Somalia | 113.8 (11.5-551.5) | 1 (0.2-3.9) | 334.3 (34.6-1571.6) | 1.1 (0.2-4.2) | 0.33 (-1.9-2.61) |
| CKD due to diabetes mellitus type 1 | South Africa | 517.9 (217.2-1019.4) | 1.2 (0.6-2.3) | 776.5 (427.4-1339.4) | 1.5 (0.8-2.5) | 0.77 (-1.75-3.35) |
| CKD due to diabetes mellitus type 1 | South Sudan | 78.9 (9.8-379.6) | 1 (0.2-3.8) | 153.2 (20.4-709.9) | 1.2 (0.3-4.5) | 0.46 (-1.78-2.76) |
| CKD due to diabetes mellitus type 1 | Spain | 219.3 (74.1-611.5) | 0.7 (0.2-2.5) | 456.7 (217.3-868.8) | 0.8 (0.3-2.1) | 0.07 (-2.74-2.97) |
| CKD due to diabetes mellitus type 1 | Sri Lanka | 186.8 (31.3-831.4) | 1.1 (0.2-4.7) | 252.1 (65.2-923.3) | 1.3 (0.3-5.4) | 1.05 (-1.57-3.74) |
| CKD due to diabetes mellitus type 1 | Sudan | 290.8 (25-1462.5) | 1 (0.2-4.3) | 662 (88.3-3042.8) | 1.3 (0.3-5.6) | 1.12 (-1.59-3.91) |
| CKD due to diabetes mellitus type 1 | Suriname | 5.1 (0.5-24) | 1.2 (0.2-5.5) | 8.4 (1.3-35.6) | 1.7 (0.2-7.7) | 1.43 (-1.12-4.05) |
| CKD due to diabetes mellitus type 1 | Sweden | 73.6 (35.1-141.7) | 0.8 (0.3-1.9) | 127.4 (74.5-208.3) | 0.9 (0.5-1.8) | 0.1 (-2.55-2.82) |
| CKD due to diabetes mellitus type 1 | Switzerland | 44.7 (15.1-125) | 0.8 (0.2-2.7) | 88.7 (37.9-174.5) | 0.9 (0.3-2.6) | 0.3 (-2.51-3.19) |
| CKD due to diabetes mellitus type 1 | Syrian Arab Republic | 257.7 (30.1-1080.7) | 1.5 (0.3-5.4) | 246.4 (76.1-752.8) | 1.8 (0.5-6) | 1.02 (-1.68-3.8) |
| CKD due to diabetes mellitus type 1 | Taiwan (Province of China) | 174.1 (50.7-579.1) | 1 (0.3-3.5) | 264 (113.2-599.4) | 1.4 (0.3-4.7) | 0.53 (-2.18-3.32) |
| CKD due to diabetes mellitus type 1 | Tajikistan | 77.7 (7.1-373.3) | 1 (0.2-4.5) | 159.6 (26.1-655.1) | 1.4 (0.3-5.4) | 1.43 (-0.77-3.67) |
| CKD due to diabetes mellitus type 1 | Thailand | 844.4 (151.9-3778.4) | 1.6 (0.3-6.9) | 735.8 (270.1-2299) | 1.4 (0.3-6) | 0.76 (-1.82-3.41) |
| CKD due to diabetes mellitus type 1 | Timor-Leste | 13.6 (1.3-67.2) | 1.1 (0.2-4.9) | 20.7 (2.5-97.6) | 1.2 (0.2-5.4) | 0.82 (-1.64-3.33) |
| CKD due to diabetes mellitus type 1 | Togo | 68.1 (5.5-319.2) | 1.2 (0.2-4.9) | 132.7 (18.2-551.2) | 1.3 (0.3-5) | 0.52 (-1.85-2.94) |
| CKD due to diabetes mellitus type 1 | Tokelau | 0 (0-0.1) | 0.9 (0.1-4.1) | 0 (0-0.1) | 1.1 (0.1-4.8) | 1.15 (-1.4-3.77) |
| CKD due to diabetes mellitus type 1 | Tonga | 1.4 (0.1-6.5) | 1 (0.1-4.3) | 1.7 (0.1-7.4) | 1.3 (0.1-5.3) | 0.93 (-1.64-3.56) |
| CKD due to diabetes mellitus type 1 | Trinidad and Tobago | 14.6 (2.1-67.1) | 1.2 (0.2-5.3) | 17 (4.4-62.3) | 1.7 (0.3-7.1) | 1.54 (-1.04-4.18) |
| CKD due to diabetes mellitus type 1 | Tunisia | 90.5 (12.8-386.7) | 1 (0.2-3.8) | 130.9 (35.7-428.6) | 1.3 (0.3-4.6) | 1.17 (-1.65-4.08) |
| CKD due to diabetes mellitus type 1 | Turkey | 710.8 (121.5-2803.6) | 1.1 (0.2-4.2) | 1160.4 (378-3300.4) | 1.6 (0.4-5.4) | 1.52 (-1.24-4.37) |
| CKD due to diabetes mellitus type 1 | Turkmenistan | 75.1 (9.4-305.1) | 1.6 (0.3-5.9) | 131.2 (27.4-456.9) | 2.5 (0.5-8.6) | 1.39 (-0.84-3.67) |
| CKD due to diabetes mellitus type 1 | Tuvalu | 0.2 (0-0.8) | 1.1 (0.1-5.3) | 0.2 (0-0.8) | 1.3 (0.2-6.2) | 1.19 (-1.3-3.75) |
| CKD due to diabetes mellitus type 1 | Uganda | 235.8 (20.9-1118) | 0.8 (0.2-3.3) | 545.8 (61.3-2493.1) | 1 (0.2-3.8) | 0.42 (-1.82-2.72) |
| CKD due to diabetes mellitus type 1 | Ukraine | 383 (82.1-1504) | 0.9 (0.1-3.9) | 468.9 (164.7-1291.8) | 1.5 (0.4-5.3) | 1.16 (-1.05-3.43) |
| CKD due to diabetes mellitus type 1 | United Arab Emirates | 33 (6.4-122) | 1.7 (0.5-5.7) | 245.3 (103.5-527.1) | 2.7 (0.8-8.1) | 0.99 (-1.74-3.8) |
| CKD due to diabetes mellitus type 1 | United Kingdom | 491.6 (401.6-630.4) | 1 (0.7-1.3) | 632.3 (534.8-763.3) | 1 (0.8-1.3) | 0.02 (-2.63-2.75) |
| CKD due to diabetes mellitus type 1 | United Republic of Tanzania | 434.6 (50.4-1848.3) | 1.2 (0.3-4.1) | 924 (136.3-3714.1) | 1.3 (0.3-4.5) | 0.44 (-1.82-2.76) |
| CKD due to diabetes mellitus type 1 | United States of America | 4146.8 (2995.9-6166.3) | 1.8 (1.3-2.8) | 5204.4 (4081.8-6884.2) | 1.8 (1.3-2.7) | 0.06 (-2.57-2.77) |
| CKD due to diabetes mellitus type 1 | United States Virgin Islands | 1.3 (0.2-5.3) | 1.2 (0.2-4.8) | 0.9 (0.3-2.9) | 1.6 (0.3-6.1) | 1.25 (-1.37-3.94) |
| CKD due to diabetes mellitus type 1 | Uruguay | 32 (6.9-135.4) | 1.1 (0.2-4.8) | 35.4 (10.8-115.1) | 1.3 (0.3-5.4) | 0.59 (-2.01-3.25) |
| CKD due to diabetes mellitus type 1 | Uzbekistan | 444.2 (64-1771) | 1.7 (0.4-6.1) | 904.9 (207.8-2892.8) | 2.6 (0.6-8.4) | 1.1 (-1.2-3.45) |
| CKD due to diabetes mellitus type 1 | Vanuatu | 2.3 (0.2-10.7) | 0.9 (0.1-4.1) | 4.4 (0.4-20.3) | 1.1 (0.1-5) | 0.94 (-1.54-3.48) |
| CKD due to diabetes mellitus type 1 | Venezuela (Bolivarian Republic of) | 254.8 (23.9-1177.4) | 1.1 (0.1-4.7) | 328.4 (62.5-1263.9) | 1.4 (0.2-5.5) | 1.21 (-1.52-4.02) |
| CKD due to diabetes mellitus type 1 | Viet Nam | 765.1 (100.5-3616.9) | 0.9 (0.2-4.1) | 1270.5 (351.8-4389.3) | 1.4 (0.3-5.1) | 1.36 (-1.13-3.91) |
| CKD due to diabetes mellitus type 1 | Yemen | 212 (18.7-1078.4) | 0.9 (0.2-4.1) | 493 (68.3-2263.2) | 1.2 (0.3-5.1) | 0.98 (-1.7-3.73) |
| CKD due to diabetes mellitus type 1 | Zambia | 124.5 (11.4-653.4) | 1 (0.2-4.4) | 262.8 (31.9-1321.6) | 1.1 (0.2-4.7) | 0.5 (-1.8-2.86) |
| CKD due to diabetes mellitus type 1 | Zimbabwe | 137.1 (19-679.3) | 1 (0.3-4.1) | 209.3 (37.7-960.8) | 1.2 (0.3-4.6) | 0.19 (-2.27-2.72) |
| CKD due to diabetes mellitus type 2 | Afghanistan | 1859.3 (1574.7-2205.1) | 26.1 (22.3-30.2) | 3501 (3005.1-4112.8) | 36.7 (32-42.4) | 0.91 (-1.84-3.73) |
| CKD due to diabetes mellitus type 2 | Albania | 253.1 (212.7-295.7) | 12 (10.2-13.9) | 925 (805.7-1064.7) | 20.3 (17.8-23.3) | 1.71 (-0.68-4.16) |
| CKD due to diabetes mellitus type 2 | Algeria | 3390.5 (2902.3-3947.2) | 26.4 (22.8-30.7) | 15599.8 (13633.4-17933.4) | 42.5 (37.4-48.3) | 1.19 (-1.58-4.04) |
| CKD due to diabetes mellitus type 2 | American Samoa | 5.2 (4.4-6.2) | 21.7 (18.8-25) | 15.7 (13.5-18.3) | 30.7 (26.7-35.2) | 1.01 (-1.62-3.71) |
| CKD due to diabetes mellitus type 2 | Andorra | 13.3 (11.4-15.3) | 22.9 (19.7-26.1) | 34 (29.6-38.7) | 22.2 (19.3-25.4) | 0.1 (-2.63-2.9) |
| CKD due to diabetes mellitus type 2 | Angola | 340.7 (293.2-398.5) | 8.8 (7.7-10.1) | 1474.3 (1250.8-1712.5) | 12.5 (10.9-14.3) | 0.82 (-1.46-3.14) |
| CKD due to diabetes mellitus type 2 | Antigua and Barbuda | 11.1 (9.7-12.7) | 21.9 (18.8-25.2) | 39 (33.8-44.2) | 34.5 (30.1-38.9) | 1.35 (-1.26-4.03) |
| CKD due to diabetes mellitus type 2 | Argentina | 7205.4 (6167.5-8364.7) | 22.1 (18.9-25.5) | 15868.1 (13881-17970.1) | 27.8 (24.3-31.5) | 0.76 (-1.89-3.48) |
| CKD due to diabetes mellitus type 2 | Armenia | 234.3 (193.6-282.1) | 8.2 (7-9.8) | 766.8 (657.3-899.5) | 17.3 (15.1-20) | 1.98 (-0.36-4.38) |
| CKD due to diabetes mellitus type 2 | Australia | 5450.9 (4883.2-6089.4) | 26.5 (23.7-29.6) | 14077 (12351.6-15833.2) | 30.1 (26.4-34) | 0.53 (-2.2-3.35) |
| CKD due to diabetes mellitus type 2 | Austria | 2721.4 (2340.9-3152.6) | 21.8 (18.9-25.5) | 5071.5 (4452.9-5782.1) | 27.5 (24.2-31.4) | 0.75 (-1.99-3.58) |
| CKD due to diabetes mellitus type 2 | Azerbaijan | 478.8 (396-572.7) | 9 (7.6-10.6) | 2054.1 (1705-2438) | 18 (15.2-20.9) | 1.98 (-0.29-4.31) |
| CKD due to diabetes mellitus type 2 | Bahamas | 31.2 (27.3-36.3) | 19.5 (17.1-22.4) | 125.1 (109.1-143.7) | 28.8 (25.3-32.7) | 1.27 (-1.29-3.9) |
| CKD due to diabetes mellitus type 2 | Bahrain | 58.1 (49.8-68) | 31.9 (27.6-36.5) | 517.4 (438-611.8) | 50 (43.6-57) | 1.18 (-1.68-4.13) |
| CKD due to diabetes mellitus type 2 | Bangladesh | 4286 (3670.1-5006.1) | 9 (7.8-10.4) | 18391.5 (15656.2-21277.2) | 13 (11.1-15) | 0.85 (-1.45-3.2) |
| CKD due to diabetes mellitus type 2 | Barbados | 52.5 (46-60.5) | 19.2 (16.6-22.2) | 157.4 (137.8-180.4) | 30.4 (26.6-34.9) | 1.31 (-1.25-3.95) |
| CKD due to diabetes mellitus type 2 | Belarus | 972.8 (830.8-1123.6) | 7.5 (6.4-8.7) | 1941.3 (1671.2-2229.4) | 12.3 (10.6-14) | 1.3 (-0.93-3.59) |
| CKD due to diabetes mellitus type 2 | Belgium | 3947.1 (3417-4547.3) | 24.5 (21.2-28.3) | 5952.2 (5216.3-6676.3) | 25.1 (22.1-28.1) | 0.2 (-2.55-3.04) |
| CKD due to diabetes mellitus type 2 | Belize | 17.8 (15.1-20.8) | 19 (16.1-22.1) | 95.6 (81.6-110.4) | 30.1 (26.1-34.5) | 1.35 (-1.18-3.94) |
| CKD due to diabetes mellitus type 2 | Benin | 265 (226.9-305.6) | 13.1 (11.4-15) | 888.8 (758.7-1030.7) | 17.1 (14.8-19.7) | 0.61 (-1.76-3.04) |
| CKD due to diabetes mellitus type 2 | Bermuda | 12.5 (10.7-14.4) | 19.5 (16.7-22.7) | 42.9 (37-48.6) | 33 (28.5-37.4) | 1.57 (-1.07-4.29) |
| CKD due to diabetes mellitus type 2 | Bhutan | 31.4 (26-37.3) | 12 (10.2-14) | 110.6 (93.6-128.6) | 17.9 (15.2-20.7) | 1.12 (-1.29-3.58) |
| CKD due to diabetes mellitus type 2 | Bolivia (Plurinational State of) | 591.6 (516.2-680.6) | 18.3 (16-20.9) | 2493 (2181.9-2861.5) | 26.9 (23.7-30.8) | 1.13 (-1.44-3.77) |
| CKD due to diabetes mellitus type 2 | Bosnia and Herzegovina | 512.7 (430.1-604.9) | 12 (10.2-13.9) | 1483.3 (1297.2-1695.1) | 23.4 (20.4-26.5) | 1.82 (-0.72-4.42) |
| CKD due to diabetes mellitus type 2 | Botswana | 85.8 (73.8-100.4) | 15.1 (13.1-17.2) | 323 (279.7-373.8) | 21.6 (18.9-24.8) | 0.84 (-1.65-3.4) |
| CKD due to diabetes mellitus type 2 | Brazil | 17679.4 (15797-19736) | 19.2 (17.2-21.4) | 64619.4 (59021.7-70002.9) | 25.4 (23.2-27.5) | 0.73 (-1.81-3.35) |
| CKD due to diabetes mellitus type 2 | Brunei Darussalam | 30.5 (26.5-35) | 31.8 (27.7-36.1) | 117.4 (101.1-135.4) | 34.5 (30.1-39.1) | 0.43 (-2.3-3.24) |
| CKD due to diabetes mellitus type 2 | Bulgaria | 1638.8 (1401.9-1924.6) | 12.6 (10.9-14.8) | 3163.4 (2770.9-3638.4) | 22.3 (19.7-25.8) | 1.73 (-0.7-4.22) |
| CKD due to diabetes mellitus type 2 | Burkina Faso | 531.7 (462.5-609.6) | 12.1 (10.5-13.7) | 1481.4 (1285.6-1674.5) | 15.9 (13.9-17.9) | 0.78 (-1.56-3.17) |
| CKD due to diabetes mellitus type 2 | Burundi | 209.3 (180.5-239.1) | 9 (7.8-10.3) | 521 (445.2-595.7) | 10.9 (9.5-12.4) | 0.33 (-1.91-2.61) |
| CKD due to diabetes mellitus type 2 | Cabo Verde | 25.3 (22-28.8) | 11 (9.5-12.5) | 75.3 (63.9-86.5) | 17.3 (14.9-19.9) | 1.1 (-1.26-3.52) |
| CKD due to diabetes mellitus type 2 | Cambodia | 534.5 (448.9-623.7) | 11.5 (9.6-13.2) | 2230.5 (1884.2-2604.2) | 17.2 (14.7-20) | 0.89 (-1.49-3.33) |
| CKD due to diabetes mellitus type 2 | Cameroon | 771 (654.9-906.3) | 17 (14.9-19.4) | 2960.2 (2544.7-3406.9) | 22.8 (20.1-26.1) | 0.66 (-1.77-3.16) |
| CKD due to diabetes mellitus type 2 | Canada | 8206.2 (7165.3-9256.1) | 24.5 (21.4-27.6) | 18560 (16177.2-21497.4) | 24.9 (21.7-28.7) | 0.34 (-2.23-2.97) |
| CKD due to diabetes mellitus type 2 | Central African Republic | 101.3 (86.3-120.1) | 8.8 (7.6-10.1) | 248.2 (211-287.8) | 11.1 (9.8-12.5) | 0.58 (-1.68-2.9) |
| CKD due to diabetes mellitus type 2 | Chad | 356.9 (304-415.1) | 12.4 (10.6-14.3) | 881.4 (758.5-1029.3) | 15.2 (13.2-17.4) | 0.48 (-1.86-2.87) |
| CKD due to diabetes mellitus type 2 | Chile | 2228 (1913.1-2576.3) | 22.5 (19.4-26) | 8699 (7608.7-9893.4) | 33.4 (29.2-38) | 1.19 (-1.54-4.01) |
| CKD due to diabetes mellitus type 2 | China | 127560.9 (112717.8-142654.5) | 15.1 (13.5-16.8) | 354156.6 (321264.6-382784) | 16.3 (14.9-17.5) | 0.25 (-2.21-2.76) |
| CKD due to diabetes mellitus type 2 | Colombia | 4334.4 (3680-5101.2) | 23.8 (20.4-27.7) | 17991 (15654.9-20822.5) | 32.5 (28.3-37.4) | 0.97 (-1.67-3.69) |
| CKD due to diabetes mellitus type 2 | Comoros | 19.4 (16.4-22.3) | 9.9 (8.5-11.2) | 59.3 (51.6-67.8) | 12.2 (10.6-13.9) | 0.38 (-1.89-2.7) |
| CKD due to diabetes mellitus type 2 | Congo | 105.1 (89.5-122.8) | 9.7 (8.4-11.1) | 388.7 (329.9-452.5) | 14.2 (12.4-16.2) | 0.82 (-1.52-3.21) |
| CKD due to diabetes mellitus type 2 | Cook Islands | 2.3 (2-2.7) | 17.9 (15.6-20.6) | 7.5 (6.5-8.5) | 28.5 (24.7-32.6) | 1.27 (-1.36-3.97) |
| CKD due to diabetes mellitus type 2 | Costa Rica | 836.8 (757.9-925.2) | 47.4 (43.2-52) | 2603.1 (2352.2-2861.9) | 46.6 (42.1-51.1) | 0.16 (-2.67-3.07) |
| CKD due to diabetes mellitus type 2 | C么te d'Ivoire | 596.7 (489-700.5) | 14.8 (12.7-16.9) | 2116.1 (1781.6-2463.8) | 18.8 (16.3-21.3) | 0.54 (-1.85-3) |
| CKD due to diabetes mellitus type 2 | Croatia | 993.2 (840-1154.3) | 15.6 (13.4-18) | 2405.1 (2082.4-2701.2) | 27.1 (23.5-30.5) | 1.64 (-0.99-4.34) |
| CKD due to diabetes mellitus type 2 | Cuba | 1488.4 (1272.5-1735.5) | 14.5 (12.4-16.9) | 5239.5 (4466.9-6051.3) | 27.2 (23.2-31.4) | 1.7 (-0.86-4.31) |
| CKD due to diabetes mellitus type 2 | Cyprus | 261.5 (223-303) | 29.7 (26.1-34) | 688 (602.1-779.8) | 31.1 (27.2-35.1) | 0.33 (-2.54-3.29) |
| CKD due to diabetes mellitus type 2 | Czechia | 1814.9 (1541.9-2105) | 13.1 (11.1-15.1) | 4867.2 (4225.8-5609.6) | 22.4 (19.4-25.9) | 1.35 (-1.2-3.96) |
| CKD due to diabetes mellitus type 2 | Democratic People's Republic of Korea | 2158 (1816.8-2530.1) | 13.4 (11.4-15.4) | 5914 (5119-6785.7) | 17.8 (15.4-20.5) | 0.71 (-1.75-3.23) |
| CKD due to diabetes mellitus type 2 | Democratic Republic of the Congo | 1448.4 (1214.3-1694.3) | 9.2 (8-10.6) | 4495.9 (3790.7-5196.3) | 12.3 (10.7-14.3) | 0.64 (-1.66-3) |
| CKD due to diabetes mellitus type 2 | Denmark | 1682.9 (1454.3-1953.1) | 19.9 (17.3-23) | 3014.6 (2639.9-3426.5) | 24.1 (21.2-27.3) | 0.64 (-2.02-3.38) |
| CKD due to diabetes mellitus type 2 | Djibouti | 12.4 (10.6-14.5) | 9.5 (8.2-10.8) | 79.9 (68.9-93.2) | 12.6 (11-14.5) | 0.59 (-1.66-2.9) |
| CKD due to diabetes mellitus type 2 | Dominica | 13.1 (11.3-15) | 22.3 (19.3-25.6) | 26.9 (23.1-31) | 31 (26.8-35.4) | 1.08 (-1.46-3.68) |
| CKD due to diabetes mellitus type 2 | Dominican Republic | 522.9 (448-616.1) | 13.6 (11.8-15.8) | 2377.3 (2028.5-2700.5) | 23.5 (20.2-26.8) | 1.55 (-0.94-4.1) |
| CKD due to diabetes mellitus type 2 | Ecuador | 976.2 (829.3-1123.3) | 18.4 (15.5-21.1) | 5929.8 (5192.6-6750) | 35.8 (31.4-40.7) | 1.93 (-0.77-4.71) |
| CKD due to diabetes mellitus type 2 | Egypt | 7386.2 (6285.2-8695.8) | 26.7 (22.9-30.8) | 30039.3 (25449.6-35153.2) | 45 (39.2-51.4) | 1.25 (-1.52-4.09) |
| CKD due to diabetes mellitus type 2 | El Salvador | 707.7 (597.8-830.5) | 23.3 (19.8-27.2) | 2379.5 (2089.8-2713.9) | 39.5 (34.6-45.1) | 2.06 (-0.6-4.79) |
| CKD due to diabetes mellitus type 2 | Equatorial Guinea | 17.5 (15.1-20.3) | 9.1 (7.9-10.4) | 82.5 (71-95.1) | 16.3 (14.2-18.5) | 1.63 (-0.74-4.07) |
| CKD due to diabetes mellitus type 2 | Eritrea | 93.2 (78.4-110.2) | 8.5 (7.4-9.8) | 302.9 (255.8-356.5) | 11 (9.5-12.8) | 0.48 (-1.76-2.78) |
| CKD due to diabetes mellitus type 2 | Estonia | 187.1 (156.7-223.8) | 9.1 (7.7-10.9) | 501.2 (428.6-585.1) | 20.4 (17.5-23.9) | 2.1 (-0.27-4.53) |
| CKD due to diabetes mellitus type 2 | Eswatini | 53 (45.3-60.8) | 18.1 (15.7-20.8) | 130.5 (111.2-148.7) | 22.3 (19.3-25.3) | 0.54 (-1.92-3.07) |
| CKD due to diabetes mellitus type 2 | Ethiopia | 1764.2 (1557.5-1995.8) | 9 (8-10) | 4767.5 (4275.2-5327.3) | 11.2 (10-12.5) | 0.38 (-1.86-2.66) |
| CKD due to diabetes mellitus type 2 | Fiji | 74.2 (62.1-87.2) | 19.4 (16.7-22.3) | 211.5 (178.7-240.3) | 25.7 (22.3-29.2) | 0.71 (-1.79-3.28) |
| CKD due to diabetes mellitus type 2 | Finland | 1162.2 (983.4-1360.2) | 15.5 (13.2-18) | 2406.9 (2097-2774.1) | 17.8 (15.6-20.4) | 0.47 (-2.17-3.18) |
| CKD due to diabetes mellitus type 2 | France | 18189 (15690.3-21061.3) | 20.8 (18-23.9) | 32305.8 (28364.9-36778) | 22.7 (20-25.8) | 0.33 (-2.4-3.14) |
| CKD due to diabetes mellitus type 2 | Gabon | 65.8 (57-76.6) | 11.4 (9.9-13) | 191.1 (166.4-219.2) | 18 (15.9-20.5) | 1.05 (-1.35-3.51) |
| CKD due to diabetes mellitus type 2 | Gambia | 46.8 (40.7-54.9) | 13.1 (11.6-14.9) | 167.3 (146.1-192.8) | 17.1 (15-19.6) | 0.63 (-1.76-3.08) |
| CKD due to diabetes mellitus type 2 | Georgia | 573 (471-680) | 8.9 (7.5-10.6) | 911 (776.3-1074.1) | 15.8 (13.6-18.5) | 1.56 (-0.76-3.94) |
| CKD due to diabetes mellitus type 2 | Germany | 31808.6 (27863.3-35858.1) | 24.3 (21.3-27.6) | 54022.4 (48788.3-60283.9) | 27.7 (25-30.6) | 0.35 (-2.39-3.18) |
| CKD due to diabetes mellitus type 2 | Ghana | 719 (613.4-845.2) | 11.4 (9.9-13) | 2727.8 (2369.1-3160.6) | 16.1 (14.1-18.4) | 0.92 (-1.48-3.37) |
| CKD due to diabetes mellitus type 2 | Greece | 4827.2 (4197.6-5529.4) | 30.4 (26.6-34.8) | 6696.2 (6009.1-7571) | 28.4 (25.3-32.2) | 0.07 (-2.76-2.99) |
| CKD due to diabetes mellitus type 2 | Greenland | 5.2 (4.4-6) | 18.1 (15.7-21) | 16.1 (14.1-18.4) | 23.6 (20.6-26.6) | 0.43 (-2.15-3.06) |
| CKD due to diabetes mellitus type 2 | Grenada | 14.1 (12.3-16.2) | 20.9 (18.1-24.1) | 42.9 (37.5-48.4) | 35.5 (31.1-40.2) | 1.46 (-1.13-4.12) |
| CKD due to diabetes mellitus type 2 | Guam | 15 (12.6-17.8) | 18.2 (15.7-20.9) | 53.7 (46.5-61.9) | 25.4 (22.2-29.1) | 1.15 (-1.39-3.75) |
| CKD due to diabetes mellitus type 2 | Guatemala | 926 (776-1084.5) | 25.2 (21.6-29.2) | 4391.2 (3794.1-5066.1) | 38.9 (33.7-44.9) | 1.42 (-1.25-4.15) |
| CKD due to diabetes mellitus type 2 | Guinea | 432 (368.6-501.4) | 12.8 (11.1-14.7) | 929.2 (809.8-1074) | 16.4 (14.4-18.9) | 0.57 (-1.78-2.99) |
| CKD due to diabetes mellitus type 2 | Guinea-Bissau | 55.4 (48-63.8) | 13.6 (11.9-15.5) | 118 (100.6-136.9) | 15.9 (13.9-18.3) | 0.37 (-1.99-2.77) |
| CKD due to diabetes mellitus type 2 | Guyana | 69.8 (59.7-80.6) | 17.4 (15-20) | 197 (167.6-228) | 28.6 (24.6-32.6) | 1.35 (-1.21-3.97) |
| CKD due to diabetes mellitus type 2 | Haiti | 566.1 (474.6-677.6) | 16.5 (14-19.4) | 1671 (1408.8-1938.5) | 21.8 (18.6-25.2) | 0.75 (-1.76-3.32) |
| CKD due to diabetes mellitus type 2 | Honduras | 521.2 (440.7-612.3) | 24.2 (20.6-28.1) | 2141 (1851.1-2494.1) | 32 (27.9-37.2) | 0.82 (-1.85-3.57) |
| CKD due to diabetes mellitus type 2 | Hungary | 1664.7 (1407-1951.9) | 11.1 (9.4-12.9) | 4530 (3949.8-5206.9) | 23.1 (20.1-26.5) | 1.92 (-0.63-4.53) |
| CKD due to diabetes mellitus type 2 | Iceland | 56.3 (49.1-65.5) | 19.3 (16.9-22.4) | 119.4 (104.3-136) | 20.3 (17.7-23.1) | 0.14 (-2.54-2.89) |
| CKD due to diabetes mellitus type 2 | India | 75873.8 (66994.2-84750.7) | 15.3 (13.7-16.9) | 222792.6 (199078.3-245038.9) | 18.2 (16.3-20) | 0.2 (-2.29-2.75) |
| CKD due to diabetes mellitus type 2 | Indonesia | 11994.9 (10583.3-13516.5) | 11.8 (10.5-13.2) | 43242.3 (38381.7-48503.1) | 17 (15.3-18.9) | 0.85 (-1.44-3.19) |
| CKD due to diabetes mellitus type 2 | Iran (Islamic Republic of) | 8652.8 (7576.7-9924.8) | 31.2 (27.8-34.8) | 32938.6 (30164.3-35878.3) | 41.4 (37.8-44.9) | 0.68 (-2.02-3.44) |
| CKD due to diabetes mellitus type 2 | Iraq | 2397.8 (2047.5-2798.3) | 30.2 (26-35) | 11405 (9809.2-13190.7) | 45.8 (40-52.6) | 1.1 (-1.69-3.96) |
| CKD due to diabetes mellitus type 2 | Ireland | 1204.2 (1074-1324.8) | 28.2 (25.1-31) | 1919.1 (1762.6-2071.7) | 23.9 (21.9-25.8) | 0.06 (-2.55-2.74) |
| CKD due to diabetes mellitus type 2 | Israel | 1479.2 (1280.8-1716.4) | 29.2 (25.4-33.8) | 4028.4 (3503.2-4629.2) | 32.2 (28.1-36.6) | 0.45 (-2.39-3.37) |
| CKD due to diabetes mellitus type 2 | Italy | 20288.6 (17952.3-22778.4) | 21.7 (19.1-24.4) | 31473.6 (28467.1-34606.4) | 20.6 (18.6-22.6) | 0.19 (-2.44-2.9) |
| CKD due to diabetes mellitus type 2 | Jamaica | 352.2 (304.4-405.9) | 20.3 (17.5-23.5) | 888.9 (781.1-1017.7) | 29 (25.4-33.2) | 1.11 (-1.47-3.77) |
| CKD due to diabetes mellitus type 2 | Japan | 52456 (47493.5-57437.4) | 30.2 (27.4-33.1) | 105931.8 (95554.3-115892.1) | 30.6 (27.9-33.2) | 0.09 (-2.7-2.96) |
| CKD due to diabetes mellitus type 2 | Jordan | 413.6 (355.7-476.9) | 30.2 (26.4-34.5) | 3764.6 (3316-4252.4) | 47.8 (42.6-53.4) | 1.23 (-1.59-4.13) |
| CKD due to diabetes mellitus type 2 | Kazakhstan | 1094.7 (914.4-1290.3) | 8.2 (6.9-9.6) | 3221.9 (2683.9-3757.8) | 16.6 (14-19.3) | 1.58 (-0.73-3.95) |
| CKD due to diabetes mellitus type 2 | Kenya | 722.2 (647.3-803.9) | 8.9 (8-9.8) | 2578.8 (2295.9-2863.7) | 11.3 (10.1-12.5) | 0.41 (-1.83-2.7) |
| CKD due to diabetes mellitus type 2 | Kiribati | 5.6 (4.7-6.7) | 13.9 (11.9-16.2) | 15.1 (12.8-17.6) | 19.2 (16.6-21.9) | 0.83 (-1.61-3.34) |
| CKD due to diabetes mellitus type 2 | Kuwait | 204.4 (174.2-238.6) | 31.6 (27.2-35.7) | 1285.5 (1107-1491.2) | 42.6 (37-48.6) | 0.88 (-1.9-3.73) |
| CKD due to diabetes mellitus type 2 | Kyrgyzstan | 245.8 (204.6-295.8) | 7.8 (6.5-9.1) | 657.3 (545.1-783.8) | 12.2 (10.2-14.4) | 0.92 (-1.3-3.19) |
| CKD due to diabetes mellitus type 2 | Lao People's Democratic Republic | 347.2 (296.5-408.5) | 16 (13.8-18.6) | 1065.8 (904.9-1229.7) | 21.9 (18.8-25.2) | 0.77 (-1.67-3.28) |
| CKD due to diabetes mellitus type 2 | Latvia | 272.1 (233-321.9) | 7.6 (6.5-9) | 547 (472.2-626.4) | 15.1 (13-17.5) | 1.61 (-0.69-3.96) |
| CKD due to diabetes mellitus type 2 | Lebanon | 616.1 (530-717.2) | 27.7 (24.3-31.8) | 2641.8 (2329.3-2967.1) | 45.5 (39.9-51.6) | 1.3 (-1.5-4.19) |
| CKD due to diabetes mellitus type 2 | Lesotho | 122.3 (104-139.6) | 14.3 (12.2-16.3) | 213 (181.7-244.3) | 19 (16.5-21.5) | 0.68 (-1.75-3.18) |
| CKD due to diabetes mellitus type 2 | Liberia | 152 (129-176.9) | 12.7 (10.9-14.7) | 364.3 (308.9-422.1) | 17.3 (15-19.6) | 0.88 (-1.51-3.34) |
| CKD due to diabetes mellitus type 2 | Libya | 520.5 (442.8-603.7) | 27.3 (23.4-31.7) | 2289.5 (1967.2-2656.3) | 43.2 (37.4-49.5) | 1.06 (-1.74-3.94) |
| CKD due to diabetes mellitus type 2 | Lithuania | 390.5 (333.1-457.2) | 8.7 (7.4-10.1) | 694.4 (603.6-791.7) | 13.2 (11.5-15.1) | 1.08 (-1.19-3.41) |
| CKD due to diabetes mellitus type 2 | Luxembourg | 141.5 (122.6-162.8) | 24.7 (21.4-28.3) | 283.9 (245.9-317.8) | 26.9 (23.2-30.3) | 0.38 (-2.41-3.26) |
| CKD due to diabetes mellitus type 2 | Madagascar | 409.8 (353-477.5) | 8.2 (7.2-9.3) | 1140.7 (983.6-1337.3) | 10.3 (8.9-11.7) | 0.41 (-1.79-2.66) |
| CKD due to diabetes mellitus type 2 | Malawi | 346 (296.8-400.4) | 9.2 (8-10.5) | 855.6 (737.7-979.2) | 11.8 (10.3-13.4) | 0.64 (-1.61-2.94) |
| CKD due to diabetes mellitus type 2 | Malaysia | 1674.8 (1430.1-1942.2) | 17.8 (15.4-20.4) | 7811 (6815.2-8935.5) | 26.3 (23-30) | 1.04 (-1.57-3.71) |
| CKD due to diabetes mellitus type 2 | Maldives | 18.1 (15.2-21.5) | 19.2 (16.6-22.3) | 104.2 (90-120.9) | 30.2 (26.2-34.9) | 1.18 (-1.41-3.83) |
| CKD due to diabetes mellitus type 2 | Mali | 485.6 (412.6-566.6) | 12 (10.3-13.7) | 1380 (1178.4-1601) | 15.5 (13.3-17.7) | 0.6 (-1.76-3) |
| CKD due to diabetes mellitus type 2 | Malta | 112.9 (98-130.5) | 25.8 (22.5-29.7) | 278.8 (244.4-315) | 27.1 (23.7-30.3) | 0.26 (-2.47-3.06) |
| CKD due to diabetes mellitus type 2 | Marshall Islands | 2.8 (2.4-3.3) | 16.1 (13.8-18.6) | 8.9 (7.6-10.4) | 23.4 (20.3-26.4) | 0.9 (-1.63-3.49) |
| CKD due to diabetes mellitus type 2 | Mauritania | 143.7 (122.8-167.6) | 13.9 (12-16.2) | 422.5 (361.8-490.3) | 19.2 (16.6-22.2) | 0.79 (-1.62-3.26) |
| CKD due to diabetes mellitus type 2 | Mauritius | 196.2 (165.2-229.8) | 25.4 (21.6-29.4) | 787.6 (681.7-900.3) | 40.1 (35-45.5) | 1.48 (-1.15-4.19) |
| CKD due to diabetes mellitus type 2 | Mexico | 13926.7 (12390.7-15710.7) | 31.3 (28-35.1) | 61209.7 (56685.9-65362.7) | 46.2 (42.9-49.4) | 1.37 (-1.37-4.18) |
| CKD due to diabetes mellitus type 2 | Micronesia (Federated States of) | 9.1 (7.7-10.7) | 17.7 (15.2-20.8) | 22.2 (18.6-26.1) | 27.2 (23.4-31.2) | 1.15 (-1.4-3.77) |
| CKD due to diabetes mellitus type 2 | Monaco | 15.4 (13.4-17.9) | 20.7 (17.8-23.9) | 22.9 (20.1-26.2) | 22.7 (19.9-25.7) | 0.32 (-2.39-3.1) |
| CKD due to diabetes mellitus type 2 | Mongolia | 135.3 (111.8-159.1) | 12 (10-14.1) | 424.7 (348.6-493) | 16.3 (13.7-18.7) | 0.7 (-1.62-3.08) |
| CKD due to diabetes mellitus type 2 | Montenegro | 116.3 (100-135.5) | 18.4 (15.9-21.5) | 285.4 (246.2-330.4) | 27.7 (24-31.7) | 1.24 (-1.28-3.83) |
| CKD due to diabetes mellitus type 2 | Morocco | 3081.6 (2675.6-3557.1) | 21.5 (18.7-24.8) | 14044.4 (12220.6-16083.1) | 39.8 (34.8-45.2) | 1.62 (-1.1-4.43) |
| CKD due to diabetes mellitus type 2 | Mozambique | 516.4 (438.8-596.9) | 8.9 (7.6-10.2) | 1222.6 (1045.7-1427.7) | 11.2 (9.8-12.9) | 0.55 (-1.67-2.82) |
| CKD due to diabetes mellitus type 2 | Myanmar | 3522.9 (3020.1-4123.3) | 14.7 (12.7-16.8) | 10655.4 (9207.2-12091.5) | 21 (18.3-23.9) | 0.97 (-1.45-3.45) |
| CKD due to diabetes mellitus type 2 | Namibia | 101.2 (87.2-119.9) | 15.3 (13.4-17.6) | 276.8 (240.1-324.5) | 19.8 (17.3-22.9) | 0.59 (-1.89-3.12) |
| CKD due to diabetes mellitus type 2 | Nauru | 0.9 (0.7-1) | 17.7 (15.3-20.6) | 1.5 (1.2-1.7) | 24 (20.8-27.7) | 0.85 (-1.66-3.42) |
| CKD due to diabetes mellitus type 2 | Nepal | 1222.4 (1042.4-1449.3) | 12.3 (10.6-14.3) | 5030.3 (4314-5817) | 20.5 (17.7-23.4) | 1.2 (-1.28-3.74) |
| CKD due to diabetes mellitus type 2 | Netherlands | 4458.4 (3939.5-5037.1) | 21.2 (18.9-23.9) | 9025.4 (7866.1-10060.5) | 24.2 (21.2-26.9) | 0.62 (-2.11-3.43) |
| CKD due to diabetes mellitus type 2 | New Zealand | 1111.6 (980.8-1284.1) | 27.2 (24.1-31.3) | 2695.4 (2380.4-3046.2) | 31.1 (27.6-35.2) | 0.55 (-2.16-3.34) |
| CKD due to diabetes mellitus type 2 | Nicaragua | 470.5 (399.5-558.8) | 28.4 (24.3-33) | 2230.6 (1915.8-2564.9) | 42.6 (36.7-48.6) | 1.31 (-1.31-4) |
| CKD due to diabetes mellitus type 2 | Niger | 339.3 (284.2-394.3) | 12.1 (10.5-13.8) | 1238.4 (1054-1443.2) | 14.7 (12.7-17) | 0.42 (-1.91-2.81) |
| CKD due to diabetes mellitus type 2 | Nigeria | 5778.8 (5144.5-6466.1) | 13 (11.6-14.5) | 15856.4 (14145.8-17503) | 17.4 (15.5-19.3) | 0.68 (-1.68-3.1) |
| CKD due to diabetes mellitus type 2 | Niue | 0.4 (0.3-0.4) | 18 (15.3-20.8) | 0.6 (0.5-0.7) | 27.1 (22.9-31.3) | 1.11 (-1.46-3.75) |
| CKD due to diabetes mellitus type 2 | North Macedonia | 262.6 (227.7-305) | 13.6 (11.9-15.7) | 958.8 (829.6-1097.2) | 26.8 (23.3-30.4) | 1.86 (-0.69-4.48) |
| CKD due to diabetes mellitus type 2 | Northern Mariana Islands | 4.9 (4.1-6) | 24.5 (21-28.6) | 19.8 (16.8-24) | 33.8 (28.9-39.8) | 0.8 (-1.88-3.56) |
| CKD due to diabetes mellitus type 2 | Norway | 1308.6 (1152.6-1491.8) | 17.9 (15.9-20.1) | 2125.9 (1911.8-2349.5) | 20.4 (18.5-22.4) | 0.73 (-1.93-3.46) |
| CKD due to diabetes mellitus type 2 | Oman | 151.4 (130.8-172.8) | 22.7 (19.7-25.9) | 865.3 (745.3-986.3) | 42.6 (37.4-48.2) | 1.61 (-1.13-4.43) |
| CKD due to diabetes mellitus type 2 | Pakistan | 7999.2 (7093.7-9017) | 13.7 (12.1-15.4) | 21609.3 (19072.7-24237.7) | 16.8 (15-18.8) | 0.74 (-1.67-3.21) |
| CKD due to diabetes mellitus type 2 | Palau | 2.2 (1.8-2.6) | 20.9 (17.6-24.6) | 8.6 (7.4-10.2) | 34.2 (29.9-39.4) | 1.42 (-1.16-4.07) |
| CKD due to diabetes mellitus type 2 | Palestine | 261.7 (224-307.1) | 29.6 (25.8-34.4) | 1189.2 (1030.3-1371.6) | 45.2 (39.9-51.3) | 0.97 (-1.77-3.79) |
| CKD due to diabetes mellitus type 2 | Panama | 386.6 (328.6-452.7) | 25.4 (21.7-29.7) | 1644.1 (1433.5-1862) | 37.4 (32.5-42.3) | 1.21 (-1.46-3.95) |
| CKD due to diabetes mellitus type 2 | Papua New Guinea | 176.2 (145-214.6) | 9 (7.7-10.6) | 645.6 (537-759.7) | 11.9 (10.1-13.9) | 0.67 (-1.57-2.96) |
| CKD due to diabetes mellitus type 2 | Paraguay | 444 (384.2-508.8) | 19.7 (17.1-22.5) | 1863.6 (1639.1-2127) | 31.2 (27.6-35.5) | 1.34 (-1.32-4.07) |
| CKD due to diabetes mellitus type 2 | Peru | 1780.9 (1513-2056.8) | 15 (12.8-17.4) | 9585 (8189.1-10937.4) | 28.9 (24.7-33.1) | 1.92 (-0.69-4.6) |
| CKD due to diabetes mellitus type 2 | Philippines | 5489.6 (4946.6-6021.4) | 18 (16.3-19.8) | 22328.1 (20227.5-24216.9) | 25.7 (23.6-27.8) | 1.09 (-1.39-3.64) |
| CKD due to diabetes mellitus type 2 | Poland | 6543.9 (5684.5-7484.2) | 14.7 (12.8-16.8) | 14663.2 (12969.3-16659.5) | 20.2 (18-22.6) | 0.17 (-2.3-2.7) |
| CKD due to diabetes mellitus type 2 | Portugal | 3175.8 (2691.8-3739.5) | 21.7 (18.5-25.3) | 6150.7 (5350.5-7184.1) | 24.2 (20.9-28) | 0.41 (-2.37-3.26) |
| CKD due to diabetes mellitus type 2 | Puerto Rico | 848 (733.9-986.4) | 23.3 (20.1-27.1) | 2271.2 (1987.3-2587) | 35.8 (31.6-40.5) | 1.37 (-1.28-4.09) |
| CKD due to diabetes mellitus type 2 | Qatar | 40.4 (34.3-47.8) | 33 (28.1-38.5) | 586.6 (497-689.3) | 53.1 (46.3-60.1) | 1.12 (-1.72-4.03) |
| CKD due to diabetes mellitus type 2 | Republic of Korea | 6234.1 (5344.1-7363) | 22 (19-25.8) | 24486.9 (21865.8-27473.5) | 25.4 (22.7-28.4) | 0.3 (-2.46-3.14) |
| CKD due to diabetes mellitus type 2 | Republic of Moldova | 269.1 (230.1-318.2) | 6.1 (5.2-7.1) | 640 (538.4-763.2) | 10.9 (9.3-13) | 1.37 (-0.87-3.65) |
| CKD due to diabetes mellitus type 2 | Romania | 3174 (2710.2-3711.7) | 11 (9.5-12.8) | 7845.3 (6892.9-8836.8) | 21.4 (18.9-23.9) | 1.62 (-0.88-4.19) |
| CKD due to diabetes mellitus type 2 | Russian Federation | 17141.6 (15089.1-19302.1) | 9.4 (8.4-10.5) | 40190.1 (36170.6-44621.8) | 17.2 (15.5-18.8) | 1.32 (-0.93-3.61) |
| CKD due to diabetes mellitus type 2 | Rwanda | 251.4 (213.7-296.7) | 9 (7.8-10.4) | 717.3 (614.6-837.3) | 11.5 (10-13.3) | 0.56 (-1.7-2.86) |
| CKD due to diabetes mellitus type 2 | Saint Kitts and Nevis | 8.2 (7-9.4) | 22.2 (19-25.3) | 26.6 (22.9-30.9) | 34.5 (30.1-39.8) | 1.35 (-1.27-4.04) |
| CKD due to diabetes mellitus type 2 | Saint Lucia | 17.8 (15.3-20.5) | 19.9 (17.3-22.9) | 76.1 (66.3-87.5) | 31 (27-35.7) | 1.26 (-1.35-3.94) |
| CKD due to diabetes mellitus type 2 | Saint Vincent and the Grenadines | 13.3 (11.5-15.4) | 18.5 (16-21.3) | 42.2 (36.8-47.9) | 28.7 (25-32.4) | 1.29 (-1.26-3.9) |
| CKD due to diabetes mellitus type 2 | Samoa | 16.8 (14.2-19.8) | 18.8 (16.1-22) | 39.6 (34-46.1) | 26.3 (22.9-30.4) | 0.84 (-1.73-3.48) |
| CKD due to diabetes mellitus type 2 | San Marino | 7.1 (6.2-8.2) | 19.3 (16.7-22) | 15.3 (13.3-17.7) | 20.5 (17.7-23.6) | 0.25 (-2.43-3.01) |
| CKD due to diabetes mellitus type 2 | Sao Tome and Principe | 10.1 (8.5-11.8) | 15.2 (13.1-17.8) | 25.5 (21.9-29.6) | 22.6 (19.8-25.8) | 1.07 (-1.34-3.55) |
| CKD due to diabetes mellitus type 2 | Saudi Arabia | 1896.9 (1657.9-2203.6) | 31.4 (27.5-35.6) | 11475.1 (9984.8-13127.3) | 52.8 (47.1-58.9) | 1.46 (-1.34-4.33) |
| CKD due to diabetes mellitus type 2 | Senegal | 434.6 (377.2-501.9) | 13.3 (11.6-15) | 1303 (1128.1-1496.8) | 16.7 (14.6-19) | 0.48 (-1.9-2.92) |
| CKD due to diabetes mellitus type 2 | Serbia | 1698.5 (1454.6-1953.8) | 14 (12.2-15.9) | 4314 (3741.4-4963.8) | 25.8 (22.5-29.4) | 1.76 (-0.86-4.46) |
| CKD due to diabetes mellitus type 2 | Seychelles | 12.5 (10.8-14.3) | 22 (19.1-25) | 39.6 (34.3-45.5) | 32.2 (28.2-36.8) | 0.98 (-1.71-3.74) |
| CKD due to diabetes mellitus type 2 | Sierra Leone | 254.9 (217.4-294.1) | 12.2 (10.4-14) | 592.8 (508.3-668.9) | 15.6 (13.6-17.6) | 0.62 (-1.71-3.01) |
| CKD due to diabetes mellitus type 2 | Singapore | 596.6 (538.3-659.8) | 27.4 (24.8-30.1) | 2775.7 (2452.8-3157.7) | 31.8 (28.4-36.1) | 0.5 (-2.28-3.37) |
| CKD due to diabetes mellitus type 2 | Slovakia | 862.4 (736.4-995.6) | 14.2 (12.2-16.4) | 2242.7 (1945-2548.9) | 23 (20-26) | 1.24 (-1.26-3.8) |
| CKD due to diabetes mellitus type 2 | Slovenia | 317.5 (272.5-369.2) | 12.9 (11.1-15) | 1000.2 (854.1-1145.1) | 22.9 (19.6-26.1) | 1.53 (-1.04-4.17) |
| CKD due to diabetes mellitus type 2 | Solomon Islands | 21.3 (17.3-25.6) | 13.9 (11.8-16.2) | 56.6 (47.8-66.6) | 15.2 (13.1-17.4) | 0.02 (-2.34-2.45) |
| CKD due to diabetes mellitus type 2 | Somalia | 208.2 (176.1-242) | 9.2 (8-10.5) | 635.2 (538.8-739.2) | 10.6 (9.2-12) | 0.33 (-1.9-2.61) |
| CKD due to diabetes mellitus type 2 | South Africa | 3492.2 (3122-3890.8) | 16.8 (15.1-18.7) | 11518.5 (10408.9-12561.4) | 24.3 (22-26.4) | 0.77 (-1.75-3.35) |
| CKD due to diabetes mellitus type 2 | South Sudan | 252.5 (218.6-292.4) | 9.8 (8.6-11.3) | 441.4 (379.1-510.7) | 11.8 (10.2-13.6) | 0.46 (-1.78-2.76) |
| CKD due to diabetes mellitus type 2 | Spain | 14143 (12447.7-16110.7) | 24.7 (21.9-28) | 23657.9 (20872.2-26718.1) | 23.6 (20.9-26.7) | 0.07 (-2.74-2.97) |
| CKD due to diabetes mellitus type 2 | Sri Lanka | 2127.9 (1848.7-2471.2) | 19.2 (16.8-22.2) | 8187.1 (7001.9-9321.5) | 28.6 (24.5-32.4) | 1.05 (-1.57-3.74) |
| CKD due to diabetes mellitus type 2 | Sudan | 2111.6 (1796.8-2461.5) | 22.5 (19.2-25.9) | 6793.2 (5910.4-7802) | 35 (30.6-39.6) | 1.12 (-1.59-3.91) |
| CKD due to diabetes mellitus type 2 | Suriname | 50.2 (42.8-58) | 18.8 (16-21.5) | 195.5 (168.4-225) | 29.7 (25.8-34.2) | 1.43 (-1.12-4.05) |
| CKD due to diabetes mellitus type 2 | Sweden | 2855.5 (2476.9-3295) | 17.5 (15.2-20.1) | 3891.5 (3355.2-4465.6) | 17.3 (15.1-19.6) | 0.1 (-2.55-2.82) |
| CKD due to diabetes mellitus type 2 | Switzerland | 2618.6 (2283.7-2985.5) | 24.2 (21.2-27.5) | 4890.8 (4216.9-5593.5) | 26.4 (22.6-30.4) | 0.3 (-2.51-3.19) |
| CKD due to diabetes mellitus type 2 | Syrian Arab Republic | 1431.3 (1207.3-1673.4) | 27.1 (23.3-31.4) | 5883.4 (5170.1-6684.4) | 42.1 (37.4-47.2) | 1.02 (-1.68-3.8) |
| CKD due to diabetes mellitus type 2 | Taiwan (Province of China) | 4573.7 (4086.6-5121.1) | 28 (25.2-31.1) | 13540.3 (12193.7-15028.1) | 31.4 (28.3-34.7) | 0.53 (-2.18-3.32) |
| CKD due to diabetes mellitus type 2 | Tajikistan | 185.8 (155.9-223.4) | 6.5 (5.5-7.7) | 711.1 (596-845.9) | 11.4 (9.8-13.1) | 1.43 (-0.77-3.67) |
| CKD due to diabetes mellitus type 2 | Thailand | 7608.2 (6472.1-8794.8) | 20.7 (17.8-23.7) | 34553.4 (30206.8-39094.9) | 31 (27.2-34.8) | 0.76 (-1.82-3.41) |
| CKD due to diabetes mellitus type 2 | Timor-Leste | 42.7 (35.9-50.9) | 14.4 (12.3-16.7) | 176.1 (152.8-205.3) | 19.8 (17.3-22.9) | 0.82 (-1.64-3.33) |
| CKD due to diabetes mellitus type 2 | Togo | 162.7 (140-192.7) | 13.1 (11.3-15.1) | 648.2 (556.5-755) | 16.7 (14.5-19.4) | 0.52 (-1.85-2.94) |
| CKD due to diabetes mellitus type 2 | Tokelau | 0.2 (0.2-0.2) | 15.2 (13.1-17.8) | 0.4 (0.3-0.4) | 24.1 (20.8-28) | 1.15 (-1.4-3.77) |
| CKD due to diabetes mellitus type 2 | Tonga | 10.6 (9.1-12.5) | 18.3 (15.8-21.2) | 21.1 (18.3-24.2) | 26 (22.6-29.7) | 0.93 (-1.64-3.56) |
| CKD due to diabetes mellitus type 2 | Trinidad and Tobago | 157.8 (136.5-181.3) | 18.3 (15.9-21.1) | 610.6 (535.8-696.9) | 30.7 (27.1-34.7) | 1.54 (-1.04-4.18) |
| CKD due to diabetes mellitus type 2 | Tunisia | 1360 (1176.1-1565.9) | 26.3 (23-30.2) | 5852.2 (5121.4-6676.8) | 42.6 (37.6-48.5) | 1.17 (-1.65-4.08) |
| CKD due to diabetes mellitus type 2 | Turkey | 7820.1 (6937.9-8868) | 22.9 (20.5-25.8) | 41374 (36218.9-47068.1) | 42.7 (37.6-48.1) | 1.52 (-1.24-4.37) |
| CKD due to diabetes mellitus type 2 | Turkmenistan | 190.3 (159.6-228.8) | 9.1 (7.8-10.8) | 643 (547.9-756.8) | 14.4 (12.4-16.8) | 1.39 (-0.84-3.67) |
| CKD due to diabetes mellitus type 2 | Tuvalu | 1 (0.8-1.2) | 14.1 (12-16.6) | 2.5 (2.1-2.9) | 23.1 (19.8-26.5) | 1.19 (-1.3-3.75) |
| CKD due to diabetes mellitus type 2 | Uganda | 543.3 (473-624.5) | 8.6 (7.5-9.8) | 1480.4 (1258.4-1690.4) | 10.4 (9-11.9) | 0.42 (-1.82-2.72) |
| CKD due to diabetes mellitus type 2 | Ukraine | 5039 (4249.3-5979.6) | 7 (6-8.4) | 7825.1 (6795.7-9052.9) | 10.3 (8.9-11.9) | 1.16 (-1.05-3.43) |
| CKD due to diabetes mellitus type 2 | United Arab Emirates | 171.7 (146.2-206.2) | 35.2 (30.7-40.6) | 3007.9 (2555.2-3635.5) | 51.8 (45.8-58.7) | 0.99 (-1.74-3.8) |
| CKD due to diabetes mellitus type 2 | United Kingdom | 20115.4 (18085.7-22686.6) | 20.9 (18.8-23.2) | 30215.2 (27274.6-33283.7) | 22.4 (20.3-24.6) | 0.02 (-2.63-2.75) |
| CKD due to diabetes mellitus type 2 | United Republic of Tanzania | 1070.3 (916.9-1234.5) | 9.9 (8.5-11.4) | 3091.7 (2686.9-3531.2) | 12.4 (10.8-14.1) | 0.44 (-1.82-2.76) |
| CKD due to diabetes mellitus type 2 | United States of America | 99641 (89218.4-110736.1) | 30.6 (27.4-33.8) | 198284.2 (180728.6-216563.9) | 33.3 (30.5-36.2) | 0.06 (-2.57-2.77) |
| CKD due to diabetes mellitus type 2 | United States Virgin Islands | 18.1 (15.4-21.2) | 20.1 (17.3-23.3) | 56.2 (48.3-64) | 31.3 (27-36) | 1.25 (-1.37-3.94) |
| CKD due to diabetes mellitus type 2 | Uruguay | 810.7 (702.2-924.7) | 20 (17.4-22.8) | 1359.1 (1196.6-1535.3) | 24.1 (21.3-27.3) | 0.59 (-2.01-3.25) |
| CKD due to diabetes mellitus type 2 | Uzbekistan | 1418.6 (1170.6-1693) | 11.7 (9.8-13.9) | 5138.4 (4246.5-6104.6) | 17.6 (14.8-20.4) | 1.1 (-1.2-3.45) |
| CKD due to diabetes mellitus type 2 | Vanuatu | 9.3 (7.8-10.9) | 13.9 (11.9-16) | 37.7 (31.5-43.3) | 20.4 (17.4-23.3) | 0.94 (-1.54-3.48) |
| CKD due to diabetes mellitus type 2 | Venezuela (Bolivarian Republic of) | 2593.7 (2230.2-3047) | 25.6 (22-29.8) | 11928.8 (10452.6-13491.5) | 38.1 (33.5-42.9) | 1.21 (-1.52-4.02) |
| CKD due to diabetes mellitus type 2 | Viet Nam | 5308.3 (4548.1-6107.9) | 13 (11.3-15) | 21256 (18212.1-24657.3) | 20.9 (18.2-24) | 1.36 (-1.13-3.91) |
| CKD due to diabetes mellitus type 2 | Yemen | 1068.3 (895-1251.9) | 21.6 (18.2-25.1) | 4383.5 (3762.6-5034.5) | 31.2 (27.1-35.8) | 0.98 (-1.7-3.73) |
| CKD due to diabetes mellitus type 2 | Zambia | 300.4 (257.1-344.9) | 10.7 (9.3-12.1) | 914.7 (779.6-1053.9) | 13.4 (11.6-15.3) | 0.5 (-1.8-2.86) |
| CKD due to diabetes mellitus type 2 | Zimbabwe | 667.7 (575.8-776.7) | 16.1 (13.9-18.5) | 1311.8 (1139.5-1503) | 18.6 (16.2-21.1) | 0.19 (-2.27-2.72) |
| CKD due to glomerulonephritis | Afghanistan | 631.2 (235-1203.6) | 5 (2.4-8.7) | 2514 (898.1-4968.9) | 6.7 (3.3-11.9) | 0.91 (-1.84-3.73) |
| CKD due to glomerulonephritis | Albania | 145.5 (50.9-283.1) | 4 (1.6-7.6) | 113.9 (62.9-188.5) | 5.2 (2.3-9.4) | 1.71 (-0.68-4.16) |
| CKD due to glomerulonephritis | Algeria | 1394 (481.6-2789.3) | 4.9 (2.2-8.9) | 3089.7 (1428-5585.2) | 7 (3.3-12.5) | 1.19 (-1.58-4.04) |
| CKD due to glomerulonephritis | American Samoa | 3.7 (1.4-7.2) | 6.2 (2.9-11.1) | 3.9 (2-6.8) | 8.5 (4.1-15.2) | 1.01 (-1.62-3.71) |
| CKD due to glomerulonephritis | Andorra | 1 (0.6-1.7) | 2.4 (1.1-4.4) | 1.7 (1.1-2.5) | 2.2 (1.1-4) | 0.1 (-2.63-2.9) |
| CKD due to glomerulonephritis | Angola | 499.9 (140.6-1014.7) | 3.1 (1.2-5.7) | 1617.8 (488.6-3179.1) | 3.7 (1.5-6.6) | 0.82 (-1.46-3.14) |
| CKD due to glomerulonephritis | Antigua and Barbuda | 3.4 (1.6-6.2) | 5.8 (2.9-10.5) | 6.7 (3.8-11.1) | 8.4 (4.2-14.9) | 1.35 (-1.26-4.03) |
| CKD due to glomerulonephritis | Argentina | 1276.8 (561-2337) | 3.8 (1.7-7) | 1612 (814.8-2728.3) | 4.4 (1.9-8) | 0.76 (-1.89-3.48) |
| CKD due to glomerulonephritis | Armenia | 138.1 (52.7-269) | 3.9 (1.5-7.5) | 134.1 (72.6-225) | 5.2 (2.4-9.4) | 1.98 (-0.36-4.38) |
| CKD due to glomerulonephritis | Australia | 434.9 (241.7-712) | 2.8 (1.4-4.9) | 804.4 (504.7-1221.3) | 3.2 (1.6-5.6) | 0.53 (-2.2-3.35) |
| CKD due to glomerulonephritis | Austria | 175.7 (100.5-267.8) | 2.6 (1.3-4.3) | 249.1 (162.7-370.5) | 2.8 (1.4-4.5) | 0.75 (-1.99-3.58) |
| CKD due to glomerulonephritis | Azerbaijan | 421.4 (153.1-821.9) | 5.2 (2-9.8) | 681.8 (310.5-1229.6) | 7.5 (3.1-14.2) | 1.98 (-0.29-4.31) |
| CKD due to glomerulonephritis | Bahamas | 13.3 (5.8-23.9) | 5.3 (2.5-9.3) | 25.7 (14-41.1) | 7.5 (3.6-13.2) | 1.27 (-1.29-3.9) |
| CKD due to glomerulonephritis | Bahrain | 24.6 (10.1-48.3) | 4.9 (2.4-8.9) | 95.4 (56.6-154.9) | 7.2 (3.9-12.6) | 1.18 (-1.68-4.13) |
| CKD due to glomerulonephritis | Bangladesh | 4257.4 (1482.7-8303.6) | 2.9 (1.3-5.1) | 5693.1 (2669.4-9858.8) | 3.7 (1.7-6.6) | 0.85 (-1.45-3.2) |
| CKD due to glomerulonephritis | Barbados | 11.5 (5.6-20.3) | 5 (2.3-9.2) | 18.1 (11-28.4) | 7.1 (3.4-12.4) | 1.31 (-1.25-3.95) |
| CKD due to glomerulonephritis | Belarus | 322.6 (140-611.2) | 3.6 (1.4-7.1) | 330.1 (184.1-535.4) | 4.6 (2-8.4) | 1.3 (-0.93-3.59) |
| CKD due to glomerulonephritis | Belgium | 249.7 (144.8-392.8) | 2.8 (1.3-4.9) | 295.6 (187.4-441.9) | 2.7 (1.4-4.7) | 0.2 (-2.55-3.04) |
| CKD due to glomerulonephritis | Belize | 12.3 (4.2-23.6) | 5.5 (2.5-9.6) | 34.6 (16.4-60.6) | 8.3 (4-14.5) | 1.35 (-1.18-3.94) |
| CKD due to glomerulonephritis | Benin | 343.3 (88.7-705.8) | 4.5 (1.7-8.6) | 929.3 (281.5-1849.5) | 5.1 (2-9.6) | 0.61 (-1.76-3.04) |
| CKD due to glomerulonephritis | Bermuda | 2.4 (1.2-3.9) | 4.4 (2-7.5) | 3.7 (2.5-5.3) | 6.4 (3.2-10.7) | 1.57 (-1.07-4.29) |
| CKD due to glomerulonephritis | Bhutan | 32.6 (11.8-65.7) | 4.1 (1.9-7.6) | 36.9 (18.9-64.5) | 5.2 (2.6-9.3) | 1.12 (-1.29-3.58) |
| CKD due to glomerulonephritis | Bolivia (Plurinational State of) | 361.6 (119-732.8) | 4.5 (2-8.4) | 641.7 (279.4-1140.2) | 5.5 (2.5-9.6) | 1.13 (-1.44-3.77) |
| CKD due to glomerulonephritis | Bosnia and Herzegovina | 127.2 (61.1-223.2) | 3.1 (1.4-5.7) | 121.9 (78.2-183.3) | 4.1 (2.1-7) | 1.82 (-0.72-4.42) |
| CKD due to glomerulonephritis | Botswana | 61.6 (22.7-111.9) | 3.9 (1.8-6.4) | 111.4 (53.2-189) | 4.7 (2.3-7.9) | 0.84 (-1.65-3.4) |
| CKD due to glomerulonephritis | Brazil | 6984.6 (5430.7-8902.8) | 4.7 (3.7-5.9) | 10483.5 (8742.3-12381.8) | 5.1 (4.1-6.2) | 0.73 (-1.81-3.35) |
| CKD due to glomerulonephritis | Brunei Darussalam | 12.5 (4.5-23.2) | 4.7 (2.2-7.9) | 21.5 (11.1-35.3) | 5.5 (2.6-9.5) | 0.43 (-2.3-3.24) |
| CKD due to glomerulonephritis | Bulgaria | 266.7 (143.9-464) | 3.6 (1.6-6.8) | 308.6 (188.8-482.9) | 5.5 (2.7-10.1) | 1.73 (-0.7-4.22) |
| CKD due to glomerulonephritis | Burkina Faso | 645.1 (183.2-1341.7) | 4.2 (1.7-8.1) | 1713 (509.6-3534.6) | 5.1 (2-9.9) | 0.78 (-1.56-3.17) |
| CKD due to glomerulonephritis | Burundi | 254.3 (82.6-530.5) | 3 (1.2-5.7) | 520.3 (179.3-1082) | 3 (1.3-5.8) | 0.33 (-1.91-2.61) |
| CKD due to glomerulonephritis | Cabo Verde | 20.3 (5.5-43.3) | 4.1 (1.4-8.2) | 26.3 (11.1-48.5) | 5.1 (2.1-9.7) | 1.1 (-1.26-3.52) |
| CKD due to glomerulonephritis | Cambodia | 579 (182.9-1159.2) | 4 (1.6-7.4) | 794.1 (339.3-1412.6) | 4.6 (2-8.1) | 0.89 (-1.49-3.33) |
| CKD due to glomerulonephritis | Cameroon | 887.8 (263.6-1900.6) | 5.7 (2.2-11.4) | 2696.7 (925.3-5607.5) | 6.8 (3-13.3) | 0.66 (-1.77-3.16) |
| CKD due to glomerulonephritis | Canada | 875.2 (460.4-1503.9) | 3.7 (1.6-6.6) | 1236.6 (724.9-1934.1) | 3.9 (1.7-7) | 0.34 (-2.23-2.97) |
| CKD due to glomerulonephritis | Central African Republic | 123.7 (36.4-255.5) | 3.1 (1.2-5.8) | 255 (84.7-502) | 3.6 (1.6-6.7) | 0.58 (-1.68-2.9) |
| CKD due to glomerulonephritis | Chad | 399.2 (118.5-820.4) | 4.1 (1.7-7.7) | 1279.7 (393-2607.2) | 4.7 (2-8.7) | 0.48 (-1.86-2.87) |
| CKD due to glomerulonephritis | Chile | 497.4 (180.7-980.3) | 3.7 (1.5-7.1) | 728.4 (393.3-1220.6) | 4.7 (2-8.8) | 1.19 (-1.54-4.01) |
| CKD due to glomerulonephritis | China | 44053.8 (35811-53621) | 3.9 (3.2-4.8) | 37892.8 (33122.3-43563.7) | 3.3 (2.7-3.9) | 0.25 (-2.21-2.76) |
| CKD due to glomerulonephritis | Colombia | 2013.9 (820.7-3842) | 5.7 (2.8-9.9) | 2960 (1615-4918.5) | 6.8 (3.2-11.9) | 0.97 (-1.67-3.69) |
| CKD due to glomerulonephritis | Comoros | 24.7 (6.9-50.3) | 3.6 (1.3-6.9) | 29.9 (10.5-57.3) | 3.8 (1.5-7.1) | 0.38 (-1.89-2.7) |
| CKD due to glomerulonephritis | Congo | 110.8 (35.3-218.7) | 3.4 (1.3-6.2) | 223.9 (86.8-418.5) | 3.8 (1.7-6.9) | 0.82 (-1.52-3.21) |
| CKD due to glomerulonephritis | Cook Islands | 0.9 (0.4-1.8) | 4.6 (2.2-8.5) | 1.1 (0.6-1.8) | 6.7 (3.5-11.9) | 1.27 (-1.36-3.97) |
| CKD due to glomerulonephritis | Costa Rica | 255.6 (109.1-478.9) | 8.2 (4.2-14) | 402.4 (228.5-647.9) | 9.3 (4.7-15.8) | 0.16 (-2.67-3.07) |
| CKD due to glomerulonephritis | C么te d'Ivoire | 907.1 (305.3-1886.5) | 5 (2.1-9.7) | 2043.3 (771.5-4158.4) | 5.8 (2.6-11) | 0.54 (-1.85-3) |
| CKD due to glomerulonephritis | Croatia | 133.6 (74.4-224.5) | 3.2 (1.5-5.9) | 155.2 (100.6-224.5) | 3.9 (2-6.6) | 1.64 (-0.99-4.34) |
| CKD due to glomerulonephritis | Cuba | 427.3 (195.2-779) | 4.3 (1.9-7.8) | 599.3 (355.8-904) | 6.1 (2.9-10.7) | 1.7 (-0.86-4.31) |
| CKD due to glomerulonephritis | Cyprus | 21 (11.2-34.3) | 2.8 (1.4-4.8) | 37.9 (23.9-55.2) | 2.8 (1.5-4.6) | 0.33 (-2.54-3.29) |
| CKD due to glomerulonephritis | Czechia | 276.4 (136.5-475) | 3.2 (1.4-5.8) | 357.5 (214.6-552.1) | 3.6 (1.7-6.4) | 1.35 (-1.2-3.96) |
| CKD due to glomerulonephritis | Democratic People's Republic of Korea | 895.6 (326.4-1709.1) | 4 (1.6-7.5) | 984.5 (522.8-1681.2) | 4.5 (1.9-8.3) | 0.71 (-1.75-3.23) |
| CKD due to glomerulonephritis | Democratic Republic of the Congo | 1801.8 (522.4-3850.3) | 3 (1.1-5.8) | 3785.2 (1274.5-7703.6) | 3.4 (1.4-6.5) | 0.64 (-1.66-3) |
| CKD due to glomerulonephritis | Denmark | 119.5 (68-194.6) | 2.6 (1.2-4.9) | 162.1 (97.6-247.7) | 2.9 (1.4-5.3) | 0.64 (-2.02-3.38) |
| CKD due to glomerulonephritis | Djibouti | 18.6 (5.9-37.5) | 3.3 (1.3-6.1) | 51.4 (19.6-97.9) | 3.8 (1.6-7) | 0.59 (-1.66-2.9) |
| CKD due to glomerulonephritis | Dominica | 5 (2-9.4) | 6.4 (2.9-11.5) | 5.2 (2.8-8.6) | 9.4 (4.2-17.4) | 1.08 (-1.46-3.68) |
| CKD due to glomerulonephritis | Dominican Republic | 352.2 (126.2-710) | 4.2 (1.9-7.9) | 670.6 (330.9-1197.1) | 6.2 (3-11.1) | 1.55 (-0.94-4.1) |
| CKD due to glomerulonephritis | Ecuador | 492.8 (177.4-966.8) | 4.3 (1.9-7.9) | 1125.8 (546.6-1949) | 6.5 (3.2-11.3) | 1.93 (-0.77-4.71) |
| CKD due to glomerulonephritis | Egypt | 3062.4 (1084.5-5906.7) | 4.7 (2.2-8.4) | 7783.7 (3409.9-14008.5) | 7.1 (3.5-12.3) | 1.25 (-1.52-4.09) |
| CKD due to glomerulonephritis | El Salvador | 348 (142.8-668.3) | 5.8 (2.8-10.3) | 702.4 (368.5-1206) | 11.1 (5.8-19) | 2.06 (-0.6-4.79) |
| CKD due to glomerulonephritis | Equatorial Guinea | 20.1 (5.5-42.6) | 3 (1.1-5.8) | 69.9 (23.3-142.9) | 4.2 (1.7-8) | 1.63 (-0.74-4.07) |
| CKD due to glomerulonephritis | Eritrea | 154.5 (45.9-316.8) | 3.1 (1.2-5.9) | 268.2 (93.6-524.7) | 3.4 (1.4-6.4) | 0.48 (-1.76-2.78) |
| CKD due to glomerulonephritis | Estonia | 52.9 (24.6-103.6) | 3.9 (1.6-8) | 56.8 (33.9-94.5) | 5.2 (2.5-10) | 2.1 (-0.27-4.53) |
| CKD due to glomerulonephritis | Eswatini | 49.6 (16-98.8) | 4.8 (2.1-8.6) | 77.4 (31.8-144.6) | 6.3 (2.8-11) | 0.54 (-1.92-3.07) |
| CKD due to glomerulonephritis | Ethiopia | 2520.5 (1513.5-3841.7) | 3.2 (2.1-4.6) | 4255.4 (2521.4-6432.1) | 3.1 (2-4.5) | 0.38 (-1.86-2.66) |
| CKD due to glomerulonephritis | Fiji | 47.7 (19.3-90.7) | 5.8 (2.7-10.6) | 65.9 (32.2-118) | 7.2 (3.5-12.9) | 0.71 (-1.79-3.28) |
| CKD due to glomerulonephritis | Finland | 82.5 (45.5-136.8) | 1.9 (0.9-3.5) | 107.2 (66.2-158.6) | 2 (1-3.6) | 0.47 (-2.17-3.18) |
| CKD due to glomerulonephritis | France | 1295.2 (688.7-2237.3) | 2.5 (1.1-4.6) | 1605.4 (994.5-2482.6) | 2.4 (1.2-4.5) | 0.33 (-2.4-3.14) |
| CKD due to glomerulonephritis | Gabon | 46.9 (15-96.8) | 3.6 (1.4-6.9) | 88.8 (34.8-174.1) | 4.5 (2-8.5) | 1.05 (-1.35-3.51) |
| CKD due to glomerulonephritis | Gambia | 62.5 (18.6-128.6) | 4.2 (1.6-8.2) | 147.3 (51.4-293.3) | 5 (2-9.3) | 0.63 (-1.76-3.08) |
| CKD due to glomerulonephritis | Georgia | 223.4 (103.3-422.1) | 4.4 (1.9-8.6) | 179.1 (96.3-317) | 5.8 (2.8-11.1) | 1.56 (-0.76-3.94) |
| CKD due to glomerulonephritis | Germany | 2055.2 (1191.8-3299) | 2.9 (1.3-5.2) | 2500.6 (1613.9-3700.5) | 2.8 (1.4-4.9) | 0.35 (-2.39-3.18) |
| CKD due to glomerulonephritis | Ghana | 748 (220-1510.3) | 3.6 (1.4-6.7) | 1861.9 (657.2-3677.5) | 4.7 (1.9-8.7) | 0.92 (-1.48-3.37) |
| CKD due to glomerulonephritis | Greece | 289.2 (178.5-448.2) | 3.1 (1.6-5.5) | 296.8 (194.7-436.1) | 2.9 (1.5-5.1) | 0.07 (-2.76-2.99) |
| CKD due to glomerulonephritis | Greenland | 1.6 (0.6-3) | 3.1 (1.3-5.7) | 1.7 (0.9-2.7) | 3.3 (1.4-5.6) | 0.43 (-2.15-3.06) |
| CKD due to glomerulonephritis | Grenada | 5.4 (2.2-10.5) | 5.7 (2.8-10.3) | 8.4 (4.8-14.2) | 8.9 (4.6-15.7) | 1.46 (-1.13-4.12) |
| CKD due to glomerulonephritis | Guam | 7.8 (3.4-15.6) | 5.2 (2.3-9.9) | 11.8 (5.9-21) | 8 (3.6-15) | 1.15 (-1.39-3.75) |
| CKD due to glomerulonephritis | Guatemala | 632.4 (213.9-1264) | 5.9 (2.8-10.7) | 1368 (649.5-2486.5) | 8.9 (4.5-15.8) | 1.42 (-1.25-4.15) |
| CKD due to glomerulonephritis | Guinea | 398.9 (116.7-869.8) | 4.3 (1.7-8.6) | 890.9 (298.7-1850.6) | 5 (2.1-9.6) | 0.57 (-1.78-2.99) |
| CKD due to glomerulonephritis | Guinea-Bissau | 71.9 (21.3-141.6) | 4.9 (1.9-8.9) | 139.2 (46.9-266.3) | 5.3 (2.2-9.5) | 0.37 (-1.99-2.77) |
| CKD due to glomerulonephritis | Guyana | 47.8 (17.4-90.9) | 5.1 (2.3-9.2) | 57.4 (28.2-99.5) | 7.6 (3.7-13.1) | 1.35 (-1.21-3.97) |
| CKD due to glomerulonephritis | Haiti | 382.5 (137.8-762.2) | 4.7 (2.1-8.7) | 754.5 (328.8-1413.9) | 5.5 (2.6-9.8) | 0.75 (-1.76-3.32) |
| CKD due to glomerulonephritis | Honduras | 336.2 (125.4-687.2) | 5.8 (2.9-10.5) | 678.4 (326.8-1239) | 6.7 (3.5-11.8) | 0.82 (-1.85-3.57) |
| CKD due to glomerulonephritis | Hungary | 256 (132.6-435.3) | 3 (1.3-5.6) | 339.5 (205.1-517.4) | 4 (2-7) | 1.92 (-0.63-4.53) |
| CKD due to glomerulonephritis | Iceland | 5.5 (2.7-9.4) | 2.3 (1-4) | 7.4 (4.3-11.4) | 2.2 (1-3.7) | 0.14 (-2.54-2.89) |
| CKD due to glomerulonephritis | India | 42126.6 (34911-50698.3) | 4.4 (3.7-5.2) | 55733.8 (47519.9-64606.2) | 4.2 (3.6-4.9) | 0.2 (-2.29-2.75) |
| CKD due to glomerulonephritis | Indonesia | 10001.4 (7597.1-12712.8) | 4.8 (3.7-6.1) | 14693.4 (11852.9-17884.6) | 5.7 (4.6-7.1) | 0.85 (-1.44-3.19) |
| CKD due to glomerulonephritis | Iran (Islamic Republic of) | 4173.3 (3245.1-5149.9) | 6.6 (5.4-7.8) | 5916.4 (5078-6888.3) | 7.6 (6.5-9.1) | 0.68 (-2.02-3.44) |
| CKD due to glomerulonephritis | Iraq | 1307 (472.9-2682) | 5.9 (2.9-10.9) | 3193.7 (1535.4-5876.3) | 8 (4.1-14.3) | 1.1 (-1.69-3.96) |
| CKD due to glomerulonephritis | Ireland | 123.1 (64.2-214.5) | 3.7 (1.8-6.6) | 160.1 (97.2-246.1) | 3.5 (1.7-5.9) | 0.06 (-2.55-2.74) |
| CKD due to glomerulonephritis | Israel | 158.8 (77.7-283.7) | 3.2 (1.6-5.7) | 330.8 (182.7-539.7) | 3.4 (1.8-5.6) | 0.45 (-2.39-3.37) |
| CKD due to glomerulonephritis | Italy | 1361.6 (1135.8-1650) | 3 (2.3-3.7) | 1407.9 (1211.9-1623.7) | 2.7 (2.2-3.5) | 0.19 (-2.44-2.9) |
| CKD due to glomerulonephritis | Jamaica | 126.9 (53.7-248.1) | 5.2 (2.5-9.6) | 176.3 (103.3-296.4) | 7 (3.7-12.2) | 1.11 (-1.47-3.77) |
| CKD due to glomerulonephritis | Japan | 4620.9 (4048.3-5186.7) | 4 (3.4-4.7) | 4871.6 (4384.5-5367.6) | 3.8 (3.2-4.4) | 0.09 (-2.7-2.96) |
| CKD due to glomerulonephritis | Jordan | 222.4 (76.9-459.3) | 5.2 (2.5-9.4) | 823.6 (414.7-1430.9) | 7.3 (3.8-12.5) | 1.23 (-1.59-4.13) |
| CKD due to glomerulonephritis | Kazakhstan | 889.3 (352.5-1794.7) | 5.1 (2.1-10.1) | 1133.8 (552-2084.4) | 6 (2.9-11.1) | 1.58 (-0.73-3.95) |
| CKD due to glomerulonephritis | Kenya | 968.8 (787.1-1162.6) | 2.8 (2.4-3.3) | 1711.7 (1439.4-2023.4) | 3.1 (2.6-3.6) | 0.41 (-1.83-2.7) |
| CKD due to glomerulonephritis | Kiribati | 5.5 (2-11.3) | 5.8 (2.6-11.1) | 9.5 (4.2-18) | 7.3 (3.4-13.4) | 0.83 (-1.61-3.34) |
| CKD due to glomerulonephritis | Kuwait | 89.6 (39.8-164.3) | 5.5 (2.8-9.5) | 257.5 (147.5-410.3) | 6.4 (3.3-11.2) | 0.88 (-1.9-3.73) |
| CKD due to glomerulonephritis | Kyrgyzstan | 338.8 (119.3-669.1) | 6.4 (2.5-12.3) | 479.5 (192.1-896.3) | 6.6 (2.7-12.1) | 0.92 (-1.3-3.19) |
| CKD due to glomerulonephritis | Lao People's Democratic Republic | 288.4 (92.2-604.4) | 5.2 (2.2-10.1) | 472.8 (195.7-909.5) | 6.1 (2.7-11.4) | 0.77 (-1.67-3.28) |
| CKD due to glomerulonephritis | Latvia | 80.9 (34.5-148.9) | 3.6 (1.3-6.9) | 69.5 (39.2-111.8) | 4.8 (2.1-8.6) | 1.61 (-0.69-3.96) |
| CKD due to glomerulonephritis | Lebanon | 171.1 (66.6-314.8) | 5.1 (2.2-8.8) | 357.7 (188.3-576.6) | 7.2 (3.5-12.1) | 1.3 (-1.5-4.19) |
| CKD due to glomerulonephritis | Lesotho | 69.4 (25.6-138.2) | 3.7 (1.7-6.7) | 97.5 (44.7-181.7) | 5.1 (2.5-9.2) | 0.68 (-1.75-3.18) |
| CKD due to glomerulonephritis | Liberia | 156 (49-308.6) | 4.2 (1.7-7.8) | 324.2 (121.4-628.4) | 5 (2.2-9) | 0.88 (-1.51-3.34) |
| CKD due to glomerulonephritis | Libya | 229.6 (89-486.2) | 4.9 (2.5-9.1) | 423.3 (246.4-727.6) | 6.9 (3.7-12.3) | 1.06 (-1.74-3.94) |
| CKD due to glomerulonephritis | Lithuania | 124.6 (54-227) | 3.9 (1.5-7.3) | 92.6 (53.1-147.5) | 4.5 (2-8.1) | 1.08 (-1.19-3.41) |
| CKD due to glomerulonephritis | Luxembourg | 9.3 (5.1-15) | 2.7 (1.2-4.7) | 16.2 (9.8-24.7) | 2.7 (1.3-4.5) | 0.38 (-2.41-3.26) |
| CKD due to glomerulonephritis | Madagascar | 568.4 (171.6-1168) | 3.2 (1.2-6.1) | 1089.5 (379.8-2180.7) | 3.1 (1.3-5.9) | 0.41 (-1.79-2.66) |
| CKD due to glomerulonephritis | Malawi | 461.8 (125.9-975.2) | 3 (1.1-5.8) | 797.7 (265.7-1608.2) | 3.4 (1.3-6.3) | 0.64 (-1.61-2.94) |
| CKD due to glomerulonephritis | Malaysia | 785.1 (286.5-1530.4) | 4 (1.7-7.3) | 1497.4 (722.8-2621) | 5.2 (2.4-9.3) | 1.04 (-1.57-3.71) |
| CKD due to glomerulonephritis | Maldives | 14.1 (4.5-28) | 4.7 (2.1-8.6) | 24.5 (13-41.3) | 5.8 (2.8-10.4) | 1.18 (-1.41-3.83) |
| CKD due to glomerulonephritis | Mali | 596.3 (166.6-1224.7) | 4.3 (1.6-8.3) | 1721.7 (517.4-3442.8) | 4.8 (1.9-8.7) | 0.6 (-1.76-3) |
| CKD due to glomerulonephritis | Malta | 10 (5.4-17.2) | 2.9 (1.4-5.3) | 13.8 (8.6-20.5) | 3.2 (1.6-5.6) | 0.26 (-2.47-3.06) |
| CKD due to glomerulonephritis | Marshall Islands | 3 (1-6.1) | 5.4 (2.3-10.1) | 4 (1.8-7.2) | 6.9 (3.2-12.5) | 0.9 (-1.63-3.49) |
| CKD due to glomerulonephritis | Mauritania | 140.1 (44.7-284.9) | 4.8 (1.9-9.2) | 302.3 (102.5-604.5) | 5.6 (2.2-10.5) | 0.79 (-1.62-3.26) |
| CKD due to glomerulonephritis | Mauritius | 65.7 (30.6-117.3) | 6.1 (3-10.6) | 112.4 (68.7-173.3) | 10 (5.1-17.3) | 1.48 (-1.15-4.19) |
| CKD due to glomerulonephritis | Mexico | 6320.9 (4979.3-7942) | 6.7 (5.5-8.1) | 12893.8 (10993.3-15050) | 10.5 (8.8-12.4) | 1.37 (-1.37-4.18) |
| CKD due to glomerulonephritis | Micronesia (Federated States of) | 7.4 (2.8-14.2) | 6.1 (2.9-10.7) | 8.8 (4.2-14.9) | 8.5 (4.1-14.3) | 1.15 (-1.4-3.77) |
| CKD due to glomerulonephritis | Monaco | 0.7 (0.4-1) | 2.5 (1.2-4.5) | 1 (0.6-1.4) | 2.5 (1.2-4.3) | 0.32 (-2.39-3.1) |
| CKD due to glomerulonephritis | Mongolia | 161.9 (59.4-326.4) | 6 (2.6-11.3) | 229.8 (101.3-417.3) | 6.7 (3-12) | 0.7 (-1.62-3.08) |
| CKD due to glomerulonephritis | Montenegro | 27 (13.2-46.3) | 4.7 (2.2-8.1) | 29.4 (17.5-44.6) | 5.1 (2.6-8.5) | 1.24 (-1.28-3.83) |
| CKD due to glomerulonephritis | Morocco | 1245.2 (461.6-2475) | 4.3 (2-7.9) | 2492.6 (1266.8-4387.9) | 7.1 (3.4-12.8) | 1.62 (-1.1-4.43) |
| CKD due to glomerulonephritis | Mozambique | 623.8 (177.7-1326) | 3 (1.2-5.9) | 1561.3 (484.4-3163.4) | 3.7 (1.5-7) | 0.55 (-1.67-2.82) |
| CKD due to glomerulonephritis | Myanmar | 2394.2 (859.8-4671.2) | 5.2 (2-9.6) | 3646.2 (1497.5-6583.2) | 6.6 (2.7-12) | 0.97 (-1.45-3.45) |
| CKD due to glomerulonephritis | Namibia | 61.8 (21.3-121.4) | 3.7 (1.6-6.5) | 101.3 (42.7-186.1) | 4.1 (1.9-7.2) | 0.59 (-1.89-3.12) |
| CKD due to glomerulonephritis | Nauru | 0.7 (0.3-1.4) | 5.8 (2.6-10.5) | 0.9 (0.4-1.8) | 7.6 (3.5-13.8) | 0.85 (-1.66-3.42) |
| CKD due to glomerulonephritis | Nepal | 1008.1 (350.9-2029.3) | 3.9 (1.8-7.3) | 1798.9 (833.6-3329.7) | 5.7 (2.8-10.4) | 1.2 (-1.28-3.74) |
| CKD due to glomerulonephritis | Netherlands | 310.7 (176-504.1) | 2.4 (1.1-4.3) | 439.1 (288-645.7) | 2.6 (1.3-4.6) | 0.62 (-2.11-3.43) |
| CKD due to glomerulonephritis | New Zealand | 107.9 (64-170.1) | 3.3 (1.9-5.5) | 197.9 (127.7-284.2) | 4.3 (2.4-6.6) | 0.55 (-2.16-3.34) |
| CKD due to glomerulonephritis | Nicaragua | 388.7 (143.3-757.1) | 8.5 (4.1-14.8) | 879.6 (455.3-1468.1) | 13.2 (6.9-21.8) | 1.31 (-1.31-4) |
| CKD due to glomerulonephritis | Niger | 594.4 (170.9-1250.7) | 4.4 (1.7-8.4) | 1650 (513.5-3379.1) | 4.4 (1.9-8.3) | 0.42 (-1.91-2.81) |
| CKD due to glomerulonephritis | Nigeria | 6131.9 (4980.1-7377.1) | 4.5 (3.7-5.3) | 14795.5 (11903.2-17958) | 4.8 (4-5.7) | 0.68 (-1.68-3.1) |
| CKD due to glomerulonephritis | Niue | 0.1 (0.1-0.3) | 5.5 (2.4-10.4) | 0.1 (0.1-0.2) | 7.6 (3.5-14.3) | 1.11 (-1.46-3.75) |
| CKD due to glomerulonephritis | North Macedonia | 66.8 (31.2-123) | 3.6 (1.6-6.8) | 93.8 (58.3-141) | 4.8 (2.5-8.5) | 1.86 (-0.69-4.48) |
| CKD due to glomerulonephritis | Northern Mariana Islands | 3 (1.4-5.2) | 6.2 (3-10.6) | 3.7 (2.1-5.8) | 8.2 (4.1-13.7) | 0.8 (-1.88-3.56) |
| CKD due to glomerulonephritis | Norway | 82 (67-100.2) | 2.1 (1.6-2.7) | 124.1 (101.9-149.6) | 2.6 (2-3.4) | 0.73 (-1.93-3.46) |
| CKD due to glomerulonephritis | Oman | 87.4 (31.3-173) | 3.8 (1.9-6.5) | 241.2 (123.9-399.8) | 5.8 (3.1-9.5) | 1.61 (-1.13-4.43) |
| CKD due to glomerulonephritis | Pakistan | 6174.1 (3468.2-9778.7) | 4.4 (2.8-6.5) | 14572.4 (8850.5-22135) | 5.6 (3.6-8.2) | 0.74 (-1.67-3.21) |
| CKD due to glomerulonephritis | Palau | 1 (0.5-1.9) | 6.7 (3.1-12) | 1.7 (1-2.7) | 10.8 (5.3-18.6) | 1.42 (-1.16-4.07) |
| CKD due to glomerulonephritis | Palestine | 164.1 (51.7-336.4) | 6 (2.7-10.7) | 392.9 (165.6-728.1) | 7.7 (3.6-13.4) | 0.97 (-1.77-3.79) |
| CKD due to glomerulonephritis | Panama | 152.4 (66.4-279.3) | 6.1 (3-10.7) | 352.4 (185.1-570.2) | 8.7 (4.4-14.4) | 1.21 (-1.46-3.95) |
| CKD due to glomerulonephritis | Papua New Guinea | 221.8 (73.8-441.8) | 4.1 (1.6-7.6) | 617.7 (218.8-1162.1) | 4.8 (2-8.4) | 0.67 (-1.57-2.96) |
| CKD due to glomerulonephritis | Paraguay | 196.4 (72-385.2) | 4.2 (2-7.6) | 418.3 (205.5-741.4) | 6.2 (3-11) | 1.34 (-1.32-4.07) |
| CKD due to glomerulonephritis | Peru | 904 (298.9-1800.3) | 3.6 (1.5-6.6) | 1862 (877.9-3359) | 5.4 (2.5-9.8) | 1.92 (-0.69-4.6) |
| CKD due to glomerulonephritis | Philippines | 3699.8 (3130.1-4366) | 4.9 (4.2-5.7) | 7810.6 (6780.7-8880.9) | 6.9 (6-7.7) | 1.09 (-1.39-3.64) |
| CKD due to glomerulonephritis | Poland | 1560.3 (1222.2-1906.2) | 4.8 (3.7-5.9) | 1244.4 (1047.3-1457.2) | 4.1 (3.2-5) | 0.17 (-2.3-2.7) |
| CKD due to glomerulonephritis | Portugal | 220.3 (117.6-352) | 2.5 (1.1-4.4) | 261.5 (165.7-383.4) | 2.5 (1.2-4.2) | 0.41 (-2.37-3.26) |
| CKD due to glomerulonephritis | Puerto Rico | 181.3 (91.9-315.1) | 5.3 (2.6-9.3) | 203.1 (134.5-297.7) | 7.9 (4-13.7) | 1.37 (-1.28-4.09) |
| CKD due to glomerulonephritis | Qatar | 23.8 (9.4-44.8) | 5.4 (2.6-9.5) | 186.9 (98.9-312.2) | 7.7 (3.8-13.1) | 1.12 (-1.72-4.03) |
| CKD due to glomerulonephritis | Republic of Korea | 1282.8 (627.6-2229.2) | 3.4 (1.6-5.9) | 1388.5 (961-1980.1) | 3 (1.5-5.1) | 0.3 (-2.46-3.14) |
| CKD due to glomerulonephritis | Republic of Moldova | 124.3 (50.8-240.9) | 2.9 (1.2-5.7) | 111.3 (66.3-183.5) | 4.1 (1.8-7.6) | 1.37 (-0.87-3.65) |
| CKD due to glomerulonephritis | Romania | 678.1 (341.3-1227.8) | 3.3 (1.5-6.4) | 672.9 (419.5-1032.8) | 4 (2-7) | 1.62 (-0.88-4.19) |
| CKD due to glomerulonephritis | Russian Federation | 8448.5 (7273-9824.3) | 6.8 (5.8-7.9) | 7938 (7003-9035.4) | 7.1 (6-8.3) | 1.32 (-0.93-3.61) |
| CKD due to glomerulonephritis | Rwanda | 331.2 (94.4-668.3) | 3.1 (1.2-5.8) | 474 (157.2-917.1) | 3.1 (1.2-5.6) | 0.56 (-1.7-2.86) |
| CKD due to glomerulonephritis | Saint Kitts and Nevis | 2.3 (1-3.9) | 5.5 (2.7-9.2) | 4.4 (2.6-6.6) | 8.1 (4.1-13.7) | 1.35 (-1.27-4.04) |
| CKD due to glomerulonephritis | Saint Lucia | 7.7 (3.1-14.8) | 5.3 (2.5-9.5) | 12.1 (7.2-19.3) | 7.8 (3.8-13.8) | 1.26 (-1.35-3.94) |
| CKD due to glomerulonephritis | Saint Vincent and the Grenadines | 5.8 (2.4-11.3) | 5.1 (2.4-9.5) | 7.7 (4.4-12.8) | 7.6 (3.8-13.8) | 1.29 (-1.26-3.9) |
| CKD due to glomerulonephritis | Samoa | 11.1 (3.7-21.8) | 5.5 (2.3-10) | 16.7 (6.7-31.7) | 7 (3.3-12.8) | 0.84 (-1.73-3.48) |
| CKD due to glomerulonephritis | San Marino | 0.5 (0.3-0.7) | 2.4 (1.1-4.2) | 0.7 (0.4-1) | 2.3 (1.1-4) | 0.25 (-2.43-3.01) |
| CKD due to glomerulonephritis | Sao Tome and Principe | 9.8 (3-19.8) | 5.7 (2.2-11.1) | 17.9 (6.7-34.6) | 7.5 (3.1-13.9) | 1.07 (-1.34-3.55) |
| CKD due to glomerulonephritis | Saudi Arabia | 964.9 (373.5-1899.5) | 5.6 (2.8-9.8) | 3053.1 (1730.4-4975.6) | 8.9 (4.8-15.4) | 1.46 (-1.34-4.33) |
| CKD due to glomerulonephritis | Senegal | 549.1 (155.7-1095.1) | 4.7 (1.7-8.8) | 995.3 (331.2-1914.3) | 5.1 (2-9.3) | 0.48 (-1.9-2.92) |
| CKD due to glomerulonephritis | Serbia | 256.5 (142.2-434.5) | 2.9 (1.4-5.2) | 300.7 (198.2-459.3) | 3.4 (1.9-5.8) | 1.76 (-0.86-4.46) |
| CKD due to glomerulonephritis | Seychelles | 3.5 (1.5-6.6) | 4.8 (2.3-8.6) | 6.6 (3.6-11) | 6.5 (3.2-11.6) | 0.98 (-1.71-3.74) |
| CKD due to glomerulonephritis | Sierra Leone | 290.4 (78.6-596.3) | 4.5 (1.7-8.6) | 594.1 (185.7-1194.3) | 5.2 (2-9.8) | 0.62 (-1.71-3.01) |
| CKD due to glomerulonephritis | Singapore | 110.4 (55.5-190.2) | 4.3 (2.1-7.6) | 223.4 (141.4-334.5) | 4.2 (2.1-7.4) | 0.5 (-2.28-3.37) |
| CKD due to glomerulonephritis | Slovakia | 177.5 (83.8-305.4) | 3.8 (1.6-6.8) | 211.1 (127.9-331.4) | 4.4 (2-7.7) | 1.24 (-1.26-3.8) |
| CKD due to glomerulonephritis | Slovenia | 46.4 (24.1-78.1) | 2.9 (1.3-5.2) | 66.4 (41.9-100.7) | 3.5 (1.7-6) | 1.53 (-1.04-4.17) |
| CKD due to glomerulonephritis | Solomon Islands | 28.4 (10.7-57.3) | 6.6 (2.9-12.2) | 49.4 (20.6-96.1) | 6.3 (2.9-11.6) | 0.02 (-2.34-2.45) |
| CKD due to glomerulonephritis | Somalia | 389.8 (110.6-767.8) | 3.2 (1.3-5.8) | 1104.1 (320-2156.3) | 3.4 (1.4-6.2) | 0.33 (-1.9-2.61) |
| CKD due to glomerulonephritis | South Africa | 1944.2 (1343.5-2683) | 4.7 (3.3-6.2) | 2762.2 (2085.1-3525.5) | 5.1 (3.9-6.6) | 0.77 (-1.75-3.35) |
| CKD due to glomerulonephritis | South Sudan | 259.4 (69-532.9) | 3 (1.1-5.7) | 482.8 (134.9-951.1) | 3.5 (1.3-6.5) | 0.46 (-1.78-2.76) |
| CKD due to glomerulonephritis | Spain | 880.3 (504.1-1379.1) | 2.6 (1.3-4.6) | 948.3 (625.5-1336) | 2 (1-3.5) | 0.07 (-2.74-2.97) |
| CKD due to glomerulonephritis | Sri Lanka | 772.1 (338.7-1479.6) | 4.5 (2.2-8.3) | 1167.1 (652.5-1961) | 5.6 (2.8-10.1) | 1.05 (-1.57-3.74) |
| CKD due to glomerulonephritis | Sudan | 1014.7 (333.6-2037.3) | 4 (1.9-7.1) | 2487.7 (965.7-4787.3) | 5.6 (2.7-9.7) | 1.12 (-1.59-3.91) |
| CKD due to glomerulonephritis | Suriname | 22.8 (9.2-43.2) | 5.7 (2.6-10.3) | 44.5 (23.2-76.5) | 8.4 (4-15.1) | 1.43 (-1.12-4.05) |
| CKD due to glomerulonephritis | Sweden | 167.1 (106-253) | 2 (1.1-3.4) | 184.5 (124.7-252.1) | 1.7 (1-2.6) | 0.1 (-2.55-2.82) |
| CKD due to glomerulonephritis | Switzerland | 163.8 (94.7-257) | 2.7 (1.3-4.7) | 225.5 (142.2-337) | 2.6 (1.3-4.4) | 0.3 (-2.51-3.19) |
| CKD due to glomerulonephritis | Syrian Arab Republic | 952.3 (330.2-2003.9) | 6 (2.7-11.3) | 925.2 (507.2-1618.8) | 6.8 (3.6-12.7) | 1.02 (-1.68-3.8) |
| CKD due to glomerulonephritis | Taiwan (Province of China) | 754.9 (404.4-1290.9) | 4.2 (2.2-7.2) | 1066.2 (669.5-1616.1) | 5.2 (2.6-9.4) | 0.53 (-2.18-3.32) |
| CKD due to glomerulonephritis | Tajikistan | 270.1 (83.3-556.1) | 3.8 (1.4-7.3) | 496.8 (187.4-963.8) | 4.4 (1.8-8.3) | 1.43 (-0.77-3.67) |
| CKD due to glomerulonephritis | Thailand | 3449.3 (1398.6-6586) | 6.2 (2.6-11.6) | 3570.5 (2277.7-5377.8) | 6.2 (3.1-10.7) | 0.76 (-1.82-3.41) |
| CKD due to glomerulonephritis | Timor-Leste | 46 (14.1-97.7) | 4.2 (1.7-8) | 77.8 (28.4-153) | 4.9 (2.1-9.1) | 0.82 (-1.64-3.33) |
| CKD due to glomerulonephritis | Togo | 239.6 (69.8-490.6) | 4.4 (1.7-8.5) | 486.1 (175.8-956.6) | 4.9 (2.1-9.1) | 0.52 (-1.85-2.94) |
| CKD due to glomerulonephritis | Tokelau | 0.1 (0-0.2) | 4.6 (2-8.8) | 0.1 (0-0.2) | 6.5 (3.1-12.1) | 1.15 (-1.4-3.77) |
| CKD due to glomerulonephritis | Tonga | 6.2 (2.1-12.6) | 5.2 (2.3-9.6) | 8.3 (3.4-15.5) | 7 (3.3-12.5) | 0.93 (-1.64-3.56) |
| CKD due to glomerulonephritis | Trinidad and Tobago | 61.5 (26.1-110.6) | 5.2 (2.3-9) | 94.6 (54.5-143.9) | 7.7 (3.7-12.9) | 1.54 (-1.04-4.18) |
| CKD due to glomerulonephritis | Tunisia | 405 (152-768.9) | 4.6 (2.1-8.3) | 703 (385.7-1176) | 6.3 (3.1-11.1) | 1.17 (-1.65-4.08) |
| CKD due to glomerulonephritis | Turkey | 2591.8 (1031.5-4887.4) | 4.3 (2-7.7) | 4690.3 (2808.4-7257.9) | 6.1 (3.3-10.4) | 1.52 (-1.24-4.37) |
| CKD due to glomerulonephritis | Turkmenistan | 270.5 (94.2-547.7) | 5.8 (2.3-11) | 429 (183.6-781.1) | 8.1 (3.5-14.7) | 1.39 (-0.84-3.67) |
| CKD due to glomerulonephritis | Tuvalu | 0.6 (0.2-1.2) | 4.8 (2-8.9) | 0.8 (0.4-1.5) | 6.6 (3.1-11.5) | 1.19 (-1.3-3.75) |
| CKD due to glomerulonephritis | Uganda | 704.2 (197.1-1507.4) | 2.4 (1-4.6) | 1584.4 (491.7-3289.3) | 2.7 (1.1-5.2) | 0.42 (-1.82-2.72) |
| CKD due to glomerulonephritis | Ukraine | 1446.2 (692.1-2612) | 3.4 (1.4-6.3) | 1364.7 (811.2-2206) | 4.5 (2.2-8) | 1.16 (-1.05-3.43) |
| CKD due to glomerulonephritis | United Arab Emirates | 113.3 (48.8-210) | 6.1 (3.2-10.5) | 828.7 (511.4-1204.7) | 8.9 (4.9-15) | 0.99 (-1.74-3.8) |
| CKD due to glomerulonephritis | United Kingdom | 1548.5 (1359.2-1766.6) | 3.1 (2.6-3.6) | 1842.9 (1649.2-2041.8) | 3.2 (2.7-3.7) | 0.02 (-2.63-2.75) |
| CKD due to glomerulonephritis | United Republic of Tanzania | 1267 (368.8-2673.9) | 3.1 (1.2-6.1) | 2544.6 (796.3-5212) | 3.4 (1.3-6.5) | 0.44 (-1.82-2.76) |
| CKD due to glomerulonephritis | United States of America | 12603.9 (10355.3-14944) | 5.6 (4.5-6.8) | 15628.8 (13599.7-17662.5) | 5.6 (4.7-6.6) | 0.06 (-2.57-2.77) |
| CKD due to glomerulonephritis | United States Virgin Islands | 5.9 (2.5-10.8) | 5.4 (2.4-9.8) | 5.5 (3.4-8.3) | 7.5 (3.6-13.4) | 1.25 (-1.37-3.94) |
| CKD due to glomerulonephritis | Uruguay | 104.9 (46-190.7) | 3.5 (1.4-6.6) | 109.3 (58.4-179.7) | 4.1 (1.6-7.5) | 0.59 (-2.01-3.25) |
| CKD due to glomerulonephritis | Uzbekistan | 1620 (651.3-3284.6) | 6.3 (2.9-12.3) | 2711.2 (1287.5-5180.2) | 7.9 (3.8-14.9) | 1.1 (-1.2-3.45) |
| CKD due to glomerulonephritis | Vanuatu | 9.1 (3-17.9) | 4.5 (2-8.3) | 20.9 (8.3-39.3) | 6 (2.7-10.5) | 0.94 (-1.54-3.48) |
| CKD due to glomerulonephritis | Venezuela (Bolivarian Republic of) | 1126.8 (497.7-2073.1) | 5.6 (2.9-9.5) | 1968.9 (1128.1-3164.3) | 7.4 (3.9-12.5) | 1.21 (-1.52-4.02) |
| CKD due to glomerulonephritis | Viet Nam | 2781.4 (1001.8-5749.8) | 3.5 (1.5-6.7) | 4273.9 (2138-7541.2) | 4.6 (2.1-8.6) | 1.36 (-1.13-3.91) |
| CKD due to glomerulonephritis | Yemen | 776.4 (229.4-1595.3) | 4.1 (1.8-7.2) | 1879.3 (679.6-3628.5) | 5.2 (2.4-9.1) | 0.98 (-1.7-3.73) |
| CKD due to glomerulonephritis | Zambia | 421.4 (121.3-930.1) | 3.4 (1.4-7) | 907.4 (311.4-1848.2) | 3.8 (1.6-7.3) | 0.5 (-1.8-2.86) |
| CKD due to glomerulonephritis | Zimbabwe | 480 (159.6-973.7) | 3.8 (1.8-6.8) | 732.8 (273.7-1411.2) | 4.2 (2-7.5) | 0.19 (-2.27-2.72) |
| CKD due to hypertension | Afghanistan | 1043.2 (876.4-1225.1) | 15.6 (13.2-18.1) | 2156.8 (1803.5-2538.7) | 22.5 (19.4-26) | 0.91 (-1.84-3.73) |
| CKD due to hypertension | Albania | 149.2 (125.7-178.3) | 7.2 (6.1-8.5) | 568 (491.1-667.7) | 12.8 (11.1-14.9) | 1.71 (-0.68-4.16) |
| CKD due to hypertension | Algeria | 1936.8 (1603.1-2313.7) | 15.9 (13.5-18.7) | 9190.7 (7859.1-10702.8) | 25.7 (22.2-29.4) | 1.19 (-1.58-4.04) |
| CKD due to hypertension | American Samoa | 3 (2.5-3.5) | 12.8 (11.2-14.6) | 9 (7.8-10.5) | 18.7 (16.2-21.3) | 1.01 (-1.62-3.71) |
| CKD due to hypertension | Andorra | 9 (7.8-10.6) | 16.2 (14.1-18.8) | 25 (22-28.6) | 16 (14-18.5) | 0.1 (-2.63-2.9) |
| CKD due to hypertension | Angola | 196.8 (166.1-235) | 5.4 (4.7-6.3) | 881.2 (745.1-1048.1) | 7.9 (6.8-9.2) | 0.82 (-1.46-3.14) |
| CKD due to hypertension | Antigua and Barbuda | 6.7 (5.8-7.9) | 12.9 (11-15.1) | 22.5 (19.5-26.2) | 20.5 (17.8-23.8) | 1.35 (-1.26-4.03) |
| CKD due to hypertension | Argentina | 4523.5 (3908-5212.7) | 14.3 (12.4-16.4) | 10594.7 (9209-12108.8) | 18.4 (15.9-20.9) | 0.76 (-1.89-3.48) |
| CKD due to hypertension | Armenia | 139.1 (113.4-169.5) | 5 (4.2-6) | 457.8 (394.4-535.6) | 10.6 (9.1-12.4) | 1.98 (-0.36-4.38) |
| CKD due to hypertension | Australia | 3624.4 (3206.4-4027.8) | 17.9 (15.9-19.8) | 9940 (8634.9-11191.5) | 20.8 (18.1-23.4) | 0.53 (-2.2-3.35) |
| CKD due to hypertension | Austria | 1961.2 (1681.7-2292) | 15.5 (13.4-18) | 3760.4 (3290-4285) | 19.4 (17.1-22.3) | 0.75 (-1.99-3.58) |
| CKD due to hypertension | Azerbaijan | 290.7 (236.5-354.2) | 5.5 (4.6-6.6) | 1193.5 (992.7-1429.4) | 11 (9.4-12.9) | 1.98 (-0.29-4.31) |
| CKD due to hypertension | Bahamas | 18.2 (15.4-21.3) | 11.4 (9.7-13.1) | 72.3 (62.4-83.2) | 17.2 (15-19.6) | 1.27 (-1.29-3.9) |
| CKD due to hypertension | Bahrain | 32.3 (27.7-38.4) | 18.6 (16.2-21.5) | 286.3 (242.6-343) | 29.2 (25.7-33.2) | 1.18 (-1.68-4.13) |
| CKD due to hypertension | Bangladesh | 2611.9 (2208.1-3037) | 5.7 (4.9-6.5) | 11196.1 (9571.6-12838.4) | 8.2 (7.1-9.3) | 0.85 (-1.45-3.2) |
| CKD due to hypertension | Barbados | 32 (27.7-37.5) | 11.3 (9.9-13.2) | 93 (80.7-106.7) | 18.3 (15.9-21) | 1.31 (-1.25-3.95) |
| CKD due to hypertension | Belarus | 601.9 (506.7-719.1) | 4.8 (4-5.7) | 1238.7 (1057.5-1444.1) | 8 (6.8-9.4) | 1.3 (-0.93-3.59) |
| CKD due to hypertension | Belgium | 2716.9 (2338.6-3163.3) | 16.7 (14.4-19.3) | 4383.4 (3861.5-5001.1) | 17.5 (15.4-19.8) | 0.2 (-2.55-3.04) |
| CKD due to hypertension | Belize | 10.7 (9-12.7) | 11.2 (9.5-13.2) | 56.9 (48.2-67.8) | 18.1 (15.6-21.1) | 1.35 (-1.18-3.94) |
| CKD due to hypertension | Benin | 160.8 (137.9-188) | 8 (7-9.2) | 551.4 (464.5-653.7) | 10.6 (9.2-12.3) | 0.61 (-1.76-3.04) |
| CKD due to hypertension | Bermuda | 7.3 (6.3-8.4) | 11.6 (10-13.4) | 25.8 (22.2-29.1) | 19.7 (16.9-22.3) | 1.57 (-1.07-4.29) |
| CKD due to hypertension | Bhutan | 18.4 (15-22.9) | 7.2 (6.1-8.6) | 68.2 (57.8-81.8) | 11.1 (9.5-13.1) | 1.12 (-1.29-3.58) |
| CKD due to hypertension | Bolivia (Plurinational State of) | 342.8 (294.1-395.2) | 11 (9.6-12.6) | 1498.4 (1317.4-1712.9) | 16.8 (14.8-19.1) | 1.13 (-1.44-3.77) |
| CKD due to hypertension | Bosnia and Herzegovina | 293.3 (247.8-345.9) | 7.2 (6.2-8.3) | 895.5 (775.6-1031.1) | 14.3 (12.4-16.3) | 1.82 (-0.72-4.42) |
| CKD due to hypertension | Botswana | 52.1 (44.3-62) | 9.6 (8.3-11.1) | 197.9 (168.6-232.9) | 13.6 (11.9-15.9) | 0.84 (-1.65-3.4) |
| CKD due to hypertension | Brazil | 10393.2 (9347.1-11454.7) | 11.6 (10.5-12.8) | 40334.4 (37043.4-43438) | 16.1 (14.8-17.3) | 0.73 (-1.81-3.35) |
| CKD due to hypertension | Brunei Darussalam | 19 (16.5-21.9) | 20.2 (17.7-23.2) | 70.1 (59.7-82.1) | 22.2 (19.3-25.2) | 0.43 (-2.3-3.24) |
| CKD due to hypertension | Bulgaria | 951 (813.5-1114.2) | 7.7 (6.6-9) | 1977.1 (1703.1-2287.8) | 14.1 (12.1-16.3) | 1.73 (-0.7-4.22) |
| CKD due to hypertension | Burkina Faso | 317.5 (271.5-378.5) | 7.5 (6.5-8.8) | 916.1 (784.1-1074.7) | 9.9 (8.6-11.6) | 0.78 (-1.56-3.17) |
| CKD due to hypertension | Burundi | 129 (110.2-151) | 5.7 (5-6.6) | 318.6 (273.9-372.7) | 6.9 (6.1-7.9) | 0.33 (-1.91-2.61) |
| CKD due to hypertension | Cabo Verde | 15.9 (13.7-18.4) | 6.7 (5.9-7.8) | 46.4 (39.7-54.5) | 10.7 (9.3-12.4) | 1.1 (-1.26-3.52) |
| CKD due to hypertension | Cambodia | 320 (267.7-380.7) | 7.1 (6.1-8.2) | 1318.2 (1135.9-1556.6) | 10.7 (9.3-12.4) | 0.89 (-1.49-3.33) |
| CKD due to hypertension | Cameroon | 456.5 (384.2-547.4) | 10.3 (8.9-12) | 1802.4 (1499.3-2159.4) | 13.9 (11.9-16) | 0.66 (-1.77-3.16) |
| CKD due to hypertension | Canada | 5973.2 (5256.5-6674.5) | 18 (15.8-20.2) | 13647.3 (11834-15628) | 18 (15.6-20.6) | 0.34 (-2.23-2.97) |
| CKD due to hypertension | Central African Republic | 58.2 (48.1-70) | 5.4 (4.7-6.3) | 149 (122.7-180) | 7 (6.1-8.1) | 0.58 (-1.68-2.9) |
| CKD due to hypertension | Chad | 214.7 (181.9-251.2) | 7.6 (6.5-8.7) | 548.9 (449.9-654.3) | 9.5 (8.1-11) | 0.48 (-1.86-2.87) |
| CKD due to hypertension | Chile | 1379.5 (1193.4-1592.5) | 14.4 (12.5-16.5) | 5550.2 (4884.8-6294.9) | 21.3 (18.8-24.2) | 1.19 (-1.54-4.01) |
| CKD due to hypertension | China | 72676.8 (64792.7-80603.3) | 9.2 (8.4-10.2) | 222288.1 (203329.3-239290) | 10.8 (10-11.7) | 0.25 (-2.21-2.76) |
| CKD due to hypertension | Colombia | 2441.5 (2055-2916.9) | 13.6 (11.6-16.1) | 10753.8 (9282.5-12413.3) | 19.4 (16.7-22.4) | 0.97 (-1.67-3.69) |
| CKD due to hypertension | Comoros | 11.7 (9.9-13.9) | 6.2 (5.4-7.2) | 36.3 (31.2-41.8) | 7.7 (6.7-8.8) | 0.38 (-1.89-2.7) |
| CKD due to hypertension | Congo | 62 (52.1-73.9) | 6 (5.2-7) | 233.1 (196.8-275.9) | 8.9 (7.7-10.2) | 0.82 (-1.52-3.21) |
| CKD due to hypertension | Cook Islands | 1.3 (1.1-1.5) | 10.7 (9.2-12.3) | 4.4 (3.8-5) | 17.3 (14.9-19.7) | 1.27 (-1.36-3.97) |
| CKD due to hypertension | Costa Rica | 458.4 (404.4-521.8) | 25.9 (23-29.3) | 1478.9 (1308.3-1680.4) | 26.7 (23.6-30.3) | 0.16 (-2.67-3.07) |
| CKD due to hypertension | C么te d'Ivoire | 355 (292.8-427.6) | 9 (7.8-10.4) | 1297.8 (1084.4-1542) | 11.7 (10-13.4) | 0.54 (-1.85-3) |
| CKD due to hypertension | Croatia | 560.6 (475-653.4) | 9.2 (7.9-10.5) | 1495.8 (1308.4-1701.1) | 16.7 (14.5-18.9) | 1.64 (-0.99-4.34) |
| CKD due to hypertension | Cuba | 872.8 (750.8-1016.8) | 8.5 (7.3-10) | 3114.1 (2681.6-3589.5) | 16.2 (14.1-18.6) | 1.7 (-0.86-4.31) |
| CKD due to hypertension | Cyprus | 161.2 (135.8-189.2) | 19.5 (17-22.1) | 466.8 (411.5-531) | 21.1 (18.5-23.9) | 0.33 (-2.54-3.29) |
| CKD due to hypertension | Czechia | 1071.7 (905.1-1235.4) | 7.8 (6.7-9) | 3079.1 (2639.1-3587.4) | 14 (12.1-16.2) | 1.35 (-1.2-3.96) |
| CKD due to hypertension | Democratic People's Republic of Korea | 1236.6 (1037.5-1459.9) | 8.2 (7-9.7) | 3625.9 (3150.8-4259.3) | 11.3 (9.8-13.2) | 0.71 (-1.75-3.23) |
| CKD due to hypertension | Democratic Republic of the Congo | 828.3 (698.7-989.7) | 5.7 (4.9-6.5) | 2684.6 (2232.5-3180.7) | 7.8 (6.6-9) | 0.64 (-1.66-3) |
| CKD due to hypertension | Denmark | 1245.4 (1055-1461) | 14.4 (12.3-16.7) | 2214.5 (1917.9-2523.6) | 17.2 (15-19.5) | 0.64 (-2.02-3.38) |
| CKD due to hypertension | Djibouti | 7.7 (6.4-9.2) | 6.1 (5.3-7) | 48.2 (40.5-56.3) | 8.1 (7-9.2) | 0.59 (-1.66-2.9) |
| CKD due to hypertension | Dominica | 7.8 (6.7-9) | 13.1 (11.4-15.1) | 15.8 (13.7-18.1) | 18.8 (16.3-21.4) | 1.08 (-1.46-3.68) |
| CKD due to hypertension | Dominican Republic | 311.7 (261.1-367.6) | 8.2 (7-9.6) | 1457.1 (1258.5-1681) | 14.5 (12.5-16.7) | 1.55 (-0.94-4.1) |
| CKD due to hypertension | Ecuador | 583.3 (497.3-684.2) | 11.2 (9.6-12.9) | 3576 (3089.3-4072.9) | 21.8 (18.9-24.7) | 1.93 (-0.77-4.71) |
| CKD due to hypertension | Egypt | 4247.2 (3530.1-5020.5) | 16.3 (13.9-18.7) | 17029.4 (14388.2-19745.8) | 27 (23.5-30.4) | 1.25 (-1.52-4.09) |
| CKD due to hypertension | El Salvador | 410.4 (342.9-493.8) | 13.5 (11.3-16) | 1449.3 (1258.7-1675.1) | 23.6 (20.4-27.3) | 2.06 (-0.6-4.79) |
| CKD due to hypertension | Equatorial Guinea | 10 (8.4-12.1) | 5.6 (4.8-6.5) | 50.2 (42.5-59.3) | 10 (8.7-11.5) | 1.63 (-0.74-4.07) |
| CKD due to hypertension | Eritrea | 56.7 (46.6-70.1) | 5.4 (4.7-6.2) | 186.7 (154.9-224) | 7 (6-8.2) | 0.48 (-1.76-2.78) |
| CKD due to hypertension | Estonia | 113.9 (95.6-133.6) | 5.7 (4.8-6.7) | 320.3 (278.8-366.9) | 12.7 (11-14.7) | 2.1 (-0.27-4.53) |
| CKD due to hypertension | Eswatini | 32.5 (27.3-39) | 11.3 (9.8-13.2) | 80.8 (67.8-96.2) | 14 (12.2-16.4) | 0.54 (-1.92-3.07) |
| CKD due to hypertension | Ethiopia | 1045.8 (930.3-1184.9) | 5.7 (5.1-6.3) | 3013.5 (2717.4-3352.6) | 7.2 (6.5-8) | 0.38 (-1.86-2.66) |
| CKD due to hypertension | Fiji | 43 (36.1-52) | 11.6 (10.1-13.5) | 121.7 (103.7-140.8) | 15.7 (13.5-18) | 0.71 (-1.79-3.28) |
| CKD due to hypertension | Finland | 893.3 (752.5-1039.5) | 12 (10.1-13.9) | 1969 (1716.6-2263.1) | 13.9 (12.1-15.8) | 0.47 (-2.17-3.18) |
| CKD due to hypertension | France | 13108.3 (11077.3-15166.5) | 14.5 (12.5-16.7) | 24693.8 (21698.4-28025.7) | 16.2 (14.3-18.4) | 0.33 (-2.4-3.14) |
| CKD due to hypertension | Gabon | 38.9 (32.9-45.7) | 7 (6-8.2) | 112.8 (94.7-134.6) | 11.1 (9.6-13) | 1.05 (-1.35-3.51) |
| CKD due to hypertension | Gambia | 28.1 (23.5-33.3) | 8 (6.9-9.3) | 104.7 (88-123.2) | 10.6 (9.1-12.2) | 0.63 (-1.76-3.08) |
| CKD due to hypertension | Georgia | 341.1 (281.7-400.6) | 5.5 (4.6-6.5) | 555.1 (469-645.6) | 9.7 (8.2-11.4) | 1.56 (-0.76-3.94) |
| CKD due to hypertension | Germany | 21908.1 (18812.4-25260.1) | 16.4 (14.2-18.7) | 39082.9 (34220.5-44152.2) | 18.7 (16.6-21) | 0.35 (-2.39-3.18) |
| CKD due to hypertension | Ghana | 428.5 (356.9-516.4) | 7 (6-8.2) | 1676 (1404.9-1991.2) | 10.1 (8.7-11.8) | 0.92 (-1.48-3.37) |
| CKD due to hypertension | Greece | 3098.6 (2659.1-3547.4) | 19.6 (16.9-22.3) | 4877.6 (4277.3-5570.8) | 19 (16.6-21.5) | 0.07 (-2.76-2.99) |
| CKD due to hypertension | Greenland | 3.7 (3.1-4.3) | 13.9 (12-16.2) | 10 (8.8-11.6) | 16.3 (14.2-18.8) | 0.43 (-2.15-3.06) |
| CKD due to hypertension | Grenada | 8.6 (7.4-9.9) | 12.2 (10.5-14.1) | 24.7 (21-28.4) | 21.1 (18.1-24.3) | 1.46 (-1.13-4.12) |
| CKD due to hypertension | Guam | 8.4 (7.1-10) | 10.8 (9.3-12.3) | 32.4 (28.2-37.8) | 15.4 (13.3-17.9) | 1.15 (-1.39-3.75) |
| CKD due to hypertension | Guatemala | 523.4 (439.1-618.1) | 14.7 (12.6-17.1) | 2613.9 (2258.6-3075.6) | 23.2 (20.2-27.2) | 1.42 (-1.25-4.15) |
| CKD due to hypertension | Guinea | 259.7 (220.8-304.7) | 7.9 (6.8-9) | 582.9 (488.8-681.9) | 10.3 (8.8-11.8) | 0.57 (-1.78-2.99) |
| CKD due to hypertension | Guinea-Bissau | 33.2 (27.6-40) | 8.3 (7.2-9.6) | 74.5 (60.8-91.2) | 10 (8.5-11.5) | 0.37 (-1.99-2.77) |
| CKD due to hypertension | Guyana | 40.9 (34.3-49) | 10.3 (8.8-12) | 114.1 (96.5-133.2) | 17.2 (14.7-19.8) | 1.35 (-1.21-3.97) |
| CKD due to hypertension | Haiti | 323.8 (273.3-386.8) | 9.9 (8.5-11.6) | 991.8 (844-1169.7) | 13.4 (11.4-15.5) | 0.75 (-1.76-3.32) |
| CKD due to hypertension | Honduras | 293.6 (250.1-352.5) | 13.9 (11.9-16.3) | 1249.8 (1081.1-1477.3) | 19 (16.6-22.2) | 0.82 (-1.85-3.57) |
| CKD due to hypertension | Hungary | 983.9 (829.4-1147.4) | 6.7 (5.7-7.8) | 2849.5 (2473.9-3319.1) | 14.4 (12.4-16.9) | 1.92 (-0.63-4.53) |
| CKD due to hypertension | Iceland | 40.3 (34.7-45.9) | 13.5 (11.7-15.4) | 87.6 (76.8-99.7) | 14.4 (12.6-16.4) | 0.14 (-2.54-2.89) |
| CKD due to hypertension | India | 43263.8 (38860.7-48106.2) | 9.1 (8.3-10.1) | 133332.8 (121484.9-146138.2) | 11.2 (10.3-12.3) | 0.2 (-2.29-2.75) |
| CKD due to hypertension | Indonesia | 7234.3 (6441.7-8057.1) | 7.4 (6.6-8.1) | 25694.3 (23048.8-28481.9) | 10.8 (9.8-11.9) | 0.85 (-1.44-3.19) |
| CKD due to hypertension | Iran (Islamic Republic of) | 4852 (4291.4-5455.2) | 18.5 (16.8-20.4) | 19551.4 (18073.8-21074.3) | 25.1 (23.3-26.9) | 0.68 (-2.02-3.44) |
| CKD due to hypertension | Iraq | 1397.9 (1199.9-1629.6) | 17.6 (15.1-20.4) | 6581 (5687.3-7623.4) | 27 (23.6-30.6) | 1.1 (-1.69-3.96) |
| CKD due to hypertension | Ireland | 773 (685.9-867.9) | 18.2 (16.2-20.5) | 1365.3 (1236.9-1513.5) | 16.8 (15.2-18.6) | 0.06 (-2.55-2.74) |
| CKD due to hypertension | Israel | 954.8 (822.6-1107.3) | 19.1 (16.5-21.6) | 2682.2 (2343.6-3085.4) | 21.1 (18.4-24.2) | 0.45 (-2.39-3.37) |
| CKD due to hypertension | Italy | 14074.8 (12606.7-15691.4) | 15.2 (13.7-16.8) | 25858.2 (23511.6-28170.4) | 15.7 (14.4-17) | 0.19 (-2.44-2.9) |
| CKD due to hypertension | Jamaica | 212 (185-247.6) | 11.9 (10.4-13.8) | 542.6 (476-622.4) | 17.5 (15.3-20.1) | 1.11 (-1.47-3.77) |
| CKD due to hypertension | Japan | 31908.9 (29333.1-34679) | 18.8 (17.3-20.3) | 77690.2 (70692.7-84111.3) | 20.1 (18.5-21.6) | 0.09 (-2.7-2.96) |
| CKD due to hypertension | Jordan | 232.9 (195.1-276.5) | 17.6 (15.2-20.3) | 2153.3 (1857.3-2489.1) | 28.1 (24.8-31.7) | 1.23 (-1.59-4.13) |
| CKD due to hypertension | Kazakhstan | 661.3 (541.5-816.6) | 5 (4.2-6.1) | 1878.1 (1574-2245.5) | 10.1 (8.6-12.1) | 1.58 (-0.73-3.95) |
| CKD due to hypertension | Kenya | 459.5 (418.3-503.2) | 5.8 (5.3-6.4) | 1630.2 (1475.2-1801.3) | 7.4 (6.7-8.2) | 0.41 (-1.83-2.7) |
| CKD due to hypertension | Kiribati | 3.3 (2.7-4.2) | 8.5 (7.2-10.1) | 9.2 (7.5-11.4) | 12.1 (10.5-14.3) | 0.83 (-1.61-3.34) |
| CKD due to hypertension | Kuwait | 116.8 (97.2-139.5) | 18.3 (15.9-20.8) | 749.9 (632.4-885) | 25.5 (22.5-29.1) | 0.88 (-1.9-3.73) |
| CKD due to hypertension | Kyrgyzstan | 154.7 (124.3-199.7) | 4.8 (3.9-5.9) | 407.3 (334.2-501.5) | 7.7 (6.5-9.2) | 0.92 (-1.3-3.19) |
| CKD due to hypertension | Lao People's Democratic Republic | 203 (166.8-245) | 9.7 (8.2-11.4) | 632.9 (529.7-761.7) | 13.4 (11.4-15.6) | 0.77 (-1.67-3.28) |
| CKD due to hypertension | Latvia | 166.1 (140.6-194.8) | 4.8 (4-5.6) | 349.1 (297.8-409.9) | 9.4 (8-11) | 1.61 (-0.69-3.96) |
| CKD due to hypertension | Lebanon | 349.1 (298.7-410.5) | 16.4 (14.1-18.9) | 1598.3 (1410.1-1835.1) | 26.9 (23.6-31.2) | 1.3 (-1.5-4.19) |
| CKD due to hypertension | Lesotho | 75.7 (63.7-88.5) | 9.1 (7.8-10.6) | 130.9 (109-153.3) | 12.1 (10.3-13.9) | 0.68 (-1.75-3.18) |
| CKD due to hypertension | Liberia | 90 (77.5-105.2) | 7.8 (6.8-8.8) | 224.6 (186.7-267.8) | 10.7 (9.2-12.4) | 0.88 (-1.51-3.34) |
| CKD due to hypertension | Libya | 303.9 (257.9-352.4) | 16.2 (14-18.7) | 1345.8 (1159.1-1572.6) | 25.9 (22.6-29.9) | 1.06 (-1.74-3.94) |
| CKD due to hypertension | Lithuania | 239.6 (202.6-280.7) | 5.4 (4.6-6.3) | 450.4 (385.7-514.4) | 8.4 (7.2-9.7) | 1.08 (-1.19-3.41) |
| CKD due to hypertension | Luxembourg | 96.6 (84.4-112.5) | 16.9 (14.7-19.3) | 201.1 (177-225.9) | 18.6 (16.2-20.9) | 0.38 (-2.41-3.26) |
| CKD due to hypertension | Madagascar | 254.7 (215.4-299.6) | 5.3 (4.6-6.1) | 700 (585.7-840.5) | 6.6 (5.7-7.5) | 0.41 (-1.79-2.66) |
| CKD due to hypertension | Malawi | 216.2 (183.7-254.5) | 6 (5.2-6.9) | 543.4 (466.7-626) | 7.7 (6.7-8.8) | 0.64 (-1.61-2.94) |
| CKD due to hypertension | Malaysia | 972.2 (829.3-1171) | 10.4 (9-12.3) | 4517.3 (3877.6-5362.9) | 15.6 (13.5-18.4) | 1.04 (-1.57-3.71) |
| CKD due to hypertension | Maldives | 10.2 (8.5-12.3) | 11.4 (9.7-13.2) | 61 (51.9-70.6) | 17.8 (15.4-20.3) | 1.18 (-1.41-3.83) |
| CKD due to hypertension | Mali | 281.6 (237.5-330.1) | 7.3 (6.3-8.4) | 845.4 (711-991.3) | 9.6 (8.4-11.1) | 0.6 (-1.76-3) |
| CKD due to hypertension | Malta | 76.2 (65.9-87.3) | 17.8 (15.5-20.3) | 196.1 (172-224) | 18.5 (16.3-20.9) | 0.26 (-2.47-3.06) |
| CKD due to hypertension | Marshall Islands | 1.7 (1.4-2) | 9.6 (8.2-11.1) | 5.2 (4.3-6.2) | 14.6 (12.4-16.6) | 0.9 (-1.63-3.49) |
| CKD due to hypertension | Mauritania | 86.8 (73.4-102.1) | 8.6 (7.4-9.8) | 260.2 (220.2-303.8) | 11.9 (10.2-13.7) | 0.79 (-1.62-3.26) |
| CKD due to hypertension | Mauritius | 111 (93.2-131.5) | 14.6 (12.3-17.1) | 441.4 (379-509.9) | 23.2 (19.9-26.7) | 1.48 (-1.15-4.19) |
| CKD due to hypertension | Mexico | 7780.6 (7005.7-8639.2) | 17.7 (16-19.5) | 34973.2 (32860.3-37223.8) | 26.8 (25.1-28.5) | 1.37 (-1.37-4.18) |
| CKD due to hypertension | Micronesia (Federated States of) | 5.4 (4.5-6.5) | 10.7 (9.2-12.6) | 13 (10.8-15.7) | 16.9 (14.5-19.8) | 1.15 (-1.4-3.77) |
| CKD due to hypertension | Monaco | 11.6 (10-13.5) | 14.8 (12.8-16.9) | 17.1 (14.7-19.6) | 16 (13.8-18.1) | 0.32 (-2.39-3.1) |
| CKD due to hypertension | Mongolia | 83.4 (66.6-103) | 7.2 (6.1-8.5) | 255.9 (212.2-310.7) | 10.2 (8.7-12) | 0.7 (-1.62-3.08) |
| CKD due to hypertension | Montenegro | 68.1 (59.1-80) | 11 (9.6-12.8) | 170.1 (145.2-200.1) | 17 (14.6-20) | 1.24 (-1.28-3.83) |
| CKD due to hypertension | Morocco | 1793.9 (1532-2084.9) | 12.9 (11.1-14.9) | 8165.6 (7010-9362.1) | 23.9 (20.6-27.1) | 1.62 (-1.1-4.43) |
| CKD due to hypertension | Mozambique | 318.7 (267.8-375.5) | 5.8 (4.9-6.6) | 762.1 (640.8-890.8) | 7.2 (6.2-8.3) | 0.55 (-1.67-2.82) |
| CKD due to hypertension | Myanmar | 2067.3 (1710.9-2461) | 8.9 (7.6-10.5) | 6290.3 (5397-7302.4) | 12.9 (11.1-14.9) | 0.97 (-1.45-3.45) |
| CKD due to hypertension | Namibia | 60.8 (51.5-70.7) | 9.6 (8.4-11) | 169.2 (144-196.1) | 12.5 (10.8-14.3) | 0.59 (-1.89-3.12) |
| CKD due to hypertension | Nauru | 0.5 (0.4-0.6) | 10.8 (9.3-12.4) | 0.9 (0.7-1.1) | 14.8 (12.5-17.2) | 0.85 (-1.66-3.42) |
| CKD due to hypertension | Nepal | 694.5 (589-832.6) | 7.3 (6.3-8.5) | 2967.9 (2550.3-3502.4) | 12.4 (10.7-14.4) | 1.2 (-1.28-3.74) |
| CKD due to hypertension | Netherlands | 3211.2 (2814.4-3649.8) | 15.2 (13.4-17.2) | 6636.9 (5781.6-7533.4) | 17.4 (15.4-19.8) | 0.62 (-2.11-3.43) |
| CKD due to hypertension | New Zealand | 733.2 (638.5-849.5) | 18 (15.8-20.8) | 1814.8 (1613.8-2035.7) | 20.7 (18.4-23.2) | 0.55 (-2.16-3.34) |
| CKD due to hypertension | Nicaragua | 268.3 (226.8-321.7) | 16.1 (13.8-18.8) | 1318.8 (1137.9-1540.5) | 25.1 (21.8-28.8) | 1.31 (-1.31-4) |
| CKD due to hypertension | Niger | 199.9 (164.4-239.7) | 7.4 (6.4-8.5) | 760.7 (619-907.5) | 9.2 (7.9-10.6) | 0.42 (-1.91-2.81) |
| CKD due to hypertension | Nigeria | 3483.2 (3153-3840.2) | 8.1 (7.3-8.9) | 9790.6 (8878.5-10683.3) | 10.9 (9.9-12.1) | 0.68 (-1.68-3.1) |
| CKD due to hypertension | Niue | 0.2 (0.2-0.3) | 10.6 (9-12.4) | 0.4 (0.3-0.4) | 16.4 (14-18.8) | 1.11 (-1.46-3.75) |
| CKD due to hypertension | North Macedonia | 153.6 (129.9-177.8) | 8.2 (7-9.5) | 562.3 (482.6-656.1) | 16.4 (14.1-19.1) | 1.86 (-0.69-4.48) |
| CKD due to hypertension | Northern Mariana Islands | 2.9 (2.4-3.7) | 14.6 (12.5-17.1) | 11.1 (9.5-13.2) | 20.5 (17.7-24) | 0.8 (-1.88-3.56) |
| CKD due to hypertension | Norway | 987.4 (887.2-1121.8) | 13.2 (11.9-14.7) | 1669.7 (1508.1-1834.8) | 15.5 (14-17) | 0.73 (-1.93-3.46) |
| CKD due to hypertension | Oman | 91.6 (77.9-108.6) | 13.9 (12.2-16.1) | 518.2 (435.5-615.5) | 25.7 (22.3-29.3) | 1.61 (-1.13-4.43) |
| CKD due to hypertension | Pakistan | 4789 (4223-5388.2) | 8.4 (7.5-9.4) | 13460 (11834-15302.4) | 10.7 (9.6-12.1) | 0.74 (-1.67-3.21) |
| CKD due to hypertension | Palau | 1.3 (1-1.5) | 12.4 (10.7-14.8) | 4.9 (4.1-5.8) | 20.8 (18.1-24.2) | 1.42 (-1.16-4.07) |
| CKD due to hypertension | Palestine | 150.9 (127.3-176.9) | 17.4 (14.9-20.3) | 685.5 (584.2-798.9) | 26.7 (23.1-30.5) | 0.97 (-1.77-3.79) |
| CKD due to hypertension | Panama | 221.8 (190.3-257.8) | 14.6 (12.6-16.9) | 981.1 (855.1-1116.5) | 22.2 (19.4-25.3) | 1.21 (-1.46-3.95) |
| CKD due to hypertension | Papua New Guinea | 102.1 (84.1-125) | 5.6 (4.8-6.6) | 395.2 (328.7-474.3) | 7.7 (6.6-8.8) | 0.67 (-1.57-2.96) |
| CKD due to hypertension | Paraguay | 259.2 (221-300.7) | 11.6 (9.9-13.4) | 1102.6 (952.7-1265.3) | 18.7 (16.2-21.3) | 1.34 (-1.32-4.07) |
| CKD due to hypertension | Peru | 1073.5 (913.8-1259.2) | 9.2 (7.9-10.7) | 5921.6 (5166.1-6703.5) | 17.8 (15.6-20.3) | 1.92 (-0.69-4.6) |
| CKD due to hypertension | Philippines | 3255.5 (2970.7-3556.3) | 11 (10-12) | 13301.4 (12240.5-14402.9) | 15.8 (14.5-17) | 1.09 (-1.39-3.64) |
| CKD due to hypertension | Poland | 3805.2 (3323-4289.5) | 8.7 (7.7-9.8) | 9342 (8412.2-10661.5) | 12.8 (11.6-14.5) | 0.17 (-2.3-2.7) |
| CKD due to hypertension | Portugal | 2076.7 (1786.4-2479.8) | 14.7 (12.8-17.2) | 4503.4 (3897.7-5203.4) | 16.6 (14.3-19) | 0.41 (-2.37-3.26) |
| CKD due to hypertension | Puerto Rico | 495.1 (425.6-568) | 13.6 (11.8-15.7) | 1392.3 (1220.3-1585.4) | 21.2 (18.5-24.3) | 1.37 (-1.28-4.09) |
| CKD due to hypertension | Qatar | 23 (18.6-27.5) | 19.1 (16.5-22.1) | 336 (276.2-398.2) | 30.6 (26.8-34.4) | 1.12 (-1.72-4.03) |
| CKD due to hypertension | Republic of Korea | 3716 (3165.1-4352) | 14.3 (12.3-16.5) | 16089.4 (14172.3-18247.7) | 16.9 (14.9-19.2) | 0.3 (-2.46-3.14) |
| CKD due to hypertension | Republic of Moldova | 161.5 (136.9-190.4) | 3.9 (3.4-4.6) | 398.8 (338-466.4) | 6.9 (5.8-8.1) | 1.37 (-0.87-3.65) |
| CKD due to hypertension | Romania | 1838.6 (1561.5-2163.6) | 6.7 (5.7-7.8) | 4912.8 (4279.6-5535.6) | 13.3 (11.6-15) | 1.62 (-0.88-4.19) |
| CKD due to hypertension | Russian Federation | 10348.1 (9246.4-11592) | 5.9 (5.3-6.6) | 24928.6 (22596.1-27297.1) | 10.9 (10-11.9) | 1.32 (-0.93-3.61) |
| CKD due to hypertension | Rwanda | 156.5 (131.1-184.4) | 5.8 (5.1-6.7) | 446.1 (381-521.2) | 7.5 (6.5-8.6) | 0.56 (-1.7-2.86) |
| CKD due to hypertension | Saint Kitts and Nevis | 4.8 (4.1-5.6) | 13.2 (11.3-15.4) | 15 (12.8-17.7) | 20.6 (17.7-24) | 1.35 (-1.27-4.04) |
| CKD due to hypertension | Saint Lucia | 10.5 (9-12.3) | 11.9 (10.3-13.8) | 45.2 (39.1-52.6) | 18.8 (16.2-21.8) | 1.26 (-1.35-3.94) |
| CKD due to hypertension | Saint Vincent and the Grenadines | 7.9 (6.8-9.1) | 11 (9.4-12.6) | 25 (21.3-28.9) | 17.4 (14.8-20.1) | 1.29 (-1.26-3.9) |
| CKD due to hypertension | Samoa | 9.7 (8.2-11.6) | 11.1 (9.6-13) | 23.5 (20.1-27.7) | 16.1 (14-18.6) | 0.84 (-1.73-3.48) |
| CKD due to hypertension | San Marino | 5.2 (4.4-6) | 13.9 (12-16) | 12.2 (10.5-13.8) | 14.8 (12.8-16.9) | 0.25 (-2.43-3.01) |
| CKD due to hypertension | Sao Tome and Principe | 6 (5.1-7.2) | 9.3 (7.9-10.9) | 15.6 (13.1-18.9) | 13.8 (11.9-16.2) | 1.07 (-1.34-3.55) |
| CKD due to hypertension | Saudi Arabia | 1100.7 (934.5-1291.2) | 18.4 (16.1-21) | 6705.3 (5647.3-7936.4) | 31.2 (27.7-34.9) | 1.46 (-1.34-4.33) |
| CKD due to hypertension | Senegal | 260.9 (221.4-307.5) | 8.1 (7.1-9.3) | 803.8 (683.2-946.4) | 10.4 (9-12) | 0.48 (-1.9-2.92) |
| CKD due to hypertension | Serbia | 952.2 (812.5-1123.7) | 8.3 (7.2-9.7) | 2654.4 (2297.4-3040.5) | 15.8 (13.8-18.1) | 1.76 (-0.86-4.46) |
| CKD due to hypertension | Seychelles | 7.4 (6.4-8.6) | 13 (11.3-15.1) | 22.7 (19.4-26.5) | 19.1 (16.6-22.3) | 0.98 (-1.71-3.74) |
| CKD due to hypertension | Sierra Leone | 154.8 (131.1-179.7) | 7.5 (6.5-8.6) | 371.5 (314.7-435.5) | 9.8 (8.5-11.2) | 0.62 (-1.71-3.01) |
| CKD due to hypertension | Singapore | 349.7 (307.6-391.8) | 16.9 (14.9-18.7) | 1720.2 (1511.2-1948.9) | 20.2 (17.7-22.8) | 0.5 (-2.28-3.37) |
| CKD due to hypertension | Slovakia | 502 (427-582.8) | 8.4 (7.2-9.7) | 1362.4 (1192.6-1566.1) | 14.1 (12.4-16.1) | 1.24 (-1.26-3.8) |
| CKD due to hypertension | Slovenia | 190.7 (163.5-222.3) | 7.8 (6.7-9.1) | 652.3 (559.9-755.1) | 14.6 (12.6-16.9) | 1.53 (-1.04-4.17) |
| CKD due to hypertension | Solomon Islands | 13.5 (10.4-17.6) | 8.9 (7.5-10.7) | 37.1 (29.5-46) | 10.3 (8.7-12.1) | 0.02 (-2.34-2.45) |
| CKD due to hypertension | Somalia | 130.7 (108.8-158.8) | 5.9 (5.1-6.7) | 398.4 (331.4-482.3) | 6.8 (5.8-7.9) | 0.33 (-1.9-2.61) |
| CKD due to hypertension | South Africa | 2166.3 (1965.9-2383.4) | 10.6 (9.6-11.7) | 6945.4 (6350.4-7576.5) | 15.1 (13.8-16.4) | 0.77 (-1.75-3.35) |
| CKD due to hypertension | South Sudan | 156.2 (136.7-180.7) | 6.3 (5.6-7.2) | 272.5 (233.1-317.4) | 7.6 (6.7-8.6) | 0.46 (-1.78-2.76) |
| CKD due to hypertension | Spain | 9341.5 (8130.7-10723) | 16.5 (14.4-18.7) | 17960.4 (15687.3-20218.2) | 16.7 (14.6-18.9) | 0.07 (-2.74-2.97) |
| CKD due to hypertension | Sri Lanka | 1224.8 (1042.1-1433.9) | 11.4 (9.8-13.2) | 4712.1 (4038.7-5504.8) | 16.9 (14.5-19.6) | 1.05 (-1.57-3.74) |
| CKD due to hypertension | Sudan | 1226.8 (1031.6-1440.4) | 13.6 (11.5-15.9) | 4103.3 (3488.5-4752.3) | 21.6 (18.8-24.7) | 1.12 (-1.59-3.91) |
| CKD due to hypertension | Suriname | 28.7 (24.1-34) | 10.9 (9.2-12.6) | 113.8 (97.6-131) | 17.7 (15.3-20.3) | 1.43 (-1.12-4.05) |
| CKD due to hypertension | Sweden | 2177.8 (1880.5-2512.6) | 13 (11.3-14.7) | 2966.5 (2503.7-3401.5) | 12.4 (10.6-14) | 0.1 (-2.55-2.82) |
| CKD due to hypertension | Switzerland | 1819.6 (1574.8-2108.2) | 16.3 (14.2-18.9) | 3556.3 (3103.2-4056.9) | 18.2 (15.9-20.8) | 0.3 (-2.51-3.19) |
| CKD due to hypertension | Syrian Arab Republic | 839.2 (717.1-990.5) | 16.3 (14.2-18.9) | 3391.5 (2927.7-3912.2) | 25.5 (22.2-29.3) | 1.02 (-1.68-3.8) |
| CKD due to hypertension | Taiwan (Province of China) | 2638.4 (2303.6-2977.1) | 17 (15-18.9) | 8324.9 (7412.3-9352.6) | 19.4 (17.3-21.7) | 0.53 (-2.18-3.32) |
| CKD due to hypertension | Tajikistan | 117.9 (94.3-146.7) | 4.1 (3.4-4.9) | 437 (362.2-533.8) | 7.3 (6.2-8.6) | 1.43 (-0.77-3.67) |
| CKD due to hypertension | Thailand | 4409 (3722.4-5258.2) | 12.2 (10.6-14.3) | 20201.5 (17682.7-22995.8) | 18.4 (16.2-21) | 0.76 (-1.82-3.41) |
| CKD due to hypertension | Timor-Leste | 25.1 (20.7-30.2) | 8.7 (7.5-10) | 104.4 (88.9-120.8) | 12.1 (10.3-13.8) | 0.82 (-1.64-3.33) |
| CKD due to hypertension | Togo | 99.6 (82.9-118.9) | 8.1 (7-9.4) | 396.3 (327.6-474.5) | 10.5 (8.9-12.1) | 0.52 (-1.85-2.94) |
| CKD due to hypertension | Tokelau | 0.1 (0.1-0.1) | 9.2 (7.9-10.5) | 0.2 (0.2-0.2) | 14.8 (12.8-16.8) | 1.15 (-1.4-3.77) |
| CKD due to hypertension | Tonga | 6.1 (5.1-7.3) | 10.8 (9.3-12.8) | 12.7 (10.9-14.8) | 15.8 (13.5-18.1) | 0.93 (-1.64-3.56) |
| CKD due to hypertension | Trinidad and Tobago | 91.7 (78.3-107.3) | 10.7 (9.2-12.5) | 355.8 (304.3-412.1) | 18.3 (15.7-21.2) | 1.54 (-1.04-4.18) |
| CKD due to hypertension | Tunisia | 773.2 (663.4-899.6) | 15.7 (13.6-18.1) | 3410.6 (2963.5-3905.2) | 25.4 (22.3-28.9) | 1.17 (-1.65-4.08) |
| CKD due to hypertension | Turkey | 4659.8 (4049.1-5332.3) | 14.2 (12.4-16) | 24297.1 (20790.4-27943.5) | 25.6 (22-29.3) | 1.52 (-1.24-4.37) |
| CKD due to hypertension | Turkmenistan | 120.8 (96-158.1) | 5.7 (4.7-6.9) | 399.4 (325.1-495.5) | 9.1 (7.6-11.1) | 1.39 (-0.84-3.67) |
| CKD due to hypertension | Tuvalu | 0.6 (0.5-0.7) | 8.5 (7.3-10.1) | 1.5 (1.2-1.8) | 14.2 (12.2-16.7) | 1.19 (-1.3-3.75) |
| CKD due to hypertension | Uganda | 336.5 (287.1-383.4) | 5.5 (4.8-6.3) | 931.3 (795.4-1071.5) | 6.7 (5.9-7.7) | 0.42 (-1.82-2.72) |
| CKD due to hypertension | Ukraine | 3096 (2606.8-3675.9) | 4.5 (3.8-5.3) | 5181.1 (4501.1-6044.8) | 7 (6.1-8.1) | 1.16 (-1.05-3.43) |
| CKD due to hypertension | United Arab Emirates | 104.5 (85.8-129.9) | 20.9 (18.3-23.7) | 1765.3 (1430.1-2204.1) | 31.3 (28.1-34.9) | 0.99 (-1.74-3.8) |
| CKD due to hypertension | United Kingdom | 14166 (12796.5-15897.3) | 14.5 (13.3-16.1) | 21994.3 (19914.8-24088.8) | 15.6 (14.2-17) | 0.02 (-2.63-2.75) |
| CKD due to hypertension | United Republic of Tanzania | 669.7 (571.8-776.1) | 6.5 (5.6-7.4) | 1940.1 (1651.6-2228.5) | 8 (6.9-9.1) | 0.44 (-1.82-2.76) |
| CKD due to hypertension | United States of America | 65628.3 (59605.9-72485.9) | 19.9 (18.1-21.8) | 129188.4 (119751.7-139094.4) | 21.5 (19.9-23.2) | 0.06 (-2.57-2.77) |
| CKD due to hypertension | United States Virgin Islands | 10.2 (8.7-11.9) | 11.7 (10-13.5) | 33.1 (28.6-38.3) | 18.7 (16.1-21.5) | 1.25 (-1.37-3.94) |
| CKD due to hypertension | Uruguay | 537.5 (469.4-614) | 13.4 (11.7-15.3) | 973.2 (858.9-1103) | 16.4 (14.5-18.6) | 0.59 (-2.01-3.25) |
| CKD due to hypertension | Uzbekistan | 895.2 (724.1-1129.6) | 7.2 (5.9-8.7) | 3099 (2556.4-3770.5) | 11 (9.3-13.1) | 1.1 (-1.2-3.45) |
| CKD due to hypertension | Vanuatu | 5.4 (4.6-6.6) | 8.6 (7.4-10) | 22.6 (18.9-27.2) | 12.8 (11.1-14.8) | 0.94 (-1.54-3.48) |
| CKD due to hypertension | Venezuela (Bolivarian Republic of) | 1462.4 (1243.1-1736.1) | 14.5 (12.5-16.8) | 6853.3 (5996.4-7777.4) | 22.4 (19.6-25.3) | 1.21 (-1.52-4.02) |
| CKD due to hypertension | Viet Nam | 3214.6 (2752.2-3813.8) | 8.1 (7-9.4) | 12643.1 (10776.9-15105.6) | 12.9 (11.1-15.1) | 1.36 (-1.13-3.91) |
| CKD due to hypertension | Yemen | 611.1 (501.5-720.8) | 13.1 (11-15.3) | 2640 (2210.8-3056.1) | 19.4 (16.7-22.3) | 0.98 (-1.7-3.73) |
| CKD due to hypertension | Zambia | 183 (156-216.7) | 6.7 (5.8-7.7) | 566.5 (477.2-671.1) | 8.5 (7.3-9.8) | 0.5 (-1.8-2.86) |
| CKD due to hypertension | Zimbabwe | 401 (339.5-475.4) | 10 (8.7-11.5) | 796.9 (670.2-950.8) | 11.7 (10.2-13.5) | 0.19 (-2.27-2.72) |
| CKD due to other and unspecified causes | Afghanistan | 15142.7 (13438.2-16910.8) | 207.4 (187.4-229.5) | 36727.2 (33123-40545.1) | 299.9 (275.3-329.7) | 0.91 (-1.84-3.73) |
| CKD due to other and unspecified causes | Albania | 2227.2 (1963.4-2494.2) | 96.5 (85.7-108) | 6968 (6239-7686) | 166.4 (150.8-182.5) | 1.71 (-0.68-4.16) |
| CKD due to other and unspecified causes | Algeria | 28684.1 (25846.7-31640) | 208.2 (189.2-229.8) | 124494 (114254.1-135253.7) | 330.8 (305.7-357.4) | 1.19 (-1.58-4.04) |
| CKD due to other and unspecified causes | American Samoa | 48.5 (43.1-53.5) | 171 (156.3-187.1) | 127 (115.5-139.6) | 253.3 (233.3-274.2) | 1.01 (-1.62-3.71) |
| CKD due to other and unspecified causes | Andorra | 102.6 (92-114.7) | 182.1 (165.4-202.2) | 274.5 (251.7-301) | 180.9 (165.3-198.7) | 0.1 (-2.63-2.9) |
| CKD due to other and unspecified causes | Angola | 3659.6 (3115.7-4197.2) | 71.4 (65.1-77.6) | 15465.6 (13471.1-17304.8) | 104.2 (94.3-113.5) | 0.82 (-1.46-3.14) |
| CKD due to other and unspecified causes | Antigua and Barbuda | 92.1 (84.6-100.3) | 177.2 (163.1-192.4) | 305.7 (278.7-333) | 279.4 (255.7-302.6) | 1.35 (-1.26-4.03) |
| CKD due to other and unspecified causes | Argentina | 55168.1 (50121.3-60909.5) | 172.3 (157.3-189.7) | 122401.5 (111824.5-133028.9) | 217.7 (199-235.3) | 0.76 (-1.89-3.48) |
| CKD due to other and unspecified causes | Armenia | 2270.2 (1972.5-2575.2) | 76.2 (67.1-85.2) | 6130 (5480.9-6813.6) | 149.5 (135.6-164.6) | 1.98 (-0.36-4.38) |
| CKD due to other and unspecified causes | Australia | 42116.7 (39793.2-44713.2) | 209.7 (198.6-222.1) | 109926.4 (100375.6-118119.5) | 240.2 (221.2-257.3) | 0.53 (-2.2-3.35) |
| CKD due to other and unspecified causes | Austria | 22559.2 (20231.5-25187) | 183.7 (166.3-202.8) | 41363.4 (37929.1-45140.6) | 224.5 (203.2-247) | 0.75 (-1.99-3.58) |
| CKD due to other and unspecified causes | Azerbaijan | 5261.1 (4622.5-5954.6) | 89 (79.5-98.8) | 18217.8 (16183.9-20303) | 164.7 (150.2-180.5) | 1.98 (-0.29-4.31) |
| CKD due to other and unspecified causes | Bahamas | 279.6 (257.7-306.6) | 159.9 (146.7-176.4) | 1023.1 (931.2-1115.2) | 240.5 (221.4-258.4) | 1.27 (-1.29-3.9) |
| CKD due to other and unspecified causes | Bahrain | 490.7 (437.2-544.7) | 236.4 (215-260.7) | 3956.6 (3522.9-4439.1) | 363.2 (332.9-393.4) | 1.18 (-1.68-4.13) |
| CKD due to other and unspecified causes | Bangladesh | 40358 (35488.9-45485.4) | 71.7 (64.8-79.2) | 151116.9 (135715.8-167129) | 106 (95.7-116.5) | 0.85 (-1.45-3.2) |
| CKD due to other and unspecified causes | Barbados | 423.9 (386.5-471.4) | 157.5 (144.2-173.9) | 1200.7 (1097.9-1314.4) | 250.3 (231.3-271) | 1.31 (-1.25-3.95) |
| CKD due to other and unspecified causes | Belarus | 8538.7 (7712.4-9607.3) | 70.3 (63.4-78.5) | 16371.3 (14740.7-18178) | 114.7 (103.9-125.6) | 1.3 (-0.93-3.59) |
| CKD due to other and unspecified causes | Belgium | 31021 (28242.3-34483.2) | 195.7 (179-214.7) | 47706.4 (43574.4-51448) | 201 (183.5-216.1) | 0.2 (-2.55-3.04) |
| CKD due to other and unspecified causes | Belize | 168.9 (150.5-184.9) | 156.4 (141.2-171.8) | 856.1 (780.9-923.7) | 253.4 (232.5-273.6) | 1.35 (-1.18-3.94) |
| CKD due to other and unspecified causes | Benin | 2833.1 (2409.6-3228.3) | 109 (99.1-119.8) | 9572.4 (8387.1-10677.4) | 143.7 (130.8-156.9) | 0.61 (-1.76-3.04) |
| CKD due to other and unspecified causes | Bermuda | 98.5 (88.7-108.4) | 156 (141.1-171.6) | 318.9 (290.9-347.8) | 260.4 (239.8-284) | 1.57 (-1.07-4.29) |
| CKD due to other and unspecified causes | Bhutan | 334.4 (292.7-372.7) | 101.1 (91.9-111.7) | 973.1 (886.4-1058.5) | 150.4 (137-164.4) | 1.12 (-1.29-3.58) |
| CKD due to other and unspecified causes | Bolivia (Plurinational State of) | 5080.4 (4562.3-5602.7) | 141.1 (129-154) | 19881.6 (18227.6-21701.4) | 211.9 (194.8-230.8) | 1.13 (-1.44-3.77) |
| CKD due to other and unspecified causes | Bosnia and Herzegovina | 4184.9 (3770.1-4622.9) | 97.9 (88.7-107) | 10972.8 (10023.1-11977.3) | 184.7 (170.2-199.1) | 1.82 (-0.72-4.42) |
| CKD due to other and unspecified causes | Botswana | 794.8 (714.2-879.6) | 122.1 (111.6-133) | 2844.4 (2593.1-3113.1) | 176 (161.4-192.3) | 0.84 (-1.65-3.4) |
| CKD due to other and unspecified causes | Brazil | 152564.7 (141142.3-165431.7) | 156.1 (144-170.2) | 525707.7 (490564.6-561260.1) | 209.8 (196.6-223.2) | 0.73 (-1.81-3.35) |
| CKD due to other and unspecified causes | Brunei Darussalam | 256.9 (234-282.2) | 237.1 (216.7-260.4) | 911.6 (828.5-1011.9) | 264.8 (244.6-288.5) | 0.43 (-2.3-3.24) |
| CKD due to other and unspecified causes | Bulgaria | 12400.2 (11068-13984.9) | 104 (94.5-115.3) | 24080.9 (21768.9-26533.2) | 187.9 (173.4-203.7) | 1.73 (-0.7-4.22) |
| CKD due to other and unspecified causes | Burkina Faso | 5489.7 (4718-6164.2) | 101.6 (92.3-111.1) | 15675.9 (13578.2-17341.4) | 133.6 (123-145.5) | 0.78 (-1.56-3.17) |
| CKD due to other and unspecified causes | Burundi | 2184.3 (1891.3-2454.5) | 75.2 (68.1-82.5) | 5210 (4565.8-5833.1) | 87.8 (80.7-96) | 0.33 (-1.91-2.61) |
| CKD due to other and unspecified causes | Cabo Verde | 236.6 (209.5-264.6) | 91.9 (83.1-101.3) | 662.5 (598.2-724.1) | 145.1 (130.8-159) | 1.1 (-1.26-3.52) |
| CKD due to other and unspecified causes | Cambodia | 5916 (5132-6610.5) | 102.7 (93.1-114.4) | 19974.3 (18026.4-22126.9) | 149.5 (135.8-163.9) | 0.89 (-1.49-3.33) |
| CKD due to other and unspecified causes | Cameroon | 8311.5 (7214.8-9306.8) | 143.8 (131.1-157.6) | 31291 (27924-34352) | 191 (175.3-207) | 0.66 (-1.77-3.16) |
| CKD due to other and unspecified causes | Canada | 69582.2 (63452.2-74103.8) | 212.5 (195-225.8) | 149773 (136650.8-166860.2) | 207.4 (189.8-230) | 0.34 (-2.23-2.97) |
| CKD due to other and unspecified causes | Central African Republic | 1061.9 (907.6-1203.4) | 73.2 (66.4-80) | 2669.2 (2360.6-2985.6) | 95.6 (87.6-103.9) | 0.58 (-1.68-2.9) |
| CKD due to other and unspecified causes | Chad | 3624.1 (3153.2-4086.5) | 102.5 (93.7-113) | 10051.8 (8652-11395.1) | 124.4 (113.9-136.7) | 0.48 (-1.86-2.87) |
| CKD due to other and unspecified causes | Chile | 17471.9 (15697.4-19291.1) | 175 (157.7-192.7) | 65135.6 (60057.8-70032.4) | 255.8 (235.8-274.8) | 1.19 (-1.54-4.01) |
| CKD due to other and unspecified causes | China | 1013766.2 (915004.5-1111441.3) | 118.2 (107.4-129.8) | 2702516.3 (2497806.1-2898326.5) | 132.6 (123.9-141.2) | 0.25 (-2.21-2.76) |
| CKD due to other and unspecified causes | Colombia | 36425.4 (32482.9-40651.8) | 183.1 (164.8-203.2) | 139243 (127124.5-151706.7) | 254.5 (232.8-276.7) | 0.97 (-1.67-3.69) |
| CKD due to other and unspecified causes | Comoros | 205.5 (172.8-234.5) | 82.7 (75-90.7) | 537.9 (491.9-592) | 102.8 (94.6-112.5) | 0.38 (-1.89-2.7) |
| CKD due to other and unspecified causes | Congo | 1084.4 (937.3-1224.2) | 82.3 (75.2-90.3) | 3740.2 (3372.1-4117.7) | 119.1 (109.8-130.1) | 0.82 (-1.52-3.21) |
| CKD due to other and unspecified causes | Cook Islands | 19.1 (16.9-21) | 140.1 (125.9-153.7) | 56.6 (51-61.9) | 230.9 (210.6-250.6) | 1.27 (-1.36-3.97) |
| CKD due to other and unspecified causes | Costa Rica | 6601.4 (6339.6-6862.7) | 353.4 (342.5-364.5) | 20386.1 (19812.2-20888.9) | 370.4 (358.6-380.6) | 0.16 (-2.67-3.07) |
| CKD due to other and unspecified causes | C么te d'Ivoire | 6921.5 (5958.6-7771.2) | 120.8 (110.7-131.4) | 22356.9 (19923.8-24393) | 155.6 (142.2-169.6) | 0.54 (-1.85-3) |
| CKD due to other and unspecified causes | Croatia | 7425.1 (6629.5-8324.2) | 121.7 (110.1-135.3) | 17731.4 (16121-19262.3) | 210.4 (191.8-227.5) | 1.64 (-0.99-4.34) |
| CKD due to other and unspecified causes | Cuba | 12194.4 (11136-13392.2) | 118.5 (107.4-129.8) | 40137.6 (36582.5-43701.8) | 220.3 (201.8-240) | 1.7 (-0.86-4.31) |
| CKD due to other and unspecified causes | Cyprus | 1872 (1674.1-2110.1) | 222.7 (205.8-243.1) | 5199.9 (4759.3-5743.2) | 239.7 (220.3-263) | 0.33 (-2.54-3.29) |
| CKD due to other and unspecified causes | Czechia | 13956.6 (12540.9-15657.8) | 105.1 (95.2-116.6) | 36319.1 (33024.9-40132.5) | 176.6 (162.1-192.7) | 1.35 (-1.2-3.96) |
| CKD due to other and unspecified causes | Democratic People's Republic of Korea | 18590.4 (16683.7-20509.5) | 111.9 (101.2-122.1) | 47932.5 (43121.3-52469.9) | 150.7 (135.8-164) | 0.71 (-1.75-3.23) |
| CKD due to other and unspecified causes | Democratic Republic of the Congo | 14968.7 (12915-17003.4) | 75.9 (68.8-83.1) | 45308.6 (40271.4-50138.2) | 103.9 (94.9-114.1) | 0.64 (-1.66-3) |
| CKD due to other and unspecified causes | Denmark | 14018.5 (12506-15617.1) | 169 (152.6-186.8) | 24135.9 (21991.4-26472.9) | 197.6 (181.3-213.5) | 0.64 (-2.02-3.38) |
| CKD due to other and unspecified causes | Djibouti | 141.3 (121.8-159.6) | 78.9 (71.9-86.8) | 740.9 (668.1-809.7) | 102.9 (94.2-112.4) | 0.59 (-1.66-2.9) |
| CKD due to other and unspecified causes | Dominica | 112.2 (103.2-122.3) | 186.3 (171.6-202.9) | 212.8 (193.4-232.2) | 259.3 (237.7-281.4) | 1.08 (-1.46-3.68) |
| CKD due to other and unspecified causes | Dominican Republic | 4919.1 (4393-5527.8) | 112.2 (101.6-125.3) | 20303.1 (18607.9-22050.1) | 197.3 (181-214.1) | 1.55 (-0.94-4.1) |
| CKD due to other and unspecified causes | Ecuador | 8579.2 (7685.4-9521.2) | 145.9 (131.5-161.5) | 47080.3 (43203.8-51473) | 282.8 (259.6-309.1) | 1.93 (-0.77-4.71) |
| CKD due to other and unspecified causes | Egypt | 65171.5 (58329-72692.8) | 214.8 (195.8-236.5) | 244702.7 (220742.2-272914.6) | 347.9 (321.7-380.2) | 1.25 (-1.52-4.09) |
| CKD due to other and unspecified causes | El Salvador | 6166.3 (5572.4-6825.5) | 185.7 (168.2-206.5) | 20315.8 (18816.7-21714) | 332 (305.9-355.9) | 2.06 (-0.6-4.79) |
| CKD due to other and unspecified causes | Equatorial Guinea | 173.6 (148.8-194.8) | 74.2 (67.5-81.3) | 845.8 (752.7-930.7) | 133.7 (121.7-146.6) | 1.63 (-0.74-4.07) |
| CKD due to other and unspecified causes | Eritrea | 1118.4 (936.3-1267.4) | 73.3 (66.9-80.5) | 3145.5 (2742.6-3466.4) | 94.4 (85.7-103.1) | 0.48 (-1.76-2.78) |
| CKD due to other and unspecified causes | Estonia | 1622.1 (1436.1-1825.9) | 84.5 (75.7-94.3) | 4004.4 (3648.5-4403.9) | 175.3 (159.6-192.6) | 2.1 (-0.27-4.53) |
| CKD due to other and unspecified causes | Eswatini | 539.5 (475.6-608.9) | 149 (135-164.3) | 1257.1 (1146.5-1377.9) | 188.5 (171.6-204.6) | 0.54 (-1.92-3.07) |
| CKD due to other and unspecified causes | Ethiopia | 19010 (17095.5-20976.2) | 74.2 (68-80.7) | 47526.8 (43557.6-51283.7) | 92 (84.2-99.8) | 0.38 (-1.86-2.66) |
| CKD due to other and unspecified causes | Fiji | 704.3 (631.5-775.3) | 157.1 (143.7-171.6) | 1749.2 (1594.4-1896.4) | 211.6 (196.1-228.4) | 0.71 (-1.79-3.28) |
| CKD due to other and unspecified causes | Finland | 10278.9 (9186.9-11579.1) | 140.8 (126.5-156.8) | 21310.9 (19423.6-23773.8) | 160.1 (146.8-175.9) | 0.47 (-2.17-3.18) |
| CKD due to other and unspecified causes | France | 146827.5 (132725.4-164888.1) | 168.8 (153.6-187.6) | 267027.4 (245233.7-288796.3) | 187.2 (172-203.5) | 0.33 (-2.4-3.14) |
| CKD due to other and unspecified causes | Gabon | 590.7 (524.3-654.4) | 92.9 (85-101.5) | 1699.4 (1534.4-1846.6) | 147.6 (135-160.1) | 1.05 (-1.35-3.51) |
| CKD due to other and unspecified causes | Gambia | 507.9 (439-577.3) | 107.4 (98.2-116.6) | 1742.7 (1576.8-1908.4) | 142.7 (130.3-155.8) | 0.63 (-1.76-3.08) |
| CKD due to other and unspecified causes | Georgia | 5169.6 (4600.4-5774.2) | 85.1 (76.9-94.5) | 7544.1 (6815.7-8273.9) | 144.7 (131.9-157.5) | 1.56 (-0.76-3.94) |
| CKD due to other and unspecified causes | Germany | 256088.5 (231790-285108.8) | 198.2 (180.8-220.6) | 429660.6 (399653.9-461697.7) | 219.3 (205.7-233.8) | 0.35 (-2.39-3.18) |
| CKD due to other and unspecified causes | Ghana | 7757.2 (6798.3-8619) | 97.2 (89-106.3) | 27756 (24960.1-30692.3) | 139.1 (126.2-152.1) | 0.92 (-1.48-3.37) |
| CKD due to other and unspecified causes | Greece | 35722.8 (32487.5-39173.9) | 230.1 (209.7-252) | 53278.3 (49262.8-57885.3) | 223.5 (204.9-241.3) | 0.07 (-2.76-2.99) |
| CKD due to other and unspecified causes | Greenland | 48.7 (43.5-53.4) | 164.1 (148.2-180.4) | 120 (110.3-132.9) | 186.5 (171.9-203.2) | 0.43 (-2.15-3.06) |
| CKD due to other and unspecified causes | Grenada | 119.4 (110-129.5) | 170.2 (156.8-185.2) | 335.5 (308.1-362.8) | 287.4 (266.6-309) | 1.46 (-1.13-4.12) |
| CKD due to other and unspecified causes | Guam | 133.5 (119-148.6) | 145.7 (131.4-161.3) | 433.6 (393.1-477.6) | 215.1 (195.6-235.5) | 1.15 (-1.39-3.75) |
| CKD due to other and unspecified causes | Guatemala | 8348 (7354.3-9296) | 196.8 (177.4-216.7) | 37199.5 (34187.2-40761.4) | 314.9 (289.9-343.1) | 1.42 (-1.25-4.15) |
| CKD due to other and unspecified causes | Guinea | 4254.3 (3744.2-4753.1) | 107.9 (97.7-119.1) | 9814.6 (8721-10857.4) | 138.1 (126.5-150.7) | 0.57 (-1.78-2.99) |
| CKD due to other and unspecified causes | Guinea-Bissau | 615.2 (534.4-693.9) | 114.9 (105.6-124.7) | 1394.7 (1239.4-1562.6) | 138 (126-150.7) | 0.37 (-1.99-2.77) |
| CKD due to other and unspecified causes | Guyana | 665.4 (595.8-729) | 143.2 (130.7-155.4) | 1665.5 (1520.1-1804.5) | 239.7 (221-258.4) | 1.35 (-1.21-3.97) |
| CKD due to other and unspecified causes | Haiti | 5401.5 (4784.4-6063.2) | 138.2 (124.3-155.3) | 15689.1 (14261.2-17207.3) | 184.1 (167.6-201.6) | 0.75 (-1.76-3.32) |
| CKD due to other and unspecified causes | Honduras | 4717.4 (4225-5300) | 191.2 (173.2-212.3) | 18490.1 (16983.2-20066.1) | 261.5 (241-284.1) | 0.82 (-1.85-3.57) |
| CKD due to other and unspecified causes | Hungary | 12711.7 (11424.7-14245.5) | 90.1 (81.1-100.1) | 34347 (31442.9-38206) | 184.8 (169-203) | 1.92 (-0.63-4.53) |
| CKD due to other and unspecified causes | Iceland | 456.1 (414.4-502.4) | 156.3 (142.8-171.4) | 948.4 (863.8-1029) | 162.1 (147.6-177.3) | 0.14 (-2.54-2.89) |
| CKD due to other and unspecified causes | India | 677976.4 (621542-737160.6) | 123 (113.1-133.8) | 1811870.9 (1664696.9-1962359.5) | 146.1 (134.4-158.1) | 0.2 (-2.29-2.75) |
| CKD due to other and unspecified causes | Indonesia | 123510.8 (113841.6-134478.4) | 104.9 (96.5-114.1) | 381338 (351242.8-416314.8) | 149.7 (138.5-161.8) | 0.85 (-1.44-3.19) |
| CKD due to other and unspecified causes | Iran (Islamic Republic of) | 74211.2 (66751.8-82458.4) | 246.2 (225.3-271) | 271032.6 (252667.7-290792.4) | 332.8 (311.4-355.7) | 0.68 (-2.02-3.44) |
| CKD due to other and unspecified causes | Iraq | 21628 (19522.3-23683.8) | 236.7 (216.4-259.9) | 95780.6 (87842.5-104669.9) | 357.9 (329.6-387.1) | 1.1 (-1.69-3.96) |
| CKD due to other and unspecified causes | Ireland | 9259.8 (8715.5-9708.3) | 222.5 (209.4-232.7) | 15625.7 (15298.3-15948.9) | 199.1 (194.9-203.4) | 0.06 (-2.55-2.74) |
| CKD due to other and unspecified causes | Israel | 11045.5 (9969.1-12201.2) | 221.3 (201.7-243) | 30703.1 (27598.1-33589.4) | 247.8 (222.5-269.7) | 0.45 (-2.39-3.37) |
| CKD due to other and unspecified causes | Italy | 159706.1 (143233.4-177010) | 176.5 (159.4-194.1) | 273053.5 (251420.7-295423.4) | 177.8 (165.1-191.5) | 0.19 (-2.44-2.9) |
| CKD due to other and unspecified causes | Jamaica | 2987.2 (2725.9-3285.9) | 165.2 (150.8-181.8) | 7343.7 (6734.3-7942.3) | 240.5 (222-259.8) | 1.11 (-1.47-3.77) |
| CKD due to other and unspecified causes | Japan | 381761.7 (351417-413968.8) | 226.2 (209.5-244) | 807226.5 (737573.3-875990) | 229.8 (212.9-245.9) | 0.09 (-2.7-2.96) |
| CKD due to other and unspecified causes | Jordan | 3729.8 (3366-4084.3) | 235.4 (217-255.3) | 30394.4 (28267.1-32747.4) | 364.1 (339.8-387.9) | 1.23 (-1.59-4.13) |
| CKD due to other and unspecified causes | Kazakhstan | 11748.2 (10298.3-13012.2) | 82.3 (73-90.8) | 28536.4 (25767.9-31683.8) | 149.7 (136.8-164.9) | 1.58 (-0.73-3.95) |
| CKD due to other and unspecified causes | Kenya | 7799.5 (7260.7-8376.8) | 74.3 (68.3-80.9) | 25091.7 (23000.4-27144) | 95.8 (88-104.4) | 0.41 (-1.83-2.7) |
| CKD due to other and unspecified causes | Kiribati | 61.5 (53.8-68.1) | 125.6 (113.5-138) | 156 (142.2-171.3) | 176.1 (162.7-190.7) | 0.83 (-1.61-3.34) |
| CKD due to other and unspecified causes | Kuwait | 1821.3 (1651.7-1978) | 236.5 (218.6-256.2) | 10763.7 (9663.7-11926.2) | 321.1 (294.4-349.1) | 0.88 (-1.9-3.73) |
| CKD due to other and unspecified causes | Kyrgyzstan | 3174.8 (2776.7-3544.4) | 85.1 (76.7-94.1) | 7153.4 (6363.3-7910.5) | 122.7 (111.2-135.2) | 0.92 (-1.3-3.19) |
| CKD due to other and unspecified causes | Lao People's Democratic Republic | 3462 (3049.7-3853.8) | 137.4 (125.1-150.9) | 9726.7 (8827.3-10597.5) | 184.8 (169.3-200.6) | 0.77 (-1.67-3.28) |
| CKD due to other and unspecified causes | Latvia | 2335.9 (2066.2-2624.9) | 70.6 (62.7-79.2) | 4433.6 (3992.5-4905) | 134.9 (122.7-148.4) | 1.61 (-0.69-3.96) |
| CKD due to other and unspecified causes | Lebanon | 4946.7 (4463.6-5508.8) | 217.1 (198-238.4) | 20617.4 (19011.6-22327.1) | 354.1 (323.3-385) | 1.3 (-1.5-4.19) |
| CKD due to other and unspecified causes | Lesotho | 1130.3 (1024.1-1246.5) | 121.6 (110.7-133.6) | 1984.8 (1819.1-2160.2) | 164.1 (151.4-176.9) | 0.68 (-1.75-3.18) |
| CKD due to other and unspecified causes | Liberia | 1518.6 (1326.8-1711.7) | 104.5 (95.6-114.7) | 3926.1 (3528.8-4325.2) | 143.9 (131.1-157.4) | 0.88 (-1.51-3.34) |
| CKD due to other and unspecified causes | Libya | 4516.9 (4079.7-5014.9) | 212.8 (192.9-235.4) | 19135.5 (17514.7-20875.4) | 340.2 (312.8-370.4) | 1.06 (-1.74-3.94) |
| CKD due to other and unspecified causes | Lithuania | 3415.2 (3079.9-3817.1) | 80 (72.4-89.2) | 5701.7 (5186.3-6206.7) | 119.9 (110.4-130.1) | 1.08 (-1.19-3.41) |
| CKD due to other and unspecified causes | Luxembourg | 1116.6 (1013.6-1234) | 198.6 (181.4-217.7) | 2249.4 (2058.4-2409.9) | 213.8 (195.9-229.8) | 0.38 (-2.41-3.26) |
| CKD due to other and unspecified causes | Madagascar | 4386.4 (3722.9-4974.3) | 68.6 (62.3-75.5) | 11793.3 (10488.8-13078.6) | 86.1 (77.7-93.8) | 0.41 (-1.79-2.66) |
| CKD due to other and unspecified causes | Malawi | 3723.8 (3215.4-4233.8) | 77.7 (70.8-84.4) | 8757.4 (7794.4-9672.2) | 99.8 (90.3-108.6) | 0.64 (-1.61-2.94) |
| CKD due to other and unspecified causes | Malaysia | 14903.5 (13566.7-16284.4) | 142.4 (128.8-155.1) | 62948.1 (57681.8-68205) | 211.6 (195-229.3) | 1.04 (-1.57-3.71) |
| CKD due to other and unspecified causes | Maldives | 167.8 (148.7-186.8) | 150.3 (136-166) | 862.6 (775.2-943.3) | 232.9 (209.4-257) | 1.18 (-1.41-3.83) |
| CKD due to other and unspecified causes | Mali | 5104.9 (4419.3-5768.4) | 100.5 (91.7-109.7) | 15109.7 (13260.5-16946.1) | 128.7 (118.1-138.8) | 0.6 (-1.76-3) |
| CKD due to other and unspecified causes | Malta | 891.5 (812.2-980.7) | 208.5 (190.6-228.2) | 2166.2 (1975.5-2384.6) | 216.4 (200.1-236.2) | 0.26 (-2.47-3.06) |
| CKD due to other and unspecified causes | Marshall Islands | 30.7 (27.5-33.9) | 135.1 (124-147.6) | 83.3 (75.8-90.7) | 199.2 (183.8-215.7) | 0.9 (-1.63-3.49) |
| CKD due to other and unspecified causes | Mauritania | 1480.3 (1310.4-1663.6) | 120.2 (109.9-131.7) | 4165.6 (3758.1-4602) | 162.7 (148-178.7) | 0.79 (-1.62-3.26) |
| CKD due to other and unspecified causes | Mauritius | 1681.5 (1523.3-1853.9) | 207.2 (187.1-226.8) | 6082.3 (5661.6-6592.5) | 330.9 (310.3-355.6) | 1.48 (-1.15-4.19) |
| CKD due to other and unspecified causes | Mexico | 121767.8 (111662.7-133739.7) | 247.8 (226.2-272.9) | 501056.5 (474340-527216.1) | 378.7 (359.1-397.7) | 1.37 (-1.37-4.18) |
| CKD due to other and unspecified causes | Micronesia (Federated States of) | 91.5 (81.9-101.6) | 150.8 (137.3-166.1) | 201.2 (180.4-220.7) | 236.7 (217.8-256.6) | 1.15 (-1.4-3.77) |
| CKD due to other and unspecified causes | Monaco | 127.7 (114.7-142.9) | 172.5 (156.5-190.5) | 185.7 (167.6-204.8) | 186.1 (170.5-203.7) | 0.32 (-2.39-3.1) |
| CKD due to other and unspecified causes | Mongolia | 1618.4 (1408.4-1815.9) | 112.9 (100.3-125.4) | 4298.4 (3856.6-4703.1) | 153.4 (138.3-167.2) | 0.7 (-1.62-3.08) |
| CKD due to other and unspecified causes | Montenegro | 914.9 (824.2-1009.3) | 145.8 (131.3-160.3) | 2140.4 (1911.4-2377.4) | 222.1 (201.3-244) | 1.24 (-1.28-3.83) |
| CKD due to other and unspecified causes | Morocco | 26176.8 (23617.3-28676.9) | 170.4 (154.9-187.8) | 110195.3 (100101.8-120004) | 312 (285.3-338.7) | 1.62 (-1.1-4.43) |
| CKD due to other and unspecified causes | Mozambique | 5321.8 (4536.3-6022.9) | 74.4 (67.6-82) | 13313.9 (11608.4-14992.1) | 95.3 (86.5-104.3) | 0.55 (-1.67-2.82) |
| CKD due to other and unspecified causes | Myanmar | 35213.7 (31038.6-39444.2) | 129.8 (116.8-144.4) | 94809.1 (86257.7-102825.7) | 185.3 (170.3-200.4) | 0.97 (-1.45-3.45) |
| CKD due to other and unspecified causes | Namibia | 914.6 (820.3-1004.9) | 123.9 (113.3-135.2) | 2406.2 (2186.1-2647.8) | 160.4 (146.6-175.9) | 0.59 (-1.89-3.12) |
| CKD due to other and unspecified causes | Nauru | 8.7 (7.7-9.6) | 144.5 (131.5-158.5) | 15 (13.4-16.4) | 208.3 (188.8-227.3) | 0.85 (-1.66-3.42) |
| CKD due to other and unspecified causes | Nepal | 11664.8 (10173.2-13119.8) | 99.9 (90.4-110.8) | 44464 (40086.2-48641.7) | 174.1 (158.5-189.5) | 1.2 (-1.28-3.74) |
| CKD due to other and unspecified causes | Netherlands | 35973.1 (33232-39005.8) | 173.8 (161.7-188.1) | 72067.8 (66371-78180.5) | 197.2 (182-213.3) | 0.62 (-2.11-3.43) |
| CKD due to other and unspecified causes | New Zealand | 8625.3 (7818.1-9550.6) | 215.1 (197.1-235.6) | 20568.8 (18674.1-22426.6) | 244.1 (223.1-265.9) | 0.55 (-2.16-3.34) |
| CKD due to other and unspecified causes | Nicaragua | 4391.5 (3928.7-4833.3) | 223.2 (202.5-247.2) | 19554 (18197.6-20942.9) | 355 (330.5-378.7) | 1.31 (-1.31-4) |
| CKD due to other and unspecified causes | Niger | 3961.3 (3351-4549) | 100 (91.4-109.7) | 13778.2 (12007.6-15379) | 122.7 (111.1-134.4) | 0.42 (-1.91-2.81) |
| CKD due to other and unspecified causes | Nigeria | 58908.4 (54781.1-63666.5) | 109.3 (100.4-119.1) | 166041.9 (153592.8-177690.7) | 146.7 (133.6-159) | 0.68 (-1.68-3.1) |
| CKD due to other and unspecified causes | Niue | 3.2 (2.9-3.5) | 147.6 (134.1-161.9) | 4.7 (4.3-5.3) | 225.5 (205.7-248) | 1.11 (-1.46-3.75) |
| CKD due to other and unspecified causes | North Macedonia | 2091.2 (1913-2298.5) | 108.7 (99.8-118.7) | 7057.3 (6248.9-7712.1) | 210.7 (190.3-228.2) | 1.86 (-0.69-4.48) |
| CKD due to other and unspecified causes | Northern Mariana Islands | 50.2 (43.5-55.9) | 193.5 (172.5-212.1) | 156.4 (139.1-178.8) | 276.5 (253.4-308.5) | 0.8 (-1.88-3.56) |
| CKD due to other and unspecified causes | Norway | 10713.2 (9601.9-12083.3) | 148.7 (135.2-164.8) | 17802.8 (16288.3-19275.6) | 172.4 (158.2-185.7) | 0.73 (-1.93-3.46) |
| CKD due to other and unspecified causes | Oman | 1395.2 (1236.4-1535.6) | 176.8 (159-193.4) | 7412.7 (6694.4-8121.2) | 320.6 (294.6-348.9) | 1.61 (-1.13-4.43) |
| CKD due to other and unspecified causes | Pakistan | 78270.2 (71511-85834.5) | 114.9 (105.3-126) | 222398.3 (205089.6-240893.6) | 147.4 (135.3-159.2) | 0.74 (-1.67-3.21) |
| CKD due to other and unspecified causes | Palau | 19.3 (17.1-21.9) | 171.9 (153.3-193.1) | 66.6 (60.1-75.3) | 281.2 (259.1-310.7) | 1.42 (-1.16-4.07) |
| CKD due to other and unspecified causes | Palestine | 2374.7 (2123.6-2654.9) | 235.9 (215-259.7) | 9996.6 (9217.5-10895.3) | 349.2 (322.3-376.9) | 0.97 (-1.77-3.79) |
| CKD due to other and unspecified causes | Panama | 3181.2 (2885.9-3505.7) | 196.3 (178.1-216.8) | 12913.8 (12100.9-13823) | 293.9 (275.1-314.8) | 1.21 (-1.46-3.95) |
| CKD due to other and unspecified causes | Papua New Guinea | 1910 (1651.9-2193.8) | 77.7 (69-87.4) | 6808.4 (5966.5-7537) | 103.3 (93.1-112.8) | 0.67 (-1.57-2.96) |
| CKD due to other and unspecified causes | Paraguay | 3812.5 (3459.4-4222) | 155.4 (141.5-172.4) | 15243.1 (13943.6-16625.6) | 250.2 (229.4-272.5) | 1.34 (-1.32-4.07) |
| CKD due to other and unspecified causes | Peru | 15692 (14231-17211.3) | 120.3 (109.6-131.9) | 77511.7 (71503.3-84413.7) | 230.6 (212.5-251.5) | 1.92 (-0.69-4.6) |
| CKD due to other and unspecified causes | Philippines | 52601.8 (48901.6-56541.3) | 149.3 (137.5-161.6) | 199751.7 (186956.7-213786.1) | 220.7 (206.9-235.6) | 1.09 (-1.39-3.64) |
| CKD due to other and unspecified causes | Poland | 51169.6 (45212.5-57306.5) | 119.4 (106.8-133) | 112282 (100815.4-125452.6) | 163.2 (148.1-180.4) | 0.17 (-2.3-2.7) |
| CKD due to other and unspecified causes | Portugal | 24042.3 (21287.3-26902.4) | 171.6 (154.2-189.3) | 49065.7 (44474.7-54190.2) | 192.3 (176-211.7) | 0.41 (-2.37-3.26) |
| CKD due to other and unspecified causes | Puerto Rico | 6687.6 (6093.8-7352.9) | 185.3 (168.6-203.9) | 17164.7 (15727.8-18722) | 287.7 (267.4-313) | 1.37 (-1.28-4.09) |
| CKD due to other and unspecified causes | Qatar | 370.3 (327.6-416.6) | 236 (213.4-261.1) | 5029.1 (4490.4-5627.4) | 374.5 (347.9-405.9) | 1.12 (-1.72-4.03) |
| CKD due to other and unspecified causes | Republic of Korea | 49503.3 (44333.4-55626.6) | 174.6 (157.8-194.5) | 184048 (170698.5-196208.4) | 196.9 (183.4-209.6) | 0.3 (-2.46-3.14) |
| CKD due to other and unspecified causes | Republic of Moldova | 2411.3 (2159-2673.8) | 55.8 (50.3-61.5) | 5369.6 (4792.9-6043.5) | 100.5 (91-112.1) | 1.37 (-0.87-3.65) |
| CKD due to other and unspecified causes | Romania | 24856.6 (22258.7-27738.1) | 91.2 (82.3-101.5) | 59173.4 (54617.1-64138) | 170.9 (158.9-184.3) | 1.62 (-0.88-4.19) |
| CKD due to other and unspecified causes | Russian Federation | 163559.4 (149070.1-180858.7) | 97.1 (90-105.9) | 345853.5 (316229.1-376277.3) | 164 (151.9-176.4) | 1.32 (-0.93-3.61) |
| CKD due to other and unspecified causes | Rwanda | 2756.6 (2336.5-3131.4) | 76.9 (69.8-84.2) | 6861.5 (6120-7552.7) | 97.5 (88.1-106.7) | 0.56 (-1.7-2.86) |
| CKD due to other and unspecified causes | Saint Kitts and Nevis | 65.3 (58.8-72.3) | 180.9 (165.1-200.1) | 205.7 (185.7-228.3) | 278.8 (257-303.9) | 1.35 (-1.27-4.04) |
| CKD due to other and unspecified causes | Saint Lucia | 152.5 (139.1-165.8) | 162.4 (149.1-175.7) | 600.9 (553.8-659.4) | 255.2 (235.8-277.2) | 1.26 (-1.35-3.94) |
| CKD due to other and unspecified causes | Saint Vincent and the Grenadines | 116.3 (104.4-127.2) | 153.2 (140-167.3) | 334.1 (304-362.6) | 238.5 (218.6-257.1) | 1.29 (-1.26-3.9) |
| CKD due to other and unspecified causes | Samoa | 156 (141-171.4) | 153.8 (141.8-167.5) | 349.8 (321.1-382.1) | 219 (202.8-238.7) | 0.84 (-1.73-3.48) |
| CKD due to other and unspecified causes | San Marino | 57.4 (51.3-64.5) | 158.6 (142.2-177.3) | 128.3 (115-142.5) | 168.7 (151.2-186.5) | 0.25 (-2.43-3.01) |
| CKD due to other and unspecified causes | Sao Tome and Principe | 101.7 (87.9-114.5) | 131.6 (117.7-145.4) | 261.8 (236.1-287.7) | 194.9 (177.9-211.3) | 1.07 (-1.34-3.55) |
| CKD due to other and unspecified causes | Saudi Arabia | 16952.7 (15498.5-18417.9) | 239.2 (219.2-258.8) | 101171.1 (92443.4-110466.6) | 400.7 (375-427.4) | 1.46 (-1.34-4.33) |
| CKD due to other and unspecified causes | Senegal | 4515.5 (3957.1-5080.5) | 108.9 (100.3-118.7) | 12577.5 (11320.9-13856.2) | 137.9 (127.2-150.2) | 0.48 (-1.9-2.92) |
| CKD due to other and unspecified causes | Serbia | 12596.6 (11317.6-13944.5) | 108.8 (98.8-119.3) | 31746.8 (28978.6-34932) | 199.7 (183.5-217.7) | 1.76 (-0.86-4.46) |
| CKD due to other and unspecified causes | Seychelles | 102.3 (93.3-112.2) | 176.3 (161-193.4) | 309 (280.5-340.5) | 256.3 (235.6-280.2) | 0.98 (-1.71-3.74) |
| CKD due to other and unspecified causes | Sierra Leone | 2577.2 (2251.3-2899.9) | 101.3 (91.9-111.7) | 6234.9 (5519.3-6914.4) | 131.4 (119.3-142.9) | 0.62 (-1.71-3.01) |
| CKD due to other and unspecified causes | Singapore | 4518.8 (4286.9-4756) | 204.8 (194-216.1) | 20490.6 (19043.6-22337.3) | 241.8 (224.7-263.1) | 0.5 (-2.28-3.37) |
| CKD due to other and unspecified causes | Slovakia | 6666.7 (6003.1-7414.1) | 113.5 (103-126.5) | 16875.5 (15337.1-18515.4) | 183.3 (168.3-199.2) | 1.24 (-1.26-3.8) |
| CKD due to other and unspecified causes | Slovenia | 2436.1 (2191.8-2715.9) | 101.7 (91.5-112.8) | 7468.9 (6762.7-8278.7) | 178.5 (162.5-196.6) | 1.53 (-1.04-4.17) |
| CKD due to other and unspecified causes | Solomon Islands | 277.8 (244.6-306.3) | 132.1 (121.1-144) | 671.5 (604.9-731.8) | 146 (133.4-157.9) | 0.02 (-2.34-2.45) |
| CKD due to other and unspecified causes | Somalia | 2595.5 (2168.1-2955.9) | 77 (70.5-84.2) | 7833 (6586.6-8852.7) | 91.1 (82.9-98.8) | 0.33 (-1.9-2.61) |
| CKD due to other and unspecified causes | South Africa | 32254.5 (29716.4-35051.2) | 139.6 (128.6-151.9) | 94837.7 (87730-101159.3) | 196.6 (182.1-209.6) | 0.77 (-1.75-3.35) |
| CKD due to other and unspecified causes | South Sudan | 2386.3 (2098.9-2676) | 77.8 (71-85.2) | 4512.7 (3972.3-5076.9) | 96.1 (87.6-105.3) | 0.46 (-1.78-2.76) |
| CKD due to other and unspecified causes | Spain | 107732.9 (98833.3-118607.1) | 193.3 (178.6-209.9) | 192460.4 (175769.4-208709.1) | 189 (172.5-204.7) | 0.07 (-2.74-2.97) |
| CKD due to other and unspecified causes | Sri Lanka | 17660.1 (16116.8-19284.6) | 150.3 (137.5-163.6) | 62808.1 (57064.5-68959.2) | 228.2 (209.2-248.2) | 1.05 (-1.57-3.74) |
| CKD due to other and unspecified causes | Sudan | 18242.4 (16256.7-20484.7) | 175.7 (158.3-195.8) | 58819.9 (53574.2-64325.7) | 270.6 (246.7-297.6) | 1.12 (-1.59-3.91) |
| CKD due to other and unspecified causes | Suriname | 432.9 (393.2-482.2) | 151.9 (138.4-168.3) | 1575.1 (1429.9-1721.5) | 245.5 (223.8-266.9) | 1.43 (-1.12-4.05) |
| CKD due to other and unspecified causes | Sweden | 23591.1 (21195.2-26748.2) | 147 (133.3-163.9) | 31288.4 (27869.5-35053.1) | 139 (125.9-153.6) | 0.1 (-2.55-2.82) |
| CKD due to other and unspecified causes | Switzerland | 20757.5 (18908.7-22835.4) | 193.3 (177.2-210.7) | 38566.5 (34925.7-41877.9) | 208.6 (189.1-226.7) | 0.3 (-2.51-3.19) |
| CKD due to other and unspecified causes | Syrian Arab Republic | 13506.2 (12059.7-15064.2) | 218.3 (199.4-239.9) | 46147.5 (42562.1-50291.3) | 331.2 (307.1-356.4) | 1.02 (-1.68-3.8) |
| CKD due to other and unspecified causes | Taiwan (Province of China) | 33091.4 (30645.2-35632.3) | 203.6 (189.5-218.7) | 100749.8 (93852.1-106826.1) | 244.6 (229.3-258.4) | 0.53 (-2.18-3.32) |
| CKD due to other and unspecified causes | Tajikistan | 2380.4 (2039-2710.5) | 64 (56.9-71.6) | 7421.1 (6552.3-8250.1) | 103.3 (93.1-114.1) | 1.43 (-0.77-3.67) |
| CKD due to other and unspecified causes | Thailand | 67189.3 (60594.7-73992.7) | 169.3 (154.1-185.5) | 258667.2 (240086.9-279783.8) | 244.4 (228.3-263.6) | 0.76 (-1.82-3.41) |
| CKD due to other and unspecified causes | Timor-Leste | 461.9 (405-521.7) | 120.3 (109.2-133.2) | 1547.9 (1407.3-1701) | 164.4 (150-179.5) | 0.82 (-1.64-3.33) |
| CKD due to other and unspecified causes | Togo | 1900.3 (1630.4-2141) | 110.4 (101-120.5) | 6616.1 (5951.8-7289.9) | 143.1 (129.5-156.3) | 0.52 (-1.85-2.94) |
| CKD due to other and unspecified causes | Tokelau | 1.7 (1.5-1.9) | 126.6 (115.4-138.1) | 2.9 (2.7-3.2) | 202.1 (185.8-220.1) | 1.15 (-1.4-3.77) |
| CKD due to other and unspecified causes | Tonga | 95.2 (86.2-106.3) | 149.3 (136.4-163.8) | 182.6 (167.5-198.7) | 214.4 (197.1-232.4) | 0.93 (-1.64-3.56) |
| CKD due to other and unspecified causes | Trinidad and Tobago | 1337.7 (1224.2-1461.2) | 148 (136-161.4) | 4699.7 (4308.6-5117.7) | 250.1 (231.1-269.2) | 1.54 (-1.04-4.18) |
| CKD due to other and unspecified causes | Tunisia | 10782 (9724.9-11986) | 202 (184-221.8) | 44269.4 (40240.7-48221.4) | 327.2 (300.6-354.8) | 1.17 (-1.65-4.08) |
| CKD due to other and unspecified causes | Turkey | 65048.1 (60087.9-70415.9) | 181 (167.3-194) | 312709.2 (287132.1-337017.3) | 328.1 (303.2-351) | 1.52 (-1.24-4.37) |
| CKD due to other and unspecified causes | Turkmenistan | 2491.9 (2171.2-2781.8) | 93.5 (84.8-103.5) | 6798.1 (6056.1-7411.4) | 144.9 (131.2-156.9) | 1.39 (-0.84-3.67) |
| CKD due to other and unspecified causes | Tuvalu | 9 (8.1-10) | 120.9 (110.8-134) | 21.3 (19.3-23.6) | 194.6 (178-212.3) | 1.19 (-1.3-3.75) |
| CKD due to other and unspecified causes | Uganda | 5611.7 (4755-6370.7) | 69.5 (63-75.8) | 15306.5 (13321.3-17078.4) | 85.9 (78.4-94.1) | 0.42 (-1.82-2.72) |
| CKD due to other and unspecified causes | Ukraine | 43009.9 (38050.1-47981) | 65.6 (58.4-72.8) | 67202.3 (61344.3-74018.1) | 100.2 (92.1-109.3) | 1.16 (-1.05-3.43) |
| CKD due to other and unspecified causes | United Arab Emirates | 1712.9 (1544.1-1905.2) | 263.4 (241.1-288.1) | 26090.8 (23029.9-29650.1) | 371.7 (344.6-402.9) | 0.99 (-1.74-3.8) |
| CKD due to other and unspecified causes | United Kingdom | 159360.8 (145071.9-177537.5) | 169.6 (155.1-186.3) | 235749.3 (215917.6-256911.9) | 177.5 (163-191.7) | 0.02 (-2.63-2.75) |
| CKD due to other and unspecified causes | United Republic of Tanzania | 11039.7 (9557.6-12450.3) | 83.5 (75.8-91.1) | 30052.4 (26738.9-33218.2) | 102.9 (94.2-111.9) | 0.44 (-1.82-2.76) |
| CKD due to other and unspecified causes | United States of America | 783441.6 (716897.6-857964.5) | 245.6 (224.9-268) | 1493906.4 (1378712.1-1604365.6) | 260.9 (242.6-279.2) | 0.06 (-2.57-2.77) |
| CKD due to other and unspecified causes | United States Virgin Islands | 150.6 (137.2-164.3) | 163 (149.7-177.3) | 416.7 (378.8-457.4) | 256.1 (235-280.1) | 1.25 (-1.37-3.94) |
| CKD due to other and unspecified causes | Uruguay | 6242 (5642.1-6874.7) | 159 (144.3-174.4) | 10763.1 (9783.8-11703.5) | 192.4 (176-207.6) | 0.59 (-2.01-3.25) |
| CKD due to other and unspecified causes | Uzbekistan | 17491.3 (15553.5-19368.4) | 117 (106.1-128.9) | 50659.2 (45066.4-56103.1) | 166.6 (150.2-182.3) | 1.1 (-1.2-3.45) |
| CKD due to other and unspecified causes | Vanuatu | 97.4 (86.4-108) | 116.7 (106.9-127.4) | 361.8 (327.4-392.8) | 174.8 (160.9-188.7) | 0.94 (-1.54-3.48) |
| CKD due to other and unspecified causes | Venezuela (Bolivarian Republic of) | 22177.4 (20004.5-24464) | 199.4 (180.7-220.9) | 92772.6 (85899.6-99556.5) | 301.9 (280.4-322.8) | 1.21 (-1.52-4.02) |
| CKD due to other and unspecified causes | Viet Nam | 48933.5 (43760.3-54099.6) | 111.3 (101-123) | 175944 (158900.2-195031.9) | 174.9 (159.5-192.2) | 1.36 (-1.13-3.91) |
| CKD due to other and unspecified causes | Yemen | 9990.7 (8852.7-11324.8) | 173.3 (156.1-192.4) | 38952.9 (35329.9-42694.4) | 246.6 (224.1-269.7) | 0.98 (-1.7-3.73) |
| CKD due to other and unspecified causes | Zambia | 3283.2 (2736-3737.1) | 86.7 (79-95.4) | 9574.2 (8494.7-10555) | 110.5 (100.6-120.5) | 0.5 (-1.8-2.86) |
| CKD due to other and unspecified causes | Zimbabwe | 6167.9 (5515.2-6834.4) | 127.9 (116.7-141.2) | 12405.2 (11222.5-13594.6) | 153.8 (140.7-167.6) | 0.19 (-2.27-2.72) |

Table S2. The number of CKD deaths and age standardized mortality of CKD caused by different reasons from 1990 to 2021.

| Cause | Location | 1990 | | 2021 | | 1990-2021 |
| --- | --- | --- | --- | --- | --- | --- |
|  |  | Number of deaths  No. ×10^3^ (95% UI) | ASMR per 100,000  No. (95% UI) | Number of deaths  No. ×10^3^ (95% UI) | ASMR per 100,000  No. (95% UI) | EAPC  No. (95% CI) |
| CKD | Afghanistan | 3009 (2175-4674.9) | 47.3 (34.5-75.9) | 4892.6 (2926.3-8625.5) | 53.9 (32.9-102.2) | 0.36 (-1.22-1.96) |
| CKD | Albania | 234 (196.1-277.1) | 12 (10-14.3) | 424.3 (321.7-540.6) | 10.6 (8.1-13.4) | -0.11 (-1.3-1.09) |
| CKD | Algeria | 2344.4 (1666.5-4128.5) | 27.1 (19-47.7) | 9784.9 (7909.3-12092.3) | 37.3 (30.6-45.5) | 1.13 (-0.42-2.7) |
| CKD | American Samoa | 6.8 (4.9-9.7) | 33.5 (24.6-48.1) | 31.3 (24.3-39.1) | 73.8 (57.4-91.7) | 2.12 (0.13-4.16) |
| CKD | Andorra | 5.4 (3.9-7.4) | 12.1 (8.8-16.7) | 16.8 (12.3-22.5) | 9.3 (6.9-12.5) | -0.33 (-1.44-0.79) |
| CKD | Angola | 1416.5 (1092.7-1792.8) | 37.6 (29.3-46.9) | 4073.1 (2988.1-5383.6) | 39.2 (27.9-50.3) | -0.01 (-1.29-1.29) |
| CKD | Antigua and Barbuda | 16.8 (15.5-18) | 30.1 (27.9-32.2) | 46 (41.8-49.4) | 47.1 (43-50.5) | 1.66 (0.11-3.24) |
| CKD | Argentina | 9185.4 (8697.1-9648.2) | 30.4 (28.6-31.9) | 15212.8 (13850.7-16266.4) | 26.3 (24-28.1) | -0.28 (-1.65-1.11) |
| CKD | Armenia | 26.6 (22.2-31.1) | 1 (0.8-1.1) | 432.4 (354.3-523.1) | 10.3 (8.4-12.4) | 3.05 (1.96-4.16) |
| CKD | Australia | 1580.2 (1435.6-1674.2) | 8.7 (7.9-9.3) | 5011.1 (4200.6-5514.9) | 9.4 (8-10.3) | 0.64 (-0.47-1.77) |
| CKD | Austria | 786.4 (724.2-834.3) | 6.6 (6.1-7) | 3410.9 (2801.2-3758.5) | 14.3 (11.9-15.6) | 2.48 (1.37-3.61) |
| CKD | Azerbaijan | 357.5 (276.8-451.6) | 6.6 (5.1-8.3) | 991.6 (716.8-1294.7) | 10.4 (7.6-13.4) | 0.95 (-0.28-2.2) |
| CKD | Bahamas | 36.7 (33.9-39.9) | 23.1 (21.3-25.1) | 144 (117.1-178.5) | 37.5 (30.7-46.2) | 1.65 (0.24-3.08) |
| CKD | Bahrain | 47.7 (35.9-63.4) | 41.7 (31.3-55.7) | 258.6 (206.6-315.9) | 52.5 (42.1-63.4) | 0.24 (-1.54-2.04) |
| CKD | Bangladesh | 9553 (6571.8-11509.6) | 14.9 (12.8-18.3) | 15175.2 (11740.3-20173) | 12.3 (9.5-16.2) | -0.46 (-1.43-0.52) |
| CKD | Barbados | 60.4 (56.7-63.6) | 20.4 (19.1-21.4) | 148.1 (117.4-181.7) | 29.3 (23.3-36) | 1.28 (-0.15-2.73) |
| CKD | Belarus | 99.7 (93.4-106.2) | 0.8 (0.8-0.9) | 359.5 (296-427.2) | 2.4 (1.9-2.8) | 0.89 (0.42-1.36) |
| CKD | Belgium | 1386.1 (1260-1478) | 8.8 (8-9.4) | 2815.5 (2274.4-3150.5) | 9 (7.5-10) | 0.39 (-0.7-1.49) |
| CKD | Belize | 24.5 (22.9-26) | 24.4 (22.8-26) | 133.8 (116.8-150.6) | 45.5 (39.5-51.1) | 2.06 (0.57-3.56) |
| CKD | Benin | 782.5 (673.3-912.2) | 37.3 (32.3-44.7) | 1939.7 (1558.4-2414) | 39.4 (32.5-48.4) | 0.07 (-1.35-1.51) |
| CKD | Bermuda | 9.2 (8.6-9.8) | 15.5 (14.5-16.5) | 22.5 (18.9-27.4) | 15.7 (13.2-19) | 0.27 (-1-1.56) |
| CKD | Bhutan | 41.8 (29.6-58.5) | 17 (12.4-24.2) | 120.4 (80.6-164.9) | 20.9 (14-28.5) | 0.51 (-0.52-1.54) |
| CKD | Bolivia (Plurinational State of) | 1325.3 (1115.2-1674.8) | 43 (36.2-53.9) | 4730.7 (3674.6-6139.9) | 58.2 (45.7-75.3) | 0.81 (-0.57-2.21) |
| CKD | Bosnia and Herzegovina | 360.6 (307.4-421.5) | 9.8 (8.3-11.4) | 685.5 (512.2-869.5) | 11 (8.3-13.9) | 0.25 (-0.8-1.32) |
| CKD | Botswana | 109.5 (79.1-156.9) | 23.1 (17-32.1) | 347.8 (269.6-478.6) | 28.5 (23-39) | 0.61 (-0.72-1.96) |
| CKD | Brazil | 15168.4 (14504.7-15698.9) | 18 (16.9-18.8) | 45536.9 (41270.8-47987.2) | 18.7 (16.9-19.7) | 0.03 (-1.14-1.21) |
| CKD | Brunei Darussalam | 32.4 (26.2-41.9) | 37.5 (30.7-47.8) | 86 (72.8-100.4) | 36.3 (30.6-42.3) | 0.39 (-0.97-1.77) |
| CKD | Bulgaria | 729.4 (668-785.4) | 7.6 (7-8.2) | 2427.7 (2049.1-2872.4) | 17.4 (14.6-20.8) | 2.31 (1.12-3.51) |
| CKD | Burkina Faso | 1555.3 (1280.9-1850.2) | 38.3 (31.8-45.8) | 3812.2 (3071.3-4632.9) | 43.7 (35.3-53) | 0.35 (-1.12-1.85) |
| CKD | Burundi | 908.6 (710.6-1147.8) | 39.5 (31-50.6) | 1514.5 (1109-2172.4) | 35 (25.8-49.9) | -0.71 (-1.9-0.5) |
| CKD | Cabo Verde | 37.8 (31.7-44.1) | 15.4 (12.7-17.9) | 123.8 (84-155) | 28.9 (19.4-36) | 1.32 (0.1-2.56) |
| CKD | Cambodia | 1049.9 (857.2-1277.4) | 21.2 (17.5-25.7) | 2681.4 (2007.5-3484.1) | 23.5 (17.8-30.2) | 0.02 (-1.47-1.53) |
| CKD | Cameroon | 2151 (1676.6-2796.6) | 52 (40-67.1) | 5927.7 (4179-8334.3) | 51.2 (37.1-71.1) | 0 (-1.39-1.42) |
| CKD | Canada | 2118.8 (1921.2-2236.5) | 6.8 (6.1-7.2) | 7789.1 (6587.9-8472.6) | 9.5 (8.2-10.3) | 0.85 (-0.23-1.94) |
| CKD | Central African Republic | 507.3 (419.7-615) | 47 (38.6-56.4) | 963.5 (698.3-1352.3) | 47.4 (35.4-63.9) | 0.03 (-1.24-1.3) |
| CKD | Chad | 750.9 (593.4-1012.6) | 26.4 (20.5-36.9) | 1810.8 (1330.1-2568.3) | 31.6 (23.4-45.6) | 0.35 (-0.98-1.69) |
| CKD | Chile | 1471.4 (1390.4-1543.5) | 16 (15-16.9) | 5201.8 (4581.6-5596.4) | 19.8 (17.5-21.3) | 0.78 (-0.52-2.1) |
| CKD | China | 102725.5 (89744.9-120908.2) | 14.2 (12.4-16.8) | 204230.2 (164736.1-246372.3) | 10.8 (8.8-13) | -0.72 (-2-0.58) |
| CKD | Colombia | 3340.2 (3176.7-3470.8) | 19.5 (18.4-20.4) | 8646.1 (7184.2-10115.7) | 15.5 (12.9-18.1) | -0.42 (-1.5-0.67) |
| CKD | Comoros | 70.5 (54.3-88) | 39 (30.8-48) | 181.5 (131.7-234.7) | 42.3 (31-54.4) | 0.04 (-1.21-1.3) |
| CKD | Congo | 531.1 (405.8-636.8) | 55.8 (41-67.2) | 1252.5 (846.8-1625.9) | 54.8 (35.6-69) | -0.26 (-1.56-1.06) |
| CKD | Cook Islands | 2 (1.5-2.6) | 17.5 (13.8-23.2) | 5.4 (4.1-6.7) | 22 (16.6-27.2) | 0.51 (-1.07-2.1) |
| CKD | Costa Rica | 263.6 (247.4-278.6) | 15.3 (14.2-16.2) | 1605.5 (1391.3-1794.1) | 29 (25.2-32.3) | 1.71 (0.57-2.86) |
| CKD | C么te d'Ivoire | 1454.9 (1170.5-1786.8) | 38.9 (32-47.7) | 4059.8 (3151-5240) | 40.5 (33-51.2) | -0.03 (-1.6-1.56) |
| CKD | Croatia | 501.7 (464.2-540) | 9.5 (8.8-10.2) | 1336.5 (1157.7-1549.4) | 13.5 (11.6-15.7) | 0.64 (-0.48-1.79) |
| CKD | Cuba | 830.9 (789-866.1) | 8.3 (7.9-8.7) | 3208.7 (2758.1-3637.9) | 16.1 (13.9-18.3) | 1.64 (0.47-2.83) |
| CKD | Cyprus | 188.3 (156.1-238.4) | 41.3 (34.1-51.5) | 342.6 (284-402.8) | 21.5 (18.1-25) | -1.78 (-3.25--0.28) |
| CKD | Czechia | 1096.2 (999.4-1200) | 8.2 (7.5-8.9) | 1561.8 (1326.1-1820.1) | 6.8 (5.8-8) | -0.28 (-1.25-0.7) |
| CKD | Democratic People's Republic of Korea | 2021.4 (1438.5-2685.1) | 14 (10.1-19.3) | 4298.1 (3284.5-5595.8) | 14 (10.7-18.3) | 0.03 (-1.31-1.39) |
| CKD | Democratic Republic of the Congo | 5867.5 (4670.8-7377.2) | 42.3 (34.5-52) | 13845.2 (9965.5-18759) | 42.9 (31.4-57.7) | -0.04 (-1.29-1.23) |
| CKD | Denmark | 413.3 (379.3-436.8) | 4.8 (4.5-5.1) | 1699.3 (1457.3-1892.7) | 12.1 (10.5-13.5) | 1.76 (0.73-2.8) |
| CKD | Djibouti | 35.3 (26.2-46.7) | 29.3 (22.4-37.7) | 210.9 (152.4-284.9) | 41.8 (31.4-54.7) | 0.96 (-0.27-2.19) |
| CKD | Dominica | 19 (16.5-21.3) | 32.4 (28.2-36.3) | 39.6 (31.7-48.8) | 50.3 (40.5-61.8) | 1.41 (-0.14-2.97) |
| CKD | Dominican Republic | 621 (533-749.6) | 16.7 (14.3-20.3) | 2392.3 (1645.8-3063.7) | 24 (16.5-30.7) | 1.45 (0.16-2.76) |
| CKD | Ecuador | 1227.3 (1150.4-1287.5) | 23.7 (22.1-25) | 6502.3 (4898.3-8821.8) | 42.1 (32.2-56) | 1.51 (0.09-2.95) |
| CKD | Egypt | 10610.2 (8477.7-16104.3) | 51.8 (40.3-82.6) | 33311.3 (27183.1-40816.3) | 71.7 (59.7-86.6) | 1.2 (-0.56-3) |
| CKD | El Salvador | 797 (702.3-1141.5) | 25.3 (22.1-36.3) | 4441 (3314-5504.4) | 69.8 (52.2-86.8) | 3.01 (1.78-4.26) |
| CKD | Equatorial Guinea | 79.1 (61.9-97.6) | 43.8 (35-53.4) | 255 (156-372.3) | 54.7 (31.3-76.5) | 0.84 (-0.46-2.16) |
| CKD | Eritrea | 357.8 (266.5-497.7) | 32.6 (24.7-45.5) | 901.8 (609.6-1461.7) | 38.1 (26.6-60.6) | 0.39 (-0.79-1.58) |
| CKD | Estonia | 120 (112.2-127.4) | 6.4 (6-6.8) | 526.6 (437.3-605.4) | 17 (14.2-19.7) | 1.82 (0.87-2.77) |
| CKD | Eswatini | 100 (78.7-124.9) | 38.7 (30.5-48.3) | 274 (173.7-382.8) | 53.5 (36.5-71.6) | 1.27 (-0.14-2.7) |
| CKD | Ethiopia | 12911.9 (10097.2-14949.2) | 69 (57-79.6) | 16917.6 (14059.5-20068) | 42.4 (34.8-50.2) | -1.95 (-3.2--0.69) |
| CKD | Fiji | 94.6 (66.7-135.9) | 27.7 (19.6-40.5) | 313.8 (231.1-410.3) | 48 (36.4-61.9) | 0.97 (-0.82-2.78) |
| CKD | Finland | 213.8 (194.2-227.3) | 3 (2.7-3.2) | 818.4 (656.6-926.7) | 4.9 (4-5.5) | 0.89 (0.12-1.68) |
| CKD | France | 6887.5 (6157-7354.8) | 7.7 (6.9-8.3) | 15132.9 (12606.4-16843.3) | 7.3 (6.2-8.1) | 0.15 (-0.84-1.16) |
| CKD | Gabon | 265.3 (212.4-318) | 51.1 (40.7-61.6) | 621.3 (324.4-821.5) | 71.5 (35.5-93) | 0.93 (-0.43-2.3) |
| CKD | Gambia | 115.4 (90.1-143.2) | 33.7 (26.8-41.3) | 402.8 (305.1-513.5) | 43.2 (33.2-54.7) | 0.53 (-0.87-1.94) |
| CKD | Georgia | 197.2 (160.6-246.5) | 3.3 (2.7-4.1) | 712.7 (574.4-897.6) | 12.3 (9.9-15.5) | 2.41 (1.21-3.64) |
| CKD | Germany | 11051.3 (10128.4-11738.8) | 8.3 (7.6-8.8) | 39563.2 (31943.4-45262.2) | 15.4 (12.6-17.4) | 2 (0.83-3.19) |
| CKD | Ghana | 1744.9 (1343.4-2489.7) | 29.5 (22.8-41.8) | 7075.9 (5563.4-8853.6) | 47.7 (37.9-59.3) | 1.59 (0.23-2.97) |
| CKD | Greece | 2985.3 (2744.4-3173.9) | 21.3 (19.4-22.7) | 7052.8 (6076.5-7641.4) | 21.3 (18.7-23) | -0.45 (-1.79-0.9) |
| CKD | Greenland | 3.2 (2.7-3.7) | 13.1 (11-15.3) | 7.6 (5.6-9.6) | 14.8 (10.7-18.6) | 0.58 (-0.65-1.82) |
| CKD | Grenada | 26.7 (24.2-29.2) | 35.7 (32.5-39.2) | 61.7 (53.7-69.1) | 58.3 (51-64.9) | 1.63 (0.08-3.22) |
| CKD | Guam | 14.1 (12.4-17.5) | 22.9 (20-28.3) | 50.6 (42.4-57.6) | 24 (20.4-27.2) | 0.92 (-0.51-2.37) |
| CKD | Guatemala | 1108.1 (1062.8-1158.3) | 33.7 (32-35.3) | 5448 (4532.5-6387) | 50.7 (42.3-59.3) | 1.94 (0.65-3.24) |
| CKD | Guinea | 1055.3 (849.7-1342.4) | 32.3 (25.5-41.8) | 1874.4 (1413.8-2581.6) | 34.5 (25.9-47.8) | 0.15 (-1.34-1.67) |
| CKD | Guinea-Bissau | 201.1 (157.4-246.2) | 50.1 (40.3-60.7) | 315.6 (245.8-404.3) | 46.4 (37-58.9) | -0.3 (-1.69-1.11) |
| CKD | Guyana | 116.9 (105.6-128.3) | 30.5 (27.7-33.4) | 349.4 (269.3-445.6) | 56.5 (43.8-71.2) | 2.57 (1.09-4.07) |
| CKD | Haiti | 961.6 (707.4-1555.3) | 28.7 (20.5-48.9) | 2177 (1206.7-4586.7) | 30.9 (17.2-64.8) | 0.34 (-1.01-1.7) |
| CKD | Honduras | 259.5 (214.9-328.5) | 12.3 (10.1-16) | 1439 (1077.4-1888.9) | 24.6 (18.8-31.7) | 1.9 (0.81-3) |
| CKD | Hungary | 841.7 (794.9-887.7) | 6.3 (5.9-6.6) | 2172.8 (1852.3-2483) | 10.1 (8.7-11.6) | 1.37 (0.32-2.43) |
| CKD | Iceland | 11.1 (9.9-12) | 3.6 (3.2-3.9) | 40.8 (32.7-45.7) | 5.8 (4.7-6.5) | 0.97 (0.14-1.8) |
| CKD | India | 58474.6 (50679.4-67535.5) | 13.1 (11.2-15.2) | 175637.1 (146072-208714.6) | 15.8 (13.1-18.7) | 0.37 (-0.59-1.34) |
| CKD | Indonesia | 18489.6 (15846.2-22279.9) | 17.3 (14.9-21.8) | 51165.5 (41552.8-63407.7) | 23 (18.8-29) | 0.58 (-0.87-2.05) |
| CKD | Iran (Islamic Republic of) | 3717.6 (2815.8-5016.6) | 17.8 (13-24.7) | 13626.7 (11261.8-15175) | 20.2 (16.5-22.6) | 0.34 (-1.09-1.79) |
| CKD | Iraq | 2947.7 (2325-3774.9) | 36.7 (28.7-47.6) | 8278.6 (5634.7-10643.7) | 43.4 (30.5-54.8) | 0.09 (-1.4-1.6) |
| CKD | Ireland | 310.5 (287.7-329.7) | 8.2 (7.5-8.7) | 703.2 (587.3-792.3) | 8.2 (6.9-9.2) | 0.4 (-0.62-1.43) |
| CKD | Israel | 970.3 (893.4-1036.5) | 22.2 (20.2-23.8) | 2789.8 (2313.8-3075.4) | 19.7 (16.5-21.7) | 0.26 (-1.06-1.6) |
| CKD | Italy | 7527.7 (6774-7922.1) | 8.8 (7.8-9.2) | 19086.4 (15313.6-21515.3) | 9.3 (7.6-10.5) | 0.08 (-0.95-1.11) |
| CKD | Jamaica | 434.5 (407.6-457.5) | 23.3 (21.9-24.6) | 935.3 (707.2-1205.2) | 29.2 (22-37.5) | 0.28 (-1.14-1.72) |
| CKD | Japan | 17679.2 (16010.1-18556.5) | 11.6 (10.4-12.3) | 52716.6 (41037.9-59205.3) | 9.5 (7.8-10.4) | -0.47 (-1.74-0.82) |
| CKD | Jordan | 360.5 (292.6-469.8) | 31.6 (25.6-41.5) | 1875.8 (1491.5-2406.4) | 33.1 (26.2-41.8) | -0.08 (-1.49-1.35) |
| CKD | Kazakhstan | 737.2 (644-832.4) | 5.3 (4.6-6) | 1981.6 (1630.8-2335.3) | 12.2 (10.1-14.4) | 1.27 (0.02-2.53) |
| CKD | Kenya | 1949.7 (1547.7-3039.3) | 25.2 (19.6-40.8) | 7330.5 (5884-9859.6) | 37.6 (29.8-49.5) | 1.29 (0.09-2.5) |
| CKD | Kiribati | 10.9 (8.9-13.3) | 30 (24.2-36.8) | 28.6 (18.4-42.3) | 43.1 (28-62.3) | 0.66 (-1.1-2.45) |
| CKD | Kuwait | 160 (144.1-177.6) | 30.7 (26.6-34.3) | 373.9 (275.9-459.2) | 16 (11.7-19.7) | -1.59 (-2.96--0.19) |
| CKD | Kyrgyzstan | 158.8 (141.1-179.8) | 4.6 (4-5.2) | 497.8 (414.6-596.4) | 10 (8.3-11.9) | 0.63 (-0.66-1.94) |
| CKD | Lao People's Democratic Republic | 917.1 (677.4-1191) | 43.9 (33.2-56.9) | 1774.6 (1241.9-2407.8) | 40.9 (29.1-54.7) | -0.44 (-2.18-1.33) |
| CKD | Latvia | 95 (89.1-100.9) | 2.9 (2.7-3.1) | 321.5 (265.3-391.8) | 7.7 (6.4-9.5) | 1.6 (0.78-2.43) |
| CKD | Lebanon | 592.2 (455.2-790.4) | 32.3 (24.7-42.7) | 2102.5 (1733.1-2533.5) | 31.9 (26.3-38.5) | 0.05 (-1.5-1.62) |
| CKD | Lesotho | 153 (119-201.9) | 19.9 (15.3-26.5) | 444.8 (311.5-595.2) | 46.2 (32.8-59.4) | 2.97 (1.49-4.47) |
| CKD | Liberia | 533.3 (426.7-673.2) | 46.4 (38-57.8) | 953.6 (705.9-1263.3) | 48.4 (36.3-63.3) | 0.31 (-1.13-1.77) |
| CKD | Libya | 502.7 (373.8-703.6) | 28.1 (20.5-40.1) | 2126.8 (1413.2-2796.1) | 46.9 (31.3-61.1) | 1.91 (0.31-3.53) |
| CKD | Lithuania | 103.5 (97.2-109.1) | 2.5 (2.3-2.6) | 332.1 (280.5-400.3) | 5.8 (4.9-7.1) | 0.81 (0.05-1.57) |
| CKD | Luxembourg | 52.2 (48.5-55.4) | 10.3 (9.5-10.9) | 158.4 (130.3-183.6) | 12.6 (10.5-14.5) | 0.67 (-0.49-1.85) |
| CKD | Madagascar | 1300.4 (1031-1793.3) | 26.7 (21-38.2) | 2660 (1941.2-3524.3) | 27 (19.7-35.4) | 0.06 (-1.08-1.21) |
| CKD | Malawi | 1437.6 (1169.6-1735.9) | 38.7 (31.2-49) | 3141 (2543.3-3817.6) | 45.4 (37.8-54.5) | 0.32 (-0.92-1.57) |
| CKD | Malaysia | 2075.1 (1794.7-2375.9) | 22.6 (19.4-25.9) | 7574.5 (6351.8-8581.4) | 29.2 (24.4-33) | 0.37 (-1.36-2.12) |
| CKD | Maldives | 40 (33.6-50.8) | 48.4 (39.7-69.3) | 87.8 (71.1-105.4) | 28.9 (23.4-34.5) | -1.64 (-3.39-0.15) |
| CKD | Mali | 1405.1 (1152.7-1734) | 38.3 (31.3-47.7) | 3001.4 (2407.4-3921.8) | 37.4 (30.3-48.1) | 0 (-1.39-1.41) |
| CKD | Malta | 45.7 (41.8-48.6) | 12.1 (10.9-12.9) | 141 (116.1-160.1) | 12.5 (10.4-14.1) | 0.13 (-1.02-1.3) |
| CKD | Marshall Islands | 5.3 (3.3-9.4) | 32.5 (20.2-58.7) | 17.5 (5.5-49.1) | 54.8 (17.4-160.8) | 1.32 (-0.48-3.16) |
| CKD | Mauritania | 393.8 (305.3-501.6) | 42.6 (33.2-54.3) | 774.3 (532.2-1095.1) | 40.4 (27.5-56.5) | -0.46 (-1.86-0.96) |
| CKD | Mauritius | 302 (287.7-315) | 44.3 (42-46.4) | 1413.5 (1303.9-1494.1) | 80.1 (74.1-84.6) | 1.7 (-0.29-3.73) |
| CKD | Mexico | 14133.2 (13700.7-14565) | 36.9 (35.4-38.4) | 67043.9 (58757.4-76982.6) | 54.3 (47.8-61.9) | 1.74 (0.49-3.01) |
| CKD | Micronesia (Federated States of) | 15.8 (10.7-24.8) | 33.5 (22.8-54.5) | 37.8 (27-53.3) | 57.6 (42.1-80.9) | 1.29 (-0.56-3.17) |
| CKD | Monaco | 5 (3.8-6.1) | 6.1 (4.6-7.4) | 12 (9.4-14.4) | 9.6 (7.6-11.4) | 0.98 (-0.04-2.01) |
| CKD | Mongolia | 173.4 (138.9-213.4) | 15.4 (12.4-18.9) | 296.3 (236-365.1) | 13.6 (10.8-16.8) | -0.51 (-1.92-0.91) |
| CKD | Montenegro | 76 (61.1-92.9) | 12.9 (10.4-15.8) | 165.4 (124-207.8) | 19.1 (14-23.9) | 1.02 (-0.14-2.19) |
| CKD | Morocco | 3070.2 (2131.1-5653) | 23.1 (15.7-45.6) | 11890.2 (8910.6-15305.5) | 40 (30.3-51.3) | 1.55 (-0.11-3.24) |
| CKD | Mozambique | 1433 (1145-1941.8) | 26 (20.8-37.4) | 3627.3 (2744.2-4921.7) | 35.7 (26.9-48.8) | 1.25 (0.05-2.46) |
| CKD | Myanmar | 6501.1 (4977.4-8335.8) | 26.6 (20.6-34.4) | 11244.5 (9000.8-14130.9) | 24.9 (20.3-31.1) | -0.52 (-2.02-1) |
| CKD | Namibia | 119.1 (92.2-168.8) | 21.8 (16.7-31) | 313.5 (230.9-422.3) | 26.7 (20.3-35.2) | 0.35 (-0.96-1.69) |
| CKD | Nauru | 1.8 (1.1-2.7) | 41.1 (26.1-68.8) | 3.4 (2.4-5) | 63 (42.9-105.4) | 0.96 (-0.88-2.83) |
| CKD | Nepal | 1249.3 (959.6-1662.6) | 13.5 (10.4-18.1) | 4047.9 (2946.9-5423.8) | 19.2 (14.1-25.1) | 1.02 (0.04-2.02) |
| CKD | Netherlands | 1324.8 (1178.7-1417.3) | 6.6 (5.9-7.1) | 4434.6 (3714.8-4925) | 10.9 (9.2-12.1) | 0.98 (-0.14-2.11) |
| CKD | New Zealand | 271.2 (249.2-288.3) | 7.3 (6.6-7.7) | 956.7 (834.8-1041.2) | 10.7 (9.4-11.6) | 0.92 (-0.14-2) |
| CKD | Nicaragua | 492.3 (443.8-663.3) | 29.4 (26.2-40.1) | 2686.4 (2147.7-3229.7) | 55.3 (44.7-66.5) | 2.35 (1.14-3.58) |
| CKD | Niger | 874.1 (706.4-1119.3) | 29.5 (23.9-38.7) | 2007 (1439.6-3037.6) | 27 (19.9-40.1) | -0.31 (-1.72-1.13) |
| CKD | Nigeria | 12895.3 (10282.3-15658.3) | 31 (25.1-37.6) | 25428.5 (19317.5-32599.4) | 31.3 (25-38.1) | -0.18 (-1.61-1.27) |
| CKD | Niue | 0.7 (0.5-0.9) | 29.9 (23.1-40.3) | 1.1 (0.7-1.7) | 56.1 (33.1-86.2) | 1.49 (-0.31-3.33) |
| CKD | North Macedonia | 173 (145.4-208.1) | 10.1 (8.5-12.2) | 369.7 (282-483.7) | 13.6 (10.7-17.3) | 0.55 (-0.69-1.82) |
| CKD | Northern Mariana Islands | 5.9 (4.5-7.7) | 37.3 (30.1-47.4) | 24.9 (20.4-30.1) | 57.6 (47.3-68.4) | 1.07 (-0.89-3.06) |
| CKD | Norway | 268.3 (240.8-281.8) | 3.5 (3.2-3.7) | 805.1 (672.8-878.2) | 6.5 (5.5-7) | 1.07 (0.22-1.92) |
| CKD | Oman | 140 (102.3-206.9) | 22.9 (16.6-33.8) | 616.4 (490.3-770.6) | 41.7 (33-50.4) | 1.99 (0.44-3.57) |
| CKD | Pakistan | 10395 (8266.2-13166.9) | 18.8 (14.9-23.7) | 31062.6 (24330-39843.6) | 26.6 (21.4-33.9) | 0.75 (-0.32-1.83) |
| CKD | Palau | 2.6 (1.9-3.5) | 29.5 (21.1-39.3) | 9 (6.4-12) | 50.2 (36.2-66.3) | 1.48 (-0.3-3.3) |
| CKD | Palestine | 348.5 (260.5-460.4) | 45.4 (34.2-61.6) | 837.9 (710.6-1009.8) | 43.2 (36.7-51.9) | -0.17 (-1.85-1.54) |
| CKD | Panama | 201 (188.1-215.4) | 13.5 (12.6-14.5) | 1381 (1090.2-1653.4) | 30.7 (24.3-36.8) | 2.33 (1.17-3.5) |
| CKD | Papua New Guinea | 220.2 (145-311.6) | 11.3 (7.9-15.9) | 728.3 (561.1-944.9) | 13.6 (10.4-19) | 0.36 (-0.92-1.65) |
| CKD | Paraguay | 355.2 (301.2-418.3) | 16.1 (13.6-19.1) | 1388.5 (1035.9-1761.3) | 24.9 (18.7-31.5) | 1.14 (-0.09-2.38) |
| CKD | Peru | 3242.2 (2795.8-3747.7) | 27 (23.3-31.4) | 10386.2 (7395.5-13295.2) | 30.9 (22.1-39.7) | 0.07 (-1.23-1.38) |
| CKD | Philippines | 8625.8 (7839-9922.3) | 31.7 (28.9-35.6) | 32882.8 (27438.3-38113) | 41.7 (35-48.1) | 1.03 (-0.68-2.78) |
| CKD | Poland | 5308.6 (5122.9-5460.2) | 12.8 (12.3-13.2) | 4980.6 (4384.3-5654.3) | 6.6 (5.8-7.6) | -1.57 (-2.61--0.52) |
| CKD | Portugal | 1597.1 (1486.1-1686.7) | 13 (12-13.8) | 4736.4 (3954.3-5301.3) | 14.5 (12.3-16.2) | 0.23 (-0.99-1.47) |
| CKD | Puerto Rico | 1091.3 (1030.8-1147.7) | 31.8 (29.9-33.4) | 2537.1 (2092.9-2995.1) | 32.1 (26.6-37.9) | 0.53 (-0.81-1.88) |
| CKD | Qatar | 27.5 (15.9-43.5) | 40.7 (22.7-66) | 198.1 (152.5-254.2) | 39.9 (32.4-49.2) | -0.47 (-2.17-1.25) |
| CKD | Republic of Korea | 4257.5 (3775.2-4740) | 18.6 (16.3-21.4) | 9823 (7901.9-11473.5) | 10.8 (8.7-12.7) | -1.32 (-2.43--0.19) |
| CKD | Republic of Moldova | 83 (79.2-86.9) | 2 (1.9-2.1) | 205.9 (179.2-242.4) | 3.6 (3.2-4.2) | 0.5 (-0.13-1.14) |
| CKD | Romania | 2550.6 (2408-2681.7) | 10.4 (9.8-10.9) | 3324.5 (2869.5-3831) | 8.6 (7.4-10) | -0.16 (-1.3-1) |
| CKD | Russian Federation | 8804.3 (8611.7-8978.8) | 5.1 (5-5.2) | 14470.6 (13013.1-16148.7) | 6.2 (5.6-6.9) | 0 (-0.79-0.8) |
| CKD | Rwanda | 1301.4 (1058.6-1534.3) | 47.9 (39.1-57) | 2067.7 (1481.1-2764.6) | 38.7 (28-51.2) | -1.4 (-2.65--0.14) |
| CKD | Saint Kitts and Nevis | 15.9 (14.7-17.1) | 43.6 (40.5-46.8) | 34.2 (28-39.7) | 57.6 (48.5-65.8) | 1.3 (-0.27-2.89) |
| CKD | Saint Lucia | 30.4 (28.8-31.9) | 37.3 (35.3-39.2) | 92.8 (76.9-110.6) | 40.3 (33.5-47.9) | 0.46 (-1.02-1.96) |
| CKD | Saint Vincent and the Grenadines | 18.7 (17.4-20) | 26.9 (24.9-28.7) | 54.1 (47.6-61.3) | 41.1 (36.2-46.4) | 1.57 (0.14-3.03) |
| CKD | Samoa | 24.5 (17.3-32.8) | 31 (22.2-41) | 58.3 (43.8-75.1) | 43.5 (33-55.5) | 0.76 (-0.99-2.54) |
| CKD | San Marino | 2 (1.7-2.5) | 5.5 (4.5-6.6) | 4.3 (2.9-5.8) | 3.9 (2.6-5.3) | 0.12 (-0.76-1) |
| CKD | Sao Tome and Principe | 33.6 (28-38.4) | 53.7 (45.3-60.8) | 69.5 (50.1-84.6) | 72.4 (51.4-86.9) | 0.88 (-0.59-2.37) |
| CKD | Saudi Arabia | 2407.7 (1797.7-3342.3) | 45.1 (33.5-64.1) | 12226.1 (9095.7-15498.3) | 79.3 (59.8-95.7) | 1.45 (-0.22-3.14) |
| CKD | Senegal | 1299.8 (1084.2-1592.6) | 40.6 (34.1-50.3) | 3034.9 (2314.9-4174.8) | 43.5 (33.6-59.2) | 0.09 (-1.28-1.48) |
| CKD | Serbia | 1464.4 (1214.1-1828.2) | 16.5 (13.6-20.8) | 2712.8 (2195.9-3251.7) | 15.9 (12.8-19.1) | -0.06 (-1.24-1.13) |
| CKD | Seychelles | 16.7 (14.5-19) | 29.3 (25.5-33.4) | 50 (40.4-58.4) | 47.2 (37.9-55.4) | 1.22 (-0.66-3.14) |
| CKD | Sierra Leone | 627.7 (495.4-784.3) | 30.1 (23.8-37.2) | 1047.6 (801.6-1392) | 29 (22.6-37.5) | -0.09 (-1.43-1.27) |
| CKD | Singapore | 263.7 (250-275.4) | 13.7 (12.8-14.4) | 833.1 (718.3-926.3) | 10.2 (8.7-11.3) | -0.35 (-1.44-0.75) |
| CKD | Slovakia | 616.8 (530.3-707.7) | 10.6 (9.1-12.2) | 831.5 (676.9-1002) | 8.9 (7.3-10.7) | -0.31 (-1.37-0.76) |
| CKD | Slovenia | 152.1 (141.9-162.7) | 6.4 (5.9-6.9) | 327.1 (261.1-414.6) | 6.1 (4.9-7.8) | 0.32 (-0.65-1.29) |
| CKD | Solomon Islands | 30.8 (12.6-50.4) | 22.1 (11.6-34.6) | 90.1 (68.9-115.6) | 25 (20.1-31.5) | 0.27 (-1.31-1.88) |
| CKD | Somalia | 1051.5 (755-1454) | 45.7 (33.4-63.2) | 2791.1 (1917.5-4130.2) | 47.9 (33.1-68.3) | 0.26 (-0.96-1.49) |
| CKD | South Africa | 4020.4 (3508-4992.9) | 19.7 (16.8-25.1) | 13501.7 (12123.7-14916.3) | 33.2 (29.6-36.6) | 1.4 (0.01-2.81) |
| CKD | South Sudan | 1008.7 (752.7-1391) | 40.9 (31-56.2) | 1949.4 (1427.7-2538.8) | 55 (39.9-71.2) | 0.79 (-0.47-2.06) |
| CKD | Spain | 6906.8 (6235.5-7340.1) | 13.3 (11.9-14.1) | 14802.3 (11854.1-16621.3) | 10.5 (8.6-11.8) | -0.35 (-1.51-0.83) |
| CKD | Sri Lanka | 2613.5 (2218.8-3080.3) | 27.7 (23.3-32.8) | 5243.4 (3566.9-7155) | 21.2 (14.6-28.5) | -0.61 (-2.26-1.06) |
| CKD | Sudan | 2043.1 (1461.5-3606.7) | 22.7 (15.9-43.5) | 5601.7 (4127.1-7494.7) | 32.1 (24.3-43.8) | 0.88 (-0.62-2.39) |
| CKD | Suriname | 70.9 (61.5-81.4) | 27.7 (24.2-31.8) | 253.5 (189.2-328.8) | 41.4 (31-53.6) | 1.34 (-0.12-2.83) |
| CKD | Sweden | 636 (577.8-678) | 3.8 (3.5-4.1) | 2517.9 (2087.3-2833.7) | 8.9 (7.4-9.9) | 1.71 (0.82-2.6) |
| CKD | Switzerland | 807.6 (722.4-860.3) | 7.1 (6.4-7.6) | 2599.4 (2041.6-2973) | 10.2 (8.1-11.7) | 1.13 (0.05-2.23) |
| CKD | Syrian Arab Republic | 1951.3 (1523.9-2563.1) | 39.1 (30.4-52.8) | 4836.2 (3387.1-6504.1) | 47.7 (33.6-62.8) | 0.18 (-1.45-1.84) |
| CKD | Taiwan (Province of China) | 2954.4 (2802-3082.6) | 24.1 (22.5-25.4) | 8813.8 (7593-9770.8) | 20 (17.4-22.1) | -0.18 (-1.77-1.43) |
| CKD | Tajikistan | 59.2 (48.5-82.9) | 1.8 (1.4-2.6) | 151.6 (105.7-219.8) | 2.4 (1.7-3.3) | 0.12 (-0.59-0.83) |
| CKD | Thailand | 7940.7 (6624.3-9995) | 24.3 (20.2-30.6) | 31733.6 (24196.9-39500.8) | 29.9 (22.8-37.1) | -0.03 (-1.7-1.66) |
| CKD | Timor-Leste | 75.7 (57.3-100.7) | 25.6 (19.7-35.6) | 238.1 (171.8-334.5) | 29.8 (21.8-41.4) | 0.33 (-1.29-1.98) |
| CKD | Togo | 381.2 (313.7-466.9) | 31 (26.1-38) | 1135.6 (853.6-1520.4) | 34.5 (26.7-45.2) | 0.18 (-1.16-1.55) |
| CKD | Tokelau | 0.3 (0.2-0.5) | 24.6 (17.1-42.5) | 0.5 (0.4-0.8) | 37.3 (27-54.7) | 0.96 (-0.74-2.68) |
| CKD | Tonga | 7 (4.8-9.7) | 13.4 (9.4-18.6) | 16.1 (11.2-21.2) | 20.6 (14.3-27) | 0.74 (-0.8-2.31) |
| CKD | Trinidad and Tobago | 192.2 (183.6-200.9) | 24.7 (23.5-25.8) | 755.1 (564.3-966.9) | 40.2 (30.1-51.3) | 1.78 (0.33-3.25) |
| CKD | Tunisia | 926.4 (705.8-1391.6) | 22.2 (17-33.3) | 3765.3 (2674.2-5162.3) | 32.1 (23.1-43.9) | 0.91 (-0.57-2.42) |
| CKD | Turkey | 9286.2 (7514.9-12586.4) | 30.6 (24.5-42.5) | 24804.6 (19763.8-30524.4) | 29.5 (23.3-36.4) | 0.17 (-1.29-1.66) |
| CKD | Turkmenistan | 208.8 (190.5-228.4) | 8.9 (8.1-9.8) | 744.2 (574.2-946.9) | 18 (14-22.7) | 1.2 (-0.22-2.65) |
| CKD | Tuvalu | 1.7 (1.3-2.7) | 27.8 (20.8-43.6) | 3.9 (2.8-5.7) | 40.7 (29.7-58.9) | 0.92 (-0.81-2.67) |
| CKD | Uganda | 1868 (1403.6-2442.6) | 31 (23.5-40.7) | 5279 (4093-6903.1) | 38.8 (30.8-50.8) | 0.36 (-0.84-1.58) |
| CKD | Ukraine | 83.8 (80.4-87.5) | 0.1 (0.1-0.1) | 1604.9 (1203.7-2049.4) | 2.4 (1.8-3.1) | 2.08 (1.66-2.5) |
| CKD | United Arab Emirates | 82.8 (56.6-118.7) | 23.1 (16-32.6) | 672 (427.1-913.8) | 37.5 (20.9-49.2) | 2.89 (1.24-4.57) |
| CKD | United Kingdom | 4239.2 (3908.1-4410.2) | 4.6 (4.2-4.8) | 9679.4 (8207.3-10541) | 6.2 (5.4-6.8) | 0.9 (0.05-1.75) |
| CKD | United Republic of Tanzania | 3665.4 (3049.2-4635.5) | 36.1 (30.3-46.2) | 8859.6 (7120.6-11155.5) | 37.7 (30.1-46.9) | 0.05 (-1.44-1.56) |
| CKD | United States of America | 27826.2 (25244.9-29209.6) | 8.5 (7.7-8.9) | 135879.9 (118166.7-146605.5) | 21.9 (19.3-23.5) | 2.19 (0.97-3.43) |
| CKD | United States Virgin Islands | 15.2 (12.5-18.5) | 19.9 (16.6-24) | 33 (22.8-43.5) | 20.5 (14.7-26.6) | 0.38 (-1.03-1.82) |
| CKD | Uruguay | 598.2 (560.9-627.5) | 15.5 (14.5-16.2) | 1167.3 (1033.7-1269.9) | 17.9 (16.1-19.4) | 0.37 (-0.86-1.62) |
| CKD | Uzbekistan | 617.2 (441.5-920) | 5 (3.4-7.9) | 3588.6 (3013.3-4203.9) | 14.2 (12.1-16.7) | 1.35 (0.01-2.72) |
| CKD | Vanuatu | 12.8 (7.9-22.5) | 22 (14.3-38.7) | 55.5 (39.8-84.5) | 34.3 (24.7-52.6) | 1.05 (-0.62-2.75) |
| CKD | Venezuela (Bolivarian Republic of) | 1587.3 (1500.6-1651.3) | 16.3 (15.2-17.1) | 11753.5 (8832.7-14984.4) | 40.5 (30.4-51.3) | 2.15 (0.97-3.35) |
| CKD | Viet Nam | 9150.1 (6656.5-11695.3) | 23.5 (17.1-30.1) | 23705.8 (16677.5-29758.8) | 27 (19.1-33.4) | 0.34 (-1.31-2.02) |
| CKD | Yemen | 871.4 (580-1629.4) | 19.1 (12.7-38.9) | 2471.5 (1678.3-4179.4) | 20.6 (14.2-34.6) | 0.09 (-1.24-1.44) |
| CKD | Zambia | 1293 (1071.5-1517.9) | 46.3 (39.5-54.3) | 3432.5 (2424.2-4961.7) | 52.2 (39.2-69.8) | 0.09 (-1.15-1.34) |
| CKD | Zimbabwe | 874.3 (693.2-1148.5) | 26 (20.5-34.2) | 2482.9 (1892.9-3316.6) | 41.4 (31.8-53.6) | 1.63 (0.19-3.08) |
| CKD due to diabetes mellitus type 1 | Afghanistan | 76.8 (46-128.1) | 1 (0.6-1.7) | 159.8 (82-282.4) | 1 (0.5-1.7) | 0.36 (-1.22-1.96) |
| CKD due to diabetes mellitus type 1 | Albania | 5 (3.5-7.4) | 0.2 (0.1-0.3) | 6.5 (4.3-10) | 0.2 (0.1-0.3) | -0.11 (-1.3-1.09) |
| CKD due to diabetes mellitus type 1 | Algeria | 56.4 (35.7-101.7) | 0.4 (0.2-0.7) | 192.8 (129.4-282.7) | 0.4 (0.3-0.6) | 1.13 (-0.42-2.7) |
| CKD due to diabetes mellitus type 1 | American Samoa | 1.8 (1.2-2.6) | 5.5 (3.7-8.1) | 6.7 (4.8-9.1) | 12.7 (9-16.9) | 2.12 (0.13-4.16) |
| CKD due to diabetes mellitus type 1 | Andorra | 0.1 (0.1-0.2) | 0.2 (0.1-0.3) | 0.2 (0.1-0.3) | 0.1 (0.1-0.2) | -0.33 (-1.44-0.79) |
| CKD due to diabetes mellitus type 1 | Angola | 72.3 (46.3-106.4) | 1.3 (0.8-2) | 214.9 (130.9-325.7) | 1.3 (0.8-1.9) | -0.01 (-1.29-1.29) |
| CKD due to diabetes mellitus type 1 | Antigua and Barbuda | 1.4 (1.1-1.7) | 2.8 (2.1-3.5) | 4 (3-5.3) | 3.5 (2.6-4.6) | 1.66 (0.11-3.24) |
| CKD due to diabetes mellitus type 1 | Argentina | 288.8 (210.6-399.8) | 0.9 (0.6-1.2) | 362.7 (261.1-507.5) | 0.7 (0.5-1) | -0.28 (-1.65-1.11) |
| CKD due to diabetes mellitus type 1 | Armenia | 0.7 (0.5-1) | 0 (0-0) | 6.7 (4.2-10.3) | 0.2 (0.1-0.3) | 3.05 (1.96-4.16) |
| CKD due to diabetes mellitus type 1 | Australia | 11.7 (10.2-13.3) | 0.1 (0.1-0.1) | 23.3 (16.1-33.5) | 0.1 (0-0.1) | 0.64 (-0.47-1.77) |
| CKD due to diabetes mellitus type 1 | Austria | 14.5 (10.3-20.1) | 0.1 (0.1-0.2) | 20 (16-25.1) | 0.1 (0.1-0.2) | 2.48 (1.37-3.61) |
| CKD due to diabetes mellitus type 1 | Azerbaijan | 11 (7-16.3) | 0.2 (0.1-0.3) | 27 (16.6-41.4) | 0.2 (0.1-0.3) | 0.95 (-0.28-2.2) |
| CKD due to diabetes mellitus type 1 | Bahamas | 5 (4-6.4) | 2.6 (2-3.3) | 17.8 (12.7-23.7) | 3.9 (2.8-5.2) | 1.65 (0.24-3.08) |
| CKD due to diabetes mellitus type 1 | Bahrain | 1.3 (0.8-1.9) | 0.4 (0.3-0.7) | 6.7 (4.3-9.7) | 0.4 (0.3-0.6) | 0.24 (-1.54-2.04) |
| CKD due to diabetes mellitus type 1 | Bangladesh | 582 (382-828.3) | 1 (0.7-1.5) | 1115.1 (705.4-1582.3) | 0.7 (0.5-1) | -0.46 (-1.43-0.52) |
| CKD due to diabetes mellitus type 1 | Barbados | 4.6 (3.6-5.8) | 1.9 (1.5-2.4) | 10.7 (7.3-15.3) | 2.4 (1.7-3.4) | 1.28 (-0.15-2.73) |
| CKD due to diabetes mellitus type 1 | Belarus | 8.8 (6.5-11.8) | 0.1 (0.1-0.1) | 35.7 (24.5-51.1) | 0.2 (0.2-0.3) | 0.89 (0.42-1.36) |
| CKD due to diabetes mellitus type 1 | Belgium | 17.1 (12-24.2) | 0.1 (0.1-0.2) | 17.9 (12.5-25.4) | 0.1 (0.1-0.1) | 0.39 (-0.7-1.49) |
| CKD due to diabetes mellitus type 1 | Belize | 2.5 (1.9-3.1) | 2.3 (1.8-3) | 17 (13.1-21.6) | 4.6 (3.5-5.9) | 2.06 (0.57-3.56) |
| CKD due to diabetes mellitus type 1 | Benin | 23.2 (15.9-33.8) | 1 (0.6-1.4) | 68.4 (45.1-100.5) | 1 (0.6-1.4) | 0.07 (-1.35-1.51) |
| CKD due to diabetes mellitus type 1 | Bermuda | 0.9 (0.7-1.1) | 1.3 (1-1.7) | 1.2 (0.9-1.7) | 1.2 (0.8-1.6) | 0.27 (-1-1.56) |
| CKD due to diabetes mellitus type 1 | Bhutan | 4 (2.4-6.2) | 1.3 (0.7-2) | 8.4 (5-12.8) | 1.2 (0.7-1.9) | 0.51 (-0.52-1.54) |
| CKD due to diabetes mellitus type 1 | Bolivia (Plurinational State of) | 107.1 (74.7-144.3) | 2.8 (1.9-3.8) | 329.9 (211.6-495.7) | 3.2 (2.1-4.9) | 0.81 (-0.57-2.21) |
| CKD due to diabetes mellitus type 1 | Bosnia and Herzegovina | 22.4 (15.8-31.2) | 0.5 (0.3-0.7) | 26.6 (18.1-37.5) | 0.5 (0.3-0.7) | 0.25 (-0.8-1.32) |
| CKD due to diabetes mellitus type 1 | Botswana | 4.1 (2.4-6.9) | 0.6 (0.4-1) | 12.6 (7.3-20.6) | 0.6 (0.4-1.1) | 0.61 (-0.72-1.96) |
| CKD due to diabetes mellitus type 1 | Brazil | 1376.6 (1072.9-1770.6) | 1.3 (1-1.6) | 2938.5 (2191.6-3832.6) | 1.1 (0.8-1.5) | 0.03 (-1.14-1.21) |
| CKD due to diabetes mellitus type 1 | Brunei Darussalam | 3 (2.2-4) | 2.1 (1.5-2.9) | 8.4 (6.3-11.2) | 1.8 (1.3-2.4) | 0.39 (-0.97-1.77) |
| CKD due to diabetes mellitus type 1 | Bulgaria | 21.3 (14.7-29.9) | 0.2 (0.1-0.3) | 48.8 (32-74.1) | 0.4 (0.3-0.7) | 2.31 (1.12-3.51) |
| CKD due to diabetes mellitus type 1 | Burkina Faso | 46.6 (31-68.5) | 0.9 (0.6-1.3) | 110.9 (74.7-158.2) | 0.9 (0.6-1.3) | 0.35 (-1.12-1.85) |
| CKD due to diabetes mellitus type 1 | Burundi | 56.4 (38-81.1) | 2 (1.3-3) | 103.4 (66.6-150.3) | 1.5 (1-2.3) | -0.71 (-1.9-0.5) |
| CKD due to diabetes mellitus type 1 | Cabo Verde | 1.1 (0.7-1.5) | 0.5 (0.3-0.7) | 4.1 (2.5-6.1) | 0.8 (0.5-1.2) | 1.32 (0.1-2.56) |
| CKD due to diabetes mellitus type 1 | Cambodia | 167.4 (123.9-226) | 2.5 (1.9-3.4) | 353.1 (235.4-511.9) | 2.2 (1.5-3.2) | 0.02 (-1.47-1.53) |
| CKD due to diabetes mellitus type 1 | Cameroon | 110.9 (72.8-160.4) | 1.9 (1.2-2.8) | 367.4 (218.5-572.3) | 2 (1.2-3.1) | 0 (-1.39-1.42) |
| CKD due to diabetes mellitus type 1 | Canada | 26.6 (18.9-37.1) | 0.1 (0.1-0.1) | 75.2 (53.2-104.7) | 0.1 (0.1-0.2) | 0.85 (-0.23-1.94) |
| CKD due to diabetes mellitus type 1 | Central African Republic | 30.5 (19.7-44.4) | 2 (1.3-2.9) | 64.4 (41.8-102.3) | 2 (1.3-3.1) | 0.03 (-1.24-1.3) |
| CKD due to diabetes mellitus type 1 | Chad | 25.2 (16.2-39) | 0.8 (0.5-1.2) | 73 (47.4-119.4) | 0.9 (0.6-1.5) | 0.35 (-0.98-1.69) |
| CKD due to diabetes mellitus type 1 | Chile | 47.8 (34.2-64.3) | 0.4 (0.3-0.6) | 94.8 (67.5-134) | 0.4 (0.3-0.5) | 0.78 (-0.52-2.1) |
| CKD due to diabetes mellitus type 1 | China | 19456.7 (15416.9-24181.1) | 1.8 (1.4-2.3) | 20687.8 (15277.1-27013.5) | 1.1 (0.8-1.4) | -0.72 (-2-0.58) |
| CKD due to diabetes mellitus type 1 | Colombia | 153.1 (112.7-203.5) | 0.7 (0.5-1) | 260.5 (172.4-376.8) | 0.5 (0.3-0.7) | -0.42 (-1.5-0.67) |
| CKD due to diabetes mellitus type 1 | Comoros | 4.2 (2.7-6.2) | 1.7 (1.1-2.5) | 9.5 (6.2-13.6) | 1.6 (1-2.3) | 0.04 (-1.21-1.3) |
| CKD due to diabetes mellitus type 1 | Congo | 30.2 (18.4-43.8) | 2.3 (1.4-3.3) | 78.6 (44.8-119) | 2 (1.2-3) | -0.26 (-1.56-1.06) |
| CKD due to diabetes mellitus type 1 | Cook Islands | 0.4 (0.3-0.5) | 2.6 (1.8-3.5) | 0.7 (0.5-1) | 3.2 (2.2-4.3) | 0.51 (-1.07-2.1) |
| CKD due to diabetes mellitus type 1 | Costa Rica | 12.2 (9-16.4) | 0.6 (0.5-0.9) | 71.5 (49.5-98.6) | 1.3 (0.9-1.8) | 1.71 (0.57-2.86) |
| CKD due to diabetes mellitus type 1 | C么te d'Ivoire | 53.4 (35-80.4) | 0.9 (0.6-1.3) | 152.9 (95.5-227.8) | 0.9 (0.6-1.4) | -0.03 (-1.6-1.56) |
| CKD due to diabetes mellitus type 1 | Croatia | 9.1 (6.4-13.2) | 0.1 (0.1-0.2) | 9.8 (6.2-15.1) | 0.1 (0.1-0.2) | 0.64 (-0.48-1.79) |
| CKD due to diabetes mellitus type 1 | Cuba | 91.7 (74.8-113.8) | 0.9 (0.7-1.1) | 249.7 (178.2-329.2) | 1.4 (1.1-1.9) | 1.64 (0.47-2.83) |
| CKD due to diabetes mellitus type 1 | Cyprus | 3.1 (2-4.7) | 0.4 (0.2-0.6) | 4.3 (2.9-6.4) | 0.2 (0.1-0.3) | -1.78 (-3.25--0.28) |
| CKD due to diabetes mellitus type 1 | Czechia | 28.3 (20.2-39.7) | 0.2 (0.2-0.3) | 18.6 (12-28.6) | 0.1 (0.1-0.2) | -0.28 (-1.25-0.7) |
| CKD due to diabetes mellitus type 1 | Democratic People's Republic of Korea | 409 (270.9-596) | 2 (1.4-2.9) | 706.9 (493-1000.8) | 2.1 (1.5-3) | 0.03 (-1.31-1.39) |
| CKD due to diabetes mellitus type 1 | Democratic Republic of the Congo | 332.3 (223.3-479.9) | 1.6 (1.1-2.4) | 843.3 (527.9-1273.8) | 1.6 (1-2.5) | -0.04 (-1.29-1.23) |
| CKD due to diabetes mellitus type 1 | Denmark | 10.4 (7.4-14.7) | 0.1 (0.1-0.2) | 22.1 (16.9-28.7) | 0.2 (0.2-0.3) | 1.76 (0.73-2.8) |
| CKD due to diabetes mellitus type 1 | Djibouti | 2.7 (1.7-4) | 1.3 (0.8-2.1) | 16.3 (10.3-24.1) | 1.8 (1.1-2.8) | 0.96 (-0.27-2.19) |
| CKD due to diabetes mellitus type 1 | Dominica | 1.5 (1.1-2) | 2.7 (1.9-3.5) | 3.7 (2.6-5.3) | 4.5 (3.1-6.3) | 1.41 (-0.14-2.97) |
| CKD due to diabetes mellitus type 1 | Dominican Republic | 65.6 (49.7-87.2) | 1.4 (1-1.9) | 242.6 (148.7-339.1) | 2.2 (1.4-3.1) | 1.45 (0.16-2.76) |
| CKD due to diabetes mellitus type 1 | Ecuador | 89.2 (68.8-113.6) | 1.4 (1.1-1.8) | 407.9 (242.4-677.9) | 2.4 (1.4-4) | 1.51 (0.09-2.95) |
| CKD due to diabetes mellitus type 1 | Egypt | 220.8 (145.8-354.1) | 0.6 (0.4-1) | 708.8 (463-1067.7) | 0.8 (0.6-1.3) | 1.2 (-0.56-3) |
| CKD due to diabetes mellitus type 1 | El Salvador | 41.1 (28.7-63.1) | 1.2 (0.9-1.9) | 218.1 (137.3-318.3) | 3.6 (2.3-5.3) | 3.01 (1.78-4.26) |
| CKD due to diabetes mellitus type 1 | Equatorial Guinea | 4.4 (2.8-6.3) | 1.8 (1.1-2.6) | 14.7 (7.8-24) | 1.9 (1-3.2) | 0.84 (-0.46-2.16) |
| CKD due to diabetes mellitus type 1 | Eritrea | 32.7 (19.5-52.7) | 1.9 (1.1-3.1) | 73.3 (44.9-119.5) | 1.8 (1.1-3) | 0.39 (-0.79-1.58) |
| CKD due to diabetes mellitus type 1 | Estonia | 12.8 (9.4-17.5) | 0.7 (0.5-0.9) | 21.9 (16.6-28.9) | 1 (0.8-1.3) | 1.82 (0.87-2.77) |
| CKD due to diabetes mellitus type 1 | Eswatini | 4.5 (2.9-6.7) | 1.2 (0.7-1.8) | 14.5 (7.3-22.6) | 1.9 (0.9-3) | 1.27 (-0.14-2.7) |
| CKD due to diabetes mellitus type 1 | Ethiopia | 1553.8 (1076.2-2005.3) | 5.7 (4-7.5) | 1630.1 (1226.2-2139.3) | 2.7 (1.9-3.6) | -1.95 (-3.2--0.69) |
| CKD due to diabetes mellitus type 1 | Fiji | 25.8 (17.2-37.1) | 4.7 (3.2-6.9) | 67.8 (46.9-95.9) | 7.3 (5.1-10.3) | 0.97 (-0.82-2.78) |
| CKD due to diabetes mellitus type 1 | Finland | 6.9 (5.1-9.5) | 0.1 (0.1-0.1) | 12.4 (9.7-15.8) | 0.1 (0.1-0.1) | 0.89 (0.12-1.68) |
| CKD due to diabetes mellitus type 1 | France | 81.4 (57.6-113.7) | 0.1 (0.1-0.1) | 105.9 (78.2-139.6) | 0.1 (0.1-0.1) | 0.15 (-0.84-1.16) |
| CKD due to diabetes mellitus type 1 | Gabon | 11.7 (7.7-17.4) | 1.9 (1.2-2.8) | 33.2 (15.6-49.7) | 2.5 (1.2-3.8) | 0.93 (-0.43-2.3) |
| CKD due to diabetes mellitus type 1 | Gambia | 4.4 (2.8-6.3) | 0.9 (0.6-1.4) | 15.2 (9.8-22.1) | 1.2 (0.7-1.7) | 0.53 (-0.87-1.94) |
| CKD due to diabetes mellitus type 1 | Georgia | 7.1 (4.8-10.4) | 0.1 (0.1-0.2) | 12.4 (8.1-18.8) | 0.3 (0.2-0.4) | 2.41 (1.21-3.64) |
| CKD due to diabetes mellitus type 1 | Germany | 178 (125-249.2) | 0.2 (0.1-0.2) | 188.8 (127.7-271.5) | 0.1 (0.1-0.2) | 2 (0.83-3.19) |
| CKD due to diabetes mellitus type 1 | Ghana | 82.1 (53.8-124.2) | 1 (0.6-1.5) | 355.3 (236-523) | 1.6 (1-2.3) | 1.59 (0.23-2.97) |
| CKD due to diabetes mellitus type 1 | Greece | 36.1 (25.7-51.4) | 0.2 (0.2-0.3) | 40.1 (31.3-51.3) | 0.2 (0.2-0.3) | -0.45 (-1.79-0.9) |
| CKD due to diabetes mellitus type 1 | Greenland | 0.1 (0.1-0.1) | 0.2 (0.1-0.3) | 0.2 (0.1-0.3) | 0.2 (0.1-0.3) | 0.58 (-0.65-1.82) |
| CKD due to diabetes mellitus type 1 | Grenada | 2.4 (1.8-3) | 3.9 (2.9-4.9) | 6.5 (4.8-8.6) | 5.3 (4-6.9) | 1.63 (0.08-3.22) |
| CKD due to diabetes mellitus type 1 | Guam | 2.2 (1.7-3) | 2.1 (1.5-2.8) | 8.2 (6.2-10.5) | 4.3 (3.3-5.4) | 0.92 (-0.51-2.37) |
| CKD due to diabetes mellitus type 1 | Guatemala | 53.7 (40.2-71.7) | 1.2 (0.9-1.6) | 287.3 (194.3-399.9) | 2.3 (1.6-3.3) | 1.94 (0.65-3.24) |
| CKD due to diabetes mellitus type 1 | Guinea | 22.8 (14.2-36) | 0.6 (0.4-1) | 45.9 (29.3-71.7) | 0.6 (0.4-1) | 0.15 (-1.34-1.67) |
| CKD due to diabetes mellitus type 1 | Guinea-Bissau | 8.4 (5.6-12.2) | 1.6 (1.1-2.4) | 15.6 (10.3-23.2) | 1.4 (0.9-2.2) | -0.3 (-1.69-1.11) |
| CKD due to diabetes mellitus type 1 | Guyana | 14.8 (11.3-19.1) | 3 (2.3-4) | 45.6 (30.2-65) | 6.1 (4.1-8.6) | 2.57 (1.09-4.07) |
| CKD due to diabetes mellitus type 1 | Haiti | 120.9 (79.1-199.7) | 2.9 (1.8-4.9) | 287.9 (153.2-611.5) | 2.9 (1.5-6.5) | 0.34 (-1.01-1.7) |
| CKD due to diabetes mellitus type 1 | Honduras | 14.3 (9.8-20.1) | 0.6 (0.4-0.8) | 72.8 (44.9-112.6) | 1 (0.6-1.6) | 1.9 (0.81-3) |
| CKD due to diabetes mellitus type 1 | Hungary | 28.1 (20.2-39.8) | 0.2 (0.1-0.3) | 30.8 (20.6-46.3) | 0.2 (0.1-0.3) | 1.37 (0.32-2.43) |
| CKD due to diabetes mellitus type 1 | Iceland | 0.1 (0.1-0.2) | 0 (0-0.1) | 0.3 (0.3-0.4) | 0.1 (0.1-0.1) | 0.97 (0.14-1.8) |
| CKD due to diabetes mellitus type 1 | India | 5715.7 (4063.1-7551.1) | 1 (0.7-1.3) | 15463.7 (10796.4-21435.5) | 1.2 (0.8-1.6) | 0.37 (-0.59-1.34) |
| CKD due to diabetes mellitus type 1 | Indonesia | 3125.3 (2358.9-3980.5) | 2.1 (1.6-2.7) | 7564.3 (5596.7-10259.8) | 2.5 (1.8-3.3) | 0.58 (-0.87-2.05) |
| CKD due to diabetes mellitus type 1 | Iran (Islamic Republic of) | 108.8 (71.3-161.9) | 0.3 (0.2-0.5) | 283.3 (199.2-389.1) | 0.3 (0.2-0.4) | 0.34 (-1.09-1.79) |
| CKD due to diabetes mellitus type 1 | Iraq | 84.1 (51.7-127.6) | 0.8 (0.5-1.3) | 236.8 (120-385.8) | 0.7 (0.4-1.2) | 0.09 (-1.4-1.6) |
| CKD due to diabetes mellitus type 1 | Ireland | 5.8 (3.9-8.2) | 0.1 (0.1-0.2) | 8.4 (5.8-11.8) | 0.1 (0.1-0.2) | 0.4 (-0.62-1.43) |
| CKD due to diabetes mellitus type 1 | Israel | 23 (16.4-31.5) | 0.5 (0.4-0.7) | 46.9 (33.4-65.3) | 0.4 (0.3-0.6) | 0.26 (-1.06-1.6) |
| CKD due to diabetes mellitus type 1 | Italy | 133.2 (92.8-189.4) | 0.2 (0.1-0.2) | 123.7 (84-184.5) | 0.1 (0.1-0.2) | 0.08 (-0.95-1.11) |
| CKD due to diabetes mellitus type 1 | Jamaica | 29.2 (22.5-37.5) | 1.7 (1.3-2.2) | 85.7 (57.3-124.5) | 2.8 (1.9-4) | 0.28 (-1.14-1.72) |
| CKD due to diabetes mellitus type 1 | Japan | 631.5 (477.7-806.7) | 0.4 (0.3-0.5) | 611.1 (444.1-828.7) | 0.3 (0.2-0.3) | -0.47 (-1.74-0.82) |
| CKD due to diabetes mellitus type 1 | Jordan | 14.6 (9.8-20.9) | 0.7 (0.5-1.1) | 69.5 (46.7-100.2) | 0.7 (0.4-0.9) | -0.08 (-1.49-1.35) |
| CKD due to diabetes mellitus type 1 | Kazakhstan | 28.3 (19.9-40.4) | 0.2 (0.1-0.3) | 46.7 (31-67) | 0.2 (0.2-0.3) | 1.27 (0.02-2.53) |
| CKD due to diabetes mellitus type 1 | Kenya | 111.5 (76-174.2) | 1.1 (0.7-1.6) | 504 (338.8-734) | 1.6 (1.1-2.4) | 1.29 (0.09-2.5) |
| CKD due to diabetes mellitus type 1 | Kiribati | 3 (2.3-3.8) | 5.7 (4.3-7.4) | 8 (4.7-12.6) | 7.8 (4.6-12.4) | 0.66 (-1.1-2.45) |
| CKD due to diabetes mellitus type 1 | Kuwait | 6 (4.4-8.4) | 0.5 (0.4-0.7) | 10.5 (6.4-15.1) | 0.2 (0.1-0.3) | -1.59 (-2.96--0.19) |
| CKD due to diabetes mellitus type 1 | Kyrgyzstan | 6.2 (4.5-8.5) | 0.2 (0.1-0.2) | 16 (10.9-23.2) | 0.3 (0.2-0.4) | 0.63 (-0.66-1.94) |
| CKD due to diabetes mellitus type 1 | Lao People's Democratic Republic | 141.3 (98-203.5) | 5.1 (3.5-7.5) | 258.2 (168.8-384.8) | 4.1 (2.6-6.1) | -0.44 (-2.18-1.33) |
| CKD due to diabetes mellitus type 1 | Latvia | 9.1 (6.8-12.4) | 0.3 (0.2-0.4) | 18.2 (12.8-26.2) | 0.6 (0.4-0.8) | 1.6 (0.78-2.43) |
| CKD due to diabetes mellitus type 1 | Lebanon | 12.3 (7.4-18.8) | 0.5 (0.3-0.8) | 20.6 (13.8-30.2) | 0.4 (0.2-0.5) | 0.05 (-1.5-1.62) |
| CKD due to diabetes mellitus type 1 | Lesotho | 3.7 (2.3-5.7) | 0.4 (0.2-0.6) | 13.6 (7.7-21) | 1 (0.6-1.6) | 2.97 (1.49-4.47) |
| CKD due to diabetes mellitus type 1 | Liberia | 16.6 (11-25.8) | 1.2 (0.8-1.9) | 41.9 (26.3-61.7) | 1.3 (0.8-1.9) | 0.31 (-1.13-1.77) |
| CKD due to diabetes mellitus type 1 | Libya | 10.1 (6.3-15.3) | 0.4 (0.3-0.6) | 51.2 (25.5-79.7) | 0.7 (0.4-1.1) | 1.91 (0.31-3.53) |
| CKD due to diabetes mellitus type 1 | Lithuania | 9 (6.6-12.2) | 0.2 (0.2-0.3) | 21.2 (14.6-30.8) | 0.5 (0.3-0.7) | 0.81 (0.05-1.57) |
| CKD due to diabetes mellitus type 1 | Luxembourg | 0.9 (0.7-1.3) | 0.2 (0.1-0.3) | 1.6 (1.1-2.3) | 0.2 (0.1-0.2) | 0.67 (-0.49-1.85) |
| CKD due to diabetes mellitus type 1 | Madagascar | 82.2 (54.5-121.2) | 1.3 (0.8-2) | 205.1 (130.7-311.4) | 1.3 (0.8-2) | 0.06 (-1.08-1.21) |
| CKD due to diabetes mellitus type 1 | Malawi | 85.3 (58.3-122.8) | 1.7 (1.2-2.5) | 204.6 (140.6-284) | 2.1 (1.4-2.9) | 0.32 (-0.92-1.57) |
| CKD due to diabetes mellitus type 1 | Malaysia | 282.7 (221.4-353.1) | 2.3 (1.7-2.9) | 847.4 (624.3-1093) | 2.6 (1.9-3.4) | 0.37 (-1.36-2.12) |
| CKD due to diabetes mellitus type 1 | Maldives | 6.2 (4.5-8.1) | 4.7 (3.4-6.3) | 10.1 (7.4-13.8) | 1.9 (1.4-2.6) | -1.64 (-3.39-0.15) |
| CKD due to diabetes mellitus type 1 | Mali | 53.5 (35.8-80.3) | 1.1 (0.7-1.6) | 113.9 (74.3-176.7) | 1 (0.6-1.5) | 0 (-1.39-1.41) |
| CKD due to diabetes mellitus type 1 | Malta | 0.9 (0.6-1.3) | 0.2 (0.1-0.3) | 1.9 (1.2-2.7) | 0.2 (0.2-0.3) | 0.13 (-1.02-1.3) |
| CKD due to diabetes mellitus type 1 | Marshall Islands | 1.4 (0.8-2.4) | 5.9 (3.4-9.9) | 5.3 (1.7-13.9) | 10.3 (3.2-26.9) | 1.32 (-0.48-3.16) |
| CKD due to diabetes mellitus type 1 | Mauritania | 13.5 (8.5-20.4) | 1.2 (0.7-1.8) | 25.2 (15.1-41.2) | 1 (0.5-1.6) | -0.46 (-1.86-0.96) |
| CKD due to diabetes mellitus type 1 | Mauritius | 41.8 (33-52.2) | 4.6 (3.6-5.8) | 154.9 (119.6-197.8) | 8.9 (7-11.1) | 1.7 (-0.29-3.73) |
| CKD due to diabetes mellitus type 1 | Mexico | 682.1 (514-901.2) | 1.3 (1-1.8) | 3933 (2726.2-5533.6) | 2.8 (2-4) | 1.74 (0.49-3.01) |
| CKD due to diabetes mellitus type 1 | Micronesia (Federated States of) | 3.8 (2.4-5.7) | 6 (3.8-9.3) | 9.9 (6.5-14.5) | 10.3 (6.9-15) | 1.29 (-0.56-3.17) |
| CKD due to diabetes mellitus type 1 | Monaco | 0.1 (0-0.1) | 0.1 (0.1-0.2) | 0.1 (0.1-0.2) | 0.2 (0.1-0.2) | 0.98 (-0.04-2.01) |
| CKD due to diabetes mellitus type 1 | Mongolia | 4.4 (2.9-6.4) | 0.3 (0.2-0.5) | 8.9 (5.9-13.6) | 0.3 (0.2-0.4) | -0.51 (-1.92-0.91) |
| CKD due to diabetes mellitus type 1 | Montenegro | 2.1 (1.4-3) | 0.3 (0.2-0.4) | 3.3 (2.1-4.9) | 0.4 (0.2-0.5) | 1.02 (-0.14-2.19) |
| CKD due to diabetes mellitus type 1 | Morocco | 48.8 (28.9-79.3) | 0.3 (0.2-0.5) | 169.4 (105.5-265.4) | 0.4 (0.3-0.7) | 1.55 (-0.11-3.24) |
| CKD due to diabetes mellitus type 1 | Mozambique | 85.9 (57.1-133.4) | 1.1 (0.7-1.8) | 258.5 (169-388.6) | 1.6 (1.1-2.5) | 1.25 (0.05-2.46) |
| CKD due to diabetes mellitus type 1 | Myanmar | 1131.3 (776.7-1551.9) | 3.5 (2.4-4.9) | 1526.6 (1079.2-2099.4) | 2.7 (1.9-3.7) | -0.52 (-2.02-1) |
| CKD due to diabetes mellitus type 1 | Namibia | 4.3 (2.7-7.3) | 0.6 (0.3-0.9) | 11.5 (7.1-18.1) | 0.7 (0.4-1) | 0.35 (-0.96-1.69) |
| CKD due to diabetes mellitus type 1 | Nauru | 0.5 (0.3-0.7) | 7.4 (4.4-11) | 1 (0.6-1.3) | 11.4 (7.5-16) | 0.96 (-0.88-2.83) |
| CKD due to diabetes mellitus type 1 | Nepal | 116.4 (73.5-171.9) | 1 (0.6-1.5) | 336.2 (205.6-506) | 1.3 (0.8-1.9) | 1.02 (0.04-2.02) |
| CKD due to diabetes mellitus type 1 | Netherlands | 17.5 (12.3-24.1) | 0.1 (0.1-0.1) | 26.6 (20.6-34.4) | 0.1 (0.1-0.1) | 0.98 (-0.14-2.11) |
| CKD due to diabetes mellitus type 1 | New Zealand | 3.9 (2.7-5.5) | 0.1 (0.1-0.2) | 8.8 (6-12.5) | 0.1 (0.1-0.2) | 0.92 (-0.14-2) |
| CKD due to diabetes mellitus type 1 | Nicaragua | 25.9 (18.3-38.4) | 1.4 (0.9-2) | 162.2 (107.9-226.4) | 2.9 (1.9-4) | 2.35 (1.14-3.58) |
| CKD due to diabetes mellitus type 1 | Niger | 22.8 (14.8-34.7) | 0.6 (0.4-0.9) | 52.3 (32.4-92.9) | 0.5 (0.3-0.8) | -0.31 (-1.72-1.13) |
| CKD due to diabetes mellitus type 1 | Nigeria | 362.5 (236.1-541.7) | 0.7 (0.4-1) | 746.4 (431-1153.9) | 0.6 (0.3-0.9) | -0.18 (-1.61-1.27) |
| CKD due to diabetes mellitus type 1 | Niue | 0.1 (0.1-0.1) | 4.8 (3.2-6.7) | 0.2 (0.1-0.3) | 8.8 (4.9-14.1) | 1.49 (-0.31-3.33) |
| CKD due to diabetes mellitus type 1 | North Macedonia | 3.6 (2.3-5.2) | 0.2 (0.1-0.3) | 6 (3.6-10) | 0.2 (0.1-0.3) | 0.55 (-0.69-1.82) |
| CKD due to diabetes mellitus type 1 | Northern Mariana Islands | 2.1 (1.4-3) | 5.9 (3.9-8.3) | 5.5 (4.1-7.1) | 8.8 (6.6-11.1) | 1.07 (-0.89-3.06) |
| CKD due to diabetes mellitus type 1 | Norway | 4.1 (2.9-6) | 0.1 (0-0.1) | 6.8 (4.7-9.8) | 0.1 (0.1-0.1) | 1.07 (0.22-1.92) |
| CKD due to diabetes mellitus type 1 | Oman | 3.9 (2.4-6.6) | 0.4 (0.2-0.6) | 18.6 (11.8-28) | 0.5 (0.3-0.9) | 1.99 (0.44-3.57) |
| CKD due to diabetes mellitus type 1 | Pakistan | 835.4 (558.8-1186.5) | 1.3 (0.8-1.8) | 2933.8 (1888.5-4072.9) | 1.8 (1.2-2.5) | 0.75 (-0.32-1.83) |
| CKD due to diabetes mellitus type 1 | Palau | 0.6 (0.4-0.8) | 4.8 (3.1-6.7) | 2 (1.2-2.8) | 8 (5.1-11.4) | 1.48 (-0.3-3.3) |
| CKD due to diabetes mellitus type 1 | Palestine | 5.8 (3.6-8.8) | 0.6 (0.3-0.9) | 17.4 (11.7-25.9) | 0.5 (0.3-0.8) | -0.17 (-1.85-1.54) |
| CKD due to diabetes mellitus type 1 | Panama | 8.9 (6.6-12.2) | 0.5 (0.4-0.7) | 53.9 (36-79.7) | 1.2 (0.8-1.8) | 2.33 (1.17-3.5) |
| CKD due to diabetes mellitus type 1 | Papua New Guinea | 65.2 (36.8-95.6) | 2.3 (1.3-3.4) | 216.4 (157.2-289.1) | 2.6 (1.9-3.5) | 0.36 (-0.92-1.65) |
| CKD due to diabetes mellitus type 1 | Paraguay | 24 (17.1-33) | 1 (0.7-1.3) | 93.3 (60.1-137.6) | 1.5 (0.9-2.2) | 1.14 (-0.09-2.38) |
| CKD due to diabetes mellitus type 1 | Peru | 219.7 (160.1-290.4) | 1.6 (1.1-2.1) | 593.4 (365.1-889.8) | 1.7 (1-2.5) | 0.07 (-1.23-1.38) |
| CKD due to diabetes mellitus type 1 | Philippines | 1295.5 (1050.5-1587.4) | 2.9 (2.3-3.6) | 4612.4 (3467.1-6060.7) | 4.4 (3.3-5.8) | 1.03 (-0.68-2.78) |
| CKD due to diabetes mellitus type 1 | Poland | 156.9 (114.3-218.2) | 0.4 (0.3-0.5) | 89.8 (60.3-137.3) | 0.1 (0.1-0.2) | -1.57 (-2.61--0.52) |
| CKD due to diabetes mellitus type 1 | Portugal | 40 (28.1-55.3) | 0.3 (0.2-0.4) | 46.7 (32.9-66.8) | 0.2 (0.2-0.3) | 0.23 (-0.99-1.47) |
| CKD due to diabetes mellitus type 1 | Puerto Rico | 86.5 (65.6-108.8) | 2.4 (1.8-3.1) | 142.3 (101.7-188.4) | 2.8 (2.1-3.7) | 0.53 (-0.81-1.88) |
| CKD due to diabetes mellitus type 1 | Qatar | 1 (0.5-1.6) | 0.4 (0.2-0.7) | 7.6 (4.9-11.9) | 0.3 (0.2-0.5) | -0.47 (-2.17-1.25) |
| CKD due to diabetes mellitus type 1 | Republic of Korea | 304.1 (232-383) | 0.8 (0.6-1) | 308.7 (219.3-416.2) | 0.3 (0.3-0.5) | -1.32 (-2.43--0.19) |
| CKD due to diabetes mellitus type 1 | Republic of Moldova | 7.4 (5.6-9.8) | 0.2 (0.1-0.2) | 19.6 (13.8-27.4) | 0.3 (0.2-0.5) | 0.5 (-0.13-1.14) |
| CKD due to diabetes mellitus type 1 | Romania | 24.8 (17.2-36.4) | 0.1 (0.1-0.1) | 40.6 (28.9-56) | 0.1 (0.1-0.2) | -0.16 (-1.3-1) |
| CKD due to diabetes mellitus type 1 | Russian Federation | 1195.8 (927.5-1537) | 0.7 (0.5-0.8) | 1431.4 (1025.3-1989.7) | 0.6 (0.4-0.8) | 0 (-0.79-0.8) |
| CKD due to diabetes mellitus type 1 | Rwanda | 76.3 (53.6-107) | 2.1 (1.5-3) | 106.8 (67.8-159) | 1.3 (0.8-2) | -1.4 (-2.65--0.14) |
| CKD due to diabetes mellitus type 1 | Saint Kitts and Nevis | 1.3 (1-1.7) | 4.1 (3.2-5.3) | 3.6 (2.5-5.1) | 4.5 (3.2-6.2) | 1.3 (-0.27-2.89) |
| CKD due to diabetes mellitus type 1 | Saint Lucia | 2.9 (2.3-3.7) | 3.2 (2.5-4.1) | 8.8 (6.4-12) | 3.7 (2.7-5) | 0.46 (-1.02-1.96) |
| CKD due to diabetes mellitus type 1 | Saint Vincent and the Grenadines | 1.8 (1.4-2.2) | 2.4 (1.8-3) | 5.4 (4-7) | 3.9 (2.9-5) | 1.57 (0.14-3.03) |
| CKD due to diabetes mellitus type 1 | Samoa | 5 (3.3-7.2) | 4.8 (3.1-6.9) | 12.2 (8.5-17) | 7.1 (5-9.9) | 0.76 (-0.99-2.54) |
| CKD due to diabetes mellitus type 1 | San Marino | 0 (0-0) | 0.1 (0.1-0.1) | 0 (0-0.1) | 0.1 (0-0.1) | 0.12 (-0.76-1) |
| CKD due to diabetes mellitus type 1 | Sao Tome and Principe | 1 (0.7-1.4) | 1.5 (1-2) | 2.9 (1.8-4.2) | 2 (1.2-2.9) | 0.88 (-0.59-2.37) |
| CKD due to diabetes mellitus type 1 | Saudi Arabia | 79.4 (48.8-121) | 0.9 (0.5-1.4) | 642.5 (378-1001.3) | 1.6 (1-2.4) | 1.45 (-0.22-3.14) |
| CKD due to diabetes mellitus type 1 | Senegal | 51.8 (35-76.6) | 1.3 (0.8-1.9) | 124.4 (81.7-197.6) | 1.3 (0.8-2) | 0.09 (-1.28-1.48) |
| CKD due to diabetes mellitus type 1 | Serbia | 47.7 (31.9-70.9) | 0.4 (0.3-0.6) | 54.2 (36.4-77) | 0.4 (0.3-0.5) | -0.06 (-1.24-1.13) |
| CKD due to diabetes mellitus type 1 | Seychelles | 1.9 (1.5-2.5) | 3.3 (2.5-4.3) | 5.7 (4.2-7.4) | 4.4 (3.3-5.7) | 1.22 (-0.66-3.14) |
| CKD due to diabetes mellitus type 1 | Sierra Leone | 18.5 (11.4-27.1) | 0.8 (0.5-1.2) | 39.3 (24.2-59.2) | 0.8 (0.5-1.2) | -0.09 (-1.43-1.27) |
| CKD due to diabetes mellitus type 1 | Singapore | 22.3 (17.4-28.4) | 0.8 (0.6-1.1) | 33.2 (24.4-44.3) | 0.4 (0.3-0.5) | -0.35 (-1.44-0.75) |
| CKD due to diabetes mellitus type 1 | Slovakia | 20.3 (13.7-29.1) | 0.4 (0.2-0.5) | 19.1 (12.7-29.2) | 0.2 (0.2-0.3) | -0.31 (-1.37-0.76) |
| CKD due to diabetes mellitus type 1 | Slovenia | 2.4 (1.8-3.1) | 0.1 (0.1-0.1) | 2 (1.2-3.2) | 0.1 (0-0.1) | 0.32 (-0.65-1.29) |
| CKD due to diabetes mellitus type 1 | Solomon Islands | 10 (3.2-17.2) | 4.9 (1.7-8.4) | 30.6 (20.4-41.7) | 5.7 (3.8-7.7) | 0.27 (-1.31-1.88) |
| CKD due to diabetes mellitus type 1 | Somalia | 86.5 (54.1-131.8) | 2.4 (1.5-3.7) | 229.8 (141.4-383.7) | 2.5 (1.5-4.3) | 0.26 (-0.96-1.49) |
| CKD due to diabetes mellitus type 1 | South Africa | 167 (121.5-233) | 0.6 (0.5-0.9) | 446.8 (312-628.7) | 0.8 (0.6-1.1) | 1.4 (0.01-2.81) |
| CKD due to diabetes mellitus type 1 | South Sudan | 57.4 (36.8-86.8) | 1.9 (1.2-2.9) | 141.1 (87.5-205.3) | 2.6 (1.6-3.9) | 0.79 (-0.47-2.06) |
| CKD due to diabetes mellitus type 1 | Spain | 124.8 (87.4-175.7) | 0.2 (0.2-0.3) | 105.9 (74.8-149.7) | 0.1 (0.1-0.2) | -0.35 (-1.51-0.83) |
| CKD due to diabetes mellitus type 1 | Sri Lanka | 295.9 (218.9-390.2) | 2.1 (1.5-2.8) | 481.2 (293.5-718.9) | 1.8 (1.1-2.7) | -0.61 (-2.26-1.06) |
| CKD due to diabetes mellitus type 1 | Sudan | 30.4 (17.9-55.5) | 0.3 (0.1-0.5) | 93.3 (55-151.8) | 0.3 (0.2-0.5) | 0.88 (-0.62-2.39) |
| CKD due to diabetes mellitus type 1 | Suriname | 9 (6.7-11.5) | 3 (2.2-3.8) | 30.1 (20.4-42) | 4.5 (3.1-6.3) | 1.34 (-0.12-2.83) |
| CKD due to diabetes mellitus type 1 | Sweden | 11.8 (8.1-17) | 0.1 (0.1-0.1) | 24.7 (16.7-36.1) | 0.1 (0.1-0.2) | 1.71 (0.82-2.6) |
| CKD due to diabetes mellitus type 1 | Switzerland | 7.5 (5.3-10.4) | 0.1 (0.1-0.1) | 9.7 (7.4-12.8) | 0.1 (0-0.1) | 1.13 (0.05-2.23) |
| CKD due to diabetes mellitus type 1 | Syrian Arab Republic | 47.2 (31.3-71.1) | 0.7 (0.4-1) | 99 (60.8-153) | 0.6 (0.4-1) | 0.18 (-1.45-1.84) |
| CKD due to diabetes mellitus type 1 | Taiwan (Province of China) | 352.3 (294.3-428.3) | 1.9 (1.5-2.3) | 497.4 (382.6-628.5) | 1.3 (1.1-1.6) | -0.18 (-1.77-1.43) |
| CKD due to diabetes mellitus type 1 | Tajikistan | 1.8 (1.2-2.7) | 0.1 (0-0.1) | 5 (2.9-8.1) | 0.1 (0-0.1) | 0.12 (-0.59-0.83) |
| CKD due to diabetes mellitus type 1 | Thailand | 1075.4 (805.3-1477) | 2.2 (1.7-3.1) | 2557.8 (1772-3583.9) | 2.6 (1.9-3.7) | -0.03 (-1.7-1.66) |
| CKD due to diabetes mellitus type 1 | Timor-Leste | 11.7 (8-16.8) | 2.4 (1.6-3.6) | 25 (16.5-37.1) | 2.5 (1.6-3.8) | 0.33 (-1.29-1.98) |
| CKD due to diabetes mellitus type 1 | Togo | 15.6 (10.4-23.4) | 0.9 (0.6-1.4) | 54.5 (34.9-84.2) | 1 (0.6-1.6) | 0.18 (-1.16-1.55) |
| CKD due to diabetes mellitus type 1 | Tokelau | 0.1 (0-0.1) | 4.1 (2.5-7.2) | 0.1 (0.1-0.1) | 6.2 (4.5-8.7) | 0.96 (-0.74-2.68) |
| CKD due to diabetes mellitus type 1 | Tonga | 1.6 (1-2.4) | 2.4 (1.6-3.7) | 2.8 (1.8-3.9) | 3.2 (2.1-4.5) | 0.74 (-0.8-2.31) |
| CKD due to diabetes mellitus type 1 | Trinidad and Tobago | 21 (16.8-26.1) | 2.2 (1.8-2.8) | 76.6 (49.4-113.6) | 4.1 (2.7-6) | 1.78 (0.33-3.25) |
| CKD due to diabetes mellitus type 1 | Tunisia | 16.4 (10.2-26.1) | 0.3 (0.2-0.4) | 54.3 (32.6-86.9) | 0.4 (0.2-0.6) | 0.91 (-0.57-2.42) |
| CKD due to diabetes mellitus type 1 | Turkey | 191.6 (125.6-285.2) | 0.4 (0.3-0.7) | 350.7 (229.3-542) | 0.4 (0.2-0.5) | 0.17 (-1.29-1.66) |
| CKD due to diabetes mellitus type 1 | Turkmenistan | 7.3 (5.3-9.8) | 0.3 (0.2-0.4) | 25.3 (16.6-38.2) | 0.5 (0.3-0.7) | 1.2 (-0.22-2.65) |
| CKD due to diabetes mellitus type 1 | Tuvalu | 0.4 (0.3-0.6) | 5.1 (3.6-7.9) | 0.8 (0.6-1.2) | 7.1 (4.9-10.3) | 0.92 (-0.81-2.67) |
| CKD due to diabetes mellitus type 1 | Uganda | 114.5 (73.5-172.3) | 1.5 (0.9-2.2) | 354 (229.6-508.7) | 1.8 (1.1-2.6) | 0.36 (-0.84-1.58) |
| CKD due to diabetes mellitus type 1 | Ukraine | 8.2 (6.2-10.8) | 0 (0-0) | 182.4 (117.4-257.1) | 0.3 (0.2-0.4) | 2.08 (1.66-2.5) |
| CKD due to diabetes mellitus type 1 | United Arab Emirates | 3.7 (2.2-5.9) | 0.4 (0.2-0.6) | 32.9 (16.8-51.8) | 0.4 (0.2-0.6) | 2.89 (1.24-4.57) |
| CKD due to diabetes mellitus type 1 | United Kingdom | 95.3 (67.8-132.7) | 0.1 (0.1-0.2) | 140 (102.9-189.2) | 0.1 (0.1-0.2) | 0.9 (0.05-1.75) |
| CKD due to diabetes mellitus type 1 | United Republic of Tanzania | 94.4 (62.5-141.5) | 0.7 (0.5-1.1) | 238.3 (155.8-367) | 0.7 (0.5-1.1) | 0.05 (-1.44-1.56) |
| CKD due to diabetes mellitus type 1 | United States of America | 661.9 (494.3-863) | 0.2 (0.2-0.3) | 3115.9 (2298.1-4118.2) | 0.6 (0.5-0.8) | 2.19 (0.97-3.43) |
| CKD due to diabetes mellitus type 1 | United States Virgin Islands | 2 (1.4-2.5) | 1.9 (1.4-2.5) | 2.7 (1.7-3.9) | 2.3 (1.6-3.3) | 0.38 (-1.03-1.82) |
| CKD due to diabetes mellitus type 1 | Uruguay | 12.1 (9.2-15.6) | 0.3 (0.3-0.4) | 17.2 (12.1-24.1) | 0.4 (0.3-0.5) | 0.37 (-0.86-1.62) |
| CKD due to diabetes mellitus type 1 | Uzbekistan | 13.4 (9.4-19.2) | 0.1 (0.1-0.1) | 104.7 (72.4-152) | 0.3 (0.2-0.4) | 1.35 (0.01-2.72) |
| CKD due to diabetes mellitus type 1 | Vanuatu | 3.6 (2-6.1) | 3.8 (2.1-6.7) | 15.4 (10-23.5) | 6.3 (4.1-9.7) | 1.05 (-0.62-2.75) |
| CKD due to diabetes mellitus type 1 | Venezuela (Bolivarian Republic of) | 80.4 (58.3-108.8) | 0.7 (0.5-0.9) | 533.5 (325.4-790.1) | 1.7 (1-2.5) | 2.15 (0.97-3.35) |
| CKD due to diabetes mellitus type 1 | Viet Nam | 998.3 (648.2-1384) | 2.1 (1.4-3) | 2337.6 (1529.6-3243.9) | 2.1 (1.3-2.8) | 0.34 (-1.31-2.02) |
| CKD due to diabetes mellitus type 1 | Yemen | 23.1 (12.6-44.7) | 0.4 (0.2-0.7) | 60.1 (33.4-107.7) | 0.3 (0.2-0.5) | 0.09 (-1.24-1.44) |
| CKD due to diabetes mellitus type 1 | Zambia | 83.9 (57-117.2) | 2.2 (1.5-3.2) | 266.4 (161.8-446.8) | 2.6 (1.6-4.4) | 0.09 (-1.15-1.34) |
| CKD due to diabetes mellitus type 1 | Zimbabwe | 27.9 (17.9-45.6) | 0.6 (0.4-0.9) | 102.2 (65-153.3) | 1.1 (0.7-1.6) | 1.63 (0.19-3.08) |
| CKD due to diabetes mellitus type 2 | Afghanistan | 595 (396.8-945) | 9.4 (6.3-15.1) | 851.3 (445.8-1547.3) | 10.9 (5.8-19.8) | 0.36 (-1.22-1.96) |
| CKD due to diabetes mellitus type 2 | Albania | 21.2 (14.9-30.1) | 1.2 (0.9-1.8) | 49.2 (33.4-73.8) | 1.2 (0.8-1.7) | -0.11 (-1.3-1.09) |
| CKD due to diabetes mellitus type 2 | Algeria | 491.7 (303.2-871.9) | 5.8 (3.6-10.8) | 2182.1 (1584.5-2940.5) | 8.1 (6-10.9) | 1.13 (-0.42-2.7) |
| CKD due to diabetes mellitus type 2 | American Samoa | 4 (2.8-5.7) | 23.7 (17-34.4) | 20.8 (16-26.1) | 51.9 (40-64.7) | 2.12 (0.13-4.16) |
| CKD due to diabetes mellitus type 2 | Andorra | 0.9 (0.6-1.4) | 2.1 (1.4-3.1) | 2.9 (1.9-4.4) | 1.6 (1.1-2.5) | -0.33 (-1.44-0.79) |
| CKD due to diabetes mellitus type 2 | Angola | 187.7 (127.2-271.5) | 6.5 (4.6-9.1) | 597.5 (395.9-858.8) | 7.2 (4.8-10.4) | -0.01 (-1.29-1.29) |
| CKD due to diabetes mellitus type 2 | Antigua and Barbuda | 7.6 (6.5-9) | 13.4 (11.6-15.8) | 21.5 (18.5-24.9) | 22.2 (19.1-25.9) | 1.66 (0.11-3.24) |
| CKD due to diabetes mellitus type 2 | Argentina | 1893 (1467.5-2366.4) | 6.2 (4.8-7.6) | 3321 (2595.2-4174.3) | 5.7 (4.5-7.1) | -0.28 (-1.65-1.11) |
| CKD due to diabetes mellitus type 2 | Armenia | 3.1 (2.3-4.1) | 0.1 (0.1-0.2) | 68.7 (49-92.7) | 1.6 (1.1-2.1) | 3.05 (1.96-4.16) |
| CKD due to diabetes mellitus type 2 | Australia | 80.1 (62.6-102.5) | 0.4 (0.3-0.6) | 390 (277.8-540.5) | 0.7 (0.5-1) | 0.64 (-0.47-1.77) |
| CKD due to diabetes mellitus type 2 | Austria | 146.2 (110.5-189.3) | 1.2 (0.9-1.5) | 662.3 (509.2-817) | 2.8 (2.2-3.4) | 2.48 (1.37-3.61) |
| CKD due to diabetes mellitus type 2 | Azerbaijan | 39.7 (27.5-56.7) | 0.8 (0.6-1.2) | 140.5 (95.6-198.8) | 1.5 (1-2.1) | 0.95 (-0.28-2.2) |
| CKD due to diabetes mellitus type 2 | Bahamas | 13.9 (11.8-16.1) | 9.7 (8.3-11.3) | 60.4 (48.3-75.8) | 16.4 (13.3-20.4) | 1.65 (0.24-3.08) |
| CKD due to diabetes mellitus type 2 | Bahrain | 9.9 (6.6-14.3) | 9.1 (6.1-13) | 55.3 (39.9-77) | 11.3 (8-15.9) | 0.24 (-1.54-2.04) |
| CKD due to diabetes mellitus type 2 | Bangladesh | 1661.5 (1271.2-2395.9) | 4 (3.1-5.6) | 4498.9 (3293.5-6445.5) | 3.7 (2.7-5.2) | -0.46 (-1.43-0.52) |
| CKD due to diabetes mellitus type 2 | Barbados | 28.9 (25-33) | 9.3 (8.2-10.6) | 73 (55.5-92.7) | 13.8 (10.5-17.5) | 1.28 (-0.15-2.73) |
| CKD due to diabetes mellitus type 2 | Belarus | 9.3 (6.9-12.4) | 0.1 (0.1-0.1) | 41.8 (30-58.2) | 0.3 (0.2-0.3) | 0.89 (0.42-1.36) |
| CKD due to diabetes mellitus type 2 | Belgium | 239.6 (178.4-316.6) | 1.5 (1.1-1.9) | 470.3 (330.8-650.4) | 1.5 (1.1-2) | 0.39 (-0.7-1.49) |
| CKD due to diabetes mellitus type 2 | Belize | 9.7 (8.5-11.2) | 10.7 (9.3-12.4) | 54.4 (45.1-64.5) | 20.3 (17-23.8) | 2.06 (0.57-3.56) |
| CKD due to diabetes mellitus type 2 | Benin | 90.9 (66.4-123) | 5.2 (3.9-7) | 234.2 (167.6-325.4) | 5.8 (4.2-8.1) | 0.07 (-1.35-1.51) |
| CKD due to diabetes mellitus type 2 | Bermuda | 4.2 (3.6-4.8) | 7.2 (6.3-8.3) | 11.2 (8.9-14.2) | 7.4 (5.9-9.4) | 0.27 (-1-1.56) |
| CKD due to diabetes mellitus type 2 | Bhutan | 10.6 (7.2-16.2) | 5.3 (3.6-7.9) | 36.8 (23.4-53.9) | 6.6 (4.2-9.6) | 0.51 (-0.52-1.54) |
| CKD due to diabetes mellitus type 2 | Bolivia (Plurinational State of) | 443.2 (341.6-591.1) | 16.5 (12.9-21.6) | 1883 (1352-2602.1) | 24 (17.5-32.6) | 0.81 (-0.57-2.21) |
| CKD due to diabetes mellitus type 2 | Bosnia and Herzegovina | 54.2 (38.9-74.7) | 1.6 (1.1-2.2) | 130.4 (93.7-174) | 2 (1.4-2.7) | 0.25 (-0.8-1.32) |
| CKD due to diabetes mellitus type 2 | Botswana | 12.8 (8.4-19.4) | 3 (2-4.4) | 43.4 (29.5-61.9) | 3.8 (2.6-5.4) | 0.61 (-0.72-1.96) |
| CKD due to diabetes mellitus type 2 | Brazil | 4642.6 (3919.3-5436.7) | 6.2 (5.2-7.3) | 17369.9 (14533.3-20606.3) | 7.2 (6-8.5) | 0.03 (-1.14-1.21) |
| CKD due to diabetes mellitus type 2 | Brunei Darussalam | 13.4 (10.7-17.7) | 17.6 (14-22.8) | 37.8 (30.2-47) | 17.1 (13.6-21.1) | 0.39 (-0.97-1.77) |
| CKD due to diabetes mellitus type 2 | Bulgaria | 75.9 (54.6-101.2) | 0.7 (0.6-1) | 280.8 (202.7-378.8) | 1.9 (1.4-2.5) | 2.31 (1.12-3.51) |
| CKD due to diabetes mellitus type 2 | Burkina Faso | 167.1 (117.8-231.5) | 4.8 (3.6-6.6) | 394.7 (283.1-549.8) | 5.4 (3.9-7.4) | 0.35 (-1.12-1.85) |
| CKD due to diabetes mellitus type 2 | Burundi | 211 (153.1-289.1) | 10.8 (7.9-14.8) | 355.3 (235.1-523.5) | 10 (6.7-15) | -0.71 (-1.9-0.5) |
| CKD due to diabetes mellitus type 2 | Cabo Verde | 6 (4.4-8.1) | 2.5 (1.9-3.3) | 21.3 (13-29.9) | 5.2 (3.2-7.3) | 1.32 (0.1-2.56) |
| CKD due to diabetes mellitus type 2 | Cambodia | 266.8 (199.7-349.5) | 6.7 (5.1-8.7) | 800.5 (560.7-1100.2) | 7.6 (5.4-10.2) | 0.02 (-1.47-1.53) |
| CKD due to diabetes mellitus type 2 | Cameroon | 336.1 (225.2-474.2) | 9.7 (6.6-13.4) | 1008.7 (652.5-1496.3) | 10.6 (6.9-15.3) | 0 (-1.39-1.42) |
| CKD due to diabetes mellitus type 2 | Canada | 561.9 (441-696.8) | 1.7 (1.4-2.2) | 1952.6 (1499.2-2563.2) | 2.4 (1.8-3.1) | 0.85 (-0.23-1.94) |
| CKD due to diabetes mellitus type 2 | Central African Republic | 77.6 (53.8-110.3) | 8.9 (6.4-12.3) | 144 (93.9-209) | 9.1 (6.3-13.2) | 0.03 (-1.24-1.3) |
| CKD due to diabetes mellitus type 2 | Chad | 101.1 (70.4-146) | 4.1 (2.9-5.8) | 225.6 (153-341.8) | 5 (3.4-7.5) | 0.35 (-0.98-1.69) |
| CKD due to diabetes mellitus type 2 | Chile | 272.9 (212.7-347.9) | 3 (2.4-3.8) | 1057.5 (806.9-1349) | 4 (3.1-5.1) | 0.78 (-0.52-2.1) |
| CKD due to diabetes mellitus type 2 | China | 43537.4 (35987.5-53065.3) | 6.8 (5.7-8.3) | 107652.1 (84625.7-134047.4) | 5.6 (4.5-7) | -0.72 (-2-0.58) |
| CKD due to diabetes mellitus type 2 | Colombia | 619.4 (490.5-767.1) | 4.1 (3.3-5.1) | 1970.8 (1457.2-2617.3) | 3.5 (2.6-4.7) | -0.42 (-1.5-0.67) |
| CKD due to diabetes mellitus type 2 | Comoros | 14.9 (10.6-20.6) | 9.8 (7.1-13.2) | 42.3 (28.5-61.2) | 10.7 (7.2-15) | 0.04 (-1.21-1.3) |
| CKD due to diabetes mellitus type 2 | Congo | 89.3 (60.8-122) | 10.9 (7.3-14.7) | 214 (133.9-300.1) | 11.1 (6.9-15.8) | -0.26 (-1.56-1.06) |
| CKD due to diabetes mellitus type 2 | Cook Islands | 1.2 (1-1.7) | 11.8 (9.3-15.9) | 3.8 (2.9-4.7) | 15 (11.4-18.4) | 0.51 (-1.07-2.1) |
| CKD due to diabetes mellitus type 2 | Costa Rica | 62.9 (49.9-78.2) | 3.9 (3.1-4.8) | 422.2 (330.4-535.8) | 7.6 (5.9-9.7) | 1.71 (0.57-2.86) |
| CKD due to diabetes mellitus type 2 | C么te d'Ivoire | 132.2 (95-184.7) | 4.7 (3.4-6.3) | 411.8 (279-583.5) | 5 (3.5-6.9) | -0.03 (-1.6-1.56) |
| CKD due to diabetes mellitus type 2 | Croatia | 59 (43.5-77.5) | 1.1 (0.8-1.4) | 174.2 (122.4-241.8) | 1.7 (1.2-2.3) | 0.64 (-0.48-1.79) |
| CKD due to diabetes mellitus type 2 | Cuba | 356.8 (313.7-406.2) | 3.6 (3.2-4.1) | 1583.1 (1327.3-1895.9) | 7.7 (6.5-9.3) | 1.64 (0.47-2.83) |
| CKD due to diabetes mellitus type 2 | Cyprus | 33.6 (22.9-47.7) | 7 (5-10.2) | 61 (43.3-84.8) | 3.7 (2.6-5.1) | -1.78 (-3.25--0.28) |
| CKD due to diabetes mellitus type 2 | Czechia | 127 (92.2-168.8) | 0.9 (0.7-1.2) | 201.8 (145.2-274.6) | 0.8 (0.6-1.2) | -0.28 (-1.25-0.7) |
| CKD due to diabetes mellitus type 2 | Democratic People's Republic of Korea | 929.6 (660.6-1254.9) | 7.2 (5.1-10.1) | 2183.2 (1674-2857.1) | 7.1 (5.5-9.6) | 0.03 (-1.31-1.39) |
| CKD due to diabetes mellitus type 2 | Democratic Republic of the Congo | 998.4 (711-1383.1) | 8.8 (6.6-11.9) | 2445.4 (1604.2-3594.7) | 9.2 (6-13.7) | -0.04 (-1.29-1.23) |
| CKD due to diabetes mellitus type 2 | Denmark | 69.2 (52.6-90.3) | 0.8 (0.6-1) | 303 (241.8-376.9) | 2.1 (1.7-2.6) | 1.76 (0.73-2.8) |
| CKD due to diabetes mellitus type 2 | Djibouti | 7.5 (5.2-10.8) | 8.2 (5.8-11.4) | 52.6 (35.9-76.1) | 12.3 (8.8-17.3) | 0.96 (-0.27-2.19) |
| CKD due to diabetes mellitus type 2 | Dominica | 8.9 (7.4-10.7) | 15 (12.5-17.8) | 18.6 (14.2-23.4) | 23.3 (18.2-29) | 1.41 (-0.14-2.97) |
| CKD due to diabetes mellitus type 2 | Dominican Republic | 191.7 (152.1-251.6) | 6.2 (4.9-8.1) | 911.4 (591-1233.4) | 9.4 (6.1-12.7) | 1.45 (0.16-2.76) |
| CKD due to diabetes mellitus type 2 | Ecuador | 395.2 (327.8-466.4) | 8.6 (7.2-10.2) | 2525.2 (1848.1-3558) | 16.5 (12.3-23) | 1.51 (0.09-2.95) |
| CKD due to diabetes mellitus type 2 | Egypt | 1812.1 (1225.6-2850.5) | 9.3 (6.2-14.8) | 6532.9 (4670.5-9047.2) | 14 (10.1-19.5) | 1.2 (-0.56-3) |
| CKD due to diabetes mellitus type 2 | El Salvador | 147.7 (111.6-226.2) | 5.2 (3.9-8) | 986.2 (658-1344.1) | 15.5 (10.3-21.3) | 3.01 (1.78-4.26) |
| CKD due to diabetes mellitus type 2 | Equatorial Guinea | 12.8 (9.1-17.8) | 8.3 (6.1-11.3) | 42.3 (22.7-63.8) | 11.5 (6.1-17.3) | 0.84 (-0.46-2.16) |
| CKD due to diabetes mellitus type 2 | Eritrea | 68.9 (47.1-102.3) | 8.8 (6-12.8) | 204.8 (132-338.4) | 10.9 (7.1-17.7) | 0.39 (-0.79-1.58) |
| CKD due to diabetes mellitus type 2 | Estonia | 9.8 (7.2-13.1) | 0.5 (0.4-0.6) | 85.2 (64.6-109.6) | 2.5 (1.9-3.1) | 1.82 (0.87-2.77) |
| CKD due to diabetes mellitus type 2 | Eswatini | 13.4 (9.4-19) | 5.9 (4.3-8.3) | 37.3 (22-55.7) | 8.3 (5-12) | 1.27 (-0.14-2.7) |
| CKD due to diabetes mellitus type 2 | Ethiopia | 4141.6 (3244.9-5141.5) | 27.4 (21.9-33) | 5989 (4758.3-7316.2) | 17.5 (13.9-21.4) | -1.95 (-3.2--0.69) |
| CKD due to diabetes mellitus type 2 | Fiji | 49.5 (33.8-73.7) | 17.9 (12.5-27.2) | 191.3 (140.7-251.9) | 32.1 (24-41.3) | 0.97 (-0.82-2.78) |
| CKD due to diabetes mellitus type 2 | Finland | 56.2 (42.9-71.2) | 0.8 (0.6-1) | 214.2 (163.8-265) | 1.3 (1-1.5) | 0.89 (0.12-1.68) |
| CKD due to diabetes mellitus type 2 | France | 1137.6 (842.8-1511.8) | 1.2 (0.9-1.6) | 2439.1 (1845.1-3080.6) | 1.2 (0.9-1.5) | 0.15 (-0.84-1.16) |
| CKD due to diabetes mellitus type 2 | Gabon | 49.8 (35.7-68.8) | 10.2 (7.4-13.9) | 121 (60.7-179.5) | 15.4 (7.4-22.8) | 0.93 (-0.43-2.3) |
| CKD due to diabetes mellitus type 2 | Gambia | 13.5 (9.5-19.3) | 4.9 (3.6-6.9) | 51.9 (34.9-74.4) | 6.5 (4.4-9.6) | 0.53 (-0.87-1.94) |
| CKD due to diabetes mellitus type 2 | Georgia | 24.8 (16.9-36.1) | 0.4 (0.3-0.6) | 115.2 (82.4-161.8) | 1.9 (1.3-2.6) | 2.41 (1.21-3.64) |
| CKD due to diabetes mellitus type 2 | Germany | 1882.4 (1392.2-2509.6) | 1.4 (1-1.8) | 6662.1 (4622.8-9474.7) | 2.6 (1.8-3.6) | 2 (0.83-3.19) |
| CKD due to diabetes mellitus type 2 | Ghana | 254.9 (175.6-375.4) | 5.5 (3.9-8.1) | 1199.3 (870.6-1678.5) | 9.6 (7-13.5) | 1.59 (0.23-2.97) |
| CKD due to diabetes mellitus type 2 | Greece | 627.8 (467.4-825) | 4.3 (3.3-5.7) | 1437.4 (1137.8-1725.7) | 4.4 (3.6-5.1) | -0.45 (-1.79-0.9) |
| CKD due to diabetes mellitus type 2 | Greenland | 0.8 (0.6-1) | 3.4 (2.6-4.3) | 2 (1.4-2.7) | 3.7 (2.6-5) | 0.58 (-0.65-1.82) |
| CKD due to diabetes mellitus type 2 | Grenada | 11.4 (9.6-13.5) | 14.9 (12.6-17.7) | 27.9 (23.2-33.6) | 26.7 (22-31.8) | 1.63 (0.08-3.22) |
| CKD due to diabetes mellitus type 2 | Guam | 5.8 (4.9-7.4) | 10.7 (9-13.2) | 24.3 (20-28.4) | 11.1 (9.1-13.1) | 0.92 (-0.51-2.37) |
| CKD due to diabetes mellitus type 2 | Guatemala | 192.3 (148-240.8) | 7.4 (5.8-9.2) | 1133.1 (840.7-1527.6) | 11.2 (8.4-14.9) | 1.94 (0.65-3.24) |
| CKD due to diabetes mellitus type 2 | Guinea | 94.3 (65-134.3) | 3.3 (2.3-4.7) | 171.3 (114.5-254.9) | 3.7 (2.5-5.5) | 0.15 (-1.34-1.67) |
| CKD due to diabetes mellitus type 2 | Guinea-Bissau | 22.9 (16.3-32) | 7.2 (5.1-9.8) | 35.9 (25.7-51.3) | 6.8 (4.9-9.5) | -0.3 (-1.69-1.11) |
| CKD due to diabetes mellitus type 2 | Guyana | 43.6 (36.8-51.5) | 13.1 (11.2-15.5) | 143.3 (110.9-184) | 24.6 (19.1-31.1) | 2.57 (1.09-4.07) |
| CKD due to diabetes mellitus type 2 | Haiti | 316.9 (217.6-550.2) | 11.6 (7.9-20.7) | 777.2 (415.3-1712.9) | 13.1 (7.1-28.4) | 0.34 (-1.01-1.7) |
| CKD due to diabetes mellitus type 2 | Honduras | 46.6 (33.6-64.7) | 2.6 (1.9-3.6) | 324.5 (222.1-447.6) | 5.9 (4.1-8) | 1.9 (0.81-3) |
| CKD due to diabetes mellitus type 2 | Hungary | 82.8 (62.5-109) | 0.6 (0.4-0.8) | 246.5 (179.9-337.7) | 1.1 (0.8-1.5) | 1.37 (0.32-2.43) |
| CKD due to diabetes mellitus type 2 | Iceland | 1.2 (0.9-1.6) | 0.4 (0.3-0.5) | 4.4 (3.3-5.7) | 0.6 (0.5-0.8) | 0.97 (0.14-1.8) |
| CKD due to diabetes mellitus type 2 | India | 15340.1 (12049.4-19023.2) | 3.9 (3.1-4.8) | 56201.7 (42578.3-70450.2) | 5.2 (4-6.5) | 0.37 (-0.59-1.34) |
| CKD due to diabetes mellitus type 2 | Indonesia | 4692.5 (3753.2-6230.6) | 5.5 (4.4-7.5) | 15859.9 (12207.2-20633.6) | 7.8 (6.1-10.6) | 0.58 (-0.87-2.05) |
| CKD due to diabetes mellitus type 2 | Iran (Islamic Republic of) | 869 (612.2-1218.6) | 4.5 (3.1-6.4) | 3432.9 (2549.9-4341.7) | 5.1 (3.8-6.6) | 0.34 (-1.09-1.79) |
| CKD due to diabetes mellitus type 2 | Iraq | 654.9 (471.3-907.3) | 8.9 (6.4-12.5) | 2079.3 (1297.9-2912.1) | 11.1 (7.1-15.5) | 0.09 (-1.4-1.6) |
| CKD due to diabetes mellitus type 2 | Ireland | 55.2 (41.6-73.3) | 1.4 (1.1-1.9) | 126.3 (88.1-172.8) | 1.4 (1-2) | 0.4 (-0.62-1.43) |
| CKD due to diabetes mellitus type 2 | Israel | 210.9 (158.8-278.3) | 4.7 (3.5-6) | 603.9 (445.7-802) | 4.3 (3.1-5.6) | 0.26 (-1.06-1.6) |
| CKD due to diabetes mellitus type 2 | Italy | 1203.3 (905.5-1593.9) | 1.3 (1-1.7) | 2874 (1974.1-4074.5) | 1.4 (1-1.9) | 0.08 (-0.95-1.11) |
| CKD due to diabetes mellitus type 2 | Jamaica | 188.9 (158.6-220) | 10.1 (8.6-11.8) | 380.3 (277.6-503.7) | 11.9 (8.7-15.9) | 0.28 (-1.14-1.72) |
| CKD due to diabetes mellitus type 2 | Japan | 6430.9 (5189-7844.4) | 4.1 (3.3-5.1) | 18934.3 (13475-24887.2) | 3.4 (2.5-4.3) | -0.47 (-1.74-0.82) |
| CKD due to diabetes mellitus type 2 | Jordan | 108.5 (81.3-142.5) | 10.7 (7.9-14.3) | 635.5 (470.8-843.5) | 11.6 (8.6-15.5) | -0.08 (-1.49-1.35) |
| CKD due to diabetes mellitus type 2 | Kazakhstan | 85 (62.4-112.5) | 0.7 (0.5-0.9) | 311.4 (225.2-429.9) | 1.9 (1.4-2.7) | 1.27 (0.02-2.53) |
| CKD due to diabetes mellitus type 2 | Kenya | 460.4 (328.5-724.1) | 7.1 (5.1-11.2) | 1855.8 (1330.5-2497.2) | 11 (7.9-14.7) | 1.29 (0.09-2.5) |
| CKD due to diabetes mellitus type 2 | Kiribati | 5.7 (4.6-7.1) | 19.1 (15.2-24) | 15.5 (10-23.1) | 28 (18.3-40.4) | 0.66 (-1.1-2.45) |
| CKD due to diabetes mellitus type 2 | Kuwait | 32.2 (25.2-40.5) | 7.1 (5.4-9.1) | 83.3 (57.1-113.1) | 3.8 (2.5-5.3) | -1.59 (-2.96--0.19) |
| CKD due to diabetes mellitus type 2 | Kyrgyzstan | 12.7 (9.2-17.3) | 0.4 (0.3-0.6) | 60.1 (43.1-80.7) | 1.3 (1-1.8) | 0.63 (-0.66-1.94) |
| CKD due to diabetes mellitus type 2 | Lao People's Democratic Republic | 286.3 (210.4-390.2) | 15.9 (11.8-21.3) | 600.4 (406.8-839.6) | 15.5 (10.5-21.2) | -0.44 (-2.18-1.33) |
| CKD due to diabetes mellitus type 2 | Latvia | 8 (5.8-10.5) | 0.2 (0.2-0.3) | 49.1 (35.4-67.3) | 1 (0.7-1.4) | 1.6 (0.78-2.43) |
| CKD due to diabetes mellitus type 2 | Lebanon | 123 (81.1-171.5) | 6.7 (4.6-9.6) | 431.6 (318.7-581.8) | 6.6 (4.9-8.8) | 0.05 (-1.5-1.62) |
| CKD due to diabetes mellitus type 2 | Lesotho | 15.7 (10.7-23.4) | 2.1 (1.4-3) | 43.7 (27.1-66.8) | 4.8 (3-7.2) | 2.97 (1.49-4.47) |
| CKD due to diabetes mellitus type 2 | Liberia | 63.7 (45.3-89) | 6.7 (4.8-9.1) | 116 (79.4-167.4) | 7.4 (5-10.4) | 0.31 (-1.13-1.77) |
| CKD due to diabetes mellitus type 2 | Libya | 87.1 (58.3-128.9) | 5.2 (3.5-7.6) | 395.2 (234.1-561.6) | 9 (5.4-12.7) | 1.91 (0.31-3.53) |
| CKD due to diabetes mellitus type 2 | Lithuania | 7.6 (5.6-9.9) | 0.2 (0.1-0.2) | 44.9 (33.3-61.7) | 0.7 (0.5-0.9) | 0.81 (0.05-1.57) |
| CKD due to diabetes mellitus type 2 | Luxembourg | 9 (6.8-11.9) | 1.7 (1.3-2.2) | 27.1 (18.8-37.9) | 2.2 (1.5-3) | 0.67 (-0.49-1.85) |
| CKD due to diabetes mellitus type 2 | Madagascar | 296.8 (208.7-446.9) | 7.3 (5.2-11) | 591.5 (395.5-842.6) | 7.7 (5.3-10.6) | 0.06 (-1.08-1.21) |
| CKD due to diabetes mellitus type 2 | Malawi | 293.6 (211.2-396.5) | 10 (7.5-13.2) | 687.7 (505.2-916.3) | 12.2 (9.1-16.1) | 0.32 (-0.92-1.57) |
| CKD due to diabetes mellitus type 2 | Malaysia | 757.4 (627.5-907.6) | 9.1 (7.5-10.9) | 3040.5 (2445.6-3664.5) | 12.1 (9.7-14.4) | 0.37 (-1.36-2.12) |
| CKD due to diabetes mellitus type 2 | Maldives | 12.6 (9.6-17.5) | 18.1 (13.9-26.6) | 31.1 (24-39.4) | 11.4 (8.8-14.5) | -1.64 (-3.39-0.15) |
| CKD due to diabetes mellitus type 2 | Mali | 166.9 (115.8-234.1) | 5.5 (4-7.4) | 376.8 (269-550.6) | 5.6 (4.1-8.2) | 0 (-1.39-1.41) |
| CKD due to diabetes mellitus type 2 | Malta | 8 (6.1-10.5) | 2.1 (1.6-2.7) | 25.2 (18-34) | 2.2 (1.6-2.9) | 0.13 (-1.02-1.3) |
| CKD due to diabetes mellitus type 2 | Marshall Islands | 2.8 (1.7-5) | 20.8 (12.8-38.6) | 9.1 (2.8-27.2) | 34.8 (10.9-104.7) | 1.32 (-0.48-3.16) |
| CKD due to diabetes mellitus type 2 | Mauritania | 53.8 (36.4-77.2) | 6.4 (4.4-9) | 111.6 (67.9-170.3) | 6.3 (3.9-9.3) | -0.46 (-1.86-0.96) |
| CKD due to diabetes mellitus type 2 | Mauritius | 111.9 (95.8-128.6) | 17.7 (15.3-20.3) | 584.8 (507.5-662.4) | 32.5 (28.5-36.7) | 1.7 (-0.29-3.73) |
| CKD due to diabetes mellitus type 2 | Mexico | 3174.4 (2565.1-3831.3) | 9.4 (7.6-11.5) | 16033.5 (12632.9-19981.6) | 13.3 (10.6-16.5) | 1.74 (0.49-3.01) |
| CKD due to diabetes mellitus type 2 | Micronesia (Federated States of) | 8.8 (6-14.3) | 21.6 (14.5-35.9) | 21.6 (15.4-30.8) | 37.7 (27.2-54.1) | 1.29 (-0.56-3.17) |
| CKD due to diabetes mellitus type 2 | Monaco | 0.9 (0.6-1.3) | 1.1 (0.7-1.5) | 2.2 (1.5-3.1) | 1.7 (1.2-2.4) | 0.98 (-0.04-2.01) |
| CKD due to diabetes mellitus type 2 | Mongolia | 20 (14-28.3) | 2 (1.4-2.9) | 34.7 (24.2-47.3) | 1.8 (1.3-2.4) | -0.51 (-1.92-0.91) |
| CKD due to diabetes mellitus type 2 | Montenegro | 7.7 (5.5-10.8) | 1.3 (0.9-1.9) | 19.1 (12.7-27.6) | 2.1 (1.4-3.1) | 1.02 (-0.14-2.19) |
| CKD due to diabetes mellitus type 2 | Morocco | 459.2 (273.9-858.5) | 3.6 (2.2-7) | 1995.3 (1402.9-2777.3) | 6.6 (4.7-9.1) | 1.55 (-0.11-3.24) |
| CKD due to diabetes mellitus type 2 | Mozambique | 301 (216.2-459.1) | 7.1 (5.2-10.5) | 770.2 (546.3-1202.4) | 10 (6.9-14.8) | 1.25 (0.05-2.46) |
| CKD due to diabetes mellitus type 2 | Myanmar | 1775 (1290-2431.4) | 8.8 (6.5-11.9) | 3794.2 (2910.6-5013.9) | 8.9 (6.9-12) | -0.52 (-2.02-1) |
| CKD due to diabetes mellitus type 2 | Namibia | 14.7 (9.7-21.6) | 2.9 (1.9-4.1) | 39.6 (26.7-58.5) | 3.6 (2.5-5.2) | 0.35 (-0.96-1.69) |
| CKD due to diabetes mellitus type 2 | Nauru | 0.9 (0.5-1.5) | 26.3 (16.7-45.7) | 1.8 (1.2-2.9) | 40.6 (27.4-72.1) | 0.96 (-0.88-2.83) |
| CKD due to diabetes mellitus type 2 | Nepal | 324.4 (229.7-455.5) | 4.1 (2.9-5.7) | 1282.6 (884.9-1818.1) | 6.2 (4.3-8.8) | 1.02 (0.04-2.02) |
| CKD due to diabetes mellitus type 2 | Netherlands | 187.6 (137.9-255.8) | 0.9 (0.7-1.2) | 640.5 (487.2-809.4) | 1.6 (1.2-2) | 0.98 (-0.14-2.11) |
| CKD due to diabetes mellitus type 2 | New Zealand | 30.2 (22.9-39) | 0.8 (0.6-1) | 116.2 (88-149.4) | 1.3 (1-1.6) | 0.92 (-0.14-2) |
| CKD due to diabetes mellitus type 2 | Nicaragua | 84.4 (64.1-119.6) | 6.2 (4.7-8.8) | 552.1 (409.2-758.3) | 12.2 (9.1-16.8) | 2.35 (1.14-3.58) |
| CKD due to diabetes mellitus type 2 | Niger | 64.2 (43.3-94.3) | 3.1 (2.1-4.4) | 176.3 (116.7-291.6) | 2.9 (1.9-4.5) | -0.31 (-1.72-1.13) |
| CKD due to diabetes mellitus type 2 | Nigeria | 1565.6 (1106.3-2108.2) | 4.4 (3.2-6) | 2823.3 (1947.9-3854.1) | 4.2 (2.9-5.8) | -0.18 (-1.61-1.27) |
| CKD due to diabetes mellitus type 2 | Niue | 0.5 (0.4-0.6) | 19.8 (15.1-26.6) | 0.7 (0.4-1.1) | 36.3 (21.3-55.8) | 1.49 (-0.31-3.33) |
| CKD due to diabetes mellitus type 2 | North Macedonia | 17.4 (12.5-23.8) | 1 (0.7-1.4) | 43.4 (28.9-63.7) | 1.5 (1-2.2) | 0.55 (-0.69-1.82) |
| CKD due to diabetes mellitus type 2 | Northern Mariana Islands | 3 (2.3-3.9) | 27 (21.9-34.2) | 17 (13.8-21.4) | 42.5 (34.3-51) | 1.07 (-0.89-3.06) |
| CKD due to diabetes mellitus type 2 | Norway | 38.3 (28.9-51) | 0.5 (0.4-0.6) | 108.5 (77.5-148.9) | 0.9 (0.6-1.2) | 1.07 (0.22-1.92) |
| CKD due to diabetes mellitus type 2 | Oman | 29.2 (19.7-43.4) | 5.3 (3.5-7.7) | 138.1 (99.3-191.5) | 9.9 (7.1-13.3) | 1.99 (0.44-3.57) |
| CKD due to diabetes mellitus type 2 | Pakistan | 2836.1 (2073-3862) | 5.7 (4.1-7.7) | 8299.9 (6160.2-11022.2) | 8.3 (6.2-11) | 0.75 (-0.32-1.83) |
| CKD due to diabetes mellitus type 2 | Palau | 1.5 (1.1-2) | 19.4 (14.1-25.6) | 5.5 (3.9-7.6) | 33.2 (23.7-44.3) | 1.48 (-0.3-3.3) |
| CKD due to diabetes mellitus type 2 | Palestine | 66.8 (45.5-96.7) | 9.1 (6.2-13.2) | 171.3 (129.3-231.1) | 9 (6.7-11.9) | -0.17 (-1.85-1.54) |
| CKD due to diabetes mellitus type 2 | Panama | 41.3 (32.3-51.5) | 3 (2.4-3.7) | 321.7 (234.2-434.6) | 7.2 (5.2-9.7) | 2.33 (1.17-3.5) |
| CKD due to diabetes mellitus type 2 | Papua New Guinea | 96 (63.2-140.2) | 6.7 (4.6-9.7) | 327.1 (239.7-462.7) | 8.2 (6.1-12.2) | 0.36 (-0.92-1.65) |
| CKD due to diabetes mellitus type 2 | Paraguay | 117.2 (92.1-148.5) | 5.8 (4.6-7.4) | 512.7 (360.9-705.6) | 9.5 (6.7-13.1) | 1.14 (-0.09-2.38) |
| CKD due to diabetes mellitus type 2 | Peru | 1038.7 (823.5-1297.7) | 9.7 (7.7-12.1) | 3969.8 (2658.8-5528.8) | 12 (8-16.8) | 0.07 (-1.23-1.38) |
| CKD due to diabetes mellitus type 2 | Philippines | 2481.7 (2039-3025.9) | 11.4 (9.5-13.5) | 10925.3 (8757.7-13662.2) | 15.2 (12.3-18.7) | 1.03 (-0.68-2.78) |
| CKD due to diabetes mellitus type 2 | Poland | 669 (498.6-870.7) | 1.6 (1.2-2) | 724.7 (547.9-965.7) | 0.9 (0.7-1.2) | -1.57 (-2.61--0.52) |
| CKD due to diabetes mellitus type 2 | Portugal | 304.7 (232.1-395.9) | 2.4 (1.8-3.1) | 939.8 (661.5-1287.5) | 2.8 (2-3.8) | 0.23 (-0.99-1.47) |
| CKD due to diabetes mellitus type 2 | Puerto Rico | 520.4 (451.4-593.6) | 15.1 (13.2-17.1) | 1382.7 (1144.2-1635.1) | 16.2 (13.5-19.3) | 0.53 (-0.81-1.88) |
| CKD due to diabetes mellitus type 2 | Qatar | 5.3 (3.1-8.4) | 9.1 (5-15.1) | 40.5 (28.8-57.7) | 9 (6.6-12.5) | -0.47 (-2.17-1.25) |
| CKD due to diabetes mellitus type 2 | Republic of Korea | 1263.5 (1068.7-1460.6) | 6 (5.1-7.1) | 4208.6 (3262.8-5249.8) | 4.6 (3.5-5.7) | -1.32 (-2.43--0.19) |
| CKD due to diabetes mellitus type 2 | Republic of Moldova | 6.2 (4.4-8.2) | 0.2 (0.1-0.2) | 21.1 (15-29.9) | 0.3 (0.2-0.5) | 0.5 (-0.13-1.14) |
| CKD due to diabetes mellitus type 2 | Romania | 88.8 (64.1-130.9) | 0.4 (0.3-0.5) | 272.4 (213.9-344.1) | 0.7 (0.5-0.8) | -0.16 (-1.3-1) |
| CKD due to diabetes mellitus type 2 | Russian Federation | 806.5 (596.8-1060.6) | 0.5 (0.4-0.6) | 2404 (1847.6-3134.7) | 1 (0.8-1.3) | 0 (-0.79-0.8) |
| CKD due to diabetes mellitus type 2 | Rwanda | 239.4 (172.7-325.8) | 10.9 (8.1-14.5) | 414.2 (285.9-608) | 8.9 (6.3-13.1) | -1.4 (-2.65--0.14) |
| CKD due to diabetes mellitus type 2 | Saint Kitts and Nevis | 7.3 (6.2-8.5) | 19 (16.4-22) | 15.7 (12.5-19.3) | 27.4 (22.3-32.8) | 1.3 (-0.27-2.89) |
| CKD due to diabetes mellitus type 2 | Saint Lucia | 13 (11.1-14.9) | 16.4 (14.2-18.8) | 41.7 (33.7-50.9) | 17.9 (14.5-21.9) | 0.46 (-1.02-1.96) |
| CKD due to diabetes mellitus type 2 | Saint Vincent and the Grenadines | 7.9 (6.7-9.1) | 11.6 (9.9-13.5) | 23.9 (20-28.7) | 17.9 (15-21.2) | 1.57 (0.14-3.03) |
| CKD due to diabetes mellitus type 2 | Samoa | 14.4 (10.4-19.8) | 20.4 (14.7-27.6) | 35.4 (26.6-46.1) | 28.4 (21.5-36.4) | 0.76 (-0.99-2.54) |
| CKD due to diabetes mellitus type 2 | San Marino | 0.4 (0.3-0.5) | 0.9 (0.7-1.3) | 0.8 (0.5-1.2) | 0.7 (0.4-1.1) | 0.12 (-0.76-1) |
| CKD due to diabetes mellitus type 2 | Sao Tome and Principe | 4.9 (3.7-6.4) | 8.6 (6.7-11.4) | 10.6 (7-14.4) | 12.3 (8-17) | 0.88 (-0.59-2.37) |
| CKD due to diabetes mellitus type 2 | Saudi Arabia | 538.4 (367.3-775) | 11.1 (7.7-16) | 2814.9 (1951.5-3813.8) | 20.1 (14-26.8) | 1.45 (-0.22-3.14) |
| CKD due to diabetes mellitus type 2 | Senegal | 181 (129.7-240.4) | 6.8 (5-8.9) | 478.8 (335.7-696.6) | 7.7 (5.4-11) | 0.09 (-1.28-1.48) |
| CKD due to diabetes mellitus type 2 | Serbia | 201 (144.3-276.3) | 2.3 (1.6-3.1) | 420.9 (313.9-535.1) | 2.4 (1.8-3) | -0.06 (-1.24-1.13) |
| CKD due to diabetes mellitus type 2 | Seychelles | 6.4 (5.2-7.9) | 11.3 (9.3-14) | 19.8 (15.3-24.6) | 19.2 (15-23.9) | 1.22 (-0.66-3.14) |
| CKD due to diabetes mellitus type 2 | Sierra Leone | 76.1 (53.6-107.5) | 4.3 (3.1-6) | 130.3 (87.4-188.1) | 4.3 (2.9-6.2) | -0.09 (-1.43-1.27) |
| CKD due to diabetes mellitus type 2 | Singapore | 118.4 (102-135.7) | 6.6 (5.7-7.5) | 416 (344.4-491) | 5.1 (4.2-6) | -0.35 (-1.44-0.75) |
| CKD due to diabetes mellitus type 2 | Slovakia | 81.1 (58.1-108) | 1.4 (1-1.8) | 120.3 (86.1-168.3) | 1.2 (0.9-1.7) | -0.31 (-1.37-0.76) |
| CKD due to diabetes mellitus type 2 | Slovenia | 14.9 (11.8-18.3) | 0.6 (0.5-0.8) | 37.3 (24.7-56.2) | 0.7 (0.5-1) | 0.32 (-0.65-1.29) |
| CKD due to diabetes mellitus type 2 | Solomon Islands | 14.6 (6.6-23.3) | 13.4 (7.5-20.5) | 42.4 (33-54.5) | 14.9 (12-18.9) | 0.27 (-1.31-1.88) |
| CKD due to diabetes mellitus type 2 | Somalia | 208.3 (139.2-312.1) | 12.5 (8.6-18.6) | 582.8 (370.6-886.4) | 13.5 (9.1-20.1) | 0.26 (-0.96-1.49) |
| CKD due to diabetes mellitus type 2 | South Africa | 492.7 (363.4-709.5) | 2.8 (2.1-4) | 1626 (1233.2-2175.5) | 4.2 (3.2-5.6) | 1.4 (0.01-2.81) |
| CKD due to diabetes mellitus type 2 | South Sudan | 250.6 (169-384.9) | 11.6 (8.1-17.3) | 452.4 (296.2-648.1) | 15.9 (10.9-22.5) | 0.79 (-0.47-2.06) |
| CKD due to diabetes mellitus type 2 | Spain | 1240.5 (934.1-1642.9) | 2.3 (1.7-3) | 2584.3 (1796.5-3648.1) | 1.8 (1.3-2.5) | -0.35 (-1.51-0.83) |
| CKD due to diabetes mellitus type 2 | Sri Lanka | 831.8 (637.6-1060.4) | 9.6 (7.3-12.1) | 2006.8 (1320.7-2852) | 8 (5.3-11.1) | -0.61 (-2.26-1.06) |
| CKD due to diabetes mellitus type 2 | Sudan | 228.6 (137.8-423.4) | 2.8 (1.7-5.3) | 699.6 (468.8-1036.5) | 4.3 (2.9-6.4) | 0.88 (-0.62-2.39) |
| CKD due to diabetes mellitus type 2 | Suriname | 28.3 (23.5-34.9) | 12.1 (10.2-14.8) | 111.3 (79.1-146.9) | 18.4 (13.2-24) | 1.34 (-0.12-2.83) |
| CKD due to diabetes mellitus type 2 | Sweden | 119.5 (88.8-157.7) | 0.7 (0.5-0.9) | 475.4 (336.9-653.4) | 1.6 (1.2-2.2) | 1.71 (0.82-2.6) |
| CKD due to diabetes mellitus type 2 | Switzerland | 118.5 (88.3-157.3) | 1 (0.8-1.3) | 395.1 (288.7-504.8) | 1.6 (1.2-2) | 1.13 (0.05-2.23) |
| CKD due to diabetes mellitus type 2 | Syrian Arab Republic | 346.4 (246.9-479.1) | 7.9 (5.6-10.9) | 1012.2 (654.4-1478.6) | 9.6 (6.2-14) | 0.18 (-1.45-1.84) |
| CKD due to diabetes mellitus type 2 | Taiwan (Province of China) | 1620.2 (1444.2-1808.4) | 14 (12.4-15.6) | 5228.4 (4422-6041.2) | 11.7 (10-13.5) | -0.18 (-1.77-1.43) |
| CKD due to diabetes mellitus type 2 | Tajikistan | 5.2 (3.7-8.2) | 0.2 (0.1-0.3) | 14.9 (9.6-22.3) | 0.3 (0.2-0.4) | 0.12 (-0.59-0.83) |
| CKD due to diabetes mellitus type 2 | Thailand | 2615.7 (2105.3-3406.9) | 9 (7.2-11.6) | 12608.3 (9428.2-16657.5) | 11.5 (8.6-15.2) | -0.03 (-1.7-1.66) |
| CKD due to diabetes mellitus type 2 | Timor-Leste | 19.5 (14.3-28.2) | 8.9 (6.7-12.5) | 80 (55.1-117.6) | 10.5 (7.2-15.2) | 0.33 (-1.29-1.98) |
| CKD due to diabetes mellitus type 2 | Togo | 44.9 (32.3-60.8) | 4.7 (3.5-6.5) | 153.1 (103.6-218.5) | 5.5 (3.9-7.9) | 0.18 (-1.16-1.55) |
| CKD due to diabetes mellitus type 2 | Tokelau | 0.2 (0.1-0.4) | 16.1 (11.1-28.1) | 0.3 (0.2-0.5) | 23.6 (16.7-36) | 0.96 (-0.74-2.68) |
| CKD due to diabetes mellitus type 2 | Tonga | 4.2 (2.9-5.7) | 8.8 (6.2-12.2) | 10.6 (7.5-14) | 14 (9.8-18.3) | 0.74 (-0.8-2.31) |
| CKD due to diabetes mellitus type 2 | Trinidad and Tobago | 79.7 (68.6-90.8) | 10.8 (9.4-12.3) | 341.7 (250.8-468) | 17.7 (13.1-24.2) | 1.78 (0.33-3.25) |
| CKD due to diabetes mellitus type 2 | Tunisia | 179.4 (119.5-279.2) | 4.4 (3-6.7) | 813.6 (537.5-1175.7) | 6.8 (4.5-9.9) | 0.91 (-0.57-2.42) |
| CKD due to diabetes mellitus type 2 | Turkey | 1858.4 (1339.3-2689.8) | 6.4 (4.6-9.3) | 5924.7 (4408-7995.1) | 6.9 (5.1-9.4) | 0.17 (-1.29-1.66) |
| CKD due to diabetes mellitus type 2 | Turkmenistan | 20.4 (15.2-26.7) | 1.1 (0.8-1.4) | 90.2 (62.5-126.1) | 2.4 (1.7-3.3) | 1.2 (-0.22-2.65) |
| CKD due to diabetes mellitus type 2 | Tuvalu | 1 (0.7-1.6) | 17.6 (13-28.9) | 2.4 (1.7-3.6) | 26.6 (19-39.5) | 0.92 (-0.81-2.67) |
| CKD due to diabetes mellitus type 2 | Uganda | 474.8 (333.8-666.7) | 9.3 (6.7-13) | 1302.1 (919.1-1845.1) | 11.8 (8.5-16.5) | 0.36 (-0.84-1.58) |
| CKD due to diabetes mellitus type 2 | Ukraine | 7.7 (5.7-10) | 0 (0-0) | 151.4 (104-209.2) | 0.2 (0.1-0.3) | 2.08 (1.66-2.5) |
| CKD due to diabetes mellitus type 2 | United Arab Emirates | 17.5 (11.1-26.7) | 5.9 (3.7-8.9) | 165.4 (105.4-240.8) | 10.1 (5.3-14.7) | 2.89 (1.24-4.57) |
| CKD due to diabetes mellitus type 2 | United Kingdom | 758.6 (571.1-984.7) | 0.8 (0.6-1) | 1703.8 (1250.1-2304.4) | 1.1 (0.8-1.4) | 0.9 (0.05-1.75) |
| CKD due to diabetes mellitus type 2 | United Republic of Tanzania | 379.7 (262.8-547.9) | 4.5 (3.2-6.3) | 993.3 (722.3-1423.9) | 4.9 (3.6-7.1) | 0.05 (-1.44-1.56) |
| CKD due to diabetes mellitus type 2 | United States of America | 7647.8 (6090-9494.7) | 2.3 (1.8-2.8) | 55205.9 (47673.1-61988.8) | 8.8 (7.7-9.9) | 2.19 (0.97-3.43) |
| CKD due to diabetes mellitus type 2 | United States Virgin Islands | 6 (4.9-7.4) | 8.6 (7.1-10.5) | 15.8 (10.6-21.6) | 8.7 (6-11.8) | 0.38 (-1.03-1.82) |
| CKD due to diabetes mellitus type 2 | Uruguay | 101.5 (91.7-109.9) | 2.6 (2.3-2.8) | 224.1 (171.8-294) | 3.4 (2.7-4.4) | 0.37 (-0.86-1.62) |
| CKD due to diabetes mellitus type 2 | Uzbekistan | 73.9 (43.4-130.5) | 0.7 (0.4-1.3) | 453.1 (337.1-604.6) | 2 (1.5-2.7) | 1.35 (0.01-2.72) |
| CKD due to diabetes mellitus type 2 | Vanuatu | 6.7 (4.4-12.2) | 14.3 (9.6-24.8) | 30.3 (21.7-46.1) | 22.2 (16.1-33.5) | 1.05 (-0.62-2.75) |
| CKD due to diabetes mellitus type 2 | Venezuela (Bolivarian Republic of) | 297 (231.5-371.1) | 3.5 (2.7-4.4) | 2615.3 (1867.2-3786.3) | 9.1 (6.5-13.1) | 2.15 (0.97-3.35) |
| CKD due to diabetes mellitus type 2 | Viet Nam | 3018.4 (2155.5-4080.2) | 8.3 (5.9-11.1) | 8536.5 (6083.3-11204.9) | 10 (7-13) | 0.34 (-1.31-2.02) |
| CKD due to diabetes mellitus type 2 | Yemen | 162.8 (100-313.7) | 4 (2.4-8) | 472.3 (297.4-768.8) | 4.1 (2.6-6.8) | 0.09 (-1.24-1.44) |
| CKD due to diabetes mellitus type 2 | Zambia | 278.4 (208-370.1) | 12.7 (9.6-16.7) | 771.4 (540.9-1108.9) | 14.9 (10.7-20.7) | 0.09 (-1.15-1.34) |
| CKD due to diabetes mellitus type 2 | Zimbabwe | 107.5 (73.9-154.7) | 3.4 (2.4-4.8) | 279.1 (186-402) | 5.3 (3.6-7.8) | 1.63 (0.19-3.08) |
| CKD due to glomerulonephritis | Afghanistan | 287.7 (189.3-450.8) | 4.1 (2.7-6.6) | 505.7 (286.5-878.9) | 4.3 (2.4-7.9) | 0.36 (-1.22-1.96) |
| CKD due to glomerulonephritis | Albania | 43.3 (31.6-56.3) | 2.1 (1.5-2.7) | 68.4 (46.9-94.6) | 1.7 (1.2-2.3) | -0.11 (-1.3-1.09) |
| CKD due to glomerulonephritis | Algeria | 219.9 (147.1-376.3) | 1.9 (1.2-3.5) | 739.2 (530.6-974.6) | 2.4 (1.7-3.3) | 1.13 (-0.42-2.7) |
| CKD due to glomerulonephritis | American Samoa | 0.2 (0.2-0.4) | 0.8 (0.5-1.2) | 0.7 (0.5-1) | 1.6 (1.1-2.2) | 2.12 (0.13-4.16) |
| CKD due to glomerulonephritis | Andorra | 0.9 (0.6-1.3) | 1.8 (1.2-2.7) | 2.3 (1.5-3.4) | 1.4 (0.9-2) | -0.33 (-1.44-0.79) |
| CKD due to glomerulonephritis | Angola | 450.6 (321.4-602.4) | 9.5 (6.7-12.8) | 1246 (905.9-1708.9) | 9.3 (6.3-12.6) | -0.01 (-1.29-1.29) |
| CKD due to glomerulonephritis | Antigua and Barbuda | 1.7 (1.4-2) | 3 (2.5-3.7) | 4.7 (3.7-5.8) | 4.7 (3.8-5.7) | 1.66 (0.11-3.24) |
| CKD due to glomerulonephritis | Argentina | 1031.7 (789.7-1317.4) | 3.3 (2.6-4.2) | 1504.5 (1123.5-1928) | 2.7 (2-3.4) | -0.28 (-1.65-1.11) |
| CKD due to glomerulonephritis | Armenia | 4.2 (3.2-5.4) | 0.1 (0.1-0.2) | 56.2 (39.9-77.5) | 1.4 (1-1.9) | 3.05 (1.96-4.16) |
| CKD due to glomerulonephritis | Australia | 450.8 (395.6-505.3) | 2.5 (2.1-2.8) | 1285.8 (955.6-1648.2) | 2.5 (1.9-3.2) | 0.64 (-0.47-1.77) |
| CKD due to glomerulonephritis | Austria | 143.5 (110.1-182) | 1.2 (0.9-1.5) | 479.4 (369.2-614.2) | 2.1 (1.7-2.6) | 2.48 (1.37-3.61) |
| CKD due to glomerulonephritis | Azerbaijan | 58.4 (40.7-80.1) | 1 (0.7-1.4) | 149 (98.6-212.7) | 1.5 (1-2.1) | 0.95 (-0.28-2.2) |
| CKD due to glomerulonephritis | Bahamas | 6.1 (5.1-7.2) | 3 (2.4-3.6) | 18.8 (14.1-24.7) | 4.6 (3.4-5.9) | 1.65 (0.24-3.08) |
| CKD due to glomerulonephritis | Bahrain | 4.5 (3-6.4) | 2.9 (2-4.3) | 22.5 (16.2-30.5) | 3.5 (2.5-4.8) | 0.24 (-1.54-2.04) |
| CKD due to glomerulonephritis | Bangladesh | 1692.2 (1065.2-2247.3) | 2.2 (1.7-2.8) | 2179 (1579.5-3040.1) | 1.7 (1.2-2.3) | -0.46 (-1.43-0.52) |
| CKD due to glomerulonephritis | Barbados | 5.9 (5-7) | 2.2 (1.9-2.6) | 13.3 (9.6-18.1) | 3 (2.2-4.1) | 1.28 (-0.15-2.73) |
| CKD due to glomerulonephritis | Belarus | 36.1 (30.7-41.9) | 0.3 (0.3-0.3) | 123.3 (95.3-157.7) | 0.8 (0.6-1.1) | 0.89 (0.42-1.36) |
| CKD due to glomerulonephritis | Belgium | 180 (132.8-233.2) | 1.1 (0.9-1.5) | 316.5 (220-435.2) | 1.1 (0.8-1.5) | 0.39 (-0.7-1.49) |
| CKD due to glomerulonephritis | Belize | 4.2 (3.7-4.9) | 3.1 (2.6-3.7) | 19.8 (16.3-23.8) | 5.5 (4.4-6.6) | 2.06 (0.57-3.56) |
| CKD due to glomerulonephritis | Benin | 192.3 (152.7-236.6) | 7.5 (5.8-9.5) | 480.1 (365.6-619.1) | 7.5 (5.6-9.9) | 0.07 (-1.35-1.51) |
| CKD due to glomerulonephritis | Bermuda | 0.9 (0.7-1.1) | 1.5 (1.2-1.8) | 1.6 (1.2-2.2) | 1.4 (1.1-1.8) | 0.27 (-1-1.56) |
| CKD due to glomerulonephritis | Bhutan | 6.8 (4.4-9.7) | 2.4 (1.6-3.5) | 17.2 (10.7-25) | 2.8 (1.7-4.1) | 0.51 (-0.52-1.54) |
| CKD due to glomerulonephritis | Bolivia (Plurinational State of) | 289.6 (228-373.3) | 6.9 (5.3-9.1) | 742.6 (519.2-1018.9) | 8 (5.8-11.1) | 0.81 (-0.57-2.21) |
| CKD due to glomerulonephritis | Bosnia and Herzegovina | 72.2 (54.8-92.7) | 1.8 (1.4-2.4) | 112.1 (83.1-143.5) | 1.8 (1.4-2.3) | 0.25 (-0.8-1.32) |
| CKD due to glomerulonephritis | Botswana | 27.5 (18.7-41.6) | 4.8 (3.2-7.1) | 80.7 (56.8-119.5) | 5.6 (4-8.2) | 0.61 (-0.72-1.96) |
| CKD due to glomerulonephritis | Brazil | 3304.8 (2833-3817) | 3.1 (2.6-3.6) | 6675.9 (5361.2-8122.2) | 2.7 (2.2-3.3) | 0.03 (-1.14-1.21) |
| CKD due to glomerulonephritis | Brunei Darussalam | 3.4 (2.5-4.6) | 3.1 (2.2-4.4) | 8.3 (6.2-10.7) | 2.9 (2.1-3.7) | 0.39 (-0.97-1.77) |
| CKD due to glomerulonephritis | Bulgaria | 161.6 (124-199) | 1.6 (1.3-1.9) | 480.1 (351.8-643.6) | 3.6 (2.7-4.7) | 2.31 (1.12-3.51) |
| CKD due to glomerulonephritis | Burkina Faso | 394.9 (309.2-503.3) | 7.8 (5.8-10) | 962.2 (735.2-1216.9) | 8.6 (6.4-11.1) | 0.35 (-1.12-1.85) |
| CKD due to glomerulonephritis | Burundi | 335.7 (245.1-445.5) | 12.7 (9.1-17.1) | 558.3 (392.3-819.6) | 10.7 (7.2-15.8) | -0.71 (-1.9-0.5) |
| CKD due to glomerulonephritis | Cabo Verde | 7.8 (6.1-9.7) | 3 (2.3-3.8) | 22.7 (14.6-30.5) | 5 (3.2-6.7) | 1.32 (0.1-2.56) |
| CKD due to glomerulonephritis | Cambodia | 103 (72.4-135.4) | 1.1 (0.9-1.5) | 149.9 (101.1-213.1) | 1 (0.7-1.4) | 0.02 (-1.47-1.53) |
| CKD due to glomerulonephritis | Cameroon | 360.8 (259.9-494.5) | 6.5 (4.6-9) | 993.3 (644.5-1430.4) | 6.1 (4.1-8.8) | 0 (-1.39-1.42) |
| CKD due to glomerulonephritis | Canada | 290.7 (225.5-366.6) | 0.9 (0.7-1.2) | 926 (703.2-1192.2) | 1.3 (1-1.6) | 0.85 (-0.23-1.94) |
| CKD due to glomerulonephritis | Central African Republic | 156.4 (121.3-197.5) | 11.6 (8.8-14.9) | 301.7 (208.8-444.4) | 11.4 (7.9-16.3) | 0.03 (-1.24-1.3) |
| CKD due to glomerulonephritis | Chad | 179.7 (134.1-244.4) | 5.2 (3.8-7.3) | 472.6 (332-690.5) | 6.2 (4.2-9.3) | 0.35 (-0.98-1.69) |
| CKD due to glomerulonephritis | Chile | 172.9 (135.9-216.3) | 1.7 (1.4-2.2) | 491.5 (369.8-637.4) | 1.9 (1.4-2.5) | 0.78 (-0.52-2.1) |
| CKD due to glomerulonephritis | China | 4939.8 (3900-6248.7) | 0.5 (0.4-0.6) | 5320.1 (3751.9-7179.9) | 0.3 (0.2-0.4) | -0.72 (-2-0.58) |
| CKD due to glomerulonephritis | Colombia | 960.4 (817.7-1113.4) | 4.9 (4-5.8) | 1969.7 (1518.5-2488.7) | 3.6 (2.7-4.5) | -0.42 (-1.5-0.67) |
| CKD due to glomerulonephritis | Comoros | 26.9 (19.8-35.3) | 12.8 (9.4-16.9) | 63.9 (44.8-84.1) | 13.5 (9.3-18) | 0.04 (-1.21-1.3) |
| CKD due to glomerulonephritis | Congo | 155.3 (112.6-200.8) | 13.3 (9.2-17.2) | 357.8 (234.6-492.1) | 12.4 (8-16.8) | -0.26 (-1.56-1.06) |
| CKD due to glomerulonephritis | Cook Islands | 0 (0-0.1) | 0.3 (0.2-0.4) | 0.1 (0.1-0.1) | 0.3 (0.2-0.5) | 0.51 (-1.07-2.1) |
| CKD due to glomerulonephritis | Costa Rica | 64.9 (53.4-77.4) | 3.5 (2.8-4.2) | 356.4 (284.8-440.6) | 6.5 (5.2-8) | 1.71 (0.57-2.86) |
| CKD due to glomerulonephritis | C么te d'Ivoire | 158.1 (117.9-213.4) | 3 (2.2-4) | 411.9 (285.2-565.2) | 3 (2.1-4.1) | -0.03 (-1.6-1.56) |
| CKD due to glomerulonephritis | Croatia | 119.9 (93.4-149.3) | 2.2 (1.7-2.7) | 271.2 (195.9-366.1) | 2.8 (2.1-3.8) | 0.64 (-0.48-1.79) |
| CKD due to glomerulonephritis | Cuba | 109.7 (93.7-128.3) | 1 (0.9-1.2) | 282.5 (219.7-364.3) | 1.6 (1.3-2.1) | 1.64 (0.47-2.83) |
| CKD due to glomerulonephritis | Cyprus | 29.7 (20.3-41.7) | 5.8 (3.9-8.3) | 50.3 (35.5-69.4) | 2.9 (2.1-3.9) | -1.78 (-3.25--0.28) |
| CKD due to glomerulonephritis | Czechia | 224 (172.5-277.4) | 1.7 (1.3-2) | 268.4 (197.1-357.2) | 1.2 (0.9-1.6) | -0.28 (-1.25-0.7) |
| CKD due to glomerulonephritis | Democratic People's Republic of Korea | 79.2 (52.4-114.2) | 0.4 (0.3-0.6) | 122.7 (83-168.8) | 0.4 (0.3-0.6) | 0.03 (-1.31-1.39) |
| CKD due to glomerulonephritis | Democratic Republic of the Congo | 1724.2 (1285.1-2255.8) | 9.7 (7.1-12.8) | 3957.3 (2791.7-5729.3) | 9.7 (6.6-14.1) | -0.04 (-1.29-1.23) |
| CKD due to glomerulonephritis | Denmark | 63.5 (47.5-81) | 0.8 (0.6-0.9) | 228.6 (183-282.4) | 1.7 (1.4-2.1) | 1.76 (0.73-2.8) |
| CKD due to glomerulonephritis | Djibouti | 13.6 (9.6-18.7) | 9.1 (6.4-12.5) | 74.7 (51-104.9) | 12.5 (8.7-17.4) | 0.96 (-0.27-2.19) |
| CKD due to glomerulonephritis | Dominica | 2.1 (1.7-2.5) | 3.4 (2.8-4.2) | 4.1 (3.1-5.3) | 5.4 (4-7.1) | 1.41 (-0.14-2.97) |
| CKD due to glomerulonephritis | Dominican Republic | 141.3 (119.8-168.5) | 2.5 (2-3) | 344.6 (252.4-439.8) | 3.2 (2.4-4.1) | 1.45 (0.16-2.76) |
| CKD due to glomerulonephritis | Ecuador | 264.7 (229.7-303.7) | 3.9 (3.3-4.6) | 940.2 (635.6-1403.9) | 5.8 (3.9-8.5) | 1.51 (0.09-2.95) |
| CKD due to glomerulonephritis | Egypt | 1050.7 (763-1583.4) | 4 (2.7-6.4) | 2996.7 (2097.4-4012.5) | 5.4 (3.7-7.2) | 1.2 (-0.56-3) |
| CKD due to glomerulonephritis | El Salvador | 232.5 (188.8-326.8) | 6.8 (5.4-9.6) | 1120.2 (788.6-1463.1) | 17.9 (12.6-23.5) | 3.01 (1.78-4.26) |
| CKD due to glomerulonephritis | Equatorial Guinea | 23.3 (17.2-31.2) | 10.7 (7.8-14.3) | 75.1 (43-115.4) | 12.1 (6.8-17.8) | 0.84 (-0.46-2.16) |
| CKD due to glomerulonephritis | Eritrea | 144.4 (100.3-211.7) | 10.6 (7.3-15.2) | 335.6 (212.9-549.6) | 11.6 (7.5-19) | 0.39 (-0.79-1.58) |
| CKD due to glomerulonephritis | Estonia | 35.9 (29.8-43) | 1.9 (1.6-2.3) | 101 (81.9-123.8) | 3.6 (2.9-4.3) | 1.82 (0.87-2.77) |
| CKD due to glomerulonephritis | Eswatini | 24.3 (18-32.9) | 7.7 (5.6-10.6) | 68 (41.1-96.2) | 11 (6.6-15.6) | 1.27 (-0.14-2.7) |
| CKD due to glomerulonephritis | Ethiopia | 2923 (2148.7-3623.5) | 11.8 (8.9-15) | 3420.5 (2662.4-4348.5) | 6.7 (5.1-8.6) | -1.95 (-3.2--0.69) |
| CKD due to glomerulonephritis | Fiji | 2.5 (1.6-3.7) | 0.5 (0.3-0.8) | 6.3 (4.3-9.2) | 0.8 (0.6-1.2) | 0.97 (-0.82-2.78) |
| CKD due to glomerulonephritis | Finland | 34.4 (25.8-43.8) | 0.5 (0.4-0.6) | 111.7 (84.1-143.7) | 0.7 (0.5-0.9) | 0.89 (0.12-1.68) |
| CKD due to glomerulonephritis | France | 956.7 (703.6-1290.8) | 1.1 (0.8-1.4) | 1838.7 (1436.4-2352) | 1 (0.8-1.2) | 0.15 (-0.84-1.16) |
| CKD due to glomerulonephritis | Gabon | 69.1 (51-89.1) | 11.8 (8.8-15.4) | 161 (86.8-224.5) | 15.5 (8-21.7) | 0.93 (-0.43-2.3) |
| CKD due to glomerulonephritis | Gambia | 30.6 (22.6-40.1) | 6.7 (4.9-8.9) | 99.4 (71.3-130.2) | 8.4 (5.9-11.3) | 0.53 (-0.87-1.94) |
| CKD due to glomerulonephritis | Georgia | 31.7 (24.1-42.4) | 0.5 (0.4-0.7) | 94.3 (65.2-131.3) | 1.7 (1.2-2.4) | 2.41 (1.21-3.64) |
| CKD due to glomerulonephritis | Germany | 2143.2 (1614.1-2726) | 1.6 (1.3-2.1) | 6433.6 (4422-8792.1) | 2.6 (1.8-3.5) | 2 (0.83-3.19) |
| CKD due to glomerulonephritis | Ghana | 709.6 (527.5-995.7) | 9.7 (7-13.8) | 2664.6 (1953.5-3468.9) | 14.9 (11-19.7) | 1.59 (0.23-2.97) |
| CKD due to glomerulonephritis | Greece | 418.2 (308-550.2) | 2.9 (2.2-3.7) | 839 (666.4-1039.1) | 2.7 (2.2-3.3) | -0.45 (-1.79-0.9) |
| CKD due to glomerulonephritis | Greenland | 0.6 (0.5-0.8) | 2 (1.5-2.6) | 1.3 (0.9-1.7) | 2.2 (1.6-2.9) | 0.58 (-0.65-1.82) |
| CKD due to glomerulonephritis | Grenada | 3.4 (2.9-4.1) | 4.6 (3.8-5.6) | 6.7 (5.2-8.4) | 6.2 (4.9-7.6) | 1.63 (0.08-3.22) |
| CKD due to glomerulonephritis | Guam | 0.4 (0.3-0.6) | 0.5 (0.4-0.7) | 1.2 (0.9-1.6) | 0.6 (0.4-0.8) | 0.92 (-0.51-2.37) |
| CKD due to glomerulonephritis | Guatemala | 335.7 (289-385.5) | 8.2 (6.8-9.7) | 1470.6 (1156.7-1822.4) | 12.6 (9.8-15.8) | 1.94 (0.65-3.24) |
| CKD due to glomerulonephritis | Guinea | 329.8 (250.3-428.7) | 8.6 (6.3-11.6) | 590.9 (418.6-843.6) | 9 (6.2-13) | 0.15 (-1.34-1.67) |
| CKD due to glomerulonephritis | Guinea-Bissau | 55.4 (42-72.1) | 10.7 (8-14) | 87.9 (64.2-116.1) | 9.3 (6.6-12.2) | -0.3 (-1.69-1.11) |
| CKD due to glomerulonephritis | Guyana | 20.6 (17.1-24.4) | 3.7 (3-4.5) | 50 (34.8-68.3) | 7.1 (5-9.6) | 2.57 (1.09-4.07) |
| CKD due to glomerulonephritis | Haiti | 227.2 (166.3-343.2) | 4.3 (3-7.2) | 433.6 (234.7-1014.4) | 4.2 (2.2-9.8) | 0.34 (-1.01-1.7) |
| CKD due to glomerulonephritis | Honduras | 78.5 (61.6-101) | 3.2 (2.4-4.3) | 366 (253.7-499) | 5.7 (4-7.7) | 1.9 (0.81-3) |
| CKD due to glomerulonephritis | Hungary | 113.8 (88.7-143.2) | 0.8 (0.7-1) | 232.5 (166.3-309.5) | 1.1 (0.8-1.5) | 1.37 (0.32-2.43) |
| CKD due to glomerulonephritis | Iceland | 1.8 (1.3-2.3) | 0.6 (0.4-0.8) | 6.4 (4.9-7.9) | 1 (0.8-1.2) | 0.97 (0.14-1.8) |
| CKD due to glomerulonephritis | India | 9260.4 (7221.4-11782.5) | 1.9 (1.4-2.4) | 24425.5 (18228.7-31095.6) | 2.2 (1.6-2.8) | 0.37 (-0.59-1.34) |
| CKD due to glomerulonephritis | Indonesia | 1722.7 (1236.3-2133.8) | 1 (0.8-1.3) | 2831.8 (2175-3643.3) | 1.1 (0.9-1.4) | 0.58 (-0.87-2.05) |
| CKD due to glomerulonephritis | Iran (Islamic Republic of) | 221.5 (161.2-316.5) | 0.8 (0.5-1.2) | 592.9 (441.3-763.3) | 0.8 (0.6-1.1) | 0.34 (-1.09-1.79) |
| CKD due to glomerulonephritis | Iraq | 270.6 (193.1-379.4) | 2.9 (2-4.1) | 666.9 (392.3-923.5) | 3 (1.8-4.2) | 0.09 (-1.4-1.6) |
| CKD due to glomerulonephritis | Ireland | 49 (36.9-63) | 1.2 (0.9-1.6) | 99.5 (70.7-132.5) | 1.2 (0.8-1.5) | 0.4 (-0.62-1.43) |
| CKD due to glomerulonephritis | Israel | 90.3 (65.8-116.4) | 2 (1.5-2.6) | 229.4 (163.3-308.3) | 1.7 (1.2-2.2) | 0.26 (-1.06-1.6) |
| CKD due to glomerulonephritis | Italy | 1134.1 (853.1-1442.6) | 1.3 (1-1.6) | 2406.5 (1640.7-3344) | 1.3 (0.9-1.7) | 0.08 (-0.95-1.11) |
| CKD due to glomerulonephritis | Jamaica | 49.5 (42.2-57.9) | 2.5 (2.1-3) | 117.5 (84.6-162.6) | 3.8 (2.7-5.3) | 0.28 (-1.14-1.72) |
| CKD due to glomerulonephritis | Japan | 227.6 (161-297.3) | 0.1 (0.1-0.2) | 574.3 (372.5-823.7) | 0.1 (0.1-0.2) | -0.47 (-1.74-0.82) |
| CKD due to glomerulonephritis | Jordan | 53.5 (39.9-72.2) | 3.6 (2.6-5) | 235.9 (174.1-316.1) | 3.5 (2.5-4.8) | -0.08 (-1.49-1.35) |
| CKD due to glomerulonephritis | Kazakhstan | 122.7 (96-151.4) | 0.9 (0.7-1.1) | 282.3 (207.1-380.2) | 1.6 (1.2-2.2) | 1.27 (0.02-2.53) |
| CKD due to glomerulonephritis | Kenya | 696.6 (521.6-1067.6) | 7.7 (5.5-12.3) | 2526.9 (1959.7-3399.1) | 11.1 (8.1-15) | 1.29 (0.09-2.5) |
| CKD due to glomerulonephritis | Kiribati | 0.3 (0.2-0.4) | 0.6 (0.4-0.7) | 0.7 (0.4-1.1) | 0.8 (0.5-1.2) | 0.66 (-1.1-2.45) |
| CKD due to glomerulonephritis | Kuwait | 15.6 (12.4-19.3) | 2.3 (1.7-2.9) | 29.3 (20.8-38.6) | 1.1 (0.7-1.5) | -1.59 (-2.96--0.19) |
| CKD due to glomerulonephritis | Kyrgyzstan | 29.3 (23.1-37.3) | 0.8 (0.6-1) | 79.8 (59.9-102.6) | 1.5 (1.1-1.9) | 0.63 (-0.66-1.94) |
| CKD due to glomerulonephritis | Lao People's Democratic Republic | 70.5 (47.9-94.3) | 2 (1.4-2.7) | 90.9 (59.5-135.6) | 1.5 (1-2.2) | -0.44 (-2.18-1.33) |
| CKD due to glomerulonephritis | Latvia | 31.1 (25.8-37) | 1 (0.8-1.1) | 84.8 (62.9-112.3) | 2.2 (1.7-2.9) | 1.6 (0.78-2.43) |
| CKD due to glomerulonephritis | Lebanon | 49.4 (33.5-69.6) | 2.4 (1.6-3.4) | 140.2 (98.2-190.4) | 2.2 (1.6-3) | 0.05 (-1.5-1.62) |
| CKD due to glomerulonephritis | Lesotho | 35.9 (24.9-49.3) | 4.2 (2.9-5.9) | 111.8 (74-160.6) | 10.1 (6.6-14.5) | 2.97 (1.49-4.47) |
| CKD due to glomerulonephritis | Liberia | 134.1 (100.3-180.5) | 9.3 (6.9-12.5) | 245.1 (169.4-331) | 9.3 (6.5-12.5) | 0.31 (-1.13-1.77) |
| CKD due to glomerulonephritis | Libya | 46.6 (31.6-67.5) | 2.3 (1.5-3.4) | 185.8 (106.8-264.9) | 3.7 (2.1-5.2) | 1.91 (0.31-3.53) |
| CKD due to glomerulonephritis | Lithuania | 39.4 (33.3-45.3) | 0.9 (0.8-1.1) | 104.9 (80-135.8) | 2 (1.5-2.6) | 0.81 (0.05-1.57) |
| CKD due to glomerulonephritis | Luxembourg | 7.9 (6-10.3) | 1.5 (1.2-2) | 21.5 (15.1-29.5) | 1.8 (1.3-2.4) | 0.67 (-0.49-1.85) |
| CKD due to glomerulonephritis | Madagascar | 483.5 (354.4-666.4) | 8.5 (6.1-12.5) | 1010.8 (708.9-1429.3) | 8.3 (5.8-11.6) | 0.06 (-1.08-1.21) |
| CKD due to glomerulonephritis | Malawi | 558.8 (435.7-712.1) | 12.6 (9.5-16.5) | 1193.6 (909.8-1506.8) | 14.4 (11-18.1) | 0.32 (-0.92-1.57) |
| CKD due to glomerulonephritis | Malaysia | 94.5 (73.4-119.1) | 0.8 (0.6-1) | 244 (184-314) | 0.8 (0.6-1.1) | 0.37 (-1.36-2.12) |
| CKD due to glomerulonephritis | Maldives | 2.9 (2.2-3.7) | 1.9 (1.4-2.6) | 3.4 (2.6-4.5) | 0.9 (0.7-1.2) | -1.64 (-3.39-0.15) |
| CKD due to glomerulonephritis | Mali | 360 (278.3-459.3) | 7.7 (5.8-10) | 758.2 (566.5-995.5) | 7.2 (5.3-9.9) | 0 (-1.39-1.41) |
| CKD due to glomerulonephritis | Malta | 7.3 (5.6-9.4) | 1.9 (1.4-2.4) | 20 (14.3-26.8) | 1.9 (1.4-2.4) | 0.13 (-1.02-1.3) |
| CKD due to glomerulonephritis | Marshall Islands | 0.1 (0.1-0.3) | 0.6 (0.3-1.1) | 0.4 (0.1-1.2) | 1 (0.3-2.8) | 1.32 (-0.48-3.16) |
| CKD due to glomerulonephritis | Mauritania | 91.6 (66.3-120.5) | 8.2 (5.8-11) | 167.5 (105.4-237.1) | 7.3 (4.6-10.5) | -0.46 (-1.86-0.96) |
| CKD due to glomerulonephritis | Mauritius | 12.5 (10.1-15.4) | 1.5 (1.2-1.8) | 42.2 (32.5-53.9) | 2.6 (2.1-3.2) | 1.7 (-0.29-3.73) |
| CKD due to glomerulonephritis | Mexico | 3633.8 (3083.3-4204.4) | 8 (6.5-9.6) | 16509.2 (12777.6-21198.3) | 12.9 (10.1-16.4) | 1.74 (0.49-3.01) |
| CKD due to glomerulonephritis | Micronesia (Federated States of) | 0.4 (0.3-0.6) | 0.6 (0.4-1) | 0.8 (0.5-1.2) | 1 (0.7-1.5) | 1.29 (-0.56-3.17) |
| CKD due to glomerulonephritis | Monaco | 0.7 (0.5-1) | 0.9 (0.6-1.2) | 1.6 (1.1-2.3) | 1.4 (1-1.9) | 0.98 (-0.04-2.01) |
| CKD due to glomerulonephritis | Mongolia | 27.3 (20-36.7) | 2.2 (1.6-3.1) | 46.7 (33.8-62.6) | 1.9 (1.4-2.6) | -0.51 (-1.92-0.91) |
| CKD due to glomerulonephritis | Montenegro | 18.9 (13.8-24.3) | 3.1 (2.3-4) | 37.3 (26.1-50) | 4.2 (3-5.6) | 1.02 (-0.14-2.19) |
| CKD due to glomerulonephritis | Morocco | 168.7 (108.4-296.8) | 1.1 (0.7-2.1) | 573.3 (390.5-805.7) | 1.8 (1.2-2.5) | 1.55 (-0.11-3.24) |
| CKD due to glomerulonephritis | Mozambique | 541 (413.9-720.1) | 8.1 (5.9-11.5) | 1398.8 (1007.4-1918.5) | 10.9 (7.8-15.1) | 1.25 (0.05-2.46) |
| CKD due to glomerulonephritis | Myanmar | 591 (398.7-811.9) | 1.5 (1.1-2.1) | 588 (430-787.3) | 1.1 (0.8-1.5) | -0.52 (-2.02-1) |
| CKD due to glomerulonephritis | Namibia | 29 (20.4-43.2) | 4.5 (3.1-6.5) | 73 (49.2-101.6) | 5.3 (3.7-7.3) | 0.35 (-0.96-1.69) |
| CKD due to glomerulonephritis | Nauru | 0 (0-0.1) | 0.7 (0.4-1.2) | 0.1 (0.1-0.1) | 1.1 (0.7-1.8) | 0.96 (-0.88-2.83) |
| CKD due to glomerulonephritis | Nepal | 198.1 (144.7-268.5) | 1.9 (1.4-2.7) | 570.2 (393.4-797.9) | 2.6 (1.8-3.6) | 1.02 (0.04-2.02) |
| CKD due to glomerulonephritis | Netherlands | 175.9 (131.7-227) | 0.9 (0.7-1.1) | 498.6 (383.2-630) | 1.3 (1-1.6) | 0.98 (-0.14-2.11) |
| CKD due to glomerulonephritis | New Zealand | 72.8 (57.2-88) | 1.9 (1.5-2.3) | 242.1 (188.3-297.2) | 2.8 (2.2-3.4) | 0.92 (-0.14-2) |
| CKD due to glomerulonephritis | Nicaragua | 149.7 (125.1-197.8) | 7.6 (6.1-10.2) | 742.9 (559.3-939.5) | 14.2 (10.5-17.9) | 2.35 (1.14-3.58) |
| CKD due to glomerulonephritis | Niger | 253.1 (192.1-332.9) | 6.4 (4.7-8.7) | 540.1 (365.4-857) | 5.4 (3.6-8.6) | -0.31 (-1.72-1.13) |
| CKD due to glomerulonephritis | Nigeria | 4407.8 (3358.5-5594) | 8.6 (6.4-11.2) | 8497.2 (5919.5-11313.6) | 8.3 (5.9-11.1) | -0.18 (-1.61-1.27) |
| CKD due to glomerulonephritis | Niue | 0 (0-0) | 0.5 (0.4-0.7) | 0 (0-0) | 1.2 (0.6-1.8) | 1.49 (-0.31-3.33) |
| CKD due to glomerulonephritis | North Macedonia | 35.9 (26.1-47.7) | 2 (1.5-2.7) | 71.4 (49.4-100.5) | 2.4 (1.8-3.3) | 0.55 (-0.69-1.82) |
| CKD due to glomerulonephritis | Northern Mariana Islands | 0.1 (0.1-0.2) | 0.4 (0.3-0.6) | 0.3 (0.2-0.4) | 0.6 (0.4-0.8) | 1.07 (-0.89-3.06) |
| CKD due to glomerulonephritis | Norway | 58.6 (43.1-74.9) | 0.8 (0.6-1) | 146.2 (106.6-192.2) | 1.2 (0.9-1.6) | 1.07 (0.22-1.92) |
| CKD due to glomerulonephritis | Oman | 13.4 (9.2-20.4) | 1.8 (1.2-2.7) | 54.1 (37.8-74.4) | 3 (2-4.1) | 1.99 (0.44-3.57) |
| CKD due to glomerulonephritis | Pakistan | 1628 (1179.4-2229.6) | 2.7 (1.9-3.7) | 4937.5 (3523-6501.3) | 3.7 (2.7-5) | 0.75 (-0.32-1.83) |
| CKD due to glomerulonephritis | Palau | 0.1 (0-0.1) | 0.5 (0.3-0.8) | 0.2 (0.1-0.2) | 0.9 (0.6-1.3) | 1.48 (-0.3-3.3) |
| CKD due to glomerulonephritis | Palestine | 30.8 (21.5-43.7) | 3.4 (2.3-4.8) | 71.8 (53.7-96.5) | 3.1 (2.2-4.2) | -0.17 (-1.85-1.54) |
| CKD due to glomerulonephritis | Panama | 54.4 (45.3-64.4) | 3.4 (2.8-4) | 326.4 (238.5-426.9) | 7.3 (5.4-9.6) | 2.33 (1.17-3.5) |
| CKD due to glomerulonephritis | Papua New Guinea | 8.1 (4.4-11.7) | 0.3 (0.2-0.4) | 25.3 (17.8-35.2) | 0.3 (0.2-0.4) | 0.36 (-0.92-1.65) |
| CKD due to glomerulonephritis | Paraguay | 68.9 (57.4-84.3) | 2.5 (2-3.2) | 212.2 (153.4-280.7) | 3.5 (2.5-4.6) | 1.14 (-0.09-2.38) |
| CKD due to glomerulonephritis | Peru | 679.4 (566.4-817.4) | 4.4 (3.6-5.4) | 1536 (1047.3-2023.8) | 4.4 (3-5.9) | 0.07 (-1.23-1.38) |
| CKD due to glomerulonephritis | Philippines | 712.6 (597.6-854.9) | 1.5 (1.2-1.8) | 1649.5 (1231.1-2069.2) | 1.7 (1.3-2.2) | 1.03 (-0.68-2.78) |
| CKD due to glomerulonephritis | Poland | 1250.1 (1009.5-1527.4) | 3 (2.4-3.6) | 1011.5 (753.1-1320.3) | 1.4 (1.1-1.8) | -1.57 (-2.61--0.52) |
| CKD due to glomerulonephritis | Portugal | 255.2 (192.3-324.7) | 2 (1.5-2.5) | 613.9 (428.7-835.6) | 2 (1.5-2.6) | 0.23 (-0.99-1.47) |
| CKD due to glomerulonephritis | Puerto Rico | 157.1 (129.3-190.1) | 4.5 (3.7-5.4) | 255.3 (209.3-308.2) | 4.3 (3.6-5.1) | 0.53 (-0.81-1.88) |
| CKD due to glomerulonephritis | Qatar | 2.8 (1.5-4.5) | 2.9 (1.6-4.7) | 18.8 (12.8-26.7) | 2.6 (1.8-3.7) | -0.47 (-2.17-1.25) |
| CKD due to glomerulonephritis | Republic of Korea | 1020.4 (872.3-1167.6) | 3.7 (3.1-4.3) | 1062.9 (761.7-1427.2) | 1.2 (0.9-1.6) | -1.32 (-2.43--0.19) |
| CKD due to glomerulonephritis | Republic of Moldova | 31.3 (26.8-36.3) | 0.7 (0.6-0.8) | 73.5 (57.2-93.8) | 1.3 (1-1.7) | 0.5 (-0.13-1.14) |
| CKD due to glomerulonephritis | Romania | 985.7 (813.1-1154.9) | 3.8 (3.2-4.4) | 627.6 (503.4-778.7) | 1.7 (1.4-2.1) | -0.16 (-1.3-1) |
| CKD due to glomerulonephritis | Russian Federation | 3920.3 (3449.3-4406.6) | 2.3 (2-2.6) | 5627.1 (4608.7-6713.1) | 2.4 (2-2.9) | 0 (-0.79-0.8) |
| CKD due to glomerulonephritis | Rwanda | 529.7 (418.2-655.4) | 16.6 (12.5-21.1) | 788.6 (541.6-1082) | 12.7 (8.6-17.4) | -1.4 (-2.65--0.14) |
| CKD due to glomerulonephritis | Saint Kitts and Nevis | 1.9 (1.5-2.3) | 5.1 (4.3-6.2) | 3.4 (2.6-4.4) | 5.4 (4.1-6.9) | 1.3 (-0.27-2.89) |
| CKD due to glomerulonephritis | Saint Lucia | 3.9 (3.3-4.7) | 4.1 (3.4-4.9) | 9.6 (7.3-12.3) | 4.4 (3.4-5.6) | 0.46 (-1.02-1.96) |
| CKD due to glomerulonephritis | Saint Vincent and the Grenadines | 2.7 (2.3-3.1) | 3.3 (2.8-3.9) | 6.1 (4.8-7.4) | 4.7 (3.8-5.8) | 1.57 (0.14-3.03) |
| CKD due to glomerulonephritis | Samoa | 0.6 (0.4-0.9) | 0.6 (0.4-0.8) | 1.3 (0.9-1.7) | 0.8 (0.5-1.1) | 0.76 (-0.99-2.54) |
| CKD due to glomerulonephritis | San Marino | 0.3 (0.2-0.4) | 0.8 (0.6-1.1) | 0.6 (0.3-0.8) | 0.6 (0.4-0.8) | 0.12 (-0.76-1) |
| CKD due to glomerulonephritis | Sao Tome and Principe | 7.3 (5.5-8.9) | 10 (7.6-12.4) | 15.1 (10.6-19.8) | 12.8 (8.7-16.6) | 0.88 (-0.59-2.37) |
| CKD due to glomerulonephritis | Saudi Arabia | 225.8 (155.5-329.7) | 3.4 (2.3-5.1) | 1147.3 (774.8-1597.5) | 5.7 (3.9-8) | 1.45 (-0.22-3.14) |
| CKD due to glomerulonephritis | Senegal | 320.1 (246.6-414.5) | 7.9 (6-10.2) | 680.1 (478.5-966) | 7.9 (5.6-10.9) | 0.09 (-1.28-1.48) |
| CKD due to glomerulonephritis | Serbia | 247.8 (181.7-335.6) | 2.6 (1.9-3.5) | 380.1 (287.7-475.8) | 2.3 (1.8-2.8) | -0.06 (-1.24-1.13) |
| CKD due to glomerulonephritis | Seychelles | 0.6 (0.5-0.8) | 1 (0.8-1.3) | 1.5 (1.1-2) | 1.4 (1-1.8) | 1.22 (-0.66-3.14) |
| CKD due to glomerulonephritis | Sierra Leone | 151.7 (108.3-199.8) | 6 (4.3-8) | 260.5 (186.4-362) | 5.6 (4-7.6) | -0.09 (-1.43-1.27) |
| CKD due to glomerulonephritis | Singapore | 71.6 (58.1-85.5) | 3.4 (2.7-4.1) | 195.9 (147.1-244) | 2.4 (1.8-3) | -0.35 (-1.44-0.75) |
| CKD due to glomerulonephritis | Slovakia | 96.5 (71.4-126.2) | 1.6 (1.2-2.1) | 116.9 (83.6-156.5) | 1.3 (0.9-1.7) | -0.31 (-1.37-0.76) |
| CKD due to glomerulonephritis | Slovenia | 38.4 (31.9-44.5) | 1.6 (1.3-1.8) | 60.5 (42.4-86) | 1.2 (0.8-1.7) | 0.32 (-0.65-1.29) |
| CKD due to glomerulonephritis | Solomon Islands | 0.9 (0.3-1.6) | 0.4 (0.2-0.7) | 2.5 (1.7-3.5) | 0.5 (0.3-0.7) | 0.27 (-1.31-1.88) |
| CKD due to glomerulonephritis | Somalia | 415.3 (294.3-601.3) | 14.7 (10-21.3) | 1100 (707.7-1648.9) | 15.1 (9.7-22.2) | 0.26 (-0.96-1.49) |
| CKD due to glomerulonephritis | South Africa | 1105.1 (878.3-1405) | 4.8 (3.7-6.3) | 2880.2 (2286-3537.9) | 6.4 (5-7.8) | 1.4 (0.01-2.81) |
| CKD due to glomerulonephritis | South Sudan | 359.7 (253.7-501.6) | 12.9 (8.9-18.3) | 715.1 (493.2-958.2) | 17 (11.5-23.2) | 0.79 (-0.47-2.06) |
| CKD due to glomerulonephritis | Spain | 975 (728.3-1260.7) | 1.9 (1.4-2.4) | 1765.9 (1222.9-2445.3) | 1.3 (1-1.8) | -0.35 (-1.51-0.83) |
| CKD due to glomerulonephritis | Sri Lanka | 136.7 (100.5-176.2) | 1.1 (0.8-1.4) | 176.8 (111.7-256.5) | 0.7 (0.5-1) | -0.61 (-2.26-1.06) |
| CKD due to glomerulonephritis | Sudan | 294.9 (201.5-494.5) | 2.6 (1.7-4.9) | 720.5 (493.5-1044.3) | 3.4 (2.3-5) | 0.88 (-0.62-2.39) |
| CKD due to glomerulonephritis | Suriname | 11 (8.8-13.3) | 3.4 (2.7-4.2) | 31 (22.6-41.8) | 5.1 (3.8-6.8) | 1.34 (-0.12-2.83) |
| CKD due to glomerulonephritis | Sweden | 119.2 (88.3-153.8) | 0.7 (0.6-0.9) | 409.2 (290.4-556.2) | 1.5 (1.1-2) | 1.71 (0.82-2.6) |
| CKD due to glomerulonephritis | Switzerland | 89.6 (66.8-116) | 0.8 (0.6-1) | 276 (204.3-361.9) | 1.2 (0.9-1.5) | 1.13 (0.05-2.23) |
| CKD due to glomerulonephritis | Syrian Arab Republic | 200.8 (147.4-273.5) | 3.1 (2.2-4.5) | 404.7 (267.9-576.3) | 3.5 (2.3-4.9) | 0.18 (-1.45-1.84) |
| CKD due to glomerulonephritis | Taiwan (Province of China) | 84 (65.1-104.5) | 0.5 (0.4-0.7) | 158.8 (114.5-207.5) | 0.4 (0.3-0.5) | -0.18 (-1.77-1.43) |
| CKD due to glomerulonephritis | Tajikistan | 10 (7.5-14) | 0.3 (0.2-0.4) | 25.6 (16.7-40.1) | 0.4 (0.2-0.5) | 0.12 (-0.59-0.83) |
| CKD due to glomerulonephritis | Thailand | 460.1 (352.4-597.7) | 1 (0.8-1.3) | 907.7 (632.9-1265.2) | 1 (0.7-1.4) | -0.03 (-1.7-1.66) |
| CKD due to glomerulonephritis | Timor-Leste | 8 (5.3-10.3) | 1.2 (0.9-1.6) | 12.7 (8.9-18.4) | 1.2 (0.8-1.7) | 0.33 (-1.29-1.98) |
| CKD due to glomerulonephritis | Togo | 101.6 (78.8-128.6) | 6.1 (4.6-7.9) | 283.2 (196.3-394.2) | 6.6 (4.7-9.1) | 0.18 (-1.16-1.55) |
| CKD due to glomerulonephritis | Tokelau | 0 (0-0) | 0.4 (0.3-0.8) | 0 (0-0) | 0.8 (0.6-1.2) | 0.96 (-0.74-2.68) |
| CKD due to glomerulonephritis | Tonga | 0.2 (0.1-0.2) | 0.2 (0.1-0.4) | 0.3 (0.2-0.4) | 0.4 (0.2-0.5) | 0.74 (-0.8-2.31) |
| CKD due to glomerulonephritis | Trinidad and Tobago | 25.4 (21.7-29.6) | 2.7 (2.3-3.1) | 77.5 (53.3-102.8) | 4.5 (3.1-5.9) | 1.78 (0.33-3.25) |
| CKD due to glomerulonephritis | Tunisia | 106.7 (72.2-167.6) | 2.2 (1.5-3.5) | 361.3 (232-520) | 3 (1.9-4.2) | 0.91 (-0.57-2.42) |
| CKD due to glomerulonephritis | Turkey | 786.9 (574.6-1122.5) | 2.2 (1.6-3.3) | 1652.4 (1131.9-2231.1) | 1.9 (1.3-2.5) | 0.17 (-1.29-1.66) |
| CKD due to glomerulonephritis | Turkmenistan | 35.7 (28.6-44.3) | 1.4 (1.1-1.8) | 120.2 (86.2-160) | 2.7 (2-3.6) | 1.2 (-0.22-2.65) |
| CKD due to glomerulonephritis | Tuvalu | 0 (0-0.1) | 0.5 (0.4-0.9) | 0.1 (0.1-0.1) | 0.7 (0.5-1.1) | 0.92 (-0.81-2.67) |
| CKD due to glomerulonephritis | Uganda | 656 (465.7-908.5) | 9.3 (6.5-13.2) | 1882.7 (1378.1-2521.5) | 11.4 (8.4-15.5) | 0.36 (-0.84-1.58) |
| CKD due to glomerulonephritis | Ukraine | 30.4 (26-34.9) | 0 (0-0.1) | 575.5 (405.2-783.6) | 0.9 (0.6-1.2) | 2.08 (1.66-2.5) |
| CKD due to glomerulonephritis | United Arab Emirates | 8.3 (5.3-12.7) | 1.7 (1-2.6) | 61.6 (36.3-91.6) | 2.4 (1.3-3.7) | 2.89 (1.24-4.57) |
| CKD due to glomerulonephritis | United Kingdom | 646.1 (491.2-822.9) | 0.7 (0.6-0.9) | 1310.3 (979.6-1689.6) | 0.9 (0.7-1.1) | 0.9 (0.05-1.75) |
| CKD due to glomerulonephritis | United Republic of Tanzania | 1913.9 (1533.8-2470) | 17.6 (13.9-23) | 4511.8 (3515.6-5905.8) | 17.9 (14.1-23.4) | 0.05 (-1.44-1.56) |
| CKD due to glomerulonephritis | United States of America | 3955.5 (3023.1-4990.1) | 1.3 (1-1.6) | 14096.5 (12072.2-16078.9) | 2.5 (2.2-2.8) | 2.19 (0.97-3.43) |
| CKD due to glomerulonephritis | United States Virgin Islands | 1.7 (1.3-2.1) | 1.9 (1.5-2.4) | 2.1 (1.5-3) | 1.7 (1.2-2.3) | 0.38 (-1.03-1.82) |
| CKD due to glomerulonephritis | Uruguay | 72 (65.8-77.9) | 1.9 (1.7-2) | 109.1 (81.6-142) | 1.8 (1.4-2.3) | 0.37 (-0.86-1.62) |
| CKD due to glomerulonephritis | Uzbekistan | 90.5 (63.1-136.9) | 0.7 (0.5-1.1) | 559.3 (420.3-717.8) | 2 (1.5-2.6) | 1.35 (0.01-2.72) |
| CKD due to glomerulonephritis | Vanuatu | 0.3 (0.2-0.6) | 0.4 (0.2-0.7) | 1.3 (0.8-2) | 0.6 (0.4-1) | 1.05 (-0.62-2.75) |
| CKD due to glomerulonephritis | Venezuela (Bolivarian Republic of) | 460.3 (387.4-533.8) | 4.2 (3.4-4.9) | 2941.8 (2068.4-3971.7) | 10 (7-13.5) | 2.15 (0.97-3.35) |
| CKD due to glomerulonephritis | Viet Nam | 502.1 (323.3-663.4) | 0.9 (0.6-1.2) | 852.5 (560.3-1162) | 0.9 (0.6-1.2) | 0.34 (-1.31-2.02) |
| CKD due to glomerulonephritis | Yemen | 91.6 (58.3-165.6) | 1.6 (0.9-3.2) | 231.7 (146-388.9) | 1.6 (1-2.7) | 0.09 (-1.24-1.44) |
| CKD due to glomerulonephritis | Zambia | 494.4 (391-605.1) | 14.7 (11.3-18.3) | 1291.5 (852.4-1969.1) | 16.2 (10.9-23.1) | 0.09 (-1.15-1.34) |
| CKD due to glomerulonephritis | Zimbabwe | 205.5 (149.3-282.7) | 5.1 (3.7-7.1) | 633.7 (440.2-871.4) | 8.6 (6-11.7) | 1.63 (0.19-3.08) |
| CKD due to hypertension | Afghanistan | 896 (605.6-1497.3) | 15.5 (10.6-26.3) | 1370.5 (796.7-2561.1) | 17.9 (10.4-35.4) | 0.36 (-1.22-1.96) |
| CKD due to hypertension | Albania | 24.6 (18.2-32.7) | 1.5 (1.1-1.9) | 56.2 (38.3-80.5) | 1.4 (1-2) | -0.11 (-1.3-1.09) |
| CKD due to hypertension | Algeria | 700.5 (443.7-1306.1) | 9.8 (6.2-17.9) | 3314 (2433.5-4299) | 13.8 (10.4-17.8) | 1.13 (-0.42-2.7) |
| CKD due to hypertension | American Samoa | 0.5 (0.3-0.7) | 2.8 (1.9-4.2) | 2.5 (1.7-3.5) | 6.2 (4.3-8.7) | 2.12 (0.13-4.16) |
| CKD due to hypertension | Andorra | 0.9 (0.6-1.4) | 2.2 (1.4-3.4) | 3.3 (2.1-4.7) | 1.7 (1.2-2.6) | -0.33 (-1.44-0.79) |
| CKD due to hypertension | Angola | 374.5 (265.9-512.7) | 13 (9.7-17) | 1156.5 (784.1-1585) | 13.9 (9.6-18.7) | -0.01 (-1.29-1.29) |
| CKD due to hypertension | Antigua and Barbuda | 5.8 (4.7-6.9) | 10.2 (8.5-12.2) | 14.5 (11.8-17.5) | 15.5 (12.8-18.5) | 1.66 (0.11-3.24) |
| CKD due to hypertension | Argentina | 2665.7 (2149.5-3190.8) | 9.1 (7.4-10.8) | 4687 (3764-5662.6) | 8 (6.5-9.7) | -0.28 (-1.65-1.11) |
| CKD due to hypertension | Armenia | 1.1 (0.8-1.4) | 0 (0-0.1) | 22.7 (16-30.7) | 0.5 (0.4-0.7) | 3.05 (1.96-4.16) |
| CKD due to hypertension | Australia | 303.6 (247.6-360.3) | 1.7 (1.4-2.1) | 1282.6 (937.4-1683.4) | 2.3 (1.7-2.9) | 0.64 (-0.47-1.77) |
| CKD due to hypertension | Austria | 148.2 (112-190.9) | 1.2 (0.9-1.5) | 818.3 (642.2-978.7) | 3.3 (2.6-3.9) | 2.48 (1.37-3.61) |
| CKD due to hypertension | Azerbaijan | 13.3 (9.3-18.9) | 0.3 (0.2-0.4) | 43.1 (28.3-60.8) | 0.5 (0.3-0.7) | 0.95 (-0.28-2.2) |
| CKD due to hypertension | Bahamas | 10.6 (8.7-12.7) | 7.1 (5.7-8.5) | 42.7 (33.2-54.1) | 11.6 (9-14.3) | 1.65 (0.24-3.08) |
| CKD due to hypertension | Bahrain | 14.6 (10.1-20.5) | 15.2 (10.1-21.3) | 83.2 (60.9-106.4) | 19.6 (15-24.5) | 0.24 (-1.54-2.04) |
| CKD due to hypertension | Bangladesh | 1187.6 (892.6-1653.5) | 2.8 (2.1-3.9) | 3012.2 (2194.9-4306.7) | 2.7 (2-3.8) | -0.46 (-1.43-0.52) |
| CKD due to hypertension | Barbados | 19.4 (15.6-23) | 6.4 (5.2-7.6) | 47 (34.4-60) | 9.2 (6.7-11.8) | 1.28 (-0.15-2.73) |
| CKD due to hypertension | Belarus | 11.6 (9.1-14.3) | 0.1 (0.1-0.1) | 44.3 (32.8-56.9) | 0.3 (0.2-0.4) | 0.89 (0.42-1.36) |
| CKD due to hypertension | Belgium | 325 (245.4-420.5) | 2 (1.6-2.6) | 728.8 (517.3-959.6) | 2.2 (1.6-2.8) | 0.39 (-0.7-1.49) |
| CKD due to hypertension | Belize | 7 (5.8-8.2) | 7.5 (6.2-8.9) | 38.3 (30.8-46.2) | 13.8 (11-16.8) | 2.06 (0.57-3.56) |
| CKD due to hypertension | Benin | 288.1 (233-351.1) | 16.6 (13.5-20.1) | 735.7 (577.4-944.1) | 17.9 (14.1-22.4) | 0.07 (-1.35-1.51) |
| CKD due to hypertension | Bermuda | 2.9 (2.4-3.5) | 5.1 (4.2-6) | 7.9 (6.1-9.9) | 5.3 (4-6.6) | 0.27 (-1-1.56) |
| CKD due to hypertension | Bhutan | 7.2 (4.7-10.3) | 3.5 (2.4-5) | 25.2 (16.5-36.6) | 4.5 (3-6.6) | 0.51 (-0.52-1.54) |
| CKD due to hypertension | Bolivia (Plurinational State of) | 353.5 (267.2-481.5) | 13.4 (10.2-17.9) | 1420 (1028.3-1943.5) | 18.8 (13.7-25.1) | 0.81 (-0.57-2.21) |
| CKD due to hypertension | Bosnia and Herzegovina | 34.1 (25.2-45.6) | 1 (0.7-1.3) | 98.2 (71.1-128.9) | 1.5 (1.1-2) | 0.25 (-0.8-1.32) |
| CKD due to hypertension | Botswana | 37.9 (26-54.8) | 9.4 (6.7-13.6) | 126.3 (95.2-180) | 11.6 (8.8-16.3) | 0.61 (-0.72-1.96) |
| CKD due to hypertension | Brazil | 4524.3 (3752-5281.4) | 6 (4.9-7.1) | 15328.2 (12335.7-18190) | 6.3 (5.1-7.6) | 0.03 (-1.14-1.21) |
| CKD due to hypertension | Brunei Darussalam | 5.7 (4.1-8.1) | 7.5 (5.5-10.5) | 15.1 (11.3-19.2) | 7.5 (5.6-9.7) | 0.39 (-0.97-1.77) |
| CKD due to hypertension | Bulgaria | 115 (88.4-143.4) | 1.3 (1-1.6) | 438 (324-567.3) | 3 (2.3-3.9) | 2.31 (1.12-3.51) |
| CKD due to hypertension | Burkina Faso | 570.4 (445.6-713.6) | 17.3 (13.6-21.9) | 1468.9 (1148.8-1851.3) | 20.3 (16.1-25) | 0.35 (-1.12-1.85) |
| CKD due to hypertension | Burundi | 151.4 (104.4-211.9) | 8.1 (5.7-11.3) | 257 (173.6-404.4) | 7.5 (5-11.7) | -0.71 (-1.9-0.5) |
| CKD due to hypertension | Cabo Verde | 15.6 (12.5-19.1) | 6.6 (5.3-8) | 54.3 (34.9-69.9) | 13 (8.4-16.7) | 1.32 (0.1-2.56) |
| CKD due to hypertension | Cambodia | 459 (361.5-588.1) | 10.3 (8.2-13.2) | 1320 (961.9-1739.1) | 12.2 (9.1-15.8) | 0.02 (-1.47-1.53) |
| CKD due to hypertension | Cameroon | 873 (656.7-1152.6) | 25.1 (18.7-32.8) | 2397 (1704.8-3461.5) | 24.3 (17.3-35) | 0 (-1.39-1.42) |
| CKD due to hypertension | Canada | 1006.6 (834-1161.4) | 3.2 (2.7-3.7) | 3981.6 (3140.7-4613.4) | 4.6 (3.7-5.4) | 0.85 (-0.23-1.94) |
| CKD due to hypertension | Central African Republic | 134 (102.6-174.1) | 15.6 (12.2-20) | 255.1 (179-368.3) | 15.9 (11.4-22.2) | 0.03 (-1.24-1.3) |
| CKD due to hypertension | Chad | 275.3 (199.5-392) | 11.4 (8.4-16.5) | 634.8 (455-954.4) | 13.9 (10.1-20.5) | 0.35 (-0.98-1.69) |
| CKD due to hypertension | Chile | 420.3 (341-502.8) | 4.8 (3.9-5.8) | 1691.3 (1312-2055) | 6.4 (5-7.7) | 0.78 (-0.52-2.1) |
| CKD due to hypertension | China | 28990.2 (23275.1-36186) | 4.4 (3.6-5.5) | 65304.6 (49343.8-82521.1) | 3.5 (2.7-4.4) | -0.72 (-2-0.58) |
| CKD due to hypertension | Colombia | 729.9 (598.3-867.4) | 4.9 (4-5.8) | 2310.9 (1686.8-2883) | 4.1 (3-5.1) | -0.42 (-1.5-0.67) |
| CKD due to hypertension | Comoros | 12.2 (8.6-16.5) | 8.5 (6.1-11.2) | 36.5 (25.1-50.4) | 9.6 (6.8-13.2) | 0.04 (-1.21-1.3) |
| CKD due to hypertension | Congo | 148.5 (104.7-192.7) | 18.9 (13.4-24) | 359.5 (233.8-486.3) | 18.9 (12-24.8) | -0.26 (-1.56-1.06) |
| CKD due to hypertension | Cook Islands | 0.2 (0.2-0.3) | 2.2 (1.5-3.1) | 0.7 (0.5-0.9) | 2.7 (1.9-3.5) | 0.51 (-1.07-2.1) |
| CKD due to hypertension | Costa Rica | 60.3 (47.8-72.7) | 3.7 (2.9-4.5) | 388.3 (296.3-477.5) | 6.9 (5.3-8.5) | 1.71 (0.57-2.86) |
| CKD due to hypertension | C么te d'Ivoire | 471.7 (358-593.6) | 16 (12.5-19.9) | 1433.7 (1083.1-1892.3) | 16.7 (13-21.3) | -0.03 (-1.6-1.56) |
| CKD due to hypertension | Croatia | 85.1 (65.9-107.3) | 1.7 (1.3-2.1) | 262.1 (190.6-341.6) | 2.6 (1.9-3.3) | 0.64 (-0.48-1.79) |
| CKD due to hypertension | Cuba | 249.2 (204.2-292.3) | 2.6 (2.1-3) | 1006.6 (803.4-1222.8) | 4.9 (3.9-5.9) | 1.64 (0.47-2.83) |
| CKD due to hypertension | Cyprus | 33.4 (22.8-48) | 8.1 (5.5-11.4) | 65.1 (45.9-87.5) | 4.3 (3-5.6) | -1.78 (-3.25--0.28) |
| CKD due to hypertension | Czechia | 184.1 (139.9-231.7) | 1.4 (1-1.7) | 305.6 (226.1-392) | 1.3 (1-1.7) | -0.28 (-1.25-0.7) |
| CKD due to hypertension | Democratic People's Republic of Korea | 517.6 (355.8-739) | 3.9 (2.7-5.6) | 1173.7 (839.1-1619.7) | 3.9 (2.8-5.4) | 0.03 (-1.31-1.39) |
| CKD due to hypertension | Democratic Republic of the Congo | 1518.7 (1116.1-2049.4) | 14.2 (10.7-18.5) | 3860.5 (2510.1-5321.5) | 14.5 (9.8-19.8) | -0.04 (-1.29-1.23) |
| CKD due to hypertension | Denmark | 67.9 (51.7-86.6) | 0.8 (0.6-1) | 307.5 (245-372.5) | 2.1 (1.7-2.6) | 1.76 (0.73-2.8) |
| CKD due to hypertension | Djibouti | 5.5 (3.8-8.1) | 6.2 (4.4-8.8) | 36.2 (24.6-51) | 9 (6.4-12) | 0.96 (-0.27-2.19) |
| CKD due to hypertension | Dominica | 6 (4.6-7.3) | 10.4 (8.2-12.5) | 12.2 (9.2-15.7) | 15.7 (11.9-20) | 1.41 (-0.14-2.97) |
| CKD due to hypertension | Dominican Republic | 190.7 (150.1-244.6) | 6 (4.7-7.8) | 815.7 (532.3-1098.6) | 8.3 (5.4-11.2) | 1.45 (0.16-2.76) |
| CKD due to hypertension | Ecuador | 371 (300.2-440.8) | 8 (6.6-9.5) | 2171.8 (1542.6-3023.3) | 14.5 (10.3-19.7) | 1.51 (0.09-2.95) |
| CKD due to hypertension | Egypt | 3317.2 (2408.2-5430.8) | 19.1 (13.7-31.4) | 10867.6 (8132.8-13580.1) | 26.3 (20.3-33.2) | 1.2 (-0.56-3) |
| CKD due to hypertension | El Salvador | 163.6 (126.5-240.5) | 5.7 (4.4-8.4) | 1044 (737.6-1347) | 16 (11.2-20.8) | 3.01 (1.78-4.26) |
| CKD due to hypertension | Equatorial Guinea | 22 (15.6-28.9) | 14.7 (11-18.9) | 70.6 (41.3-107.2) | 18.9 (10.6-27.6) | 0.84 (-0.46-2.16) |
| CKD due to hypertension | Eritrea | 50.6 (33.2-75.4) | 6.4 (4.4-9.3) | 145.8 (92.4-246.5) | 8 (5.2-12.8) | 0.39 (-0.79-1.58) |
| CKD due to hypertension | Estonia | 18.1 (14.3-22.3) | 0.9 (0.8-1.1) | 128.7 (102.8-155.2) | 3.9 (3.1-4.6) | 1.82 (0.87-2.77) |
| CKD due to hypertension | Eswatini | 32.7 (24.1-42.8) | 14.9 (11-19.6) | 88.4 (57.2-126.5) | 19.9 (13.6-27.1) | 1.27 (-0.14-2.7) |
| CKD due to hypertension | Ethiopia | 2774.5 (2102.7-3576.4) | 18 (13.5-22.4) | 4125.1 (3149.5-5257.4) | 11.8 (8.9-14.9) | -1.95 (-3.2--0.69) |
| CKD due to hypertension | Fiji | 10.1 (6.6-15.1) | 3.4 (2.2-5.2) | 34.1 (22.2-47.4) | 5.7 (3.9-7.9) | 0.97 (-0.82-2.78) |
| CKD due to hypertension | Finland | 12.9 (9.6-16.8) | 0.2 (0.1-0.2) | 61.4 (44.2-78.8) | 0.4 (0.3-0.4) | 0.89 (0.12-1.68) |
| CKD due to hypertension | France | 1737.5 (1298.3-2234.2) | 1.9 (1.5-2.4) | 4174.6 (3310.1-4955.8) | 1.9 (1.5-2.2) | 0.15 (-0.84-1.16) |
| CKD due to hypertension | Gabon | 83.6 (62.8-105.4) | 17.7 (13.6-22) | 189.1 (98.2-261.6) | 24.8 (12.4-33.6) | 0.93 (-0.43-2.3) |
| CKD due to hypertension | Gambia | 41 (30.7-53.1) | 15 (11.7-18.9) | 157.5 (115.8-207.6) | 19.4 (14.4-25) | 0.53 (-0.87-1.94) |
| CKD due to hypertension | Georgia | 7.9 (5.5-11.4) | 0.1 (0.1-0.2) | 36.8 (26-49.4) | 0.6 (0.4-0.8) | 2.41 (1.21-3.64) |
| CKD due to hypertension | Germany | 1866.2 (1388.1-2421.4) | 1.4 (1-1.7) | 7847.6 (5371.6-10536.1) | 3 (2.1-3.9) | 2 (0.83-3.19) |
| CKD due to hypertension | Ghana | 445.4 (313.8-669.8) | 9.7 (7-14.6) | 1947.4 (1456.9-2509.9) | 15.7 (11.7-20.2) | 1.59 (0.23-2.97) |
| CKD due to hypertension | Greece | 479.5 (354.9-619.3) | 3.5 (2.6-4.5) | 1237.5 (976.5-1485.6) | 3.5 (2.9-4.2) | -0.45 (-1.79-0.9) |
| CKD due to hypertension | Greenland | 1.2 (1-1.5) | 6 (4.6-7.4) | 3.3 (2.2-4.3) | 7 (4.8-9.2) | 0.58 (-0.65-1.82) |
| CKD due to hypertension | Grenada | 8.6 (7-10.3) | 11.2 (9.2-13.5) | 18.8 (14.6-23.3) | 18.4 (14.4-22.4) | 1.63 (0.08-3.22) |
| CKD due to hypertension | Guam | 4.7 (3.7-6.1) | 8.3 (6.6-10.7) | 15 (12.3-17.5) | 7 (5.7-8.1) | 0.92 (-0.51-2.37) |
| CKD due to hypertension | Guatemala | 210.3 (168.4-255.7) | 8.4 (6.8-10.1) | 1198.2 (906.9-1507.1) | 12 (9.2-15.1) | 1.94 (0.65-3.24) |
| CKD due to hypertension | Guinea | 404.9 (304-546.8) | 14.6 (10.9-19.9) | 750.5 (548.6-1068.2) | 16.1 (11.9-22.5) | 0.15 (-1.34-1.67) |
| CKD due to hypertension | Guinea-Bissau | 68.3 (51.3-85.8) | 21.4 (16.2-26.6) | 112 (84-149.5) | 20.5 (15.9-26.5) | -0.3 (-1.69-1.11) |
| CKD due to hypertension | Guyana | 33.5 (27.4-40.5) | 9.7 (7.9-11.7) | 98.6 (70-132.5) | 17 (12.2-22.1) | 2.57 (1.09-4.07) |
| CKD due to hypertension | Haiti | 242.6 (159.8-451) | 8.8 (5.9-16.8) | 582.2 (309.3-1231.7) | 9.5 (5.1-20.4) | 0.34 (-1.01-1.7) |
| CKD due to hypertension | Honduras | 50.8 (38.1-71.1) | 2.9 (2.1-4) | 322.8 (225.9-428.4) | 6 (4.3-7.9) | 1.9 (0.81-3) |
| CKD due to hypertension | Hungary | 152.4 (119-187.5) | 1.1 (0.9-1.4) | 465.5 (340.3-608.2) | 2.1 (1.6-2.7) | 1.37 (0.32-2.43) |
| CKD due to hypertension | Iceland | 3.2 (2.5-4) | 1 (0.8-1.3) | 11.2 (8.4-13.8) | 1.5 (1.2-1.9) | 0.97 (0.14-1.8) |
| CKD due to hypertension | India | 10871.3 (7995-13778.1) | 2.7 (2.1-3.4) | 33964.4 (25786.8-44097.8) | 3.2 (2.5-4.1) | 0.37 (-0.59-1.34) |
| CKD due to hypertension | Indonesia | 8114.6 (6592.2-10204.5) | 8.2 (6.6-10.6) | 23839.5 (18432-29878.7) | 11.1 (8.7-14.6) | 0.58 (-0.87-2.05) |
| CKD due to hypertension | Iran (Islamic Republic of) | 1460.5 (1051.1-2073.9) | 7.8 (5.6-11.3) | 5998.7 (4695-7076.8) | 9.1 (7.1-10.8) | 0.34 (-1.09-1.79) |
| CKD due to hypertension | Iraq | 861.1 (616.4-1179.6) | 11.7 (8.3-16.2) | 2525.2 (1632.4-3329.2) | 14.6 (9.8-18.9) | 0.09 (-1.4-1.6) |
| CKD due to hypertension | Ireland | 54.5 (41-70.7) | 1.5 (1.1-1.9) | 135.1 (98.6-181.2) | 1.5 (1.1-2.1) | 0.4 (-0.62-1.43) |
| CKD due to hypertension | Israel | 230.2 (176.3-294.1) | 5.5 (4.2-6.9) | 716.5 (507.9-928.8) | 4.9 (3.5-6.3) | 0.26 (-1.06-1.6) |
| CKD due to hypertension | Italy | 2012.1 (1562.8-2507.5) | 2.3 (1.8-2.9) | 5749.1 (4101.2-7409.8) | 2.7 (1.9-3.4) | 0.08 (-0.95-1.11) |
| CKD due to hypertension | Jamaica | 154.1 (126.5-180.8) | 8.3 (6.8-9.8) | 322.1 (227.3-426.1) | 9.8 (6.9-12.9) | 0.28 (-1.14-1.72) |
| CKD due to hypertension | Japan | 2351.7 (1770.7-3034.6) | 1.6 (1.2-2) | 8055.3 (5458.6-10963) | 1.4 (1-1.8) | -0.47 (-1.74-0.82) |
| CKD due to hypertension | Jordan | 90.9 (67.8-125.6) | 9.5 (6.9-13.1) | 512.1 (375.9-675.5) | 10.2 (7.6-13.1) | -0.08 (-1.49-1.35) |
| CKD due to hypertension | Kazakhstan | 26.5 (19.5-34.6) | 0.2 (0.2-0.3) | 94.6 (68.9-128.7) | 0.6 (0.4-0.9) | 1.27 (0.02-2.53) |
| CKD due to hypertension | Kenya | 345.4 (236.3-557.3) | 5.5 (3.8-8.8) | 1310.3 (929.3-1781.9) | 8.1 (5.8-11) | 1.29 (0.09-2.5) |
| CKD due to hypertension | Kiribati | 1 (0.8-1.4) | 3.3 (2.4-4.5) | 2.8 (1.6-4.4) | 4.7 (2.8-7.3) | 0.66 (-1.1-2.45) |
| CKD due to hypertension | Kuwait | 46.4 (37.2-55) | 10.4 (8.2-12.5) | 124.1 (87.1-161.3) | 5.7 (3.9-7.5) | -1.59 (-2.96--0.19) |
| CKD due to hypertension | Kyrgyzstan | 5.3 (3.9-6.8) | 0.2 (0.1-0.2) | 20.2 (14.6-26.1) | 0.5 (0.3-0.6) | 0.63 (-0.66-1.94) |
| CKD due to hypertension | Lao People's Democratic Republic | 381.2 (271.2-516.3) | 20.1 (14.7-27.3) | 788.5 (530.8-1088.9) | 19.2 (13.3-26.3) | -0.44 (-2.18-1.33) |
| CKD due to hypertension | Latvia | 7 (5.5-8.6) | 0.2 (0.2-0.2) | 31.5 (22.5-42.3) | 0.7 (0.5-0.9) | 1.6 (0.78-2.43) |
| CKD due to hypertension | Lebanon | 195.6 (138.1-272.1) | 11.4 (8.1-15.9) | 798.3 (615-1022.3) | 11.9 (9.2-15.2) | 0.05 (-1.5-1.62) |
| CKD due to hypertension | Lesotho | 58.5 (43.3-79.6) | 8.2 (6.1-11.2) | 160.7 (109.6-221.3) | 18.6 (12.5-24.2) | 2.97 (1.49-4.47) |
| CKD due to hypertension | Liberia | 186.6 (142.7-245.2) | 20.3 (15.7-26) | 356 (260.3-480.1) | 21.7 (16.1-28.5) | 0.31 (-1.13-1.77) |
| CKD due to hypertension | Libya | 167.1 (114.6-245.6) | 10.1 (6.8-15.2) | 714.2 (468.5-967.8) | 16.8 (11.2-22.6) | 1.91 (0.31-3.53) |
| CKD due to hypertension | Lithuania | 11.8 (9.4-14.6) | 0.3 (0.2-0.3) | 48.3 (35.5-62.8) | 0.8 (0.6-1) | 0.81 (0.05-1.57) |
| CKD due to hypertension | Luxembourg | 9.3 (7-12.1) | 1.9 (1.4-2.4) | 31 (22.1-41.6) | 2.4 (1.7-3.2) | 0.67 (-0.49-1.85) |
| CKD due to hypertension | Madagascar | 214.9 (150-325.8) | 5.5 (3.8-8.5) | 418.9 (276.7-583.6) | 5.6 (3.9-7.8) | 0.06 (-1.08-1.21) |
| CKD due to hypertension | Malawi | 227.2 (163.9-307.5) | 8.2 (6.1-11.3) | 536.9 (397.3-700.1) | 9.8 (7.6-12.6) | 0.32 (-0.92-1.57) |
| CKD due to hypertension | Malaysia | 903.3 (754.2-1082.1) | 10.1 (8.3-12.1) | 3354.3 (2733.7-4010.1) | 13.3 (10.9-15.8) | 0.37 (-1.36-2.12) |
| CKD due to hypertension | Maldives | 16.9 (13.2-22.8) | 22.9 (17.3-33.9) | 41.8 (33.6-50.8) | 14.2 (11.4-17.5) | -1.64 (-3.39-0.15) |
| CKD due to hypertension | Mali | 492.4 (380.5-648.9) | 16.8 (13-22.1) | 1109.8 (843.7-1505.2) | 16.9 (13.3-22.2) | 0 (-1.39-1.41) |
| CKD due to hypertension | Malta | 8 (6-10.2) | 2.2 (1.6-2.8) | 26.4 (18.6-34.8) | 2.2 (1.6-3) | 0.13 (-1.02-1.3) |
| CKD due to hypertension | Marshall Islands | 0.6 (0.3-1) | 3.9 (2.2-7.4) | 1.8 (0.5-4.9) | 6.5 (1.9-18.8) | 1.32 (-0.48-3.16) |
| CKD due to hypertension | Mauritania | 154.2 (115.9-198.2) | 18.9 (13.9-24.4) | 321.7 (215.2-462) | 18.5 (12.5-26.5) | -0.46 (-1.86-0.96) |
| CKD due to hypertension | Mauritius | 131 (111.9-148) | 20 (17.4-22.5) | 616.6 (521.3-702.4) | 35.2 (30.1-40) | 1.7 (-0.29-3.73) |
| CKD due to hypertension | Mexico | 3051.3 (2454.1-3635.3) | 9.3 (7.6-11.1) | 14842.4 (11615.8-18234) | 12.5 (9.8-15.1) | 1.74 (0.49-3.01) |
| CKD due to hypertension | Micronesia (Federated States of) | 1.7 (1-2.8) | 4 (2.4-6.7) | 3.9 (2.6-5.9) | 6.6 (4.5-10) | 1.29 (-0.56-3.17) |
| CKD due to hypertension | Monaco | 0.9 (0.6-1.3) | 1.1 (0.8-1.5) | 2.3 (1.6-3.2) | 1.8 (1.2-2.4) | 0.98 (-0.04-2.01) |
| CKD due to hypertension | Mongolia | 7.1 (5.1-9.7) | 0.7 (0.5-1) | 12.8 (9.2-17.4) | 0.7 (0.5-0.9) | -0.51 (-1.92-0.91) |
| CKD due to hypertension | Montenegro | 24.7 (18.1-32) | 4.3 (3.1-5.5) | 58.4 (39.8-81.3) | 6.9 (4.7-9.5) | 1.02 (-0.14-2.19) |
| CKD due to hypertension | Morocco | 632.9 (392.3-1265.6) | 5.2 (3.1-10.9) | 2598 (1813.6-3548.4) | 9.2 (6.4-12.5) | 1.55 (-0.11-3.24) |
| CKD due to hypertension | Mozambique | 218.5 (155.7-329.5) | 5.5 (4-8.1) | 571.6 (407.1-835.4) | 7.6 (5.3-11) | 1.25 (0.05-2.46) |
| CKD due to hypertension | Myanmar | 2709.2 (2014.5-3680) | 12 (9-16.3) | 5088.7 (3816.5-6664.8) | 11.7 (9.1-15) | -0.52 (-2.02-1) |
| CKD due to hypertension | Namibia | 42 (30.1-62.9) | 8.8 (6.4-13.4) | 114.5 (81-155.5) | 10.8 (7.9-14.3) | 0.35 (-0.96-1.69) |
| CKD due to hypertension | Nauru | 0.2 (0.1-0.3) | 5 (2.9-9.1) | 0.4 (0.2-0.5) | 7.3 (4.6-13.4) | 0.96 (-0.88-2.83) |
| CKD due to hypertension | Nepal | 219.5 (152.3-304.6) | 2.8 (1.9-4) | 786.8 (554-1083.7) | 4 (2.8-5.6) | 1.02 (0.04-2.02) |
| CKD due to hypertension | Netherlands | 321.9 (245.9-407.8) | 1.6 (1.2-2) | 1271.2 (997.5-1499.4) | 3.1 (2.4-3.6) | 0.98 (-0.14-2.11) |
| CKD due to hypertension | New Zealand | 66.5 (52.9-80.2) | 1.8 (1.4-2.1) | 271 (210-330.8) | 2.8 (2.2-3.5) | 0.92 (-0.14-2) |
| CKD due to hypertension | Nicaragua | 93.5 (72.3-128.9) | 6.8 (5.2-9.3) | 576.9 (434.2-744.7) | 12.7 (9.7-16.3) | 2.35 (1.14-3.58) |
| CKD due to hypertension | Niger | 278.8 (209.1-393) | 13.5 (10.3-18.4) | 775.1 (546.7-1216.2) | 13 (9.5-20) | -0.31 (-1.72-1.13) |
| CKD due to hypertension | Nigeria | 4423.9 (3407.1-5665) | 12.8 (10-16.1) | 9284.7 (6996.2-12134.8) | 13.8 (10.7-17.3) | -0.18 (-1.61-1.27) |
| CKD due to hypertension | Niue | 0.1 (0.1-0.1) | 3.6 (2.5-5) | 0.1 (0.1-0.2) | 6.4 (3.5-10.4) | 1.49 (-0.31-3.33) |
| CKD due to hypertension | North Macedonia | 34.4 (24.8-45.3) | 2.1 (1.5-2.8) | 80.8 (55.8-110) | 3.1 (2.3-4.1) | 0.55 (-0.69-1.82) |
| CKD due to hypertension | Northern Mariana Islands | 0.3 (0.2-0.4) | 2.1 (1.5-3.1) | 1.2 (0.9-1.7) | 3.2 (2.2-4.4) | 1.07 (-0.89-3.06) |
| CKD due to hypertension | Norway | 64 (48.8-80.4) | 0.8 (0.6-1) | 228.4 (169.6-291.4) | 1.8 (1.3-2.3) | 1.07 (0.22-1.92) |
| CKD due to hypertension | Oman | 41.9 (29-64.9) | 7.8 (5.3-12.2) | 192.9 (141.7-249.7) | 14.7 (10.8-18.8) | 1.99 (0.44-3.57) |
| CKD due to hypertension | Pakistan | 1952.7 (1444-2661.9) | 3.9 (2.9-5.3) | 5790.9 (4291.9-7813.2) | 5.6 (4.2-7.4) | 0.75 (-0.32-1.83) |
| CKD due to hypertension | Palau | 0.3 (0.2-0.4) | 3.6 (2.4-5) | 1 (0.6-1.4) | 6 (4-8.3) | 1.48 (-0.3-3.3) |
| CKD due to hypertension | Palestine | 114 (78.7-159) | 16.5 (11.6-23.5) | 275.6 (212.5-347.9) | 15.8 (12.1-19.9) | -0.17 (-1.85-1.54) |
| CKD due to hypertension | Panama | 45.4 (36.6-54.5) | 3.3 (2.6-3.9) | 345.9 (246.5-435.3) | 7.6 (5.4-9.6) | 2.33 (1.17-3.5) |
| CKD due to hypertension | Papua New Guinea | 21.3 (13.2-32.8) | 1.3 (0.8-2) | 72.7 (49.9-106.8) | 1.6 (1.1-2.5) | 0.36 (-0.92-1.65) |
| CKD due to hypertension | Paraguay | 113.9 (88.1-142) | 5.6 (4.3-7) | 472.7 (334.7-646.4) | 8.7 (6.2-11.8) | 1.14 (-0.09-2.38) |
| CKD due to hypertension | Peru | 1002.5 (794.3-1260.7) | 9.2 (7.3-11.5) | 3543.9 (2394.5-4623.3) | 10.6 (7.1-13.8) | 0.07 (-1.23-1.38) |
| CKD due to hypertension | Philippines | 3781.3 (3232.5-4525.7) | 15.3 (13.2-17.9) | 15064.3 (12064.4-18047.6) | 19.7 (16-23.3) | 1.03 (-0.68-2.78) |
| CKD due to hypertension | Poland | 854.3 (677.5-1046.1) | 2.1 (1.7-2.5) | 918.5 (678.6-1174.2) | 1.2 (0.9-1.5) | -1.57 (-2.61--0.52) |
| CKD due to hypertension | Portugal | 267.7 (201.6-338.2) | 2.2 (1.7-2.8) | 907.9 (637.1-1211.3) | 2.7 (1.9-3.5) | 0.23 (-0.99-1.47) |
| CKD due to hypertension | Puerto Rico | 276.7 (219.9-335.4) | 8.4 (6.6-10.1) | 655.4 (522.8-806.7) | 7.4 (6.1-8.9) | 0.53 (-0.81-1.88) |
| CKD due to hypertension | Qatar | 8.3 (4.5-13.6) | 15 (7.9-25.4) | 60.8 (44.4-82.1) | 14.7 (11.1-19.1) | -0.47 (-2.17-1.25) |
| CKD due to hypertension | Republic of Korea | 1110.5 (966.5-1271.7) | 5.7 (4.9-6.7) | 3136.7 (2293.8-3966.8) | 3.5 (2.6-4.4) | -1.32 (-2.43--0.19) |
| CKD due to hypertension | Republic of Moldova | 8.9 (7-11.1) | 0.2 (0.2-0.3) | 24.9 (18.7-31.5) | 0.4 (0.3-0.5) | 0.5 (-0.13-1.14) |
| CKD due to hypertension | Romania | 207.9 (171.5-243.4) | 0.9 (0.8-1) | 296.6 (231-366) | 0.7 (0.6-0.9) | -0.16 (-1.3-1) |
| CKD due to hypertension | Russian Federation | 877 (701.6-1090) | 0.5 (0.4-0.6) | 1813.9 (1376.5-2290.4) | 0.8 (0.6-1) | 0 (-0.79-0.8) |
| CKD due to hypertension | Rwanda | 219.6 (165.5-287.2) | 10.5 (8-13.7) | 404.7 (268.7-569.5) | 9.2 (6.1-12.7) | -1.4 (-2.65--0.14) |
| CKD due to hypertension | Saint Kitts and Nevis | 4.9 (3.9-5.9) | 14 (11.5-16.3) | 10.5 (7.9-13.2) | 18.7 (14.6-22.8) | 1.3 (-0.27-2.89) |
| CKD due to hypertension | Saint Lucia | 9.6 (7.9-11.2) | 12.6 (10.4-14.7) | 30.2 (23.3-37.6) | 13.2 (10.2-16.4) | 0.46 (-1.02-1.96) |
| CKD due to hypertension | Saint Vincent and the Grenadines | 5.8 (4.8-6.8) | 8.7 (7.2-10.4) | 17.2 (13.6-20.6) | 13.4 (10.6-16) | 1.57 (0.14-3.03) |
| CKD due to hypertension | Samoa | 2.9 (1.8-4.2) | 4 (2.6-5.8) | 6.9 (4.8-9.5) | 5.5 (3.8-7.4) | 0.76 (-0.99-2.54) |
| CKD due to hypertension | San Marino | 0.4 (0.3-0.5) | 1 (0.7-1.4) | 0.9 (0.5-1.3) | 0.7 (0.4-1.1) | 0.12 (-0.76-1) |
| CKD due to hypertension | Sao Tome and Principe | 12.9 (10.2-15.3) | 23.4 (18.5-27.8) | 27.9 (19.1-34.8) | 32.3 (21.8-40.2) | 0.88 (-0.59-2.37) |
| CKD due to hypertension | Saudi Arabia | 694.6 (477.5-1043.8) | 14.8 (10.4-22.5) | 3410.7 (2400.6-4436.8) | 26.4 (18.8-33.1) | 1.45 (-0.22-3.14) |
| CKD due to hypertension | Senegal | 452.6 (360.6-588.3) | 17.3 (13.9-21.8) | 1184.4 (878.8-1644.5) | 19.1 (14.5-26) | 0.09 (-1.28-1.48) |
| CKD due to hypertension | Serbia | 427.7 (325.9-576.5) | 5.1 (3.8-6.9) | 914.8 (724.3-1125.8) | 5.3 (4.2-6.4) | -0.06 (-1.24-1.13) |
| CKD due to hypertension | Seychelles | 7.5 (6.4-9) | 13.3 (11.2-15.9) | 22.4 (18.1-27.3) | 21.7 (17.5-26.2) | 1.22 (-0.66-3.14) |
| CKD due to hypertension | Sierra Leone | 229.3 (170.7-298.6) | 13.3 (10.1-17.2) | 396.5 (291-537.2) | 13 (9.9-17.1) | -0.09 (-1.43-1.27) |
| CKD due to hypertension | Singapore | 31.6 (24.3-39.4) | 1.8 (1.4-2.3) | 124.7 (90.5-164.3) | 1.5 (1.1-2) | -0.35 (-1.44-0.75) |
| CKD due to hypertension | Slovakia | 100.6 (76.1-129.4) | 1.7 (1.3-2.2) | 148.9 (109.8-195) | 1.6 (1.2-2) | -0.31 (-1.37-0.76) |
| CKD due to hypertension | Slovenia | 12.1 (9.8-16.2) | 0.5 (0.4-0.7) | 49.3 (33.7-68.6) | 0.9 (0.6-1.2) | 0.32 (-0.65-1.29) |
| CKD due to hypertension | Solomon Islands | 3.1 (1.2-5.2) | 2.5 (1.3-4.1) | 9.1 (6.4-12.4) | 2.9 (2.1-3.8) | 0.27 (-1.31-1.88) |
| CKD due to hypertension | Somalia | 156.2 (103.3-227.2) | 9.3 (6.2-13.8) | 412.7 (270.8-656.8) | 9.7 (6.5-14.6) | 0.26 (-0.96-1.49) |
| CKD due to hypertension | South Africa | 1223.4 (1008.6-1565) | 6.6 (5.4-8.7) | 5550.4 (4695.9-6582.2) | 14.4 (12.2-17) | 1.4 (0.01-2.81) |
| CKD due to hypertension | South Sudan | 174 (116.4-262.4) | 8.5 (6-12.4) | 314 (214.8-439.7) | 11.3 (7.7-15.4) | 0.79 (-0.47-2.06) |
| CKD due to hypertension | Spain | 1204.7 (915.6-1571) | 2.3 (1.8-3) | 2908.1 (2034.6-3974) | 2 (1.4-2.6) | -0.35 (-1.51-0.83) |
| CKD due to hypertension | Sri Lanka | 1296.9 (1039.3-1599) | 14.5 (11.5-17.7) | 2513.7 (1626.8-3508.9) | 10.4 (6.9-14.3) | -0.61 (-2.26-1.06) |
| CKD due to hypertension | Sudan | 804.1 (530.5-1521.7) | 10.4 (6.8-20.9) | 2356.7 (1696-3309) | 15 (11-21.2) | 0.88 (-0.62-2.39) |
| CKD due to hypertension | Suriname | 20.2 (16.1-25.3) | 8.3 (6.5-10.5) | 73.5 (51-100.6) | 12.2 (8.5-16.6) | 1.34 (-0.12-2.83) |
| CKD due to hypertension | Sweden | 108.5 (81.2-141.3) | 0.6 (0.5-0.8) | 455.3 (318.9-606.8) | 1.5 (1.1-2) | 1.71 (0.82-2.6) |
| CKD due to hypertension | Switzerland | 244.5 (192.1-306) | 2.1 (1.7-2.6) | 710.8 (520-876.7) | 2.7 (2-3.3) | 1.13 (0.05-2.23) |
| CKD due to hypertension | Syrian Arab Republic | 574.4 (418.9-830.6) | 13.5 (9.7-19.8) | 1608.4 (1108.8-2196.7) | 17.3 (11.9-23.4) | 0.18 (-1.45-1.84) |
| CKD due to hypertension | Taiwan (Province of China) | 808.6 (646.3-979.4) | 7.1 (5.6-8.7) | 2738.5 (2096.5-3377.8) | 6.1 (4.6-7.6) | -0.18 (-1.77-1.43) |
| CKD due to hypertension | Tajikistan | 2 (1.4-3) | 0.1 (0.1-0.1) | 5.5 (3.6-8.1) | 0.1 (0.1-0.2) | 0.12 (-0.59-0.83) |
| CKD due to hypertension | Thailand | 3592.6 (2858.3-4678.8) | 11.6 (9.1-15.3) | 15302.8 (11191.5-19795.2) | 14.3 (10.5-18.5) | -0.03 (-1.7-1.66) |
| CKD due to hypertension | Timor-Leste | 31.6 (22.2-44.8) | 12.5 (8.9-17.3) | 114.9 (77.7-172.6) | 15.1 (10.6-22) | 0.33 (-1.29-1.98) |
| CKD due to hypertension | Togo | 130 (101.6-165.7) | 13.5 (10.8-17.2) | 426.2 (314.8-580.8) | 15.3 (11.6-20.4) | 0.18 (-1.16-1.55) |
| CKD due to hypertension | Tokelau | 0 (0-0.1) | 3 (1.9-5.5) | 0.1 (0-0.1) | 4.3 (2.8-6.6) | 0.96 (-0.74-2.68) |
| CKD due to hypertension | Tonga | 0.7 (0.5-1.1) | 1.5 (1-2.2) | 1.8 (1.2-2.6) | 2.4 (1.5-3.3) | 0.74 (-0.8-2.31) |
| CKD due to hypertension | Trinidad and Tobago | 60.4 (49.4-71.3) | 8.4 (6.9-9.8) | 238.8 (170.2-315) | 12.8 (9.2-16.7) | 1.78 (0.33-3.25) |
| CKD due to hypertension | Tunisia | 197.5 (129.7-313) | 5.3 (3.6-8.4) | 882.9 (583.9-1289.4) | 7.8 (5.1-11.6) | 0.91 (-0.57-2.42) |
| CKD due to hypertension | Turkey | 2998.7 (2226.8-4414) | 10.8 (8-15.9) | 8736.8 (6173.5-11304.1) | 10.6 (7.5-13.8) | 0.17 (-1.29-1.66) |
| CKD due to hypertension | Turkmenistan | 6.9 (5.3-8.9) | 0.4 (0.3-0.5) | 29.6 (21.1-41.3) | 0.8 (0.6-1.1) | 1.2 (-0.22-2.65) |
| CKD due to hypertension | Tuvalu | 0.2 (0.1-0.3) | 3.3 (2.2-5.5) | 0.4 (0.3-0.6) | 4.8 (3.2-7.2) | 0.92 (-0.81-2.67) |
| CKD due to hypertension | Uganda | 313.1 (210.8-445.6) | 6.5 (4.4-9.1) | 882 (624.9-1192.5) | 8.1 (5.8-10.8) | 0.36 (-0.84-1.58) |
| CKD due to hypertension | Ukraine | 9.4 (7.6-11.5) | 0 (0-0) | 182.4 (125.7-253.2) | 0.3 (0.2-0.3) | 2.08 (1.66-2.5) |
| CKD due to hypertension | United Arab Emirates | 22.7 (15.2-33.4) | 7.6 (5.1-11.3) | 185.8 (118.1-263.2) | 12.6 (7-17.3) | 2.89 (1.24-4.57) |
| CKD due to hypertension | United Kingdom | 561.2 (424.6-722.6) | 0.6 (0.5-0.8) | 1328.4 (955.1-1763.8) | 0.8 (0.6-1.1) | 0.9 (0.05-1.75) |
| CKD due to hypertension | United Republic of Tanzania | 389.7 (282.8-538.4) | 4.9 (3.6-6.7) | 1021.1 (737.2-1362.5) | 5.2 (3.8-7) | 0.05 (-1.44-1.56) |
| CKD due to hypertension | United States of America | 11428 (9351-13522) | 3.4 (2.8-4) | 55559.6 (45510.1-62185.1) | 8.7 (7.2-9.7) | 2.19 (0.97-3.43) |
| CKD due to hypertension | United States Virgin Islands | 5.1 (3.9-6.7) | 7 (5.5-8.9) | 11.7 (8-15.5) | 7.3 (5.1-9.7) | 0.38 (-1.03-1.82) |
| CKD due to hypertension | Uruguay | 182.6 (166.4-196) | 4.7 (4.3-5.1) | 394.8 (306.1-481.8) | 5.8 (4.6-7) | 0.37 (-0.86-1.62) |
| CKD due to hypertension | Uzbekistan | 25.1 (15.1-44.8) | 0.2 (0.1-0.4) | 151.4 (109.4-201.5) | 0.7 (0.5-0.9) | 1.35 (0.01-2.72) |
| CKD due to hypertension | Vanuatu | 1.3 (0.7-2.4) | 2.6 (1.6-4.6) | 5.7 (3.7-9.3) | 3.9 (2.6-6.3) | 1.05 (-0.62-2.75) |
| CKD due to hypertension | Venezuela (Bolivarian Republic of) | 339.5 (273.5-404.3) | 3.9 (3.2-4.7) | 2792.3 (1897.3-3750) | 9.9 (6.7-13.2) | 2.15 (0.97-3.35) |
| CKD due to hypertension | Viet Nam | 4406.4 (3136.3-5672.2) | 11.8 (8.4-15.1) | 11649.7 (7776.8-14754.4) | 13.7 (9.3-17.1) | 0.34 (-1.31-2.02) |
| CKD due to hypertension | Yemen | 248.3 (146.7-482.7) | 6.5 (3.9-13.7) | 784.5 (507-1311) | 7.4 (4.8-12.7) | 0.09 (-1.24-1.44) |
| CKD due to hypertension | Zambia | 201.5 (152.6-260.1) | 9.5 (7.3-12.1) | 557.1 (375.7-819.2) | 10.8 (7.5-14.8) | 0.09 (-1.15-1.34) |
| CKD due to hypertension | Zimbabwe | 316.5 (237.3-426.7) | 10.8 (8.1-14.4) | 830.8 (604.1-1157.5) | 16.4 (12.1-22) | 1.63 (0.19-3.08) |
| CKD due to other and unspecified causes | Afghanistan | 1153.7 (823.3-1730) | 17.4 (12.2-27.5) | 2005.4 (1197.6-3498.8) | 19.8 (11.5-37.2) | 0.36 (-1.22-1.96) |
| CKD due to other and unspecified causes | Albania | 139.9 (116.3-171.2) | 7 (5.8-8.6) | 244 (179.9-315.5) | 6.2 (4.6-8) | -0.11 (-1.3-1.09) |
| CKD due to other and unspecified causes | Algeria | 876 (593-1547.9) | 9.2 (6-16.7) | 3356.8 (2596.6-4325.5) | 12.6 (9.5-16.5) | 1.13 (-0.42-2.7) |
| CKD due to other and unspecified causes | American Samoa | 0.2 (0.2-0.4) | 0.7 (0.4-1.1) | 0.6 (0.4-0.8) | 1.4 (1-2) | 2.12 (0.13-4.16) |
| CKD due to other and unspecified causes | Andorra | 2.5 (1.7-3.5) | 5.8 (4-8.1) | 8.1 (5.6-11.1) | 4.4 (3.1-6.1) | -0.33 (-1.44-0.79) |
| CKD due to other and unspecified causes | Angola | 331.4 (242.9-427) | 7.3 (5.3-9.6) | 858.2 (611.7-1194.4) | 7.5 (5.2-10.6) | -0.01 (-1.29-1.29) |
| CKD due to other and unspecified causes | Antigua and Barbuda | 0.4 (0.3-0.5) | 0.7 (0.6-1) | 1.3 (1-1.7) | 1.3 (1-1.7) | 1.66 (0.11-3.24) |
| CKD due to other and unspecified causes | Argentina | 3306.2 (2659.8-3899) | 11 (8.9-12.8) | 5337.6 (4296.2-6410.4) | 9.3 (7.5-11.1) | -0.28 (-1.65-1.11) |
| CKD due to other and unspecified causes | Armenia | 17.4 (14.5-20.3) | 0.6 (0.5-0.7) | 278.1 (222.7-345) | 6.6 (5.3-8.2) | 3.05 (1.96-4.16) |
| CKD due to other and unspecified causes | Australia | 734.1 (657.8-804.6) | 4 (3.6-4.5) | 2029.4 (1577.9-2470) | 3.8 (3-4.6) | 0.64 (-0.47-1.77) |
| CKD due to other and unspecified causes | Austria | 333.9 (268.4-392.6) | 2.8 (2.3-3.3) | 1431 (1156.7-1664.4) | 5.9 (4.9-6.8) | 2.48 (1.37-3.61) |
| CKD due to other and unspecified causes | Azerbaijan | 235 (181.5-301.6) | 4.3 (3.3-5.5) | 632 (463.4-831.3) | 6.7 (4.9-8.6) | 0.95 (-0.28-2.2) |
| CKD due to other and unspecified causes | Bahamas | 1.2 (1-1.5) | 0.7 (0.6-0.9) | 4.4 (3.1-6.2) | 1.1 (0.8-1.5) | 1.65 (0.24-3.08) |
| CKD due to other and unspecified causes | Bahrain | 17.5 (12.4-24.4) | 14.1 (9.9-19.6) | 90.9 (69.8-118.3) | 17.7 (12.9-23.2) | 0.24 (-1.54-2.04) |
| CKD due to other and unspecified causes | Bangladesh | 4429.7 (2233.9-5757.6) | 4.9 (3.7-5.9) | 4369.9 (3315.8-5741.2) | 3.5 (2.7-4.6) | -0.46 (-1.43-0.52) |
| CKD due to other and unspecified causes | Barbados | 1.6 (1.2-2) | 0.6 (0.4-0.7) | 4.1 (2.8-5.8) | 0.8 (0.6-1.2) | 1.28 (-0.15-2.73) |
| CKD due to other and unspecified causes | Belarus | 33.8 (28.3-39.4) | 0.3 (0.2-0.3) | 114.4 (86.7-145.6) | 0.8 (0.6-0.9) | 0.89 (0.42-1.36) |
| CKD due to other and unspecified causes | Belgium | 624.3 (504.1-726.5) | 4 (3.3-4.6) | 1282 (972.5-1575.4) | 4.1 (3.2-4.9) | 0.39 (-0.7-1.49) |
| CKD due to other and unspecified causes | Belize | 1 (0.9-1.2) | 0.8 (0.6-1) | 4.4 (3.3-5.7) | 1.4 (1-1.8) | 2.06 (0.57-3.56) |
| CKD due to other and unspecified causes | Benin | 188 (149.9-228.3) | 7.1 (5.6-8.9) | 421.1 (315.6-540.9) | 7.3 (5.4-9.7) | 0.07 (-1.35-1.51) |
| CKD due to other and unspecified causes | Bermuda | 0.2 (0.2-0.3) | 0.4 (0.3-0.5) | 0.5 (0.4-0.7) | 0.4 (0.3-0.5) | 0.27 (-1-1.56) |
| CKD due to other and unspecified causes | Bhutan | 13.2 (8.7-18.5) | 4.6 (3.2-6.6) | 32.8 (20.7-45.9) | 5.6 (3.6-7.9) | 0.51 (-0.52-1.54) |
| CKD due to other and unspecified causes | Bolivia (Plurinational State of) | 131.9 (101.1-172.8) | 3.4 (2.5-4.5) | 355.1 (250.4-515.2) | 4.2 (2.9-6) | 0.81 (-0.57-2.21) |
| CKD due to other and unspecified causes | Bosnia and Herzegovina | 177.6 (141.9-210.8) | 4.9 (3.9-5.8) | 318.2 (237.1-408.7) | 5.2 (3.9-6.6) | 0.25 (-0.8-1.32) |
| CKD due to other and unspecified causes | Botswana | 27.2 (18.7-39.8) | 5.4 (3.7-7.8) | 84.8 (62-118.5) | 6.8 (5-9.8) | 0.61 (-0.72-1.96) |
| CKD due to other and unspecified causes | Brazil | 1320.1 (1092.1-1561.1) | 1.4 (1.1-1.7) | 3224.4 (2515.2-4015.8) | 1.3 (1-1.7) | 0.03 (-1.14-1.21) |
| CKD due to other and unspecified causes | Brunei Darussalam | 6.9 (5.3-8.9) | 7.2 (5.3-9.5) | 16.4 (12.3-21.1) | 7 (5.2-9.2) | 0.39 (-0.97-1.77) |
| CKD due to other and unspecified causes | Bulgaria | 355.6 (302.5-410.3) | 3.8 (3.3-4.4) | 1180.1 (928.7-1459.2) | 8.5 (6.7-10.6) | 2.31 (1.12-3.51) |
| CKD due to other and unspecified causes | Burkina Faso | 376.3 (294.6-473.2) | 7.5 (5.7-9.6) | 875.6 (663.3-1141.2) | 8.5 (6.3-11.4) | 0.35 (-1.12-1.85) |
| CKD due to other and unspecified causes | Burundi | 154.1 (113.4-205.9) | 6 (4.4-8.2) | 240.5 (169.3-360.5) | 5.2 (3.5-8) | -0.71 (-1.9-0.5) |
| CKD due to other and unspecified causes | Cabo Verde | 7.3 (5.6-9.3) | 2.8 (2.1-3.5) | 21.4 (14.1-30.1) | 4.9 (3.2-7.1) | 1.32 (0.1-2.56) |
| CKD due to other and unspecified causes | Cambodia | 53.8 (35.2-71.3) | 0.5 (0.4-0.7) | 57.8 (39.8-81) | 0.4 (0.3-0.6) | 0.02 (-1.47-1.53) |
| CKD due to other and unspecified causes | Cameroon | 470.3 (334.1-618.9) | 8.8 (6.2-12) | 1161.3 (772.1-1644.4) | 8.2 (5.3-11.9) | 0 (-1.39-1.42) |
| CKD due to other and unspecified causes | Canada | 232.9 (178.4-298.8) | 0.8 (0.6-1) | 853.7 (614.3-1161.3) | 1.1 (0.8-1.4) | 0.85 (-0.23-1.94) |
| CKD due to other and unspecified causes | Central African Republic | 108.8 (84.2-136.2) | 8.8 (6.7-11.5) | 198.3 (136.1-280.2) | 8.9 (6.2-12.9) | 0.03 (-1.24-1.3) |
| CKD due to other and unspecified causes | Chad | 169.6 (130.5-225.1) | 4.9 (3.6-6.7) | 404.8 (298.9-559) | 5.6 (3.9-8.2) | 0.35 (-0.98-1.69) |
| CKD due to other and unspecified causes | Chile | 557.4 (465.3-642.3) | 6 (5-6.9) | 1866.7 (1486.7-2243.5) | 7.2 (5.7-8.5) | 0.78 (-0.52-2.1) |
| CKD due to other and unspecified causes | China | 5801.4 (4667.7-6927.5) | 0.6 (0.5-0.7) | 5265.7 (3853.7-7042.4) | 0.3 (0.2-0.4) | -0.72 (-2-0.58) |
| CKD due to other and unspecified causes | Colombia | 877.4 (733.3-1027.3) | 4.9 (4-5.9) | 2134.2 (1636.4-2729) | 3.8 (2.9-4.9) | -0.42 (-1.5-0.67) |
| CKD due to other and unspecified causes | Comoros | 12.2 (8.9-16.5) | 6.2 (4.4-8.3) | 29.4 (19.7-40.8) | 6.8 (4.4-9.6) | 0.04 (-1.21-1.3) |
| CKD due to other and unspecified causes | Congo | 107.8 (81.2-141) | 10.5 (7.3-13.7) | 242.6 (158.3-330.7) | 10.4 (6.4-14.2) | -0.26 (-1.56-1.06) |
| CKD due to other and unspecified causes | Cook Islands | 0.1 (0.1-0.1) | 0.7 (0.5-0.9) | 0.2 (0.1-0.2) | 0.8 (0.6-1.1) | 0.51 (-1.07-2.1) |
| CKD due to other and unspecified causes | Costa Rica | 63.3 (51.5-76) | 3.6 (2.8-4.4) | 367 (293.4-456.5) | 6.6 (5.3-8.3) | 1.71 (0.57-2.86) |
| CKD due to other and unspecified causes | C么te d'Ivoire | 639.5 (508.4-804.9) | 14.3 (11-17.7) | 1649.5 (1206.1-2156.4) | 14.9 (11.2-19.2) | -0.03 (-1.6-1.56) |
| CKD due to other and unspecified causes | Croatia | 228.7 (189.5-268) | 4.4 (3.7-5.1) | 619.2 (483.6-768.7) | 6.3 (4.9-7.8) | 0.64 (-0.48-1.79) |
| CKD due to other and unspecified causes | Cuba | 23.5 (18.8-29) | 0.2 (0.2-0.3) | 86.9 (63.9-116.5) | 0.5 (0.3-0.6) | 1.64 (0.47-2.83) |
| CKD due to other and unspecified causes | Cyprus | 88.5 (63.3-114.3) | 20 (15-25.8) | 161.8 (125.2-201.5) | 10.5 (8.3-13.2) | -1.78 (-3.25--0.28) |
| CKD due to other and unspecified causes | Czechia | 532.8 (448.9-617.3) | 4 (3.4-4.6) | 767.5 (595.9-930.7) | 3.4 (2.6-4.1) | -0.28 (-1.25-0.7) |
| CKD due to other and unspecified causes | Democratic People's Republic of Korea | 86 (58.7-123.8) | 0.5 (0.3-0.7) | 111.7 (80-157.4) | 0.4 (0.3-0.6) | 0.03 (-1.31-1.39) |
| CKD due to other and unspecified causes | Democratic Republic of the Congo | 1293.9 (962.6-1613.5) | 7.9 (5.8-10.1) | 2738.7 (1890.5-3849.4) | 8 (5.3-11.6) | -0.04 (-1.29-1.23) |
| CKD due to other and unspecified causes | Denmark | 202.3 (167.3-229.6) | 2.4 (2-2.7) | 838 (701.4-951.3) | 5.9 (5-6.7) | 1.76 (0.73-2.8) |
| CKD due to other and unspecified causes | Djibouti | 6 (4.3-8.2) | 4.4 (3.1-6.3) | 31.1 (21.5-44.1) | 6.1 (4.2-8.5) | 0.96 (-0.27-2.19) |
| CKD due to other and unspecified causes | Dominica | 0.6 (0.4-0.8) | 0.9 (0.7-1.3) | 1.1 (0.8-1.5) | 1.4 (1.1-2) | 1.41 (-0.14-2.97) |
| CKD due to other and unspecified causes | Dominican Republic | 31.7 (25.4-39.5) | 0.6 (0.5-0.8) | 78 (54.5-106.7) | 0.8 (0.5-1.1) | 1.45 (0.16-2.76) |
| CKD due to other and unspecified causes | Ecuador | 107.2 (86.9-128.6) | 1.8 (1.4-2.2) | 457.2 (306-676.6) | 2.9 (2-4.2) | 1.51 (0.09-2.95) |
| CKD due to other and unspecified causes | Egypt | 4209.4 (3249.9-6223.2) | 18.7 (13.4-29.1) | 12205.3 (9471.8-15567.5) | 25.1 (19.6-32) | 1.2 (-0.56-3) |
| CKD due to other and unspecified causes | El Salvador | 212.1 (168.6-295) | 6.4 (5-9.2) | 1072.4 (786.9-1449) | 16.8 (12.3-23) | 3.01 (1.78-4.26) |
| CKD due to other and unspecified causes | Equatorial Guinea | 16.5 (12.5-21.2) | 8.2 (5.9-10.9) | 52.4 (31.3-79) | 10.3 (5.6-15.3) | 0.84 (-0.46-2.16) |
| CKD due to other and unspecified causes | Eritrea | 61.3 (42.9-89.1) | 4.9 (3.5-7.1) | 142.3 (93.3-234) | 5.8 (3.7-9) | 0.39 (-0.79-1.58) |
| CKD due to other and unspecified causes | Estonia | 43.4 (36.3-51.2) | 2.4 (2-2.8) | 189.8 (153.4-229.7) | 6.2 (5-7.4) | 1.82 (0.87-2.77) |
| CKD due to other and unspecified causes | Eswatini | 25.1 (18.4-32.7) | 8.9 (6.3-11.7) | 65.7 (41-95.2) | 12.4 (7.9-17.3) | 1.27 (-0.14-2.7) |
| CKD due to other and unspecified causes | Ethiopia | 1519 (1120.1-1874.8) | 6.1 (4.7-7.8) | 1752.9 (1358-2183.2) | 3.7 (2.7-4.7) | -1.95 (-3.2--0.69) |
| CKD due to other and unspecified causes | Fiji | 6.6 (4.3-9.8) | 1.2 (0.8-1.8) | 14.3 (10.2-19.4) | 2.1 (1.5-2.8) | 0.97 (-0.82-2.78) |
| CKD due to other and unspecified causes | Finland | 103.4 (83.5-119.4) | 1.5 (1.2-1.7) | 418.6 (328.5-489.1) | 2.5 (2-2.9) | 0.89 (0.12-1.68) |
| CKD due to other and unspecified causes | France | 2974.2 (2351-3573.7) | 3.4 (2.7-4) | 6574.6 (5375.5-7598.2) | 3.2 (2.7-3.6) | 0.15 (-0.84-1.16) |
| CKD due to other and unspecified causes | Gabon | 51 (37.3-65) | 9.5 (6.9-12.3) | 116.9 (59.8-163.3) | 13.4 (6.5-18.6) | 0.93 (-0.43-2.3) |
| CKD due to other and unspecified causes | Gambia | 25.9 (19.7-33.1) | 6.1 (4.6-8) | 78.8 (56.3-106.7) | 7.7 (5.3-10.8) | 0.53 (-0.87-1.94) |
| CKD due to other and unspecified causes | Georgia | 125.8 (100.3-159.1) | 2.1 (1.7-2.6) | 454 (359.5-578.7) | 7.8 (6.2-10.1) | 2.41 (1.21-3.64) |
| CKD due to other and unspecified causes | Germany | 4981.4 (4032-5910.5) | 3.8 (3.1-4.4) | 18431 (13630.6-22927) | 7.1 (5.4-8.8) | 2 (0.83-3.19) |
| CKD due to other and unspecified causes | Ghana | 253 (186.7-360.5) | 3.7 (2.6-5.4) | 909.3 (679.2-1205.1) | 5.9 (4.1-8.1) | 1.59 (0.23-2.97) |
| CKD due to other and unspecified causes | Greece | 1423.7 (1145.3-1651.1) | 10.3 (8.5-11.9) | 3498.8 (2974.3-3919.7) | 10.5 (9.2-11.7) | -0.45 (-1.79-0.9) |
| CKD due to other and unspecified causes | Greenland | 0.4 (0.3-0.5) | 1.5 (1.1-1.9) | 0.8 (0.5-1.1) | 1.6 (1.1-2.4) | 0.58 (-0.65-1.82) |
| CKD due to other and unspecified causes | Grenada | 0.8 (0.7-1.1) | 1.1 (0.9-1.4) | 1.7 (1.3-2.3) | 1.6 (1.2-2.1) | 1.63 (0.08-3.22) |
| CKD due to other and unspecified causes | Guam | 1 (0.8-1.3) | 1.4 (1.1-1.9) | 1.9 (1.6-2.5) | 1 (0.8-1.2) | 0.92 (-0.51-2.37) |
| CKD due to other and unspecified causes | Guatemala | 316.1 (273-367.6) | 8.5 (7-10.1) | 1358.8 (1069.6-1716.2) | 12.5 (9.9-15.7) | 1.94 (0.65-3.24) |
| CKD due to other and unspecified causes | Guinea | 203.5 (157-261.7) | 5.1 (3.8-6.8) | 315.9 (233-429.2) | 5.1 (3.7-7.2) | 0.15 (-1.34-1.67) |
| CKD due to other and unspecified causes | Guinea-Bissau | 46.1 (34.4-58.9) | 9.2 (7-12.1) | 64.1 (47.7-84.4) | 8.3 (6.1-11.5) | -0.3 (-1.69-1.11) |
| CKD due to other and unspecified causes | Guyana | 4.4 (3.6-5.5) | 0.9 (0.7-1.2) | 11.9 (8.1-16.7) | 1.8 (1.2-2.5) | 2.57 (1.09-4.07) |
| CKD due to other and unspecified causes | Haiti | 54 (37.9-80.2) | 1.1 (0.7-1.8) | 96.2 (51.8-215.5) | 1.1 (0.6-2.5) | 0.34 (-1.01-1.7) |
| CKD due to other and unspecified causes | Honduras | 69.2 (54.6-88.7) | 3.1 (2.3-4.1) | 352.9 (249.8-499.7) | 6 (4.2-8.3) | 1.9 (0.81-3) |
| CKD due to other and unspecified causes | Hungary | 464.6 (408.3-515.2) | 3.5 (3.1-3.9) | 1197.4 (972.2-1431.5) | 5.6 (4.6-6.7) | 1.37 (0.32-2.43) |
| CKD due to other and unspecified causes | Iceland | 4.8 (3.8-5.6) | 1.6 (1.3-1.8) | 18.3 (14.6-21.6) | 2.6 (2.1-3) | 0.97 (0.14-1.8) |
| CKD due to other and unspecified causes | India | 17287 (13891.7-20895.6) | 3.7 (2.9-4.4) | 45581.9 (36262.8-56659.9) | 4.1 (3.3-5.2) | 0.37 (-0.59-1.34) |
| CKD due to other and unspecified causes | Indonesia | 834.5 (591.3-1074.1) | 0.5 (0.4-0.6) | 1069.9 (828.7-1382.8) | 0.5 (0.4-0.6) | 0.58 (-0.87-2.05) |
| CKD due to other and unspecified causes | Iran (Islamic Republic of) | 1057.9 (802.4-1459) | 4.4 (3.1-6.2) | 3318.8 (2560.8-4143) | 4.9 (3.7-6.2) | 0.34 (-1.09-1.79) |
| CKD due to other and unspecified causes | Iraq | 1077 (814.9-1421.4) | 12.3 (9-16.5) | 2770.4 (1774.3-3780) | 14 (9.5-18.7) | 0.09 (-1.4-1.6) |
| CKD due to other and unspecified causes | Ireland | 146.1 (118.6-169.8) | 3.9 (3.2-4.6) | 333.9 (258.7-407.9) | 3.9 (3-4.7) | 0.4 (-0.62-1.43) |
| CKD due to other and unspecified causes | Israel | 415.9 (334-494.8) | 9.6 (7.8-11.3) | 1193.1 (908.4-1445.7) | 8.4 (6.5-10.1) | 0.26 (-1.06-1.6) |
| CKD due to other and unspecified causes | Italy | 3045.1 (2453.6-3580.5) | 3.6 (2.9-4.2) | 7933.1 (5844.6-9781.6) | 3.9 (2.9-4.7) | 0.08 (-0.95-1.11) |
| CKD due to other and unspecified causes | Jamaica | 12.7 (10.3-15.8) | 0.7 (0.5-0.8) | 29.6 (19.8-43.2) | 1 (0.6-1.4) | 0.28 (-1.14-1.72) |
| CKD due to other and unspecified causes | Japan | 8037.4 (6536-9391.6) | 5.4 (4.4-6.3) | 24541.6 (17584.5-30257.9) | 4.4 (3.4-5.2) | -0.47 (-1.74-0.82) |
| CKD due to other and unspecified causes | Jordan | 93.1 (72.7-120.4) | 7.2 (5.3-9.7) | 422.8 (311.9-573) | 7.2 (5.3-9.7) | -0.08 (-1.49-1.35) |
| CKD due to other and unspecified causes | Kazakhstan | 474.6 (407.5-547.9) | 3.4 (2.9-4) | 1246.6 (1005.5-1485.2) | 7.8 (6.3-9.3) | 1.27 (0.02-2.53) |
| CKD due to other and unspecified causes | Kenya | 335.8 (252.5-492.6) | 3.9 (2.7-5.9) | 1133.6 (834.9-1536.9) | 5.7 (4-7.9) | 1.29 (0.09-2.5) |
| CKD due to other and unspecified causes | Kiribati | 0.9 (0.7-1.1) | 1.3 (1.1-1.7) | 1.6 (1-2.7) | 1.7 (1.1-2.9) | 0.66 (-1.1-2.45) |
| CKD due to other and unspecified causes | Kuwait | 59.8 (50.9-69.6) | 10.4 (8.2-12.5) | 126.7 (92.4-162.8) | 5.2 (3.7-6.9) | -1.59 (-2.96--0.19) |
| CKD due to other and unspecified causes | Kyrgyzstan | 105.2 (91.7-120.2) | 3 (2.6-3.4) | 321.7 (265-388.7) | 6.4 (5.3-7.8) | 0.63 (-0.66-1.94) |
| CKD due to other and unspecified causes | Lao People's Democratic Republic | 37.7 (22.5-52) | 0.9 (0.6-1.2) | 36.6 (25.1-51.7) | 0.6 (0.4-0.9) | -0.44 (-2.18-1.33) |
| CKD due to other and unspecified causes | Latvia | 39.9 (34-45.5) | 1.2 (1.1-1.4) | 137.9 (105.6-175.4) | 3.3 (2.5-4.1) | 1.6 (0.78-2.43) |
| CKD due to other and unspecified causes | Lebanon | 212 (151.8-290.4) | 11.2 (7.9-15.1) | 711.8 (536.1-930.8) | 10.8 (8.3-14) | 0.05 (-1.5-1.62) |
| CKD due to other and unspecified causes | Lesotho | 39.1 (28.9-54) | 4.9 (3.6-6.9) | 115.1 (75.5-162.6) | 11.7 (7.8-16.4) | 2.97 (1.49-4.47) |
| CKD due to other and unspecified causes | Liberia | 132.3 (100.5-168.7) | 9 (6.8-11.8) | 194.6 (134.4-264.1) | 8.8 (5.7-12.2) | 0.31 (-1.13-1.77) |
| CKD due to other and unspecified causes | Libya | 191.8 (141.4-270) | 10.1 (7.2-14.3) | 780.4 (477.9-1052.8) | 16.8 (10.6-22.6) | 1.91 (0.31-3.53) |
| CKD due to other and unspecified causes | Lithuania | 35.7 (30.4-42.3) | 0.9 (0.7-1) | 112.8 (85-144.7) | 2 (1.5-2.5) | 0.81 (0.05-1.57) |
| CKD due to other and unspecified causes | Luxembourg | 25 (20.4-29.1) | 5 (4.2-5.8) | 77.2 (59.7-95.2) | 6.1 (4.8-7.5) | 0.67 (-0.49-1.85) |
| CKD due to other and unspecified causes | Madagascar | 223 (170.2-306.4) | 4.1 (2.9-5.9) | 433.7 (305.8-601.7) | 4.1 (2.9-5.8) | 0.06 (-1.08-1.21) |
| CKD due to other and unspecified causes | Malawi | 272.7 (213.7-341.2) | 6.1 (4.6-8.1) | 518.3 (394.3-699.5) | 7 (5.2-9.4) | 0.32 (-0.92-1.57) |
| CKD due to other and unspecified causes | Malaysia | 37.1 (28.5-46.6) | 0.3 (0.2-0.4) | 88.2 (65.3-113.5) | 0.3 (0.2-0.4) | 0.37 (-1.36-2.12) |
| CKD due to other and unspecified causes | Maldives | 1.5 (1-1.9) | 0.8 (0.6-1) | 1.3 (1-1.7) | 0.4 (0.3-0.5) | -1.64 (-3.39-0.15) |
| CKD due to other and unspecified causes | Mali | 332.2 (255.9-414.9) | 7.3 (5.5-9.4) | 642.7 (504-836.1) | 6.8 (5-9.2) | 0 (-1.39-1.41) |
| CKD due to other and unspecified causes | Malta | 21.5 (17.4-25.1) | 5.8 (4.7-6.7) | 67.6 (52.1-82.4) | 6 (4.7-7.2) | 0.13 (-1.02-1.3) |
| CKD due to other and unspecified causes | Marshall Islands | 0.4 (0.2-0.7) | 1.3 (0.8-2.4) | 0.8 (0.2-2.4) | 2.2 (0.6-6.4) | 1.32 (-0.48-3.16) |
| CKD due to other and unspecified causes | Mauritania | 80.8 (58.4-109.3) | 7.9 (5.7-11) | 148.4 (96.2-220.4) | 7.3 (4.6-10.8) | -0.46 (-1.86-0.96) |
| CKD due to other and unspecified causes | Mauritius | 4.7 (3.8-5.8) | 0.6 (0.4-0.7) | 15.1 (11.6-19.3) | 1 (0.8-1.2) | 1.7 (-0.29-3.73) |
| CKD due to other and unspecified causes | Mexico | 3591.5 (2973.2-4231.2) | 8.8 (7.1-10.6) | 15725.9 (12317.9-19576.3) | 12.7 (9.9-15.8) | 1.74 (0.49-3.01) |
| CKD due to other and unspecified causes | Micronesia (Federated States of) | 1 (0.7-1.6) | 1.3 (0.9-2.2) | 1.5 (1-2.2) | 2 (1.4-3) | 1.29 (-0.56-3.17) |
| CKD due to other and unspecified causes | Monaco | 2.3 (1.7-3) | 2.9 (2.1-3.7) | 5.7 (4.2-7.2) | 4.5 (3.4-5.6) | 0.98 (-0.04-2.01) |
| CKD due to other and unspecified causes | Mongolia | 114.6 (89.7-142.6) | 10 (7.8-12.5) | 193.2 (151.2-236.6) | 8.9 (6.9-11.1) | -0.51 (-1.92-0.91) |
| CKD due to other and unspecified causes | Montenegro | 22.7 (17.1-29.4) | 3.9 (2.9-5) | 47.3 (33.4-63.1) | 5.5 (3.9-7.5) | 1.02 (-0.14-2.19) |
| CKD due to other and unspecified causes | Morocco | 1760.6 (1213.8-3154.6) | 12.9 (8.5-24.9) | 6554.2 (4814.8-8614.6) | 21.9 (16.2-29) | 1.55 (-0.11-3.24) |
| CKD due to other and unspecified causes | Mozambique | 286.6 (222.4-359.3) | 4.2 (3.1-5.7) | 628.2 (461.2-839) | 5.5 (3.9-7.7) | 1.25 (0.05-2.46) |
| CKD due to other and unspecified causes | Myanmar | 294.6 (177.3-415.1) | 0.7 (0.5-1) | 247 (180.3-317.9) | 0.5 (0.4-0.6) | -0.52 (-2.02-1) |
| CKD due to other and unspecified causes | Namibia | 29.2 (21.2-42.1) | 5.1 (3.6-7.3) | 74.9 (50.9-104.2) | 6.3 (4.4-8.7) | 0.35 (-0.96-1.69) |
| CKD due to other and unspecified causes | Nauru | 0.1 (0.1-0.2) | 1.7 (1.1-2.7) | 0.2 (0.1-0.3) | 2.4 (1.6-4.1) | 0.96 (-0.88-2.83) |
| CKD due to other and unspecified causes | Nepal | 391 (288-511.1) | 3.7 (2.7-5) | 1072.1 (745.4-1485.3) | 5.1 (3.6-7) | 1.02 (0.04-2.02) |
| CKD due to other and unspecified causes | Netherlands | 621.9 (503.1-731.5) | 3.2 (2.6-3.7) | 1997.7 (1637.3-2286.8) | 4.9 (4.1-5.6) | 0.98 (-0.14-2.11) |
| CKD due to other and unspecified causes | New Zealand | 97.9 (80.8-115) | 2.6 (2.2-3.1) | 318.6 (253.5-387.3) | 3.6 (2.9-4.4) | 0.92 (-0.14-2) |
| CKD due to other and unspecified causes | Nicaragua | 138.8 (115.8-181.2) | 7.4 (5.9-9.9) | 652.4 (494.6-850.5) | 13.4 (10.1-17.3) | 2.35 (1.14-3.58) |
| CKD due to other and unspecified causes | Niger | 255.2 (189.3-316.7) | 6 (4.6-7.9) | 463.2 (316.1-711) | 5.1 (3.5-7.7) | -0.31 (-1.72-1.13) |
| CKD due to other and unspecified causes | Nigeria | 2135.6 (1673.2-2667.8) | 4.5 (3.4-5.8) | 4077 (2874.9-5394.1) | 4.3 (3.1-5.8) | -0.18 (-1.61-1.27) |
| CKD due to other and unspecified causes | Niue | 0 (0-0) | 1.2 (0.9-1.6) | 0.1 (0-0.1) | 3.4 (2-5.3) | 1.49 (-0.31-3.33) |
| CKD due to other and unspecified causes | North Macedonia | 81.7 (65-100.7) | 4.8 (3.7-5.9) | 168 (124.8-225.9) | 6.3 (4.7-8.2) | 0.55 (-0.69-1.82) |
| CKD due to other and unspecified causes | Northern Mariana Islands | 0.4 (0.3-0.5) | 1.8 (1.2-2.5) | 0.9 (0.7-1.2) | 2.6 (1.8-3.7) | 1.07 (-0.89-3.06) |
| CKD due to other and unspecified causes | Norway | 103.3 (81.2-122.6) | 1.4 (1.1-1.6) | 315.3 (241.5-384.7) | 2.5 (1.9-3) | 1.07 (0.22-1.92) |
| CKD due to other and unspecified causes | Oman | 51.6 (36.6-76.8) | 7.7 (5.3-11.6) | 212.7 (162-269.6) | 13.7 (10.2-17.8) | 1.99 (0.44-3.57) |
| CKD due to other and unspecified causes | Pakistan | 3142.8 (2296.8-4205.8) | 5.2 (3.9-7.1) | 9100.5 (6920.4-11995.5) | 7.2 (5.5-9.6) | 0.75 (-0.32-1.83) |
| CKD due to other and unspecified causes | Palau | 0.1 (0.1-0.2) | 1.2 (0.8-1.7) | 0.3 (0.2-0.4) | 2.2 (1.5-3) | 1.48 (-0.3-3.3) |
| CKD due to other and unspecified causes | Palestine | 131.1 (94.1-178.4) | 15.8 (11.1-22.3) | 301.8 (240-378.7) | 14.9 (11.5-18.9) | -0.17 (-1.85-1.54) |
| CKD due to other and unspecified causes | Panama | 51 (42-60.1) | 3.3 (2.7-4) | 333.2 (249.3-434.3) | 7.4 (5.6-9.7) | 2.33 (1.17-3.5) |
| CKD due to other and unspecified causes | Papua New Guinea | 29.6 (14.8-43.5) | 0.7 (0.5-1) | 86.9 (62.3-111.3) | 0.9 (0.7-1.1) | 0.36 (-0.92-1.65) |
| CKD due to other and unspecified causes | Paraguay | 31.2 (24.3-38.6) | 1.2 (0.9-1.5) | 97.6 (69.6-136.2) | 1.7 (1.2-2.4) | 1.14 (-0.09-2.38) |
| CKD due to other and unspecified causes | Peru | 302 (242.7-365.3) | 2.1 (1.6-2.6) | 743.1 (519.3-1007.6) | 2.2 (1.5-3) | 0.07 (-1.23-1.38) |
| CKD due to other and unspecified causes | Philippines | 354.7 (271.8-436) | 0.7 (0.5-0.8) | 631.4 (482.1-817.5) | 0.7 (0.5-0.9) | 1.03 (-0.68-2.78) |
| CKD due to other and unspecified causes | Poland | 2378.3 (2033-2676.3) | 5.8 (5-6.5) | 2236.2 (1784.2-2700.4) | 3 (2.4-3.6) | -1.57 (-2.61--0.52) |
| CKD due to other and unspecified causes | Portugal | 729.5 (594.5-851.9) | 6.1 (5.1-7) | 2228.2 (1709-2701.3) | 6.8 (5.3-8.2) | 0.23 (-0.99-1.47) |
| CKD due to other and unspecified causes | Puerto Rico | 50.5 (39.6-63.6) | 1.5 (1.2-1.8) | 101.5 (81.4-127.3) | 1.4 (1.1-1.7) | 0.53 (-0.81-1.88) |
| CKD due to other and unspecified causes | Qatar | 10.2 (5.9-16.2) | 13.4 (7.6-21.5) | 70.4 (52.3-93) | 13.2 (9.5-17.6) | -0.47 (-2.17-1.25) |
| CKD due to other and unspecified causes | Republic of Korea | 559 (480.2-650.5) | 2.4 (2-2.9) | 1106.1 (758.5-1485.3) | 1.3 (0.9-1.7) | -1.32 (-2.43--0.19) |
| CKD due to other and unspecified causes | Republic of Moldova | 29.1 (24.8-33.6) | 0.7 (0.6-0.8) | 66.8 (53-83.1) | 1.2 (1-1.5) | 0.5 (-0.13-1.14) |
| CKD due to other and unspecified causes | Romania | 1243.3 (1055.9-1439.6) | 5.3 (4.5-6) | 2087.4 (1788.3-2407.5) | 5.4 (4.6-6.3) | -0.16 (-1.3-1) |
| CKD due to other and unspecified causes | Russian Federation | 2004.7 (1617.9-2413.6) | 1.2 (1-1.4) | 3194.2 (2441.1-4070.6) | 1.4 (1.1-1.7) | 0 (-0.79-0.8) |
| CKD due to other and unspecified causes | Rwanda | 236.4 (182.7-301.2) | 7.9 (5.9-10.2) | 353.4 (227.6-496.3) | 6.5 (4.1-9.3) | -1.4 (-2.65--0.14) |
| CKD due to other and unspecified causes | Saint Kitts and Nevis | 0.5 (0.4-0.6) | 1.3 (1-1.7) | 1 (0.7-1.3) | 1.6 (1.1-2.1) | 1.3 (-0.27-2.89) |
| CKD due to other and unspecified causes | Saint Lucia | 0.9 (0.8-1.2) | 1 (0.8-1.3) | 2.5 (1.8-3.5) | 1.1 (0.8-1.5) | 0.46 (-1.02-1.96) |
| CKD due to other and unspecified causes | Saint Vincent and the Grenadines | 0.6 (0.5-0.8) | 0.8 (0.7-1.1) | 1.5 (1.1-2) | 1.1 (0.9-1.5) | 1.57 (0.14-3.03) |
| CKD due to other and unspecified causes | Samoa | 1.6 (1.1-2.3) | 1.3 (0.8-1.9) | 2.6 (1.9-3.5) | 1.7 (1.2-2.2) | 0.76 (-0.99-2.54) |
| CKD due to other and unspecified causes | San Marino | 1 (0.8-1.2) | 2.6 (2-3.4) | 2.1 (1.3-2.9) | 1.9 (1.2-2.6) | 0.12 (-0.76-1) |
| CKD due to other and unspecified causes | Sao Tome and Principe | 7.5 (5.8-9.2) | 10.3 (7.8-13) | 12.9 (9.4-17) | 12.9 (9.1-17.2) | 0.88 (-0.59-2.37) |
| CKD due to other and unspecified causes | Saudi Arabia | 869.5 (622.1-1211.2) | 14.8 (10.3-21.1) | 4210.6 (2989.6-5505.7) | 25.5 (18.2-33.3) | 1.45 (-0.22-3.14) |
| CKD due to other and unspecified causes | Senegal | 294.3 (232.6-371.1) | 7.4 (5.7-9.4) | 567.2 (414.4-800.2) | 7.6 (5.5-10.7) | 0.09 (-1.28-1.48) |
| CKD due to other and unspecified causes | Serbia | 540.2 (415.2-689.3) | 6.1 (4.7-8) | 942.9 (745.6-1158.7) | 5.6 (4.5-6.9) | -0.06 (-1.24-1.13) |
| CKD due to other and unspecified causes | Seychelles | 0.2 (0.2-0.3) | 0.4 (0.3-0.5) | 0.6 (0.4-0.7) | 0.5 (0.4-0.7) | 1.22 (-0.66-3.14) |
| CKD due to other and unspecified causes | Sierra Leone | 152.1 (113-199.7) | 5.8 (4.3-7.6) | 221 (160.1-307.2) | 5.3 (3.8-7.4) | -0.09 (-1.43-1.27) |
| CKD due to other and unspecified causes | Singapore | 19.8 (15.1-24.8) | 1 (0.8-1.3) | 63.4 (44.8-85.5) | 0.8 (0.6-1.1) | -0.35 (-1.44-0.75) |
| CKD due to other and unspecified causes | Slovakia | 318.3 (260.3-376.3) | 5.5 (4.5-6.5) | 426.2 (325.1-539.8) | 4.6 (3.5-5.8) | -0.31 (-1.37-0.76) |
| CKD due to other and unspecified causes | Slovenia | 84.3 (74.5-93.3) | 3.6 (3.1-3.9) | 178 (135.1-234.3) | 3.3 (2.5-4.3) | 0.32 (-0.65-1.29) |
| CKD due to other and unspecified causes | Solomon Islands | 2.2 (0.8-3.8) | 0.9 (0.4-1.5) | 5.5 (3.9-7.4) | 1.1 (0.8-1.4) | 0.27 (-1.31-1.88) |
| CKD due to other and unspecified causes | Somalia | 185.1 (134.1-257.1) | 6.8 (4.7-9.8) | 465.9 (312.9-731.6) | 7.2 (4.6-10.8) | 0.26 (-0.96-1.49) |
| CKD due to other and unspecified causes | South Africa | 1032.2 (814.2-1312.9) | 4.9 (3.8-6.4) | 2998.3 (2427.8-3670.3) | 7.4 (5.9-9.2) | 1.4 (0.01-2.81) |
| CKD due to other and unspecified causes | South Sudan | 167 (120.2-230.9) | 6 (4.3-8.5) | 326.9 (224.4-444.6) | 8.2 (5.7-11.3) | 0.79 (-0.47-2.06) |
| CKD due to other and unspecified causes | Spain | 3361.8 (2791.3-3912) | 6.6 (5.5-7.6) | 7438 (5637-9087.5) | 5.2 (4.1-6.2) | -0.35 (-1.51-0.83) |
| CKD due to other and unspecified causes | Sri Lanka | 52.1 (38.5-67.7) | 0.4 (0.3-0.6) | 64.9 (41.6-95.8) | 0.3 (0.2-0.4) | -0.61 (-2.26-1.06) |
| CKD due to other and unspecified causes | Sudan | 685.1 (483.6-1125.3) | 6.7 (4.4-12.7) | 1731.5 (1213.4-2335.4) | 9.1 (6.4-12.6) | 0.88 (-0.62-2.39) |
| CKD due to other and unspecified causes | Suriname | 2.5 (1.9-3.2) | 0.8 (0.6-1.1) | 7.5 (5.1-10.6) | 1.2 (0.9-1.7) | 1.34 (-0.12-2.83) |
| CKD due to other and unspecified causes | Sweden | 277 (227.2-323.7) | 1.7 (1.4-1.9) | 1153.3 (862.6-1410.5) | 4 (3.1-4.9) | 1.71 (0.82-2.6) |
| CKD due to other and unspecified causes | Switzerland | 347.4 (278.6-411.2) | 3.1 (2.6-3.6) | 1207.9 (933.9-1432.4) | 4.7 (3.7-5.5) | 1.13 (0.05-2.23) |
| CKD due to other and unspecified causes | Syrian Arab Republic | 782.5 (600.6-988.9) | 13.9 (10.2-18.6) | 1711.9 (1180.8-2336.8) | 16.6 (11.3-22.6) | 0.18 (-1.45-1.84) |
| CKD due to other and unspecified causes | Taiwan (Province of China) | 89.3 (73.5-109.7) | 0.6 (0.5-0.8) | 190.7 (135.8-256.4) | 0.5 (0.4-0.6) | -0.18 (-1.77-1.43) |
| CKD due to other and unspecified causes | Tajikistan | 40.1 (32.9-55.3) | 1.2 (0.9-1.7) | 100.7 (70.5-145.1) | 1.6 (1.1-2.2) | 0.12 (-0.59-0.83) |
| CKD due to other and unspecified causes | Thailand | 197 (147.9-254.7) | 0.4 (0.3-0.6) | 357.1 (251.2-509) | 0.4 (0.3-0.6) | -0.03 (-1.7-1.66) |
| CKD due to other and unspecified causes | Timor-Leste | 4.9 (2.8-6.5) | 0.6 (0.4-0.8) | 5.5 (3.9-7.7) | 0.5 (0.3-0.7) | 0.33 (-1.29-1.98) |
| CKD due to other and unspecified causes | Togo | 89.1 (69.7-110.7) | 5.7 (4.3-7.4) | 218.7 (152.8-304.6) | 6.2 (4.4-8.6) | 0.18 (-1.16-1.55) |
| CKD due to other and unspecified causes | Tokelau | 0 (0-0) | 1 (0.6-1.8) | 0 (0-0) | 2.3 (1.7-3.5) | 0.96 (-0.74-2.68) |
| CKD due to other and unspecified causes | Tonga | 0.3 (0.2-0.4) | 0.4 (0.3-0.6) | 0.6 (0.4-0.8) | 0.6 (0.4-0.9) | 0.74 (-0.8-2.31) |
| CKD due to other and unspecified causes | Trinidad and Tobago | 5.7 (4.5-6.9) | 0.7 (0.5-0.8) | 20.6 (14.4-29.3) | 1.1 (0.8-1.6) | 1.78 (0.33-3.25) |
| CKD due to other and unspecified causes | Tunisia | 426.5 (311.6-633.3) | 10 (7.2-14.5) | 1653.3 (1153.7-2323.6) | 14.2 (10-19.7) | 0.91 (-0.57-2.42) |
| CKD due to other and unspecified causes | Turkey | 3450.5 (2615.7-4662.4) | 10.7 (7.9-14.9) | 8140.1 (6064.7-10603.2) | 9.7 (7.3-12.8) | 0.17 (-1.29-1.66) |
| CKD due to other and unspecified causes | Turkmenistan | 138.4 (124.4-153.1) | 5.8 (5.2-6.5) | 478.9 (366.8-619.8) | 11.6 (8.9-14.9) | 1.2 (-0.22-2.65) |
| CKD due to other and unspecified causes | Tuvalu | 0.1 (0.1-0.1) | 1.2 (0.9-1.9) | 0.2 (0.1-0.2) | 1.5 (1.1-2.2) | 0.92 (-0.81-2.67) |
| CKD due to other and unspecified causes | Uganda | 309.5 (232.9-403.6) | 4.5 (3.2-6.1) | 858.2 (633.8-1163.2) | 5.7 (4.1-8.1) | 0.36 (-0.84-1.58) |
| CKD due to other and unspecified causes | Ukraine | 28.1 (23.7-32.6) | 0 (0-0.1) | 513.2 (373.1-686.7) | 0.8 (0.6-1.1) | 2.08 (1.66-2.5) |
| CKD due to other and unspecified causes | United Arab Emirates | 30.5 (20-44.5) | 7.5 (4.9-11.2) | 226.4 (139.1-316.6) | 12 (6.7-16.9) | 2.89 (1.24-4.57) |
| CKD due to other and unspecified causes | United Kingdom | 2178.1 (1818.9-2463.5) | 2.4 (2-2.7) | 5196.9 (4233.2-6014.8) | 3.4 (2.8-3.8) | 0.9 (0.05-1.75) |
| CKD due to other and unspecified causes | United Republic of Tanzania | 887.8 (689.7-1147.8) | 8.4 (6.4-11.2) | 2095 (1555.8-2802) | 8.8 (6.4-11.9) | 0.05 (-1.44-1.56) |
| CKD due to other and unspecified causes | United States of America | 4133 (2512.4-5837.9) | 1.3 (0.8-1.8) | 7901.9 (6483.8-9348.5) | 1.3 (1.1-1.6) | 2.19 (0.97-3.43) |
| CKD due to other and unspecified causes | United States Virgin Islands | 0.4 (0.3-0.5) | 0.5 (0.4-0.7) | 0.7 (0.4-1) | 0.4 (0.3-0.6) | 0.38 (-1.03-1.82) |
| CKD due to other and unspecified causes | Uruguay | 230 (209.5-252.1) | 6 (5.5-6.6) | 422.2 (335.9-515.5) | 6.5 (5.3-7.8) | 0.37 (-0.86-1.62) |
| CKD due to other and unspecified causes | Uzbekistan | 414.3 (294-626.1) | 3.3 (2.2-5.3) | 2320 (1886.9-2768.6) | 9.2 (7.5-10.9) | 1.35 (0.01-2.72) |
| CKD due to other and unspecified causes | Vanuatu | 0.9 (0.6-1.5) | 0.9 (0.5-1.5) | 2.9 (1.9-4.4) | 1.3 (0.9-2.1) | 1.05 (-0.62-2.75) |
| CKD due to other and unspecified causes | Venezuela (Bolivarian Republic of) | 410 (343.9-484.9) | 4.1 (3.3-4.9) | 2870.6 (2067.1-3933.3) | 9.9 (7.1-13.6) | 2.15 (0.97-3.35) |
| CKD due to other and unspecified causes | Viet Nam | 224.8 (137.5-299.9) | 0.4 (0.3-0.5) | 329.5 (219.3-447.7) | 0.4 (0.3-0.5) | 0.34 (-1.31-2.02) |
| CKD due to other and unspecified causes | Yemen | 345.6 (229.2-631.5) | 6.7 (4.3-13.7) | 923 (609.1-1576.4) | 7.2 (4.7-12.3) | 0.09 (-1.24-1.44) |
| CKD due to other and unspecified causes | Zambia | 234.8 (183.1-292.2) | 7.1 (5.5-9.2) | 546 (367.6-818.1) | 7.7 (5.3-11.2) | 0.09 (-1.15-1.34) |
| CKD due to other and unspecified causes | Zimbabwe | 216.9 (163.5-289.6) | 6.1 (4.5-8.5) | 637.2 (453.3-860.1) | 10.1 (7.2-13.9) | 1.63 (0.19-3.08) |

Table S3. Predicted trends of CKD incidence and deaths in the next decade (2021-2031).

| Year | Number of incidence | Number of deaths | Type |
| --- | --- | --- | --- |
| 1990 | 7790704.9 | 552672.8 | Actual |
| 1991 | 8015667.1 | 565920.1 | Actual |
| 1992 | 8243753.2 | 581769.6 | Actual |
| 1993 | 8472928.4 | 597750.9 | Actual |
| 1994 | 8702549.7 | 614246.1 | Actual |
| 1995 | 8933923.8 | 634204.5 | Actual |
| 1996 | 9172943.6 | 652310.8 | Actual |
| 1997 | 9424628.0 | 671585.6 | Actual |
| 1998 | 9685681.3 | 700270.6 | Actual |
| 1999 | 9956485.2 | 729117.9 | Actual |
| 2000 | 10237730.3 | 764663.2 | Actual |
| 2001 | 10540857.3 | 793950.1 | Actual |
| 2002 | 10867175.9 | 821501.6 | Actual |
| 2003 | 11207543.4 | 850620.5 | Actual |
| 2004 | 11558354.8 | 874341.1 | Actual |
| 2005 | 11909103.3 | 899261.3 | Actual |
| 2006 | 12281082.7 | 919424.8 | Actual |
| 2007 | 12688414.9 | 948517.0 | Actual |
| 2008 | 13115766.3 | 984299.9 | Actual |
| 2009 | 13557767.0 | 1019429.4 | Actual |
| 2010 | 13995322.7 | 1058754.9 | Actual |
| 2011 | 14453135.7 | 1099616.4 | Actual |
| 2012 | 14951800.4 | 1136307.9 | Actual |
| 2013 | 15472942.4 | 1184499.7 | Actual |
| 2014 | 16009965.4 | 1229564.1 | Actual |
| 2015 | 16537858.6 | 1280016.6 | Actual |
| 2016 | 17088768.0 | 1329849.9 | Actual |
| 2017 | 17677953.3 | 1369779.7 | Actual |
| 2018 | 18278682.3 | 1410445.4 | Actual |
| 2019 | 18879365.6 | 1462675.3 | Actual |
| 2020 | 19469747.8 | 1499449.3 | Actual |
| 2021 | 19935037.8 | 1527638.7 | Actual |
| 2022 | 20310903.7 | 1555828.1 | Forecast |
| 2023 | 20622843.6 | 1584017.5 | Forecast |
| 2024 | 20889084.9 | 1612206.9 | Forecast |
| 2025 | 21122657.9 | 1640396.3 | Forecast |
| 2026 | 21332877.5 | 1668585.6 | Forecast |
| 2027 | 21526402.6 | 1696775.0 | Forecast |
| 2028 | 21707993.3 | 1724964.4 | Forecast |
| 2029 | 21881052.5 | 1753153.8 | Forecast |
| 2030 | 22048012.9 | 1781343.2 | Forecast |
| 2031 | 22210613.5 | 1809532.6 | Forecast |

Table S4. Changes in incidence cases and deaths number according to population-level determinants from 1990 to 2021.

| Category | CKD | | | | | | | |
| --- | --- | --- | --- | --- | --- | --- | --- | --- |
|  | Incidence cases | | | | Deaths | | | |
|  | Overall difference | Aging | Population | Epidemiological change | Overall difference | Aging | Population | Epidemiological change |
| Location |  |  |  |  |  |  |  |  |
| High-middle SDI | 11750880247.000 | 762174860.659 (6.49%) | 2052264622.235 (17.46%) | 8936440764.133 (76.05%) | 919088365.100 | 150472117.917 (16.37%) | 142417924.332 (15.5%) | 626198322.828 (68.13%) |
| Low-middle SDI | 14055828351.000 | 1352412379.396 (9.62%) | 4858994272.354 (34.57%) | 7844421699.276 (55.81%) | 747767806.200 | 112565369.289 (15.05%) | 249837366.798 (33.41%) | 385365070.091 (51.54%) |
| Low SDI | 6315372670.000 | 128278170.124 (2.03%) | 3089645757.705 (48.92%) | 3097448742.231 (49.05%) | 258440113.400 | 6628503.802 (2.56%) | 134660570.111 (52.11%) | 117151039.523 (45.33%) |
| High SDI | 9225983751.000 | 712318634.879 (7.72%) | 1715452799.562 (18.59%) | 6798212316.784 (73.69%) | 650987243.000 | 101857675.533 (15.65%) | 110777631.025 (17.02%) | 438351936.444 (67.34%) |
| Middle SDI | 16308966996.000 | 3097383765.152 (18.99%) | 4205984893.521 (25.79%) | 9005598336.853 (55.22%) | 818799175.000 | 223156058.326 (27.25%) | 205659853.785 (25.12%) | 389983262.921 (47.63%) |
| Sex |  |  |  |  |  |  |  |  |
| Both | 15594294730.000 | 694262917.455 (4.45%) | 4673211442.074 (29.97%) | 10226820370.671 (65.58%) | 1043730054.000 | 152328352.393 (14.59%) | 272825165.11 (26.14%) | 618576536.748 (59.27%) |
| Male | 3452870054.000 | 126796408.142 (3.67%) | 1033856681.814 (29.94%) | 2292216963.959 (66.39%) | 277573250.300 | 38017424.62 (13.7%) | 72557092.008 (26.14%) | 166998733.669 (60.16%) |
| Female | 4349610591.000 | 219963410.414 (5.06%) | 1306709616.215 (30.04%) | 2822937564.314 (64.9%) | 244698956.700 | 37966204.58 (15.52%) | 63922004.19 (26.12%) | 142810747.954 (58.36%) |

%: contribute to the total changes

Table S5. Age, period and cohort effects of CKD incidence and mortality rate in global.

| Factors | CKD incidence | | | CKD mortality rate | | |
| --- | --- | --- | --- | --- | --- | --- |
|  | RR | 95% CI | | RR | 95% CI | |
|  |  | Lower | Upper |  | Lower | Upper |
| Age |  |  |  |  |  |  |
| 15-19 | 0.01 | 0.01 | 0.01 | 0.00 | 0.00 | 0.00 |
| 20-24 | 0.01 | 0.01 | 0.01 | 0.00 | 0.00 | 0.00 |
| 25-29 | 0.01 | 0.01 | 0.01 | 0.00 | 0.00 | 0.00 |
| 30-34 | 0.02 | 0.01 | 0.02 | 0.00 | 0.00 | 0.00 |
| 35-39 | 0.03 | 0.03 | 0.03 | 0.00 | 0.00 | 0.00 |
| 40-44 | 0.06 | 0.05 | 0.06 | 0.00 | 0.00 | 0.00 |
| 45-49 | 0.10 | 0.09 | 0.10 | 0.01 | 0.00 | 0.01 |
| 50-54 | 0.17 | 0.16 | 0.18 | 0.01 | 0.01 | 0.01 |
| 55-59 | 0.29 | 0.27 | 0.30 | 0.02 | 0.01 | 0.02 |
| 60-64 | 0.48 | 0.46 | 0.50 | 0.02 | 0.02 | 0.03 |
| 65-69 | 0.80 | 0.77 | 0.84 | 0.04 | 0.04 | 0.05 |
| 70-74 | 1.34 | 1.28 | 1.40 | 0.08 | 0.06 | 0.09 |
| 75-79 | 2.17 | 2.07 | 2.28 | 0.15 | 0.13 | 0.18 |
| 80-84 | 3.70 | 3.53 | 3.88 | 0.37 | 0.31 | 0.44 |
| 85-89 | 6.18 | 5.89 | 6.48 | 1.24 | 1.05 | 1.47 |
| 90-94 | 12.83 | 12.21 | 13.48 | 5.24 | 4.43 | 6.20 |
| Period |  |  |  |  |  |  |
| 1992.5 | 1.03 | 1.00 | 1.07 | 1.13 | 1.02 | 1.25 |
| 1997.5 | 1.08 | 1.05 | 1.11 | 1.08 | 1.00 | 1.17 |
| 2002.5 | 1.05 | 1.03 | 1.08 | 1.04 | 0.98 | 1.11 |
| 2007.5 | 1.00 | 1.00 | 1.00 | 1.00 | 1.00 | 1.00 |
| 2012.5 | 0.92 | 0.90 | 0.94 | 0.89 | 0.84 | 0.94 |
| 2017.5 | 0.83 | 0.80 | 0.85 | 0.81 | 0.75 | 0.87 |
| 2022.5 | 0.74 | 0.72 | 0.76 | 0.69 | 0.63 | 0.76 |
| Cohort |  |  |  |  |  |  |
| 1900 | 5.70 | 5.35 | 6.09 | 3.80 | 3.17 | 4.56 |
| 1905 | 3.94 | 3.73 | 4.16 | 3.04 | 2.55 | 3.62 |
| 1910 | 2.92 | 2.77 | 3.07 | 2.57 | 2.16 | 3.06 |
| 1915 | 2.42 | 2.30 | 2.55 | 2.35 | 1.97 | 2.80 |
| 1920 | 2.20 | 2.10 | 2.31 | 2.28 | 1.92 | 2.71 |
| 1925 | 1.83 | 1.75 | 1.92 | 1.91 | 1.61 | 2.26 |
| 1930 | 1.55 | 1.48 | 1.62 | 1.65 | 1.39 | 1.95 |
| 1935 | 1.43 | 1.37 | 1.50 | 1.57 | 1.32 | 1.85 |
| 1940 | 1.36 | 1.30 | 1.42 | 1.46 | 1.23 | 1.72 |
| 1945 | 1.35 | 1.29 | 1.42 | 1.44 | 1.22 | 1.71 |
| 1950 | 1.21 | 1.15 | 1.27 | 1.25 | 1.05 | 1.49 |
| 1955 | 1.04 | 0.99 | 1.09 | 1.07 | 0.89 | 1.28 |
| 1960 | 1.00 | 1.00 | 1.00 | 1.00 | 1.00 | 1.00 |
| 1965 | 0.93 | 0.88 | 0.99 | 0.91 | 0.73 | 1.13 |
| 1970 | 0.86 | 0.80 | 0.93 | 0.81 | 0.62 | 1.05 |
| 1975 | 0.87 | 0.79 | 0.95 | 0.79 | 0.58 | 1.08 |
| 1980 | 0.91 | 0.82 | 1.02 | 0.79 | 0.55 | 1.15 |
| 1985 | 0.90 | 0.78 | 1.03 | 0.74 | 0.47 | 1.15 |
| 1990 | 0.83 | 0.70 | 1.00 | 0.66 | 0.39 | 1.12 |
| 1995 | 0.88 | 0.72 | 1.08 | 0.67 | 0.36 | 1.25 |
| 2000 | 0.96 | 0.76 | 1.22 | 0.69 | 0.33 | 1.44 |
| 2005 | 1.01 | 0.77 | 1.33 | 0.67 | 0.26 | 1.73 |
| 2010 | 1.00 | 0.71 | 1.42 | 0.58 | 0.15 | 2.28 |
| 2015 | 0.98 | 0.59 | 1.62 | 0.48 | 0.07 | 3.60 |

Table S6. The trends in slope index of inequality and concentration index of CKD crude incidence and mortality rates from 1990 to 2021.

| year | CKD | |
| --- | --- | --- |
|  | Incidence | Mortality |
|  | SII | SII |
| 1990 | 138.02 (115.34, 160.71) | -3.55 (-5.93, -1.17) |
| 1991 | 143.4 (120.23, 166.57) | -3.41 (-5.82, -1) |
| 1992 | 147.74 (123.64, 171.84) | -3.37 (-5.83, -0.91) |
| 1993 | 154.69 (130.16, 179.23) | -3.08 (-5.52, -0.64) |
| 1994 | 162.15 (137.01, 187.3) | -2.84 (-5.29, -0.39) |
| 1995 | 170.87 (145.04, 196.69) | -2.22 (-4.73, 0.28) |
| 1996 | 179.53 (153.01, 206.05) | -1.76 (-4.29, 0.76) |
| 1997 | 187.97 (160.74, 215.2) | -1.25 (-3.83, 1.33) |
| 1998 | 195.29 (168.89, 221.69) | -0.54 (-3.2, 2.12) |
| 1999 | 204.47 (177.61, 231.34) | 0.41 (-2.38, 3.2) |
| 2000 | 212.63 (185.64, 239.63) | 1.72 (-1.24, 4.69) |
| 2001 | 221.47 (193.68, 249.26) | 2.71 (-0.35, 5.76) |
| 2002 | 234.49 (205.46, 263.51) | 3.48 (0.32, 6.64) |
| 2003 | 244.2 (214.11, 274.29) | 4.01 (0.77, 7.24) |
| 2004 | 254.16 (223.72, 284.6) | 4.12 (0.91, 7.33) |
| 2005 | 261.12 (230.89, 291.36) | 4.57 (1.38, 7.77) |
| 2006 | 271.78 (240.47, 303.08) | 4.97 (1.55, 8.39) |
| 2007 | 281.7 (250.01, 313.4) | 5.4 (2.01, 8.79) |
| 2008 | 288.79 (256.77, 320.8) | 5.98 (2.47, 9.49) |
| 2009 | 296.73 (264.85, 328.6) | 6.44 (2.87, 10.02) |
| 2010 | 302.71 (270.02, 335.41) | 7.13 (3.37, 10.88) |
| 2011 | 311.58 (277.88, 345.28) | 7.79 (3.92, 11.65) |
| 2012 | 317.55 (282.68, 352.41) | 8.27 (4.41, 12.12) |
| 2013 | 324.53 (289.43, 359.63) | 8.78 (4.72, 12.83) |
| 2014 | 332.17 (296.72, 367.63) | 9.51 (5.26, 13.75) |
| 2015 | 337.98 (302.17, 373.78) | 10.39 (5.99, 14.78) |
| 2016 | 344.54 (308.89, 380.18) | 11.03 (6.5, 15.56) |
| 2017 | 352.08 (314.53, 389.62) | 11.53 (6.95, 16.1) |
| 2018 | 360.82 (323.53, 398.1) | 11.97 (7.1, 16.84) |
| 2019 | 372.71 (335.11, 410.31) | 12.6 (7.7, 17.5) |
| 2020 | 381.34 (343.37, 419.31) | 12.23 (7.33, 17.12) |
| 2021 | 382.35 (343.74, 420.97) | 12.09 (7.1, 17.07) |
|  | CI | CI |
| 1990 | -0.26 (-0.26, -0.26) | 0.05 (-0.14, 0.24) |
| 2021 | -0.26 (-0.26, -0.26) | -0.09 (-0.28, 0.1) |

SII, slope index of inequality.

CI, concentration index.

Table S7. Contribution of different causes to chronic kidney disease incident cases and deaths in different regions and periods

| Year | Location | Measure | CKD due to hypertension (%) | CKD due to glomerulonephritis (%) | CKD due to other and unspecified causes (%) | CKD due to diabetes mellitus type 1 (%) | CKD due to diabetes mellitus type 2 (%) |
| --- | --- | --- | --- | --- | --- | --- | --- |
| 1990 | Organization of Islamic cooperation | Incident cases | 5 | 5.7 | 79.3 | 1.6 | 8.4 |
| 1990 | Association of Southeast Asian nations | Incident cases | 4.9 | 5.4 | 79.9 | 1.5 | 8.3 |
| 1990 | Nordic region | Incident cases | 7.4 | 0.6 | 81.9 | 0.3 | 9.8 |
| 1990 | Health system grouping levels | Incident cases | 6 | 3.1 | 80.4 | 0.8 | 9.7 |
| 1990 | Gulf cooperation council | Incident cases | 5.2 | 4.6 | 80.2 | 1.2 | 8.9 |
| 1990 | High-middle SDI | Incident cases | 6.1 | 2.6 | 80.7 | 0.6 | 10 |
| 1990 | Commonwealth | Incident cases | 5.5 | 4.5 | 79.8 | 1.2 | 9 |
| 1990 | Middle SDI | Incident cases | 5.3 | 4.3 | 80 | 1.1 | 9.3 |
| 1990 | Low SDI | Incident cases | 4.6 | 7.6 | 77.8 | 2.1 | 7.9 |
| 1990 | Low-middle SDI | Incident cases | 5 | 5.4 | 79.5 | 1.4 | 8.7 |
| 1990 | High SDI | Incident cases | 6.8 | 1.1 | 81.3 | 0.3 | 10.4 |
| 1990 | World bank income levels | Incident cases | 6 | 3.1 | 80.4 | 0.8 | 9.7 |
| 1990 | European Union | Incident cases | 7 | 0.9 | 81.5 | 0.3 | 10.3 |
| 1990 | Unknown region | Incident cases | 5.3 | 4.3 | 80.1 | 1.2 | 9.1 |
| 1990 | OECD Countries | Incident cases | 6.8 | 1.3 | 81.2 | 0.4 | 10.3 |
| 1990 | Four World Regions | Incident cases | 6 | 3.1 | 80.4 | 0.8 | 9.7 |
| 1990 | African Union | Incident cases | 4.9 | 6.6 | 78.4 | 1.9 | 8.2 |
| 1990 | G20 | Incident cases | 6.2 | 2.6 | 80.7 | 0.7 | 9.9 |
| 1990 | Sahel region | Incident cases | 4.6 | 8.1 | 77.4 | 2.3 | 7.7 |
| 1990 | WHO region | Incident cases | 6 | 3.1 | 80.4 | 0.8 | 9.7 |
| 1990 | World Bank regions | Incident cases | 6 | 3.1 | 80.4 | 0.8 | 9.7 |
| 1990 | South Asia | Incident cases | 5.1 | 5.3 | 79.6 | 1.3 | 8.8 |
| 1990 | North Africa and Middle East | Incident cases | 5.4 | 4.1 | 80.2 | 1.1 | 9.3 |
| 1990 | Sub-Saharan Africa | Incident cases | 4.5 | 8.3 | 77.2 | 2.4 | 7.5 |
| 1990 | Latin America and Caribbean | Incident cases | 5.3 | 4.1 | 80.3 | 1 | 9.3 |
| 1990 | Southeast Asia, East Asia, and Oceania | Incident cases | 5.5 | 3.9 | 80 | 0.9 | 9.6 |
| 1990 | Iceland | Incident cases | 7.2 | 1 | 81.4 | 0.3 | 10.1 |
| 1990 | Central Europe, Eastern Europe, and Central Asia | Incident cases | 5.5 | 3.6 | 80.9 | 1 | 9.2 |
| 1990 | High-income | Incident cases | 6.9 | 1 | 81.3 | 0.3 | 10.4 |
| 2021 | Organization of Islamic cooperation | Incident cases | 5.6 | 3.3 | 80.9 | 0.9 | 9.4 |
| 2021 | Association of Southeast Asian nations | Incident cases | 5.7 | 2.5 | 81.4 | 0.7 | 9.7 |
| 2021 | Nordic region | Incident cases | 7.6 | 0.5 | 81.6 | 0.4 | 9.9 |
| 2021 | Health system grouping levels | Incident cases | 6.4 | 1.8 | 81.2 | 0.5 | 10.1 |
| 2021 | Gulf cooperation council | Incident cases | 5.5 | 2.5 | 82 | 0.6 | 9.4 |
| 2021 | High-middle SDI | Incident cases | 6.7 | 1.2 | 81.5 | 0.3 | 10.3 |
| 2021 | Commonwealth | Incident cases | 6 | 2.8 | 80.8 | 0.7 | 9.6 |
| 2021 | Middle SDI | Incident cases | 6.1 | 1.9 | 81.4 | 0.4 | 10.2 |
| 2021 | Low SDI | Incident cases | 5 | 6 | 79.2 | 1.6 | 8.1 |
| 2021 | Low-middle SDI | Incident cases | 5.7 | 3.1 | 81 | 0.8 | 9.5 |
| 2021 | High SDI | Incident cases | 7.2 | 0.7 | 81.3 | 0.3 | 10.5 |
| 2021 | World bank income levels | Incident cases | 6.4 | 1.8 | 81.2 | 0.5 | 10.1 |
| 2021 | European Union | Incident cases | 7.4 | 0.5 | 81.6 | 0.3 | 10.1 |
| 2021 | Unknown region | Incident cases | 5.7 | 2.5 | 81.3 | 0.6 | 9.9 |
| 2021 | OECD Countries | Incident cases | 7.1 | 0.8 | 81.4 | 0.3 | 10.4 |
| 2021 | Four World Regions | Incident cases | 6.4 | 1.8 | 81.2 | 0.5 | 10.1 |
| 2021 | African Union | Incident cases | 5.3 | 4.6 | 80 | 1.3 | 8.9 |
| 2021 | G20 | Incident cases | 6.7 | 1.3 | 81.3 | 0.4 | 10.3 |
| 2021 | Sahel region | Incident cases | 4.6 | 7.3 | 78.4 | 2 | 7.6 |
| 2021 | WHO region | Incident cases | 6.4 | 1.8 | 81.2 | 0.5 | 10.1 |
| 2021 | World Bank regions | Incident cases | 6.4 | 1.8 | 81.2 | 0.5 | 10.1 |
| 2021 | South Asia | Incident cases | 5.8 | 2.8 | 81 | 0.6 | 9.7 |
| 2021 | North Africa and Middle East | Incident cases | 5.9 | 2.1 | 81.4 | 0.5 | 10 |
| 2021 | Sub-Saharan Africa | Incident cases | 4.9 | 6.4 | 78.9 | 1.9 | 7.9 |
| 2021 | Latin America and Caribbean | Incident cases | 6 | 1.9 | 81.5 | 0.5 | 10.1 |
| 2021 | Southeast Asia, East Asia, and Oceania | Incident cases | 6.4 | 1.6 | 81.3 | 0.4 | 10.4 |
| 2021 | Iceland | Incident cases | 7.5 | 0.6 | 81.3 | 0.3 | 10.2 |
| 2021 | Central Europe, Eastern Europe, and Central Asia | Incident cases | 6.2 | 1.7 | 81.6 | 0.6 | 9.9 |
| 2021 | High-income | Incident cases | 7.4 | 0.6 | 81.3 | 0.3 | 10.4 |
| 1990 | Organization of Islamic cooperation | Deaths | 30.5 | 15.8 | 28.3 | 6 | 19.4 |
| 1990 | Association of Southeast Asian nations | Deaths | 44.3 | 7.9 | 3.7 | 15 | 29.1 |
| 1990 | Nordic region | Deaths | 16.7 | 18 | 44.7 | 2.2 | 18.4 |
| 1990 | Health system grouping levels | Deaths | 27 | 15 | 22.3 | 8.9 | 26.8 |
| 1990 | Gulf cooperation council | Deaths | 28.9 | 9.4 | 36.3 | 3.3 | 22.1 |
| 1990 | High-middle SDI | Deaths | 25.1 | 13.2 | 25.5 | 9.6 | 26.6 |
| 1990 | Commonwealth | Deaths | 21.8 | 20.8 | 27.3 | 7.4 | 22.6 |
| 1990 | Middle SDI | Deaths | 30.7 | 11.6 | 13.2 | 12.8 | 31.7 |
| 1990 | Low SDI | Deaths | 23.7 | 27.2 | 21.7 | 6.8 | 20.7 |
| 1990 | Low-middle SDI | Deaths | 25.5 | 16.9 | 26.3 | 8.3 | 23 |
| 1990 | High SDI | Deaths | 26 | 13 | 31.2 | 3.4 | 26.4 |
| 1990 | World bank income levels | Deaths | 27 | 15 | 22.3 | 8.9 | 26.8 |
| 1990 | European Union | Deaths | 19.1 | 18 | 45.4 | 1.8 | 15.7 |
| 1990 | Unknown region | Deaths | 29.2 | 11 | 39.1 | 2.5 | 18.2 |
| 1990 | OECD Countries | Deaths | 24.9 | 15.1 | 34.6 | 2.7 | 22.6 |
| 1990 | Four World Regions | Deaths | 27 | 15 | 22.3 | 8.9 | 26.7 |
| 1990 | African Union | Deaths | 27.2 | 26.1 | 23.9 | 4.9 | 17.8 |
| 1990 | G20 | Deaths | 25.7 | 13.1 | 22.6 | 9.9 | 28.8 |
| 1990 | Sahel region | Deaths | 32.8 | 28.7 | 21.4 | 3.9 | 13.2 |
| 1990 | WHO region | Deaths | 27 | 15.1 | 22.4 | 8.9 | 26.6 |
| 1990 | World Bank regions | Deaths | 27 | 15 | 22.3 | 8.9 | 26.8 |
| 1990 | South Asia | Deaths | 17.9 | 16 | 31.7 | 9.1 | 25.3 |
| 1990 | North Africa and Middle East | Deaths | 31 | 9.1 | 38.5 | 2.3 | 19.1 |
| 1990 | Sub-Saharan Africa | Deaths | 26.4 | 30.6 | 19.2 | 5.7 | 18 |
| 1990 | Latin America and Caribbean | Deaths | 25.8 | 23.6 | 16.2 | 7 | 27.4 |
| 1990 | Southeast Asia, East Asia, and Oceania | Deaths | 33.9 | 5.7 | 4.9 | 17.4 | 38.1 |
| 1990 | Iceland | Deaths | 28.6 | 16.4 | 43.2 | 1.1 | 10.7 |
| 1990 | Central Europe, Eastern Europe, and Central Asia | Deaths | 12.7 | 30.5 | 40.2 | 6.5 | 10.1 |
| 1990 | High-income | Deaths | 25.8 | 13.1 | 35.1 | 2.5 | 23.6 |
| 2021 | Organization of Islamic cooperation | Deaths | 32.7 | 13.1 | 25.9 | 5.8 | 22.5 |
| 2021 | Association of Southeast Asian nations | Deaths | 46.8 | 4.6 | 1.8 | 12.3 | 34.6 |
| 2021 | Nordic region | Deaths | 18.1 | 15.3 | 46.6 | 1.1 | 18.8 |
| 2021 | Health system grouping levels | Deaths | 29.7 | 12.7 | 20.2 | 6.2 | 31.2 |
| 2021 | Gulf cooperation council | Deaths | 28.3 | 9.3 | 34.4 | 5 | 23 |
| 2021 | High-middle SDI | Deaths | 28.5 | 9.9 | 24.5 | 5.6 | 31.4 |
| 2021 | Commonwealth | Deaths | 23.4 | 18.6 | 23.8 | 7.3 | 26.9 |
| 2021 | Middle SDI | Deaths | 33.1 | 10.3 | 12.8 | 8.3 | 35.5 |
| 2021 | Low SDI | Deaths | 24.8 | 26 | 21.3 | 6.4 | 21.6 |
| 2021 | Low-middle SDI | Deaths | 26.9 | 15.7 | 23.4 | 7.3 | 26.6 |
| 2021 | High SDI | Deaths | 30.1 | 10 | 25 | 2.1 | 32.8 |
| 2021 | World bank income levels | Deaths | 29.7 | 12.7 | 20.2 | 6.2 | 31.2 |
| 2021 | European Union | Deaths | 22 | 14.4 | 46.5 | 0.8 | 16.4 |
| 2021 | Unknown region | Deaths | 30.5 | 9.6 | 36.8 | 2.6 | 20.4 |
| 2021 | OECD Countries | Deaths | 28.5 | 12.8 | 28.4 | 2.2 | 28.1 |
| 2021 | Four World Regions | Deaths | 29.8 | 12.7 | 20.2 | 6.2 | 31.2 |
| 2021 | African Union | Deaths | 29.6 | 23.6 | 24.3 | 4.3 | 18.1 |
| 2021 | G20 | Deaths | 28.6 | 10.9 | 20.3 | 6 | 34 |
| 2021 | Sahel region | Deaths | 34.5 | 27.4 | 20.4 | 4.2 | 13.6 |
| 2021 | WHO region | Deaths | 29.7 | 12.8 | 20.3 | 6.2 | 31 |
| 2021 | World Bank regions | Deaths | 29.7 | 12.7 | 20.2 | 6.2 | 31.2 |
| 2021 | South Asia | Deaths | 19.3 | 14.2 | 26.6 | 8.8 | 31.1 |
| 2021 | North Africa and Middle East | Deaths | 32.8 | 7.9 | 35.6 | 2.3 | 21.4 |
| 2021 | Sub-Saharan Africa | Deaths | 28.9 | 29.9 | 18.4 | 5.2 | 17.6 |
| 2021 | Latin America and Caribbean | Deaths | 27.3 | 20.1 | 16.2 | 6 | 30.4 |
| 2021 | Southeast Asia, East Asia, and Oceania | Deaths | 38.4 | 3.4 | 2.2 | 11.1 | 45 |
| 2021 | Iceland | Deaths | 27.6 | 15.7 | 44.9 | 0.8 | 10.9 |
| 2021 | Central Europe, Eastern Europe, and Central Asia | Deaths | 14 | 24.4 | 42.8 | 4.8 | 14 |
| 2021 | High-income | Deaths | 29.6 | 10.5 | 28.9 | 1.5 | 29.5 |


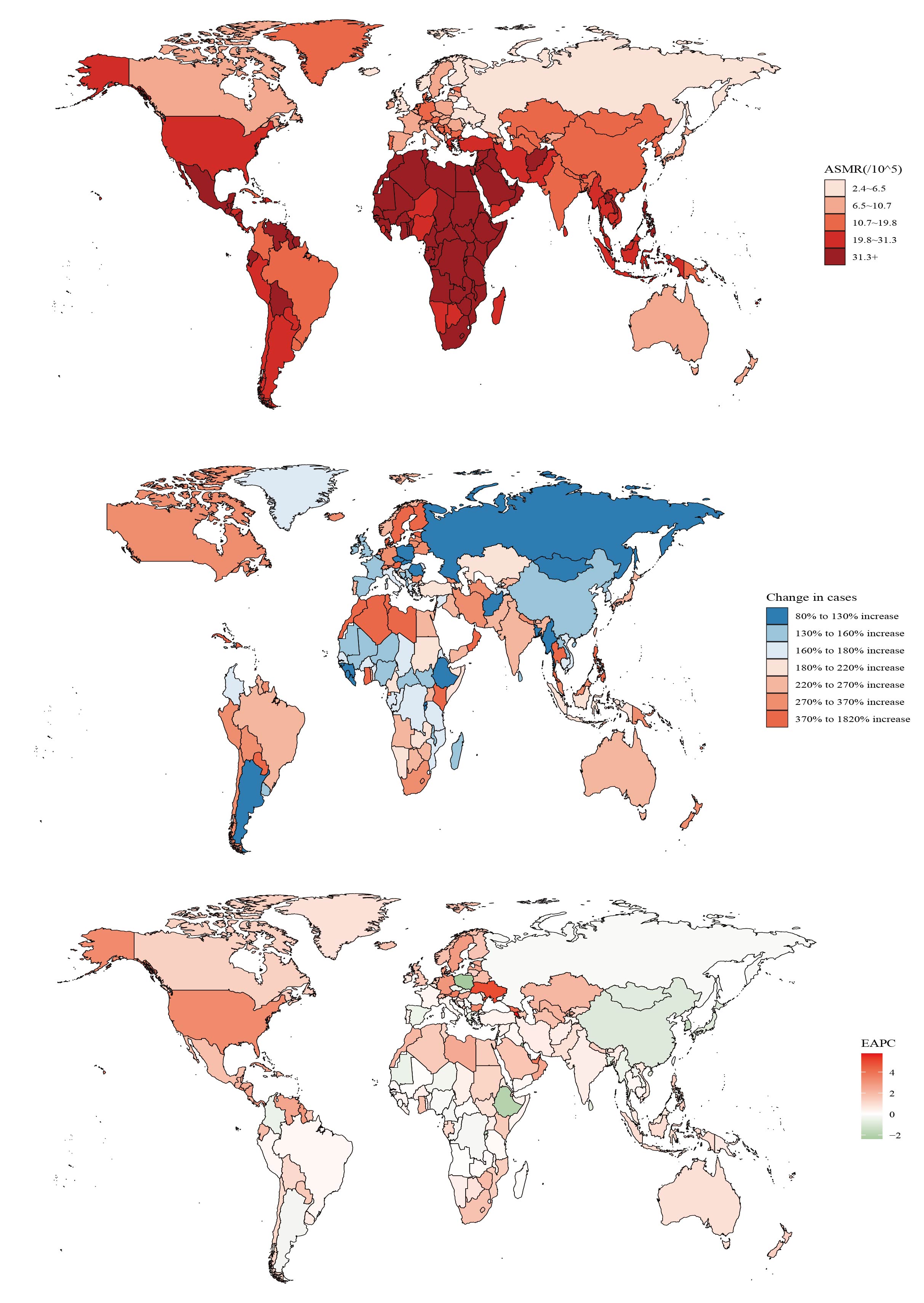


Figure S1. The global disease burden of CKD for both sexes in 204 countries and territories. (A) The ASMR of CKD in 2021; (B) The relative change in deaths of CKD between 1990 and 2021; (C) The EAPC of CKD ASMR from 1990 to 2021. ASMR, age standardized mortality rate; EAPC, estimated annual percentage change.
